# Trends in the full blood count blood test and colorectal cancer detection: a longitudinal, case-control study of UK primary care patient data

**DOI:** 10.3310/nihropenres.13266.1

**Published:** 2022-04-21

**Authors:** Pradeep S. Virdee, Julietta Patnick, Peter Watkinson, Jacqueline Birks, Tim A. Holt

**Affiliations:** 1 Nuffield Department of Primary Care Health Sciences, University of Oxford, Oxford, OX2 6GG, UK; 2 Nuffield Department of Population Health, University of Oxford, Oxford, OX3 7LF, UK; 3 Kadoorie Centre for Critical Care Research and Education, Oxford University Hospitals NHS Trust, Oxford, OX3 9DU, UK; 4 Centre for Statistics in Medicine, NDORMS, University of Oxford, Oxford, OX3 7LD, UK

**Keywords:** full blood count, blood test, primary care, colorectal cancer, clinical practice research datalink

## Abstract

**Background:**

The full blood count (FBC) is a common blood test performed in general practice. It consists of many individual parameters that may change over time due to colorectal cancer. Such changes are likely missed in practice. We identified trends in these FBC parameters to facilitate early detection of colorectal cancer.

**Methods:**

We performed a retrospective, case-control, longitudinal analysis of UK primary care patient data. LOWESS smoothing and mixed effects models were derived to compare trends in each FBC parameter between patients diagnosed and not diagnosed over a prior 10-year period.

**Results:**

There were 399,405 males (2.3%, n = 9,255 diagnosed) and 540,544 females (1.5%, n = 8,153 diagnosed) in the study. There was no difference between cases and controls in FBC trends between 10 and four years before diagnosis. Within four years of diagnosis, trends in many FBC levels statistically significantly differed between cases and controls, including red blood cell count, haemoglobin, white blood cell count, and platelets (interaction between time and colorectal cancer presence: p <0.05). FBC trends were similar between Duke’s Stage A and D colorectal tumours, but started around one year earlier in Stage D diagnoses.

**Conclusions:**

Trends in FBC parameters are different between patients with and without colorectal cancer for up to four years prior to diagnosis. Such trends could help earlier identification.

## Plain English summary

Colorectal cancer is a common type of cancer in the UK. It is the second most common cause of cancer-related death in the UK. Chances of surviving depend heavily on the tumour stage at diagnosis, which represents how much the tumour has developed. If diagnosed and treated at the earliest stage, where the tumour is confined to the colon, nine in 10 patients are expected to be alive five years later. If diagnosed at the latest stage, when the cancer has spread outside the colon, this drops to one in 10 surviving. The majority of UK patients with colorectal cancer are diagnosed with late-stage tumours, so are likely to die. Detecting and treating the cancer earlier can save lives.

There is a blood test called the Full Blood Count, which is commonly ordered by doctors for many reasons. This test includes many blood levels, such as haemoglobin, which carries oxygen around the body. Growing tumours cause subtle changes in the blood levels over time, but it is unclear what these changes are and if they could help find cancer in the early stages.

In our study, we looked at blood tests from almost one million patients in the UK, including around 17,000 with colorectal cancer. We checked how blood levels change over 10 years before diagnosis. We found that in the few years before patients are diagnosed, patients usually had blood levels that rapidly started increasing or declining (depending on the blood level) and this was often not seen in patients without colorectal cancer. Our study highlights that using trends over time in blood test results may be useful to identify colorectal cancer. Such trends could facilitate earlier detection because they were present for years before diagnosis. That would improve the chances of successful treatment and chances of survival.

## Introduction

Population incidence rates for colorectal cancer have been decreasing only slightly yearly since 2012. Colorectal cancer currently accounts for 11% of all new cancers diagnosed in the UK, being the fourth most common type of cancer. It is the second most common cause of cancer-related death. Prognosis is heavily influenced by tumour stage at diagnosis, which can be assessed in various ways. Five-year survival is 93% if diagnosed at Stage I, where the cancer is confined to the bowel lining, and 10% if at Stage IV, where it has spread to other organs ( Cancer Research UK - Bowel cancer statistics).

Symptoms for colorectal cancer, such as abdominal pain and change in bowel habit, often appear when the disease has developed to a relatively late-stage, where it is difficult to treat and the likelihood of survival reduced. Current evidence suggests that symptoms are on average first reported to clinical care less than six months before diagnosis ^1^. Identifying colorectal cancer at earlier stages, where the likelihood of survival is greatest and before overt symptoms appear, would be of considerable benefit to reduce mortality ^2^.

The full blood count (FBC), a blood test commonly performed in primary care practices, may play a role in earlier detection ^3^. For example, anaemia determined from the FBC test is a known risk factor for diagnosis and may warrant further investigation under the current screening programme if due to iron deficiency ( WHO: Guide to early diagnosis, NICE: Suspected cancer recognition and referral). The FBC test consists of up to 20 individual parameters, including haemoglobin, platelet count, and white blood cell count.

Our recent systematic review identified 53 studies that assessed the FBC blood test for colorectal cancer diagnosis ^4, 5^. Our review indicated that diagnosed patients have a significantly lower red blood cell count, haemoglobin, and mean corpuscular volume and higher red blood cell distribution width, white blood cell count, and platelets within six months of diagnosis compared to patients not diagnosed. Smaller differences were observed compared to those observed earlier than six months before diagnosis, suggesting changes in the FBC differ over time. There may be relevant trends that could help identify patients who have a diagnosis.

The aim of this study was to identify trends in FBC parameter levels prior to colorectal cancer diagnosis and compare trends to those in patients without a diagnosis. To identify opportunities for earlier detection, we also assessed trends between tumour stages in diagnosed patients.

## Methods

Study reporting follows the Strengthening the Reporting of Observational Studies in Epidemiology (STROBE) guidelines ^6^.

### Study design

We performed a retrospective case-control study to explore changes in FBC results before a diagnosis compared to patients without a diagnosis. FBC data were obtained from a UK primary care database, the Clinical Practice Research Datalink ( CPRD) GOLD, and diagnosis data from the UK National Cancer Registration and Analysis Service (NCRAS). CPRD has ethical approval from the Health Research Authority to hold anonymised patient data and support research using that data. The CPRD Independent Scientific Advisory Committee’s approval of data access for individual research projects includes ethics approval and consent for those projects. Ethical approval was therefore covered for this study by the CPRD (protocol 14_195RMn2A2R). Clinical codes to extract data from CPRD and NCRAS are in Table 1 and Table 2.

**Table 1.** Clinical codes relating to the full blood count from CPRD.

FBC parameter	Entity code	Medical code
Red blood cell count	194	17, 13788, 26931, 26932, 26933, 44213, 50182, 57136, 58853, 70079
White blood cell count	207	15, 1955, 3372, 4760, 4996, 13817, 13818, 18516, 22293, 26325, 26946, 26947, 26948, 38198, 45115, 48015, 48341, 53865, 92372
Haemoglobin	173	4, 739, 795, 3942, 10404, 13755, 26272, 26908, 26909, 26910, 26912, 26913, 33284, 35749, 39601, 41531
Haematocrit/packed cell volume	312	40, 99, 14240, 19836, 23476, 27143, 27144, 27145, 41478, 55365, 62347,
Mean corpuscular volume	182	10, 2480, 13774, 26920, 26921, 26922, 41160, 52874,
Mean corpuscular haemoglobin	180	20, 23214, 26917, 26918, 40170, 47174, 49225, 51616, 61951,
Mean corpuscular haemoglobin concentration	181	30, 26919, 39202, 47345, 55183, 64474, 72488
Red blood cell distribution width	361	64, 1191, 1962, 9933, 19837, 51484, 52050
Platelets	189	7, 3320, 4006, 4415, 26926, 26927, 37666
Mean platelet volume	-	14166
Basophil count	313	25, 27146, 27147, 27148, 53404
Eosinophil count	168	22, 13742, 18531, 26905, 26906
Lymphocyte count	208	19, 3189, 11240, 23120, 23121, 26949, 26950, 32932, 34551, 37677, 42346, 74019
Monocyte count	183	21, 9248, 13776, 26923, 26924, 26925, 44189, 72849
Neutrophil count	184	18, 4463, 13777, 15725, 23112, 23113, 31382, 105211,

**Table 2.** Clinical codes relating to colorectal cancer from NCRAS.

ICD-10 code	Term
C18	Malignant neoplasm of colon
C18.0	Caecum (incl.: Ileocaecal valve)
C18.1	Appendix
C18.2	Ascending colon
C18.3	Hepatic flexure
C18.4	Transverse colon
C18.5	Splenic flexure
C18.6	Descending colon
C18.7	Sigmoid colon (Incl.: Sigmoid (flexure); Excl.: rectosigmoid junction (C19))
C18.8	Overlapping lesion of colon
C18.9	Colon, unspecified (Incl.: Large intestine NOS)
C19	Malignant neoplasm of rectosigmoid junction (Incl.: Colon with rectum, Rectosigmoid (colon))
C20	Malignant neoplasm of rectum (Incl.: Rectal ampulla)
C21	Malignant neoplasm of anus and anal canal
C21.0	Anus, unspecified (Excl.: anal: margin (C43.5, C44.5), skin (C43.5, C44.5) perianal skin (C43.5, C44.5))
C21.1	Anal canal (Incl.: Anal sphincter)
C21.2	Cloacogenic zone
C21.8	Overlapping lesion of rectum, anus and anal canal (Incl.: Anorectal junction, Anorectum, and Malignant neoplasm of rectum, anus and anal canal whose point of origin cannot be classified to any one of the categories C20-C21.2)

Study entry was defined as the latest date of registration with the practice, patient’s 40 ^th^ birthday, or 1 ^st^ January 2000. Study exit was defined as the earliest date of leaving the practice, date of death, or 14 ^th^ January 2014 (the NCRAS data-cut date).

### Participants

Patients with at least one FBC blood test within 10 years before index date (defined below) were included. Patients were excluded if registered with their primary care practice for less than one year, had a history of colorectal cancer before study entry, or diagnosed after study exit. Patients diagnosed with another cancer type before or simultaneously with colorectal cancer diagnosis were excluded. Patients with an available date of diagnosis but no indication of the cancer type were excluded.

### Clinical outcome

The outcome was the first diagnosis of colorectal cancer in the NCRAS database. For cases (patients diagnosed), the index date was the date of colorectal cancer diagnosis in the patient’s study period. For controls (patients without a diagnosis), the index date was a randomly selected date in the patient’s study period. A random date was chosen to mimic the sporadic nature of diagnoses in the overall study period for cases.

### Demographic and FBC variables

Year of birth and sex were available for all patients in the CPRD dataset. We extracted the date of each FBC test and included 14 of the 20 parameters (exposure variables of interest) that make up the FBC in this study. We excluded five: percentage basophils, eosinophils, lymphocytes, monocytes, and neutrophils because we used the corresponding counts (also FBC parameters). Additionally, red blood cell distribution width was excluded because this parameter is not recorded in general practice so was missing for almost all FBCs. We excluded FBC results outside biologically plausible ranges, such as negative values (see Table 3 for further details), FBCs performed earlier than 10 years before index date, and FBCs performed after index date.

**Table 3.** Details of the full blood count blood test parameters included in the analysis.

Full blood count parameter	Biologically plausible range	Number (%) missing ^1^	
		Males (n=1,193,619 FBCs)	Females (n=1,874,609 FBCs)
Red blood cell count (10 ^12^/L)	2.5 – 7.5	103,607 (8.7%)	165,608 (8.8%)
Haemoglobin (g/dL)	0.3 – 21.0	22,637 (1.9%)	34,678 (1.8%)
Haematocrit/packed cell volume (L/L)	0.1 – 0.6	968,246 (81.1%)	1,514,243 (80.8%)
Mean corpuscular volume (fL)	53 – 125	57,807 (4.8%)	86,606 (4.6%)
Mean corpuscular haemoglobin (pg)	22 – 36	92,460 (7.7%)	150,566 (8.0%)
Mean corpuscular haemoglobin concentration (g/dL)	27 – 37	165,983 (13.9%)	262,094 (14.0%)
Red cell distribution width (%)	9 – 15	1,193,587 (100%)	1,874,530 (100%)
Platelet count (10 ^9^/L)	0 – 1500	71,385 (6.0%)	114,575 (6.1%)
Mean platelet volume (fL)	5 – 14	1,040,399 (87.2%)	1,630,832 (87.0%)
White blood cell count (10 ^9^/L)	0 – 850	223,059 (18.7%)	351,858 (18.8%)
Basophil count (10 ^9^/L)	0 – 1.5	326,914 (27.4%)	535,356 (28.6%)
Eosinophil count (10 ^9^/L)	0 – 8	322,907 (27.1%)	529,122 (28.2%)
Lymphocyte count (10 ^9^/L)	0 – 850	289,640 (24.3%)	457,255 (24.4%)
Monocyte count (10 ^9^/L)	0 – 40	298,940 (25.0%)	473,324 (25.2%)
Neutrophil count (10 ^9^/L)	0 – 850	285,027 (23.9%)	451,291 (24.1%)

^1^This is missing data that remained after applying known mathematical equations that can be used to derive many components.

Iron-deficiency anaemia warrants further investigation for colorectal cancer. Anaemia was defined as haemoglobin level <13 g/dL for men and <12 g/dL for women, as recommended by the National Institute for Health and Care Excellence (NICE) ( NICE: Suspected cancer recognition and referral, NICE: anaemia - iron deficiency) and World Health Organisation (WHO) ( WHO: anaemia). Microcytic anaemia, commonly present in iron-deficiency anaemia ( Mean Corpuscular Volume), was defined as the presence of microcytosis (mean corpuscular volume <80 fL, as recommended by NICE ( NICE: Investigations to confirm iron deficiency anaemia)) with anaemia.

Each FBC parameter has a normal or reference range of values considered to reflect a healthy individual ( Mayo Clinic: Complete blood count). Reference ranges differ based on age and sex and may differ slightly across primary care practices ( Royal Wolverhampton NHS UK, York Hospitals NHS UK, Maidstone and Tunbridge Wells NHS UK, Gloucestershire Hospitals NHS UK). We used reference ranges provided by the Department of Laboratory Haematology at Oxford University Hospitals Trust in this study ( Table 4) ( Oxford University Hospitals NHS UK) for comparison with trends.

**Table 4.** Normal reference range for patients aged 13+ years for each FBC parameter.

FBC parameter	Males normal range	Females normal range
Red blood cell count (10 ^9^/L)	4.5–5.5	3.8–4.8
Haemoglobin (g/dL)	13–17	12–15
Haematocrit (L/L)	0.40–0.50	0.36–0.46
Mean corpuscular volume (fL)	83–101	83–101
Mean corpuscular haemoglobin (pg)	27–32	27–32
Mean corpuscular haemoglobin concentration (g/dL)	31.5–34.5	31.5–34.5
Platelets (1012/L)	150–400	150–400
Mean platelet volume (fL)	9.0–12.1	9.0–12.1
White blood cell count (10 ^12^/L)	4–11	4–11
Basophil count (10 ^12^/L)	0.02–0.10	0.02–0.10
Eosinophil count (10 ^12^/L)	0.02–0.50	0.02–0.50
Lymphocyte count (10 ^12^/L)	1–4	1–4
Monocyte count (10 ^12^/L)	0.2–1.0	0.2–1.0
Neutrophil count (10 ^12^/L)	2–7	2–7

A detailed account of our data preparation and validation processes has previously been reported ^7^. We also previously provided summary statistics for each FBC parameter.

### Statistical analysis

We used LOWESS smoothing to describe trends in FBC parameters graphically for 10-yearly age groups. Controls with many FBCs are likely to have some other condition/disease, which could affect blood levels. Therefore, for both cases and controls, we randomly selected three FBCs per patient (if there were more than three) to reduce the influence of these many effected FBCs on LOWESS trends.

Mixed effects models were developed for each FBC parameter separately (using all available FBCs per patient), using restricted maximum likelihood estimation. To model differences in FBC levels between cases and controls over time, colorectal cancer status (yes/no) and time to index date (years) were included as fixed effects together with an interaction between them. Each model was adjusted for age at index date (years) as a fixed effect and interactions between age and time and age and colorectal cancer status were included if trends over time or by colorectal cancer status differed by age group upon graphical inspection of LOWESS plots. Each patient was modelled using a random intercept and time using a random slope with an unstructured covariance matrix to account for correlation in repeated measures.

Non-linearity of continuous variables was based on visual inspection, Akaike information criteria, and Bayesian information criteria, which compared linear splines, restricted cubic splines, and fractional polynomials, and number of knots and knot locations ^8– 10^. Where non-linear, time to index date was modelled using piecewise linear splines with three knots: at one, two, and four years before index date. Age at index date was modelled using piecewise linear splines with knots at ages 60, 70, and 80 years for red blood cell-related parameters and platelet count and were variable for white blood cell count-related parameters.

To identify opportunities for early detection, we assessed differences in FBC levels over time between cases diagnosed at Duke’s tumour Stage A (earliest stage) and D (latest stage). Mixed effects models were developed using the same methods described above but were limited to cases alone and included Duke’s tumour stage (A versus D) accordingly as fixed effects instead of colorectal cancer status. To explore the association between microcytic anaemia and diagnosis, we calculated the proportion of patients with microcytic anaemia per six-monthly time band, up to five years before index date. Microcytic anaemia presence was based on any FBC in the time band, if a patient had multiple, and proportions were calculated out of the number of patients in the time band. Additionally, we derived age-adjusted odds ratios (95% confidence interval (CI)) for microcytic anaemia presence using logistic regression for each time band separately. We visually compared trends to microcytic anaemia thresholds and FBC reference ranges to identify whether trends can pre-date single-value, iron-deficiency referral thresholds and blood-abnormality.

All analyses were stratified by sex. A two-sided significance level of 5% was used for all statistical analyses. Analyses were conducted using Stata/SE 15.1 (RRID: SCR_012763). Alternative, open-access software, such as R (RRID: SCR_001905), can perform the equivalent analyses.

### Sensitivity analysis

We recreated the trends and mixed effects models for each FBC parameter using a matched design. Cases and controls were matched 1:5 on age at index and follow-up time. Follow-up was time (years) from first FBC to index, converted into six-monthly bands for matching. A random index date within the control’s study period was used instead of the index date of their matched case because the latter heavily reduced the sample size. For example, many controls had an index date after study exit or had no FBCs before index.

## Results

**Figure 1.**
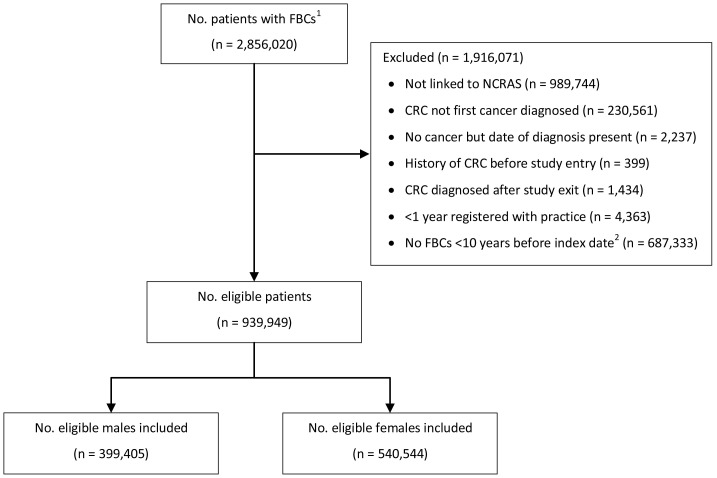
Patient flow chart. ^1^Number of patients available in the CPRD data extract. ^2^Index date was the date of diagnosis for cases and a randomly selected date in the patient’s study period for controls. Abbreviations: FBC = full blood count; CRC = colorectal cancer; NCRAS = National Cancer Registration and Analysis Service.

We identified 939,949 patients with at least one FBC within 10 years before index date who satisfied the eligibility criteria and were included in the study ( Figure 1). There were 399,405 males with 1,193,619 FBC tests among them and 540,544 females with 1,874,609 FBC tests among them. See Table 5 for a description of the patient sample.

**Table 5.** Characteristics of the patient sample.

	Diagnosis of colorectal cancer			No diagnosis of colorectal cancer		
	Number	Mean age ^1^ (SD)	Age ^1^ range	Number	Mean age ^1^ (SD)	Age ^1^ range
**Male**	9,255	70.8 (10.6)	40 – 101	390,150	59.5 (12.9)	40 – 111
**Female**	8,153	73.0 (11.8)	40 – 101	532,391	60.6 (14.7)	40 – 109
**Total**	17,408	71.8 (11.2)	40 – 101	922,541	60.1 (14.0)	40 – 111

^1^Age at index date: date of diagnosis (cases) or a randomly selected date in the patient’s study period (controls)

### The FBC test

On average, 14 of the 15 parameters were available within a FBC across FBCs for both males and females separately. Red blood cell distribution width was the FBC parameter missing for almost 100% of FBC tests for both males and females. This is likely because this parameter was historically not reported to the general practice by haematology laboratories, despite being automatically derived by haematology analysers (i.e. machines). Consequently, red blood cell distribution width was excluded from further analyses. Haemoglobin had the least amount of missing data, missing for 1.9% (n = 22,637) and 1.8% (n = 34,678) of FBC tests for males and females, respectively. Missing data for each parameter are provided in Table 3.

### Colorectal cancer and the FBC

**Figure 2.**
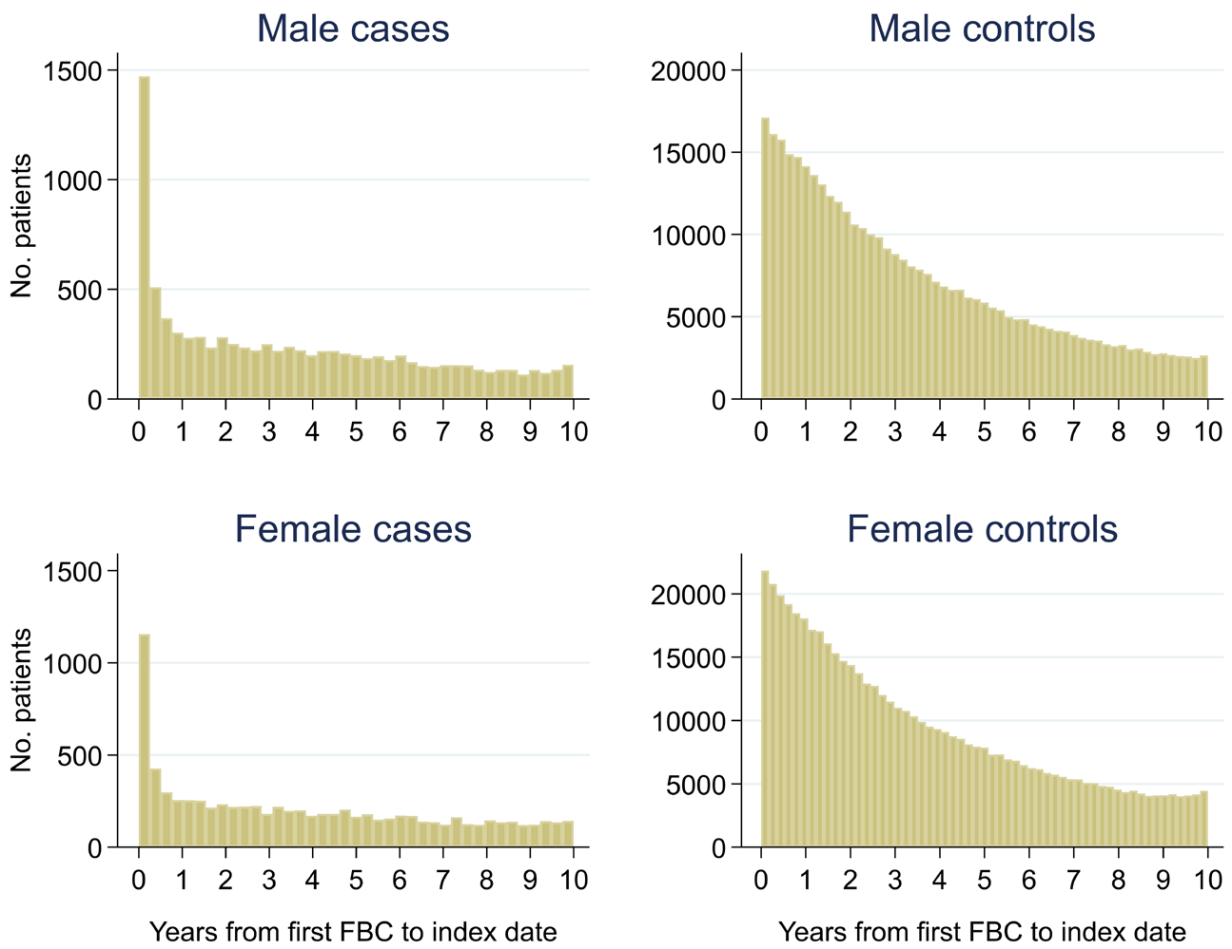
Histogram of follow-up time from first FBC to index date ^1,2^. ^1^Index date was the date of diagnosis for cases and a randomly selected date in the patient’s study period for controls. ^2^The spike at time=0 in cases is likely due to patients undergoing cancer investigation. This was not expected to influence trends, as the trends rely on sufficient data at each time-point, not comparability of follow-up.

**Figure 3.**
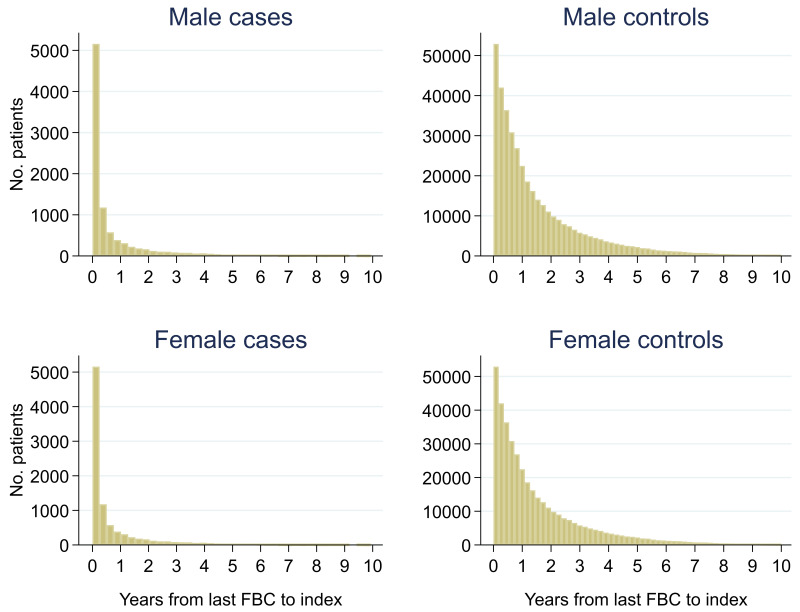
Histogram of time between last FBC and index date ^1,2^. ^1^Index date was the date of diagnosis for cases and a randomly selected date in the patient’s study period for controls. ^2^The spike at time=0 in cases is likely due to patients undergoing cancer investigation. This was not expected to influence trends, as the trends rely on sufficient data at each time-point, not comparability of follow-up.

Of 399,405 males, 2.3% (n = 9,255) had a colorectal cancer diagnosis and of 540,544 females, 1.5% (n = 8,153) were diagnosed. Median (min, max) follow-up was 3.0 (0, 10.0) for male cases, 2.7 (0, 10.0) for male controls, 3.3 (0, 10.0) for female cases and 2.8 (0, 10.0) for female controls ( Figure 2). Median (min, max) time between the last FBC and index date was 0.2 (0, 9.9) years for male cases, 1.0 (0, 10.0) years for male controls, 0.2 (0, 10.0) years for female cases and 0.9 (0, 10.0) years for female controls ( Figure 3).

Mixed models for red blood cell-related parameters are in Table 6 (males) and Table 7 (females), platelet-related in Table 8, and white blood cell count-related in Table 9 (males) and Table 10 (females). The presence of colorectal cancer was statistically significantly associated with all parameter levels (p <0.05 for each model) except white blood cell count and eosinophil count for both males and females.

**Table 6.** Mixed effect model coefficients for red blood cell count parameters - males.

Variable	RBC	Hb	Hc	MCV	MCH	MCHC
*N*	*376516*	*394601*	*93128*	*388464*	*380868*	*360071*
*n cases*	*9024*	*9161*	*2256*	*8792*	*8631*	*8363*
**Fixed effects:**						
Constant	5.2721 (5.2458, 5.2984)	15.6416 (15.5582, 15.7249)	0.4627 (0.4577, 0.4677)	86.8880 (86.7174, 87.0586)	29.6513 (29.5910, 29.7115)	34.0711 (34.0369, 34.1054)
Age at index date ^1^ (years)	-0.0077 (-0.0082, -0.0072)	-0.0142 (-0.0158, -0.0126)	-0.0004 (-0.0005, -0.0003)	0.0777 (0.0744, 0.0810)	0.0194 (0.0183, 0.0206)	-0.0067 (-0.0074, -0.0061)
Age at index date ^1^ – knot at 60 (years)	-0.0037 (-0.0051, -0.0024)	-0.0307 (-0.0350, -0.0264)	-0.0008 (-0.0010, -0.0005)	-0.0551 (-0.0642, -0.0461)	-0.0281 (-0.0313, -0.0250)	-0.0117 (-0.0134, -0.0100)
Age at index date ^1^ – knot at 70 (years)	-0.0127 (-0.0147, -0.0107)	-0.0326 (-0.0388, -0.0263)	-0.0007 (-0.0011, -0.0003)	0.0094 (-0.0044, 0.0233)	0.0010 (-0.0039, 0.0059)	-0.0043 (-0.0069, -0.0016)
Age at index date ^1^ – knot at 80 (years)	-0.0027 (-0.0050, -0.0003)	-0.0040 (-0.0115, 0.0034)	-0.0004 (-0.0009, 0.0000)	0.0289 (0.0112, 0.0465)	0.0080 (0.0019, 0.0143)	0.0021 (-0.0012, 0.0054)
Time to index date ^1^ (years)	0.0148 (-0.0197, 0.0493)	-0.0125 (-0.1250, 0.1000)	-0.0014 (-0.0083, 0.0056)	-0.1748 (-0.2074, -0.1422)	-0.0137 (-0.0258, -0.0017)	0.0421 (0.0324, 0.0519)
Time to index date ^1^ date – knot at one (years)	-0.0107 (-0.0673, 0.0460)	-0.0014 (-0.1867, 0.1839)	0.0026 (-0.0089, 0.0142)	0.0625 (0.0108, 0.1141)	-0.0018 (-0.0209, 0.0173)	-0.0205 (-0.0361, -0.0049)
Time to index date ^1^ date – knot at two (years)	0.0054 (-0.0348, 0.0456)	-0.0063 (-0.1378, 0.1252)	-0.0047 (-0.0131, 0.0037)	-0.0148 (-0.0481, 0.0185)	0.0257 (0.0135, 0.0381)	0.0298 (0.0197, 0.0400)
Time to index date ^1^ date – knot at four (years)	-0.0395 (-0.0646, -0.0144)	-0.0437 (-0.1257, 0.0383)	0.0038 (-0.0017, 0.0094)	0.1234 (0.1068, 0.1400)	0.0037 (-0.0024, 0.0098)	-0.0324 (-0.0374, -0.0274)
CRC present	-0.2529 (-0.4629, -0.0428)	-2.5579 (-3.1632, -1.9526)	-0.0740 (-0.1091, -0.0389)	-8.0698 (-10.5710, -5.5686)	-2.1303 (-3.0312, -1.2293)	-1.7476 (-2.2370, -1.2582)
CRC present by time to index date ^1^ interaction	0.1869 (0.1732, 0.2005)	1.4226 (1.3771, 1.4682)	0.0321 (0.0293, 0.0350)	4.5147 (4.3692, 4.6603)	1.4979 (1.4426, 1.5532)	0.7227 (0.6773, 0.7681)
CRC present by time to index date ^1^ (knot at one) interaction	-0.1625 (-0.1868, -0.1381)	-1.2030 (-1.2841, -1.1220)	-0.0246 (-0.0298, -0.0195)	-3.9394 (-4.1983, -3.6805)	-1.2412 (-1.3386, -1.1438)	-0.6063 (-0.6876, -0.5250)
CRC present by time to index date ^1^ (knot at two) interaction	-0.0208 (-0.0384, -0.0033)	-0.1037 (-0.1622, -0.0451)	-0.0050 (-0.0088, -0.0011)	-0.1012 (-0.2886, 0.0862)	-0.0784 (-0.1483, -0.0086)	-0.0287 (-0.0876, 0.0302)
CRC present by time to index date ^1^ (knot at four) interaction	-0.0035 (-0.0120, 0.0051)	-0.1046 (-0.1332, -0.0761)	-0.0020 (-0.0041, 0.0000)	-0.4093 (-0.5016, -0.3170)	-0.1545 (-0.1888, -0.1201)	-0.0828 (-0.1114, -0.0542)
Time by age interaction	-0.0000 (-0.0007, 0.0006)	0.0007 (-0.0015, 0.0028)	0.0000 (-0.0001, 0.0002)			
Time (knot at one) by age interaction	0.0003 (-0.0008, 0.0013)	0.0003 (-0.0032, 0.0038)	-0.0001 (-0.0003, 0.0002)			
Time (knot at two) by age interaction	-0.0002 (-0.0009, 0.0006)	0.0000 (-0.0025, 0.0025)	0.0001 (-0.0001, 0.0003)			
Time (knot at four) by age interaction	0.0006 (0.0001, 0.0010)	0.0006 (-0.0009, 0.0021)	-0.0001 (-0.0002, 0.0000)			
Time by age (knot at 60) interaction	0.0016 (-0.0000, 0.0033)	0.0065 (0.0011, 0.0120)	0.0003 (-0.0001, 0.0006)			
Time (knot at one) by age (knot at 60) interaction	-0.0019 (-0.0046, 0.0007)	-0.0067 (-0.0155, 0.0021)	-0.0002 (-0.0007, 0.0004)			
Time (knot at two) by age (knot at 60) interaction	0.0011 (-0.0006, 0.0029)	0.0032 (-0.0027, 0.0090)	-0.0001 (-0.0005, 0.0003)			
Time (knot at four) by age (knot at 60) interaction	-0.0008 (-0.0018, 0.0001)	-0.0019 (-0.0050, 0.0012)	0.0001 (-0.0001, 0.0003)			
Time by age (knot at 70) interaction	0.0009 (-0.0014, 0.0031)	0.0003 (-0.0073, 0.0078)	-0.0002 (-0.0007, 0.0003)			
Time (knot at one) by age (knot at 70) interaction	0.0029 (-0.0007, 0.0065)	0.0095 (-0.0024, 0.0215)	0.0004 (-0.0004, 0.0011)			
Time (knot at two) by age (knot at 70) interaction	-0.0035 (-0.0059, -0.0012)	-0.0109 (-0.0186, -0.0033)	-0.0002 (-0.0007, 0.0003)			
Time (knot at four) by age (knot at 70) interaction	0.0006 (-0.0005, 0.0018)	0.0025 (-0.0012, 0.0062)	0.0000 (-0.0003, 0.0003)			
Time by age (knot at 80) interaction	-0.0011 (-0.0037, 0.0015)	-0.0047 (-0.0135, 0.0041)	0.0000 (-0.0006, 0.0006)			
Time (knot at one) by age (knot at 80) interaction	-0.0028 (-0.0070, 0.0014)	-0.0092 (-0.0232, 0.0048)	-0.0005 (-0.0014, 0.0004)			
Time (knot at two) by age (knot at 80) interaction	0.0048 (0.0021, 0.0075)	0.0199 (0.0108, 0.0290)	0.0006 (0.0000, 0.0012)			
Time (knot at four) by age (knot at 80) interaction	-0.0014 (-0.0027, -0.0000)	-0.0078 (-0.0121, -0.0034)	-0.0001 (-0.0004, 0.0002)			
CRC presence by age interaction	0.0004 (-0.0034, 0.0041)	0.0129 (0.0021, 0.0237)	0.0005 (-0.0001, 0.0011)	0.0441 (-0.0004, 0.0887)	0.0059 (-0.0101, 0.0219)	0.0141 (0.0054, 0.0228)
CRC presence by age (knot at 60) interaction	-0.0003 (-0.0066, 0.0061)	-0.0241 (-0.0422, -0.0059)	-0.0010 (-0.0021, 0.0001)	-0.1196 (-0.1951, -0.0441)	-0.0385 (-0.0656, -0.0115)	-0.0250 (-0.0395, -0.0104)
CRC presence by age (knot at 70) interaction	0.0053 (-0.0008, 0.0115)	0.0155 (-0.0020, 0.0330)	0.0011 (0.0001, 0.0022)	-0.0011 (-0.0747, 0.0724)	0.0161 (-0.0100, 0.0423)	0.0116 (-0.0022, 0.0255)
CRC presence by age (knot at 80) interaction	-0.0094 (-0.0160, -0.0027)	-0.0128 (-0.0319, 0.0063)	-0.0013 (-0.0023, -0.0002)	0.0924 (0.0114, 0.1734)	0.0014 (-0.0272, 0.0301)	-0.0124 (-0.0274, 0.0026)
**Random effects:**						
Intercept for patient (variance)	0.1740 (0.1729, 0.1751)	1.4811 (1.4713, 1.4909)	0.0012 (0.0011, 0.0012)	25.9019 (25.7479, 26.0569)	3.1215 (3.1022, 3.1409)	0.7548 (0.7481, 0.7615)
Slope for time to index date ^1^ (variance)	0.0020 (0.0019, 0.0020)	0.0248 (0.0244, 0.0252)	0.0000 (0.0000, 0.0000)	0.3127 (0.3078, 0.3178)	0.0384 (0.0378, 0.0390)	0.0137 (0.0134, 0.0141)
Covariance between Intercept for patient and slope for time to index date (covariance)	-0.0097 (-0.0099, -0.0095)	-0.1099 (-0.1117, -0.1082)	-0.0001 (-0.0001, -0.0001)	-1.3578 (-1.3820, -1.3336)	-0.1680 (-0.1711, -0.1650)	-0.0622 (-0.0635, -0.0609)
Residual (variance)	0.0506 (0.0505, 0.0508)	0.6220 (0.6200, 0.6241)	0.0005 (0.0005, 0.0005)	6.0476 (6.0268, 6.0685)	0.8071 (0.8043, 0.8100)	0.5825 (0.5804, 0.5845)

^1^Index date was the date of diagnosis for cases and a randomly selected date in the patient’s study period for controls. Abbreviations: RBC = red blood cells; Hb = haemoglobin; Hc = haematocrit; MCV = mean corpuscular volume; MCH = mean corpuscular haemoglobin; MCHC = mean corpuscular haemoglobin concentration.

**Table 7.** Mixed effect model coefficients for red blood cell count parameters - females.

Variable	RBC	Hb	Hc	MCV	MCH	MCHC
*N*	509513	534243	129276	526287	514876	488199
*n cases*	7976	8070	2052	7745	7567	7396
**Fixed effects:**						
Constant	4.1865 (4.1675, 4.2056)	12.1175 (12.0546, 12.1803)	0.3706 (0.3668, 0.3743)	87.1667 (87.0087, 87.3246)	29.1787 (29.1224, 29.2350)	33.0309 (33.0000, 33.0618)
Age at index date ^1^ (years)	0.0050 (0.0047, 0.0054)	0.0238 (0.0225, 0.0250)	0.0006 (0.0005, 0.0007)	0.0649 (0.0617, 0.0680)	0.0182 (0.0171, 0.0193)	0.0033 (0.0027, 0.0039)
Age at index date ^1^ – knot at 60 (years)	-0.0107 (-0.0117, -0.0096)	-0.0503 (-0.0538, -0.0467)	-0.0012 (-0.0014, -0.0009)	-0.0621 (-0.0710, -0.0532)	-0.0264 (-0.0295, -0.0232)	-0.0113 (-0.0130, -0.0096)
Age at index date ^1^ – knot at 70 (years)	-0.0079 (-0.0094, -0.0063)	-0.0188 (-0.0238, -0.0138)	-0.0007 (-0.0010, -0.0004)	-0.0023 (-0.0154, 0.0108)	-0.0047 (-0.0093, -0.0000)	-0.0088 (-0.0112, -0.0063)
Age at index date ^1^ – knot at 80 (years)	-0.0009 (-0.0024, 0.0006)	0.0014 (-0.0036, 0.0064)	0.0001 (-0.0002, 0.0004)	0.0190 (0.0056, 0.0324)	0.0060 (0.0012, 0.0108)	0.0014 (-0.0011, 0.0039)
Time to index date ^1^ (years)	-0.0161 (-0.0404, 0.0082)	-0.2697 (-0.3529, -0.1865)	-0.0111 (-0.0161, -0.0060)	-0.3001 (-0.3289, -0.2712)	-0.0532 (-0.0637, -0.0427)	0.0267 (0.0186, 0.0347)
Time to index date ^1^ date – knot at one (years)	-0.0063 (-0.0461, 0.0335)	0.2064 (0.0697, 0.3430)	0.0101 (0.0017, 0.0185)	0.1804 (0.1348, 0.2261)	0.0352 (0.0186, 0.0518)	-0.0103 (-0.0231, 0.0025)
Time to index date ^1^ date – knot at two (years)	-0.0215 (-0.0496, 0.0066)	-0.1740 (-0.2709, -0.0772)	-0.0072 (-0.0133, -0.0011)	0.0340 (0.0045, 0.0635)	0.0399 (0.0291, 0.0506)	0.0349 (0.0266, 0.0432)
Time to index date ^1^ date – knot at four (years)	0.0420 (0.0248, 0.0593)	0.2626 (0.2031, 0.3221)	0.0085 (0.0046, 0.0125)	0.0727 (0.0580, 0.0873)	-0.0073 (-0.0126, -0.0019)	-0.0373 (-0.0414, -0.0332)
CRC present	-0.2530 (-0.4467, -0.0593)	-2.0165 (-2.6006, -1.4324)	-0.0508 (-0.0843, -0.0173)	-6.9632 (-9.7568, -4.1697)	-2.3591 (-3.3783, -1.3399)	-1.1348 (-1.6733, -0.5962)
CRC present by time to index date ^1^ interaction	0.1643 (0.1517, 0.1769)	1.3311 (1.2874, 1.3748)	0.0366 (0.0337, 0.0394)	4.8123 (4.6482, 4.9764)	1.6663 (1.6043, 1.7283)	0.6327 (0.5858, 0.6796)
CRC present by time to index date ^1^ (knot at one) interaction	-0.1247 (-0.1468, -0.1025)	-1.0582 (-1.1352, -0.9813)	-0.0303 (-0.0354, -0.0253)	-4.1124 (-4.4004, -3.8244)	-1.3530 (-1.4605, -1.2454)	-0.4455 (-0.5282, -0.3628)
CRC present by time to index date ^1^ (knot at two) interaction	-0.0336 (-0.0493, -0.0178)	-0.1575 (-0.2123, -0.1026)	-0.0047 (-0.0083, -0.0011)	-0.1472 (-0.3527, 0.0583)	-0.1389 (-0.2148, -0.0630)	-0.1358 (-0.1951, -0.0766)
CRC present by time to index date ^1^ (knot at four) interaction	-0.0053 (-0.0130, 0.0023)	-0.0888 (-0.1152, -0.0624)	-0.0012 (-0.0031, 0.0007)	-0.4000 (-0.5002, -0.2999)	-0.1338 (-0.1708, -0.0969)	-0.0112 (-0.0400, 0.0176)
Time by age interaction	0.0003 (-0.0002, 0.0007)	0.0043 (0.0027, 0.0059)	0.0002 (0.0001, 0.0003)			
Time (knot at one) by age interaction	0.0001 (-0.0007, 0.0009)	-0.0037 (-0.0064, -0.0010)	-0.0002 (-0.0003, -0.0000)			
Time (knot at two) by age interaction	0.0004 (-0.0001, 0.0010)	0.0038 (0.0019, 0.0056)	0.0001 (0.0000, 0.0003)			
Time (knot at four) by age interaction	-0.0010 (-0.0013, -0.0006)	-0.0056 (-0.0067, -0.0044)	-0.0002 (-0.0002, -0.0001)			
Time by age (knot at 60) interaction	0.0010 (-0.0003, 0.0023)	0.0015 (-0.0029, 0.0060)	0.0001 (-0.0002, 0.0003)			
Time (knot at one) by age (knot at 60) interaction	-0.0002 (-0.0023, 0.0018)	0.0039 (-0.0032, 0.0110)	0.0001 (-0.0004, 0.0005)			
Time (knot at two) by age (knot at 60) interaction	-0.0005 (-0.0019, 0.0009)	-0.0061 (-0.0108, -0.0014)	-0.0002 (-0.0005, 0.0001)			
Time (knot at four) by age (knot at 60) interaction	0.0015 (0.0008, 0.0022)	0.0100 (0.0076, 0.0124)	0.0003 (0.0001, 0.0005)			
Time by age (knot at 70) interaction	0.0000 (-0.0017, 0.0017)	-0.0025 (-0.0085, 0.0035)	-0.0002 (-0.0006, 0.0002)			
Time (knot at one) by age (knot at 70) interaction	0.0002 (-0.0025, 0.0030)	-0.0002 (-0.0098, 0.0094)	0.0003 (-0.0003, 0.0009)			
Time (knot at two) by age (knot at 70) interaction	-0.0006 (-0.0023, 0.0012)	0.0010 (-0.0051, 0.0072)	-0.0000 (-0.0004, 0.0003)			
Time (knot at four) by age (knot at 70) interaction	-0.0002 (-0.0011, 0.0006)	-0.0041 (-0.0070, -0.0011)	-0.0001 (-0.0003, 0.0001)			
Time by age (knot at 80) interaction	-0.0003 (-0.0020, 0.0014)	-0.0044 (-0.0103, 0.0016)	-0.0000 (-0.0004, 0.0004)			
Time (knot at one) by age (knot at 80) interaction	-0.0004 (-0.0031, 0.0023)	0.0047 (-0.0048, 0.0141)	-0.0001 (-0.0007, 0.0005)			
Time (knot at two) by age (knot at 80) interaction	0.0006 (-0.0012, 0.0023)	-0.0012 (-0.0073, 0.0049)	-0.0001 (-0.0005, 0.0003)			
Time (knot at four) by age (knot at 80) interaction	-0.0004 (-0.0013, 0.0004)	0.0002 (-0.0027, 0.0031)	0.0002 (-0.0000, 0.0004)			
CRC presence by age interaction	0.0010 (-0.0025, 0.0045)	0.0057 (-0.0048, 0.0163)	0.0002 (-0.0004, 0.0008)	0.0151 (-0.0355, 0.0657)	0.0069 (-0.0115, 0.0254)	0.0051 (-0.0046, 0.0149)
CRC presence by age (knot at 60) interaction	-0.0030 (-0.0095, 0.0034)	-0.0255 (-0.0449, -0.0061)	-0.0010 (-0.0021, 0.0001)	-0.0800 (-0.1733, 0.0132)	-0.0370 (-0.0710, -0.0031)	-0.0188 (-0.0366, -0.0010)
CRC presence by age (knot at 70) interaction	0.0027 (-0.0036, 0.0090)	0.0163 (-0.0026, 0.0352)	0.0009 (-0.0002, 0.0019)	0.0025 (-0.0892, 0.0943)	0.0068 (-0.0264, 0.0400)	0.0083 (-0.0088, 0.0255)
CRC presence by age (knot at 80) interaction	-0.0006 (-0.0060, 0.0049)	-0.0038 (-0.0200, 0.0124)	-0.0001 (-0.0011, 0.0008)	0.0359 (-0.0429, 0.1147)	0.0080 (-0.0205, 0.0364)	-0.0027 (-0.0173, 0.0119)
**Random effects:**						
Intercept for patient (variance)	0.1372 (0.1365, 0.1380)	1.2528 (1.2456, 1.2601)	0.0010 (0.0010, 0.0010)	29.6896 (29.5368, 29.8432)	3.7164 (3.6968, 3.7362)	0.9040 (0.8976, 0.9104)
Slope for time to index date ^1^ (variance)	0.0015 (0.0015, 0.0016)	0.0212 (0.0209, 0.0215)	0.0000 (0.0000, 0.0000)	0.4220 (0.4166, 0.4276)	0.0538 (0.0531, 0.0545)	0.0174 (0.0171, 0.0177)
Covariance between Intercept for patient and slope for time to index date (covariance)	-0.0071 (-0.0072, -0.0069)	-0.0829 (-0.0842, -0.0816)	-0.0001 (-0.0001, -0.0001)	-1.4631 (-1.4882, -1.4380)	-0.1961 (-0.1994, -0.1929)	-0.0713 (-0.0725, -0.0701)
Residual (variance)	0.0447 (0.0446, 0.0449)	0.5960 (0.5945, 0.5976)	0.0005 (0.0004, 0.0005)	7.9522 (7.9311, 7.9734)	1.0205 (1.0178, 1.0233)	0.6239 (0.6222, 0.6256)

^1^Index date was the date of diagnosis for cases and a randomly selected date in the patient’s study period for controls. Abbreviations: RBC = red blood cells; Hb = haemoglobin; Hc = haematocrit; MCV = mean corpuscular volume; MCH = mean corpuscular haemoglobin; MCHC = mean corpuscular haemoglobin concentration.

**Table 8.** Mixed effect model coefficients for platelet parameters.

Variable	Males		Females	
	Platelets	MPV	Platelets	MPV
*N*	*383016*	*59412*	*517999*	*81765*
*n cases*	*1283*	*8912*	*1175*	*7868*
**Fixed effects:**						
Constant	257.1629 (254.9297, 259.3962)	9.5231 (9.4712, 9.5749)	276.9223 (274.8745, 278.9700)	9.6562 (9.6165, 9.6959)
Age at index date ^1^ (years)	-0.2013 (-0.2445, -0.1581)	-0.0031 (-0.0040, -0.0023)	-0.0199 (-0.0603, 0.0205)	-0.0046 (-0.0052, -0.0040)
Age at index date ^1^ – knot at 60 (years)	-0.5702 (-0.6874, -0.4529)		-0.4970 (-0.6110, -0.3830)	
Age at index date ^1^ – knot at 70 (years)	0.4389 (0.2598, 0.6180)		0.9261 (0.7584, 1.0938)	
Age at index date ^1^ – knot at 80 (years)	0.1632 (-0.0635, 0.3899)		-0.7335 (-0.9056, -0.5615)	
Time to index date ^1^ (years)	2.7155 (2.2004, 3.2306)	-0.0974 (-0.1178, -0.0770)	3.1482 (2.7212, 3.5753)	-0.0902 (-0.1057, -0.0746)
Time to index date ^1^ date – knot at one (years)	-0.7797 (-1.6028, 0.0434)	0.0433 (0.0112, 0.0753)	-1.3100 (-1.9898, -0.6302)	0.0331 (0.0087, 0.0576)
Time to index date ^1^ date – knot at two (years)	0.3853 (-0.1495, 0.9200)	0.0291 (0.0084, 0.0498)	-0.1326 (-0.5733, 0.3080)	0.0453 (0.0295, 0.0612)
Time to index date ^1^ date – knot at four (years)	-2.2486 (-2.5110, -1.9862)	0.0148 (0.0038, 0.0258)	-2.1814 (-2.3969, -1.9658)	-0.0010 (-0.0094, 0.0074)
CRC present	128.6209 (96.3364, 160.9054)	-0.3766 (-0.4700, -0.2831)	110.9094 (74.8031, 147.0156)	-0.4480 (-0.5434, -0.3525)
CRC present by time to index date ^1^ interaction	-57.3824 (-59.7265, -55.0383)	0.1735 (0.0770, 0.2699)	-70.0709 (-72.5177, -67.6241)	0.3215 (0.2301, 0.4130)
CRC present by time to index date ^1^ (knot at one) interaction	50.8421 (46.6410, 55.0433)	-0.1107 (-0.2790, 0.0576)	61.9149 (57.5957, 66.2340)	-0.2983 (-0.4579, -0.1387)
CRC present by time to index date ^1^ (knot at two) interaction	2.9225 (-0.1281, 5.9731)	-0.0245 (-0.1446, 0.0955)	3.9056 (0.8180, 6.9931)	-0.0328 (-0.1470, 0.0813)
CRC present by time to index date ^1^ (knot at four) interaction	3.5438 (2.0653, 5.0224)	-0.0356 (-0.0989, 0.0276)	3.0603 (1.5774, 4.5433)	0.0439 (-0.0152, 0.1031)
CRC presence by age interaction	-0.9456 (-1.5201, -0.3710)		-0.4272 (-1.0804, 0.2260)	
CRC presence by age (knot at 60) interaction	0.7702 (-0.2018, 1.7422)		1.2526 (0.0544, 2.4507)	
CRC presence by age (knot at 70) interaction	0.9991 (0.0572, 1.9410)		-0.8446 (-2.0166, 0.3275)	
CRC presence by age (knot at 80) interaction	-2.1333 (-3.1669, -1.0998)		-0.0662 (-1.0705, 0.9380)	
**Random effects:**						
Intercept for patient (variance)	3.8e+03 (3.7e+03, 3.8e+03)	1.8264 (1.7987, 1.8546)	4.5e+03 (4.5e+03, 4.6e+03)	1.8457 (1.8225, 1.8691)
Slope for time to index date ^1^ (variance)	39.5014 (38.6595, 40.3616)	0.0271 (0.0260, 0.0282)	47.4155 (46.6445, 48.1993)	0.0274 (0.0266, 0.0283)
Covariance between Intercept for patient and slope for time to index date (covariance)	-1.7e+02 (-1.7e+02, -1.6e+02)	-0.1103 (-0.1151, -0.1055)	-1.9e+02 (-2.0e+02, -1.9e+02)	-0.1117 (-0.1155, -0.1079)
Residual (variance)	1.6e+03 (1.6e+03, 1.7e+03)	0.2919 (0.2890, 0.2949)	1.8e+03 (1.8e+03, 1.8e+03)	0.2884 (0.2862, 0.2906)

^1^Index date was the date of diagnosis for cases and a randomly selected date in the patient’s study period for controls. Abbreviations: MPV = mean platelet volume.

**Table 9.** Mixed effect model coefficients for white blood cell count parameters - males.

Variable	WBC	Basophils	Eosinophils	Lymphocytes	Monocytes	Neutrophils
*N*	*356191*	*328523*	*329715*	*338453*	*335510*	*339093*
*n cases*	*8133*	*7869*	*7892*	*8113*	*8055*	*8509*
**Fixed effects:**						
Constant	6.8860 (6.7850, 6.9871)	0.0653 (0.0645, 0.0660)	0.2148 (0.2110, 0.2186)	2.4595 (2.4323, 2.4867)	0.4892 (0.4808, 0.4977)	3.9542 (3.8711, 4.0372)
Age at index date ^1^ (years)	0.0050 (0.0031, 0.0070)	0.0000 (-0.0000, 0.0000)	0.0003 (0.0002, 0.0003)	-0.0063 (-0.0067, -0.0058)	0.0013 (0.0012, 0.0015)	0.0048 (0.0032, 0.0063)
Age at index date ^1^ – knot at 60 (years)	0.0025 (-0.0025, 0.0074)				0.0008 (0.0003, 0.0012)	0.0141 (0.0113, 0.0170)
Age at index date ^1^ – knot at 70 (years)	0.0066 (0.0004, 0.0127)		0.0006 (0.0005, 0.0008)		0.0001 (-0.0003, 0.0006)	
Age at index date ^1^ – knot at 80 (years)				-0.0135 (-0.0165, -0.0105)		0.0010 (-0.0049, 0.0070)
Age at index date ^1^ – knot at 85 (years)	-0.0126 (-0.0254, 0.0002)					
Time to index date ^1^ (years)	0.0046 (-0.0261, 0.0354)	-0.0002 (-0.0002, -0.0001)	-0.0009 (-0.0011, -0.0008)	0.0102 (-0.0019, 0.0223)	-0.0040 (-0.0065, -0.0016)	-0.0436 (-0.0706, -0.0166)
Time to index date ^1^ date – knot at one (years)	-0.0248 (-0.0743, 0.0246)			0.0058 (-0.0134, 0.0249)	-0.0031 (-0.0070, 0.0008)	0.0254 (-0.0186, 0.0694)
Time to index date ^1^ date – knot at two (years)	0.0081 (-0.0244, 0.0405)			-0.0029 (-0.0152, 0.0094)	0.0021 (-0.0005, 0.0046)	0.0091 (-0.0206, 0.0388)
Time to index date ^1^ date – knot at four (years)	0.0105 (-0.0054, 0.0264)			-0.0025 (-0.0088, 0.0037)	0.0004 (-0.0008, 0.0018)	0.0086 (-0.0064, 0.0235)
CRC present	-0.5156 (-1.9304, 0.8992)	0.0018 (0.0006, 0.0031)	-0.0198 (-0.0636, 0.0241)	-0.1594 (-0.2052, -0.1136)	0.1034 (0.0959, 0.1108)	1.4463 (0.3253, 2.5673)
CRC present by time to index date ^1^ interaction	-0.8386 (-0.9812, -0.6959)	-0.0002 (-0.0005, 0.0001)	-0.0021 (-0.0031, -0.0012)	0.1662 (0.1123, 0.2202)	-0.0823 (-0.0935, -0.0710)	-0.9473 (-1.0745, -0.8201)
CRC present by time to index date ^1^ (knot at one) interaction	0.6959 (0.4376, 0.9541)			-0.1616 (-0.2574, -0.0658)	0.0806 (0.0602, 0.1011)	0.8945 (0.6613, 1.1278)
CRC present by time to index date ^1^ (knot at two) interaction	0.1083 (-0.0814, 0.2980)			0.0037 (-0.0658, 0.0731)	-0.0079 (-0.0230, 0.0072)	0.0181 (-0.1570, 0.1933)
CRC present by time to index date ^1^ (knot at four) interaction	0.0194 (-0.0721, 0.1110)			-0.0147 (-0.0497, 0.0202)	0.0100 (0.0026, 0.0174)	0.0487 (-0.0376, 0.1349)
CRC presence by age interaction	0.0332 (0.0081, 0.0583)		0.0006 (-0.0001, 0.0013)			-0.0053 (-0.0249, 0.0144)
CRC presence by age (knot at 60) interaction	-0.0825 (-0.1236, -0.0415)					0.0015 (-0.0232, 0.0263)
CRC presence by age (knot at 70) interaction	0.0546 (0.0221, 0.0872)		-0.0026 (-0.0037, -0.0014)			
CRC presence by age (knot at 80) interaction						-0.0134 (-0.0403, 0.0135)
CRC presence by age (knot at 85) interaction	-0.0695 (-0.1293, -0.0097)					
**Random effects:**						
Intercept for patient (variance)	5.3129 (5.2597, 5.3667)	0.0016 (0.0016, 0.0017)	0.0219 (0.0217, 0.0221)	2.3665 (2.3502, 2.3830)	0.0348 (0.0344, 0.0351)	2.1016 (2.0698, 2.1338)
Slope for time to index date ^1^ (variance)	0.0876 (0.0853, 0.0899)	0.0000 (0.0000, 0.0000)	0.0003 (0.0003, 0.0003)	0.0583 (0.0574, 0.0592)	0.0005 (0.0005, 0.0006)	0.0410 (0.0389, 0.0432)
Covariance between Intercept for patient and slope for time to index date (covariance)	-0.3649 (-0.3756, -0.3542)	-0.0001 (-0.0001, -0.0001)	-0.0011 (-0.0012, -0.0011)	-0.2447 (-0.2483, -0.2410)	-0.0014 (-0.0014, -0.0013)	0.1588 (0.1528, 0.1648)
Residual (variance)	5.8167 (5.7964, 5.8372)	0.0017 (0.0017, 0.0017)	0.0141 (0.0140, 0.0141)	0.6405 (0.6380, 0.6430)	0.0334 (0.0333, 0.0336)	4.7837 (4.7650, 4.8024)

^1^Index date was the date of diagnosis for cases and a randomly selected date in the patient’s study period for controls. Abbreviations: WBC = white blood cell count.

**Table 10.** Mixed effect model coefficients for white blood cell count parameters - females.

Variable	WBC	Basophils	Eosinophils	Lymphocytes	Monocytes	Neutrophils
*N*	*485552*	*444145*	*445731*	*461204*	*456981*	*461893*
*n cases*	*7249*	*6941*	*6966*	*7224*	*7155*	*7574*
Fixed effects:						
Constant	8.1097 (8.0197, 8.1997)	0.0614 (0.0608, 0.0619)	0.1950 (0.1922, 0.1978)	2.0705 (2.0519, 2.0891)	0.5118 (0.5057, 0.5179)	5.7413 (5.6847, 5.7978)
Age at index date ^1^ (years)	-0.0218 (-0.0235, -0.0200)	0.0001 (0.0001, 0.0001)	0.0001 (0.0000, 0.0001)	-0.0001 (-0.0004, 0.0002)	-0.0005 (-0.0006, -0.0004)	-0.0311 (-0.0322, -0.0300)
Age at index date ^1^ – knot at 60 (years)	0.0461 (0.0416, 0.0507)				0.0037 (0.0033, 0.0040)	0.0652 (0.0633, 0.0671)
Age at index date ^1^ – knot at 70 (years)	0.0041 (-0.0013, 0.0095)		0.0001 (0.0000, 0.0002)		0.0017 (0.0012, 0.0022)	
Age at index date ^1^ – knot at 80 (years)				-0.0337 (-0.0357, -0.0316)	-0.0029 (-0.0034, -0.0024)	-0.0131 (-0.0161, -0.0100)
Age at index date ^1^ – knot at 85 (years)	-0.0395 (-0.0473, -0.0317)					
Age at index date ^1^ – knot at 90 (years)				0.0420 (0.0353, 0.0486)		
Time to index date ^1^ (years)	0.0004 (-0.0289, 0.0298)	-0.0003 (-0.0003, -0.0003)	-0.0004 (-0.0005, -0.0002)	0.0192 (0.0098, 0.0286)	-0.0042 (-0.0058, -0.0025)	-0.0226 (-0.0417, -0.0035)
Time to index date ^1^ date – knot at one (years)	-0.0040 (-0.0512, 0.0433)			-0.0118 (-0.0268, 0.0032)	0.0004 (-0.0023, 0.0031)	0.0269 (-0.0039, 0.0577)
Time to index date ^1^ date – knot at two (years)	-0.0011 (-0.0320, 0.0299)			0.0020 (-0.0077, 0.0117)	-0.0016 (-0.0034, 0.0001)	-0.0066 (-0.0270, 0.0137)
Time to index date ^1^ date – knot at four (years)	0.0114 (-0.0036, 0.0265)			-0.0039 (-0.0086, 0.0008)	0.0022 (0.0013, 0.0030)	0.0130 (0.0029, 0.0231)
CRC present	0.4778 (-1.0104, 1.9660)	0.0016 (0.0003, 0.0029)	0.0020 (-0.0378, 0.0418)	-0.1701 (-0.2112, -0.1290)	0.1282 (0.1214, 0.1350)	1.5446 (1.4734, 1.6159)
CRC present by time to index date ^1^ interaction	-1.4093 (-1.5821, -1.2365)	-0.0003 (-0.0006, -0.0000)	-0.0019 (-0.0028, -0.0011)	0.1314 (0.0773, 0.1855)	-0.1098 (-0.1195, -0.1002)	-1.4270 (-1.5393, -1.3147)
CRC present by time to index date ^1^ (knot at one) interaction	1.4600 (1.1503, 1.7696)			-0.1227 (-0.2185, -0.0269)	0.1090 (0.0918, 0.1263)	1.4181 (1.2162, 1.6200)
CRC present by time to index date ^1^ (knot at two) interaction	-0.0444 (-0.2687, 0.1799)			0.0302 (-0.0384, 0.0989)	-0.0007 (-0.0132, 0.0118)	0.0331 (-0.1145, 0.1808)
CRC present by time to index date ^1^ (knot at four) interaction	-0.0650 (-0.1713, 0.0413)			-0.0378 (-0.0706, -0.0049)	-0.0036 (-0.0096, 0.0025)	-0.1051 (-0.1766, -0.0335)
CRC presence by age interaction	0.0190 (-0.0079, 0.0459)		0.0002 (-0.0004, 0.0008)			
CRC presence by age (knot at 60) interaction	-0.0325 (-0.0800, 0.0150)					
CRC presence by age (knot at 70) interaction	0.0125 (-0.0251, 0.0501)		-0.0011 (-0.0021, -0.0001)			
CRC presence by age (knot at 85) interaction	0.0202 (-0.0244, 0.0647)					
Random effects:						
Intercept for patient (variance)	4.9756 (4.9232, 5.0286)	0.0016 (0.0016, 0.0016)	0.0169 (0.0167, 0.0170)	1.3624 (1.3534, 1.3716)	0.0295 (0.0292, 0.0297)	1.9321 (1.9110, 1.9535)
Slope for time to index date ^1^ (variance)	0.1120 (0.1090, 0.1151)	0.0000 (0.0000, 0.0000)	0.0002 (0.0002, 0.0002)	0.0147 (0.0144, 0.0151)	0.0004 (0.0004, 0.0004)	0.0471 (0.0459, 0.0484)
Covariance between Intercept for patient and slope for time to index date (covariance)	-0.4378 (-0.4496, -0.4261)	-0.0001 (-0.0001, -0.0001)	-0.0009 (-0.0009, -0.0009)	-0.1000 (-0.1017, -0.0983)	-0.0018 (-0.0019, -0.0018)	-0.1056 (-0.1102, -0.1010)
Residual (variance)	9.0166 (8.9919, 9.0413)	0.0016 (0.0016, 0.0016)	0.0116 (0.0116, 0.0116)	0.7342 (0.7320, 0.7364)	0.0243 (0.0242, 0.0244)	3.6243 (3.6139, 3.6347)

^1^Index date was the date of diagnosis for cases and a randomly selected date in the patient’s study period for controls. Abbreviations: WBC = white blood cell count.

**Figure 4.**
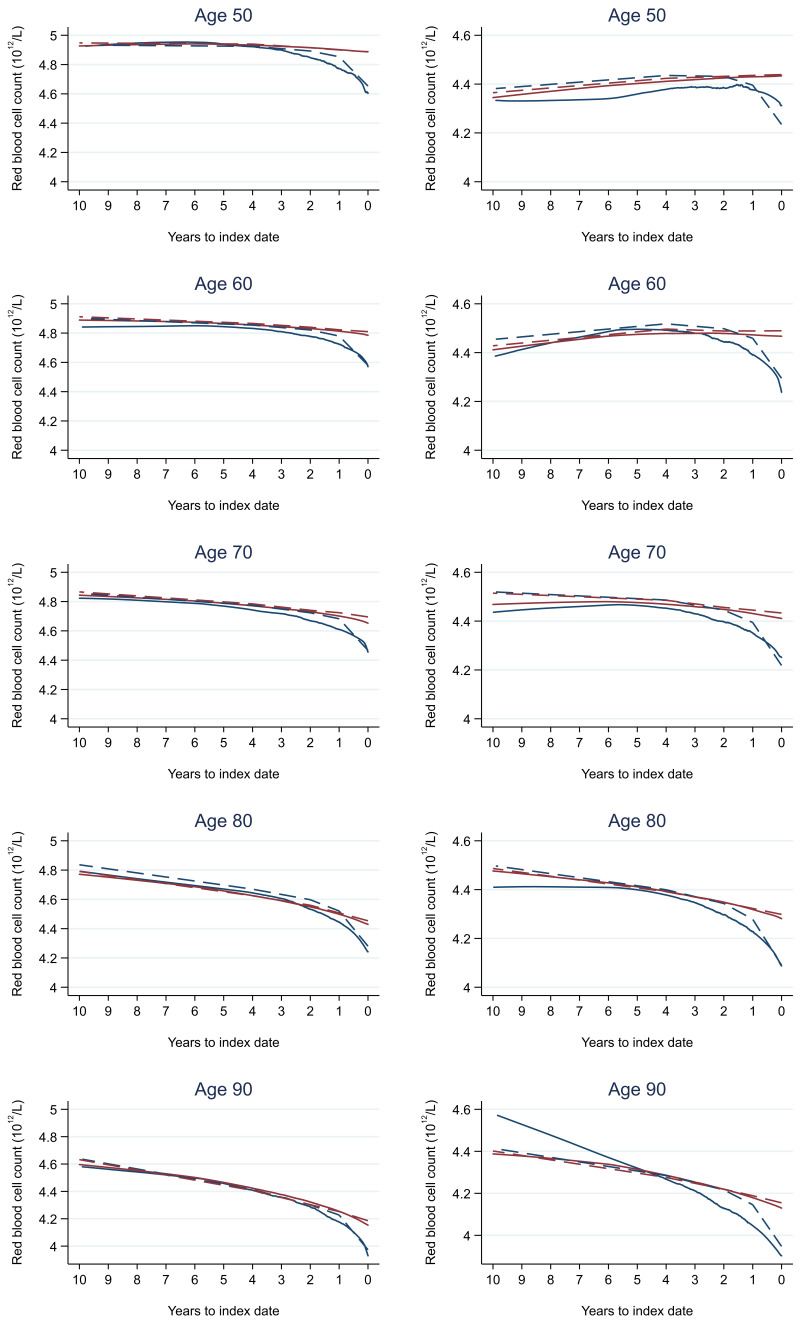
Red blood cell count trends between cases and controls, by age at index date ^1,2^. ^1^Index date was the date of diagnosis for cases and a randomly selected date in the patient’s study period for controls. ^2^LOWESS trends are age (+/- 3 years) and modelled (fixed effects) trends are taken at that specific age. Legend: males (left) and females (right). Colorectal cancer (blue line) and no cancer (red line). LOWESS trend (solid line) and modelled trend (dashed line).

Trends in cases and controls for all FBC parameters in Figure 4– Figure 17. In the raw data (LOWESS curves), there was no apparent difference in trends measured from 10 to four years before index date between cases and controls for both males and females. Within four years before index date, levels changed steadily over time in patients without a diagnosis, such as the reduction in haemoglobin over time that may be due to increasing age. However, cases had trends in FBC levels that diverged from controls for all parameters, with the interaction term between presence of colorectal cancer and time to index date (including splines terms) reaching statistical significance in the mixed effects models (p <0.05 for each FBC parameter model), except basophil count in males (coefficient = -0.0002 (95% CI = -0.0005, 0.0001)). Additionally for cases, our Figures indicate that the rate of change in many FBC levels increased as the time to diagnosis approached. Individualised trends (raw and predicted) are given for four cases ( Figure 18) and four controls ( Figure 19).

**Figure 5.**
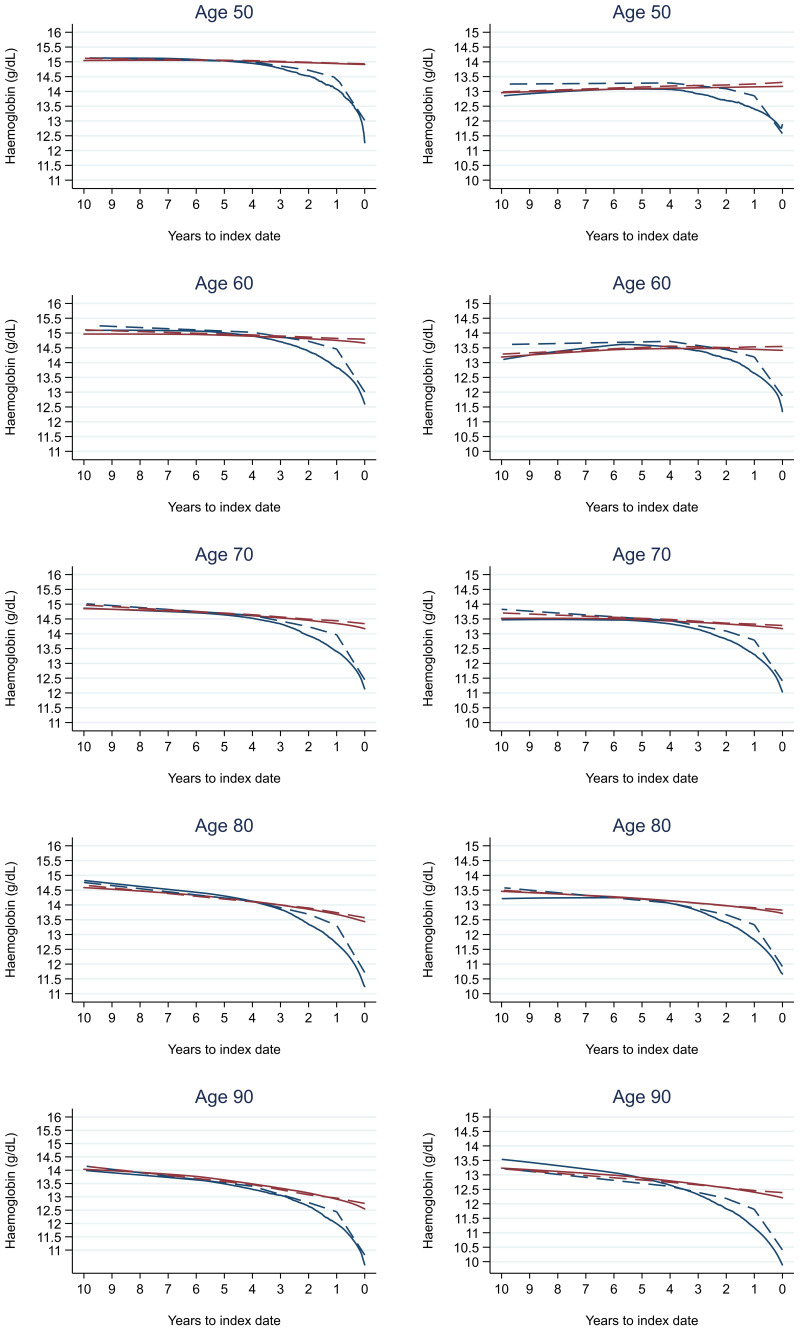
Haemoglobin trends between cases and controls, by age at index date ^1,2^. ^1^Index date was the date of diagnosis for cases and a randomly selected date in the patient’s study period for controls. ^2^LOWESS trends are age (+/- 3 years) and modelled (fixed effects) trends are taken at that specific age. Legend: males (left) and females (right). Colorectal cancer (blue line) and no cancer (red line). LOWESS trend (solid line) and modelled trend (dashed line).

**Figure 6.**
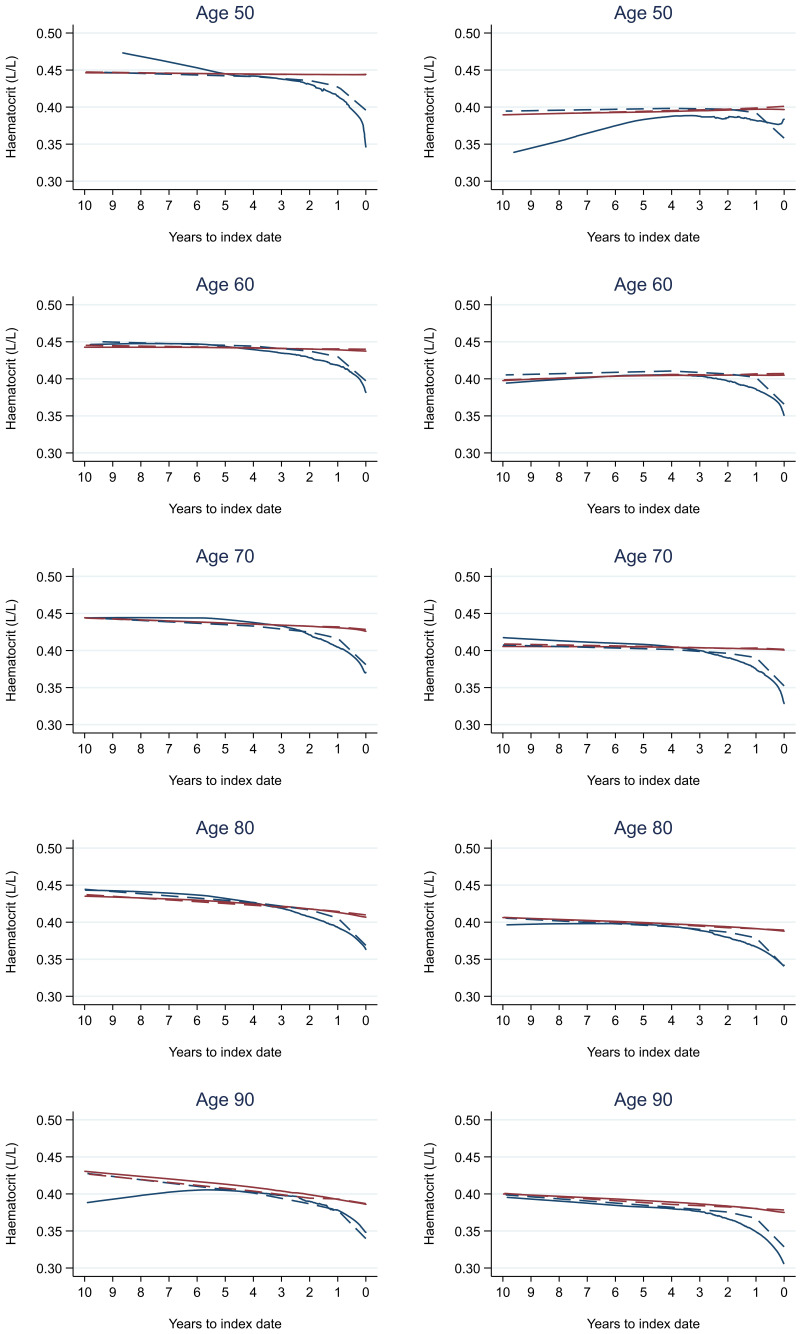
Haematocrit trends between cases and controls, by age at index date ^1,2^. ^1^Index date was the date of diagnosis for cases and a randomly selected date in the patient’s study period for controls. ^2^LOWESS trends are age (+/- 3 years) and modelled (fixed effects) trends are taken at that specific age. Legend: males (left) and females (right). Colorectal cancer (blue line) and no cancer (red line). LOWESS trend (solid line) and modelled trend (dashed line).

**Figure 7.**
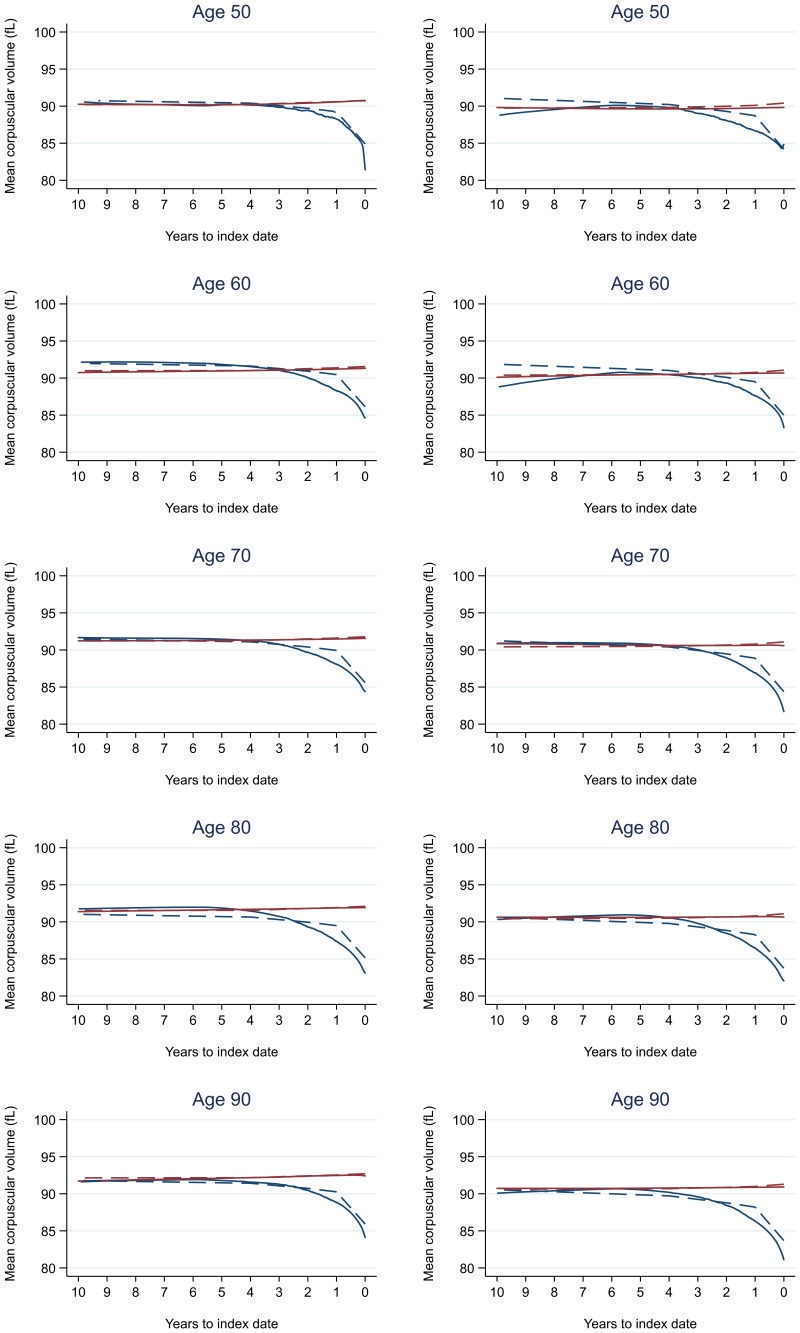
Mean corpuscular volume trends between cases and controls, by age at index date ^1,2^. ^1^Index date was the date of diagnosis for cases and a randomly selected date in the patient’s study period for controls. ^2^LOWESS trends are age (+/- 3 years) and modelled (fixed effects) trends are taken at that specific age. Legend: males (left) and females (right). Colorectal cancer (blue line) and no cancer (red line). LOWESS trend (solid line) and modelled trend (dashed line).

**Figure 8.**
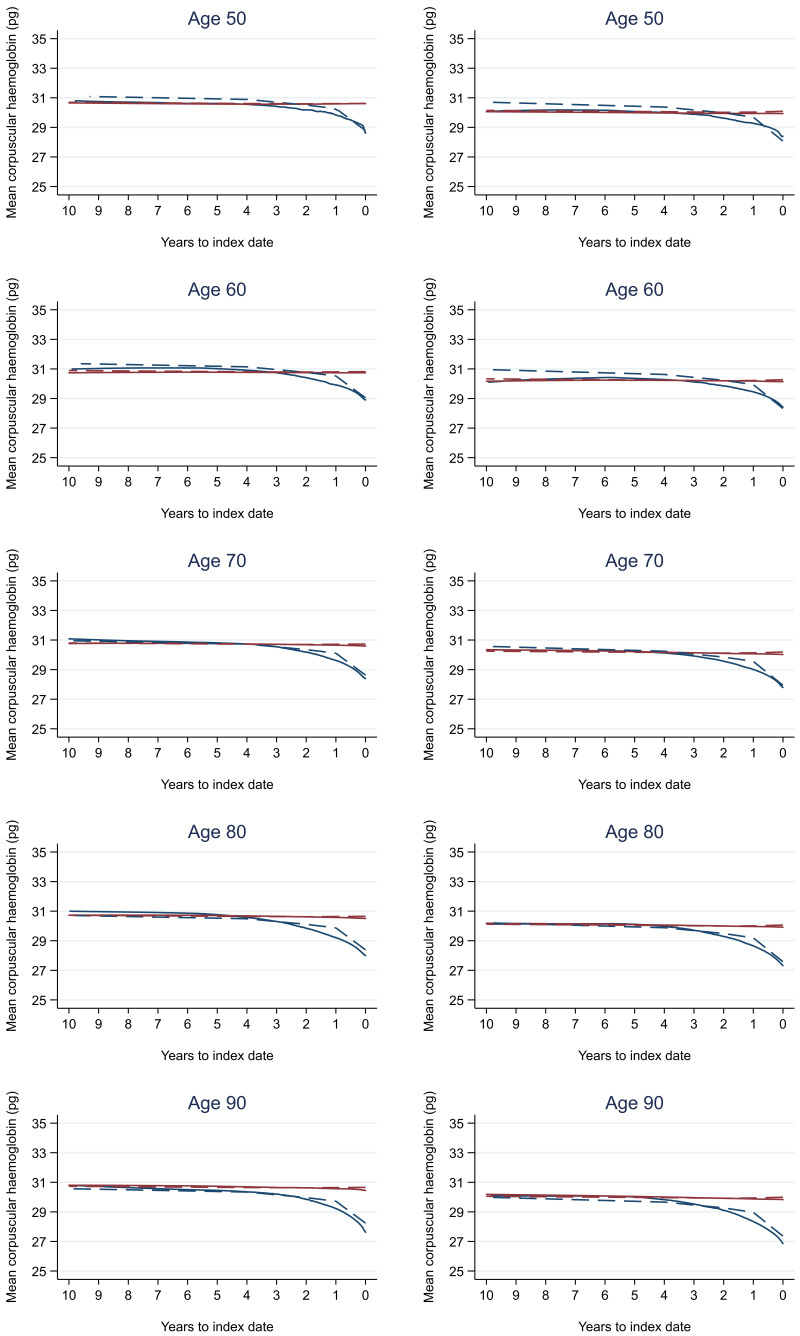
Mean corpuscular haemoglobin trends between cases and controls, by age at index date ^1,2^. ^1^Index date was the date of diagnosis for cases and a randomly selected date in the patient’s study period for controls. ^2^LOWESS trends are age (+/- 3 years) and modelled (fixed effects) trends are taken at that specific age. Legend: males (left) and females (right). Colorectal cancer (blue line) and no cancer (red line). LOWESS trend (solid line) and modelled trend (dashed line).

**Figure 9.**
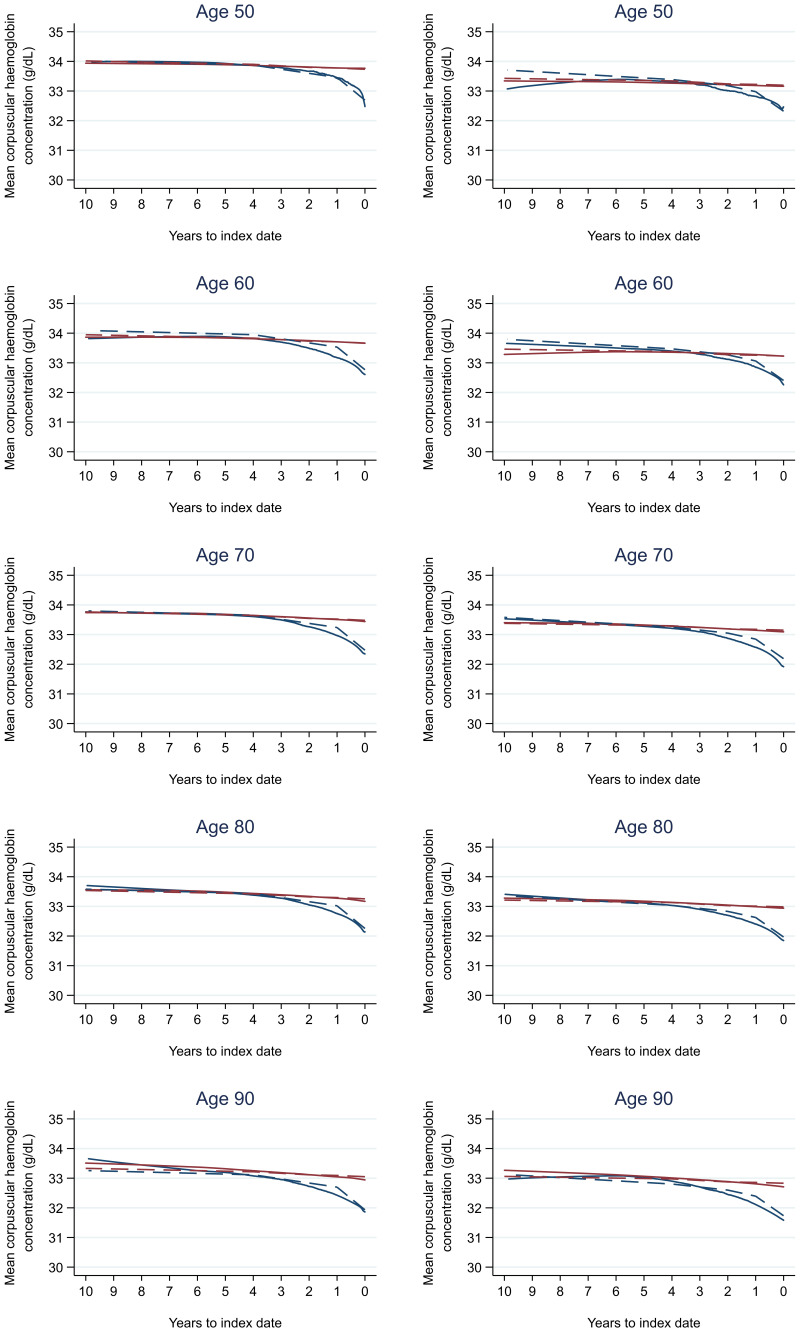
Mean corpuscular haemoglobin concentration trends between cases and controls, by age at index date ^1,2^. ^1^Index date was the date of diagnosis for cases and a randomly selected date in the patient’s study period for controls. ^2^LOWESS trends are age (+/- 3 years) and modelled (fixed effects) trends are taken at that specific age. Legend: males (left) and females (right). Colorectal cancer (blue line) and no cancer (red line). LOWESS trend (solid line) and modelled trend (dashed line).

**Figure 10.**
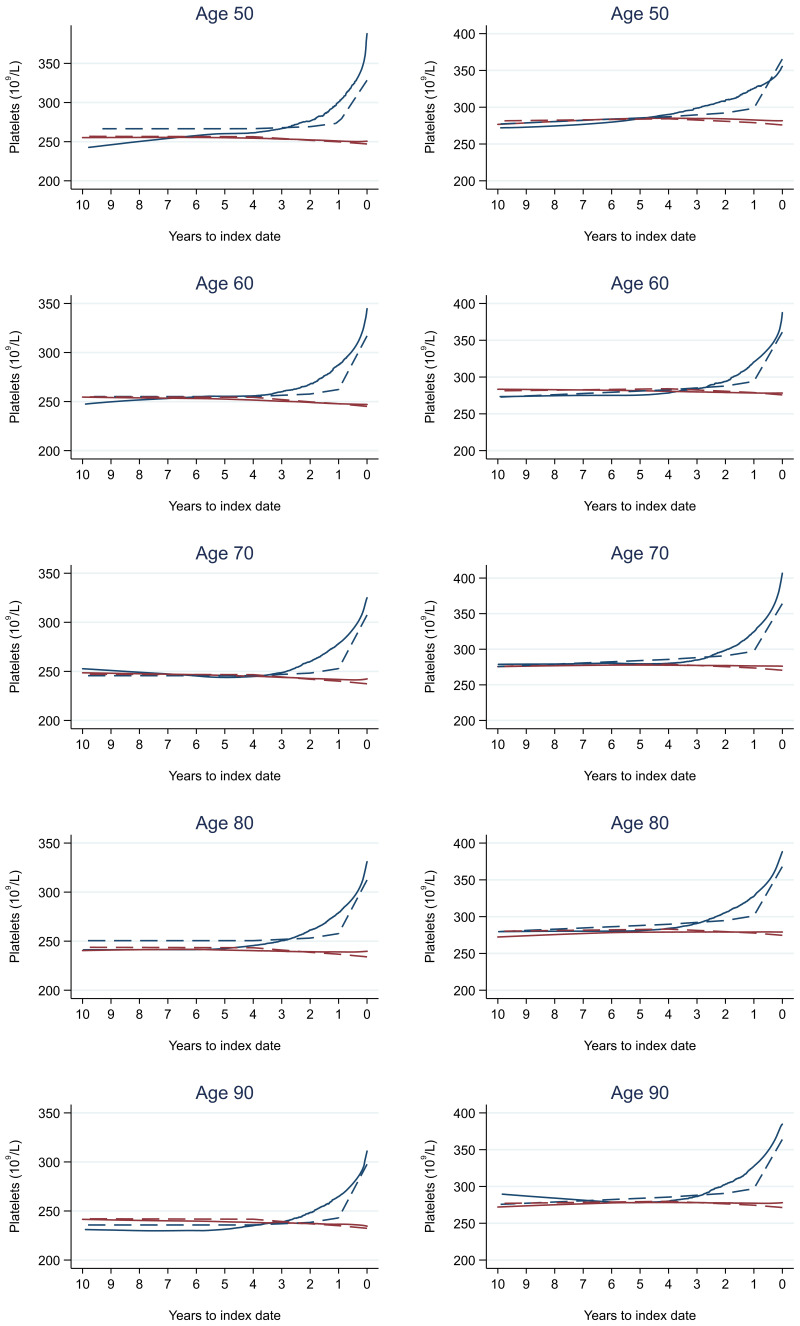
Platelets trends between cases and controls, by age at index date ^1,2^. ^1^Index date was the date of diagnosis for cases and a randomly selected date in the patient’s study period for controls. ^2^LOWESS trends are age (+/- 3 years) and modelled (fixed effects) trends are taken at that specific age. Legend: males (left) and females (right). Colorectal cancer (blue line) and no cancer (red line). LOWESS trend (solid line) and modelled trend (dashed line).

**Figure 11.**
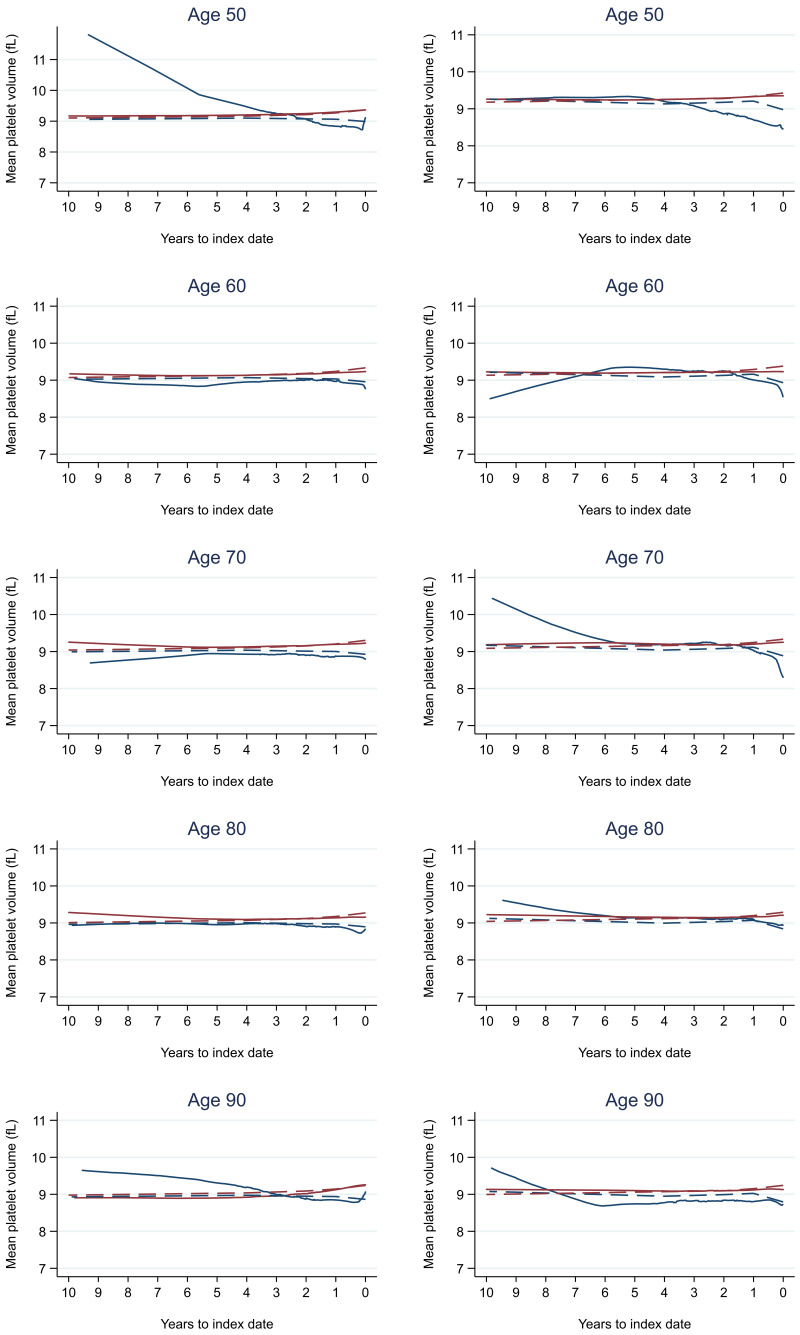
Mean platelet volume trends between cases and controls, by age at index date ^1,2^. ^1^Index date was the date of diagnosis for cases and a randomly selected date in the patient’s study period for controls. ^2^LOWESS trends are age (+/- 3 years) and modelled (fixed effects) trends are taken at that specific age. Legend: males (left) and females (right). Colorectal cancer (blue line) and no cancer (red line). LOWESS trend (solid line) and modelled trend (dashed line).

**Figure 12.**
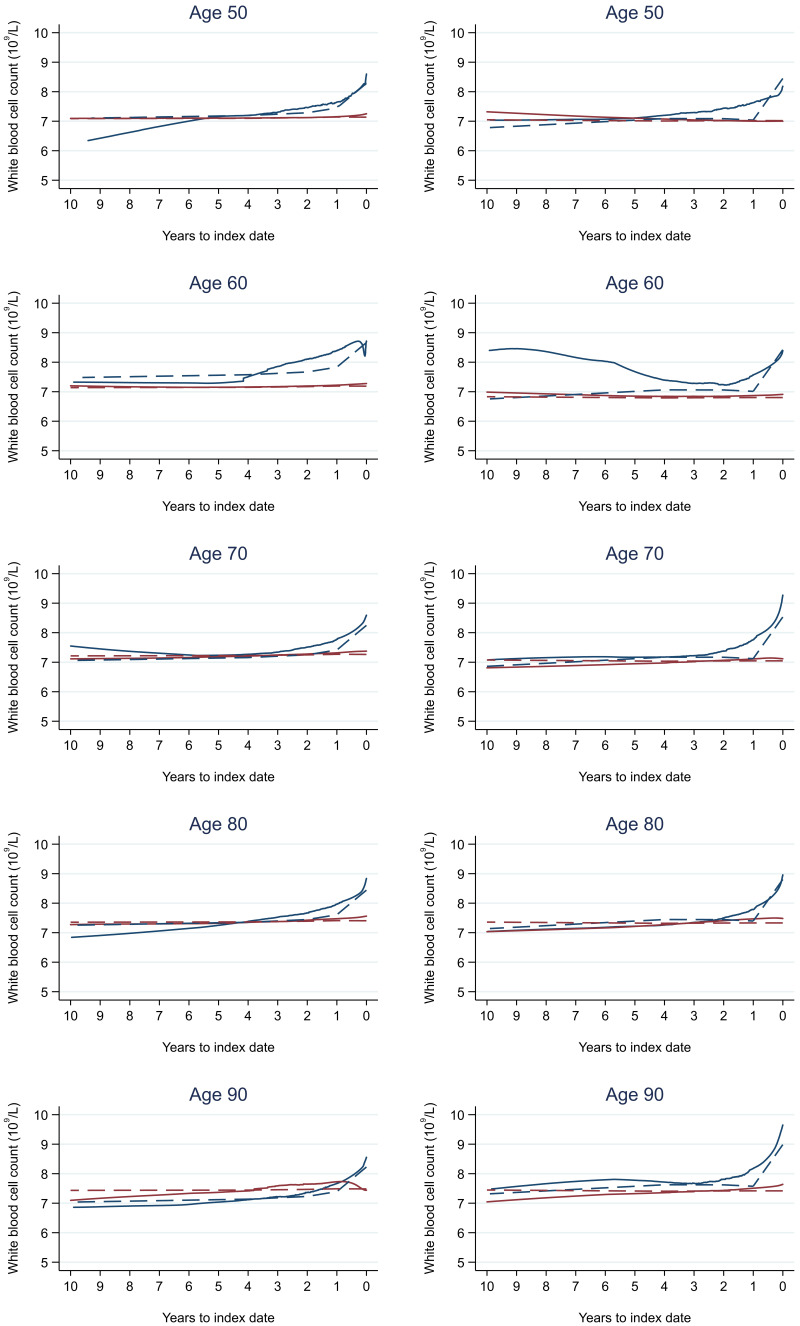
White blood cell count trends between cases and controls, by age at index date ^1,2^. ^1^Index date was the date of diagnosis for cases and a randomly selected date in the patient’s study period for controls. ^2^LOWESS trends are age (+/- 3 years) and modelled (fixed effects) trends are taken at that specific age. Legend: males (left) and females (right). Colorectal cancer (blue line) and no cancer (red line). LOWESS trend (solid line) and modelled trend (dashed line).

**Figure 13.**
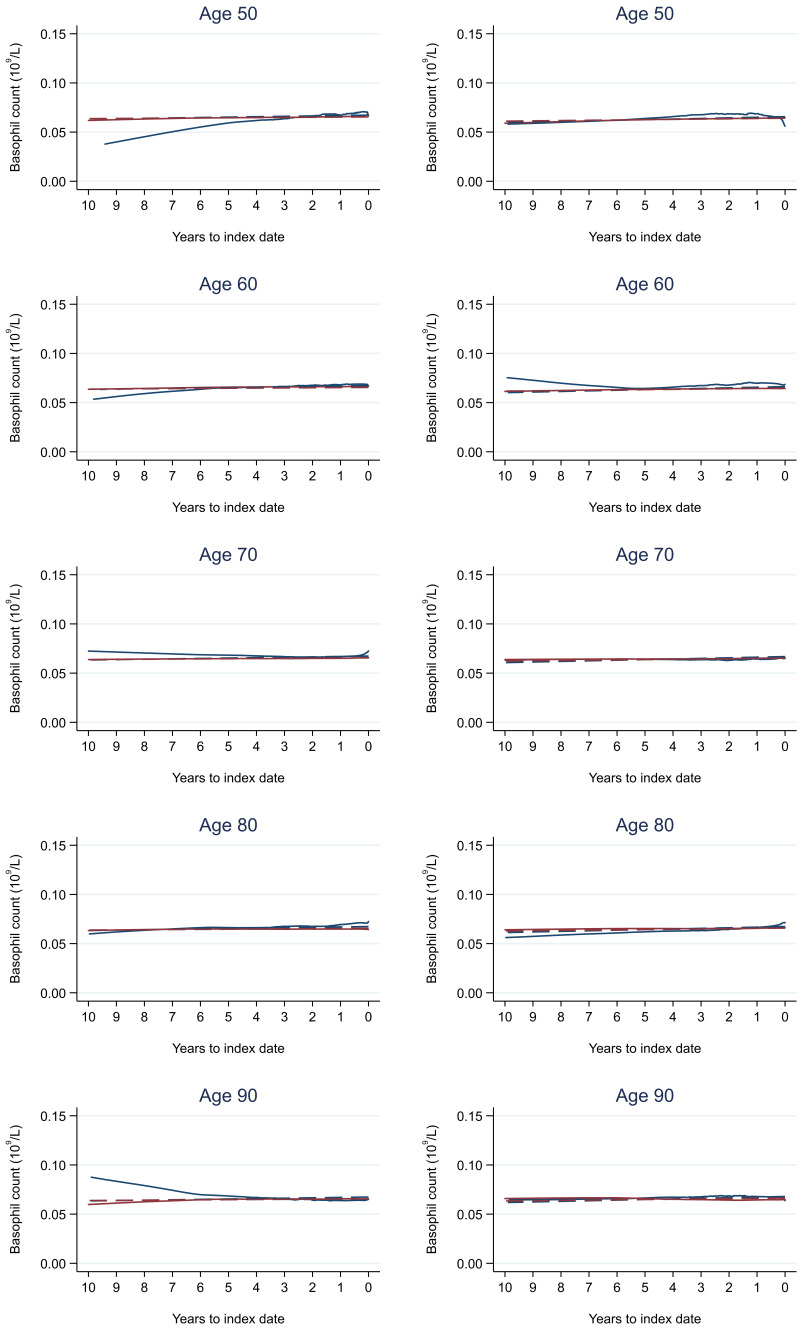
Basophil count trends between cases and controls, by age at index date ^1,2^. ^1^Index date was the date of diagnosis for cases and a randomly selected date in the patient’s study period for controls. ^2^LOWESS trends are age (+/- 3 years) and modelled (fixed effects) trends are taken at that specific age. Legend: males (left) and females (right). Colorectal cancer (blue line) and no cancer (red line). LOWESS trend (solid line) and modelled trend (dashed line).

**Figure 14.**
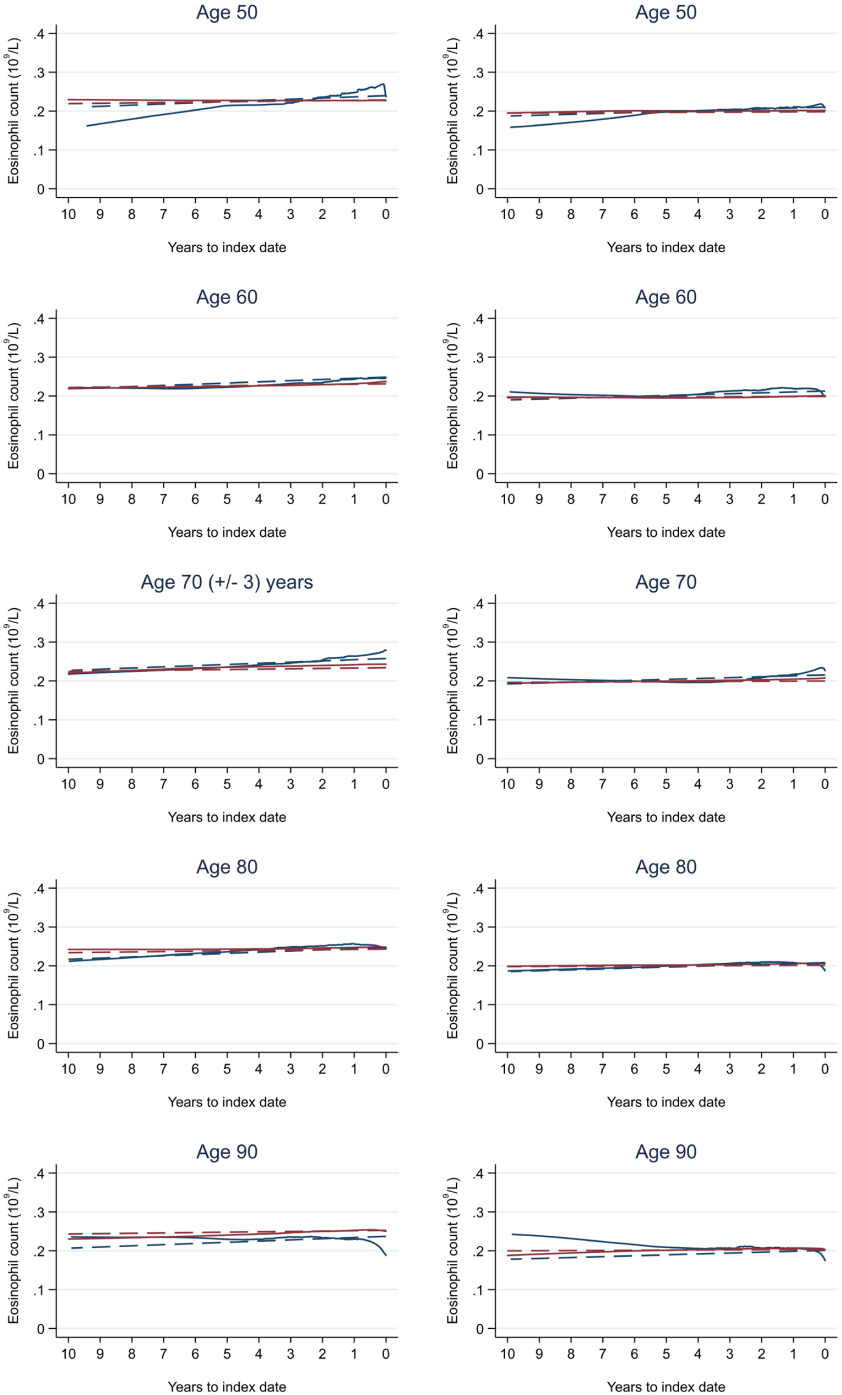
Eosinophil count trends between cases and controls, by age at index date ^1,2^. ^1^Index date was the date of diagnosis for cases and a randomly selected date in the patient’s study period for controls. ^2^LOWESS trends are age (+/- 3 years) and modelled (fixed effects) trends are taken at that specific age. Legend: males (left) and females (right). Colorectal cancer (blue line) and no cancer (red line). LOWESS trend (solid line) and modelled trend (dashed line).

**Figure 15.**
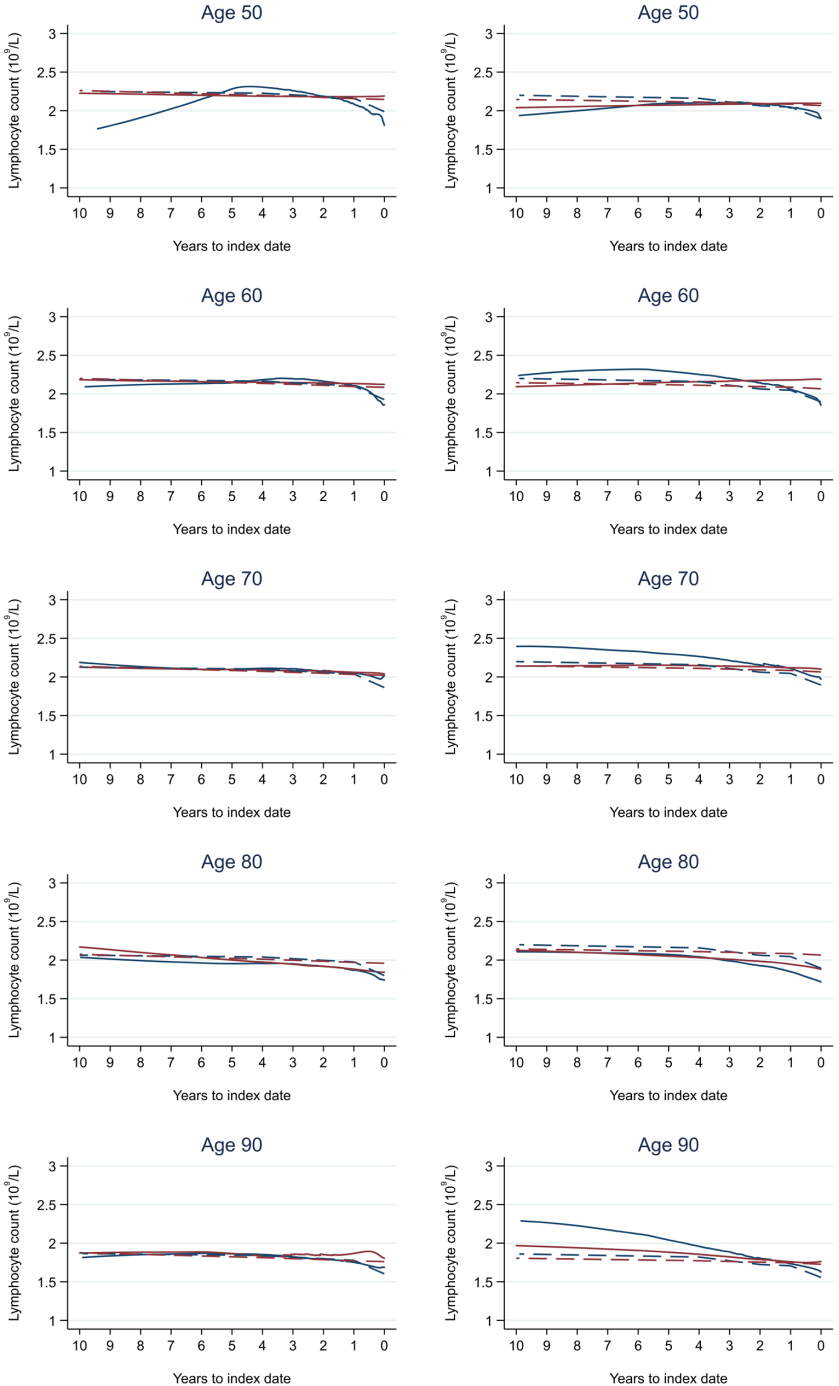
Lymphocyte trends between cases and controls, by age at index date ^1,2^. ^1^Index date was the date of diagnosis for cases and a randomly selected date in the patient’s study period for controls. ^2^LOWESS trends are age (+/- 3 years) and modelled (fixed effects) trends are taken at that specific age. Legend: males (left) and females (right). Colorectal cancer (blue line) and no cancer (red line). LOWESS trend (solid line) and modelled trend (dashed line).

**Figure 16.**
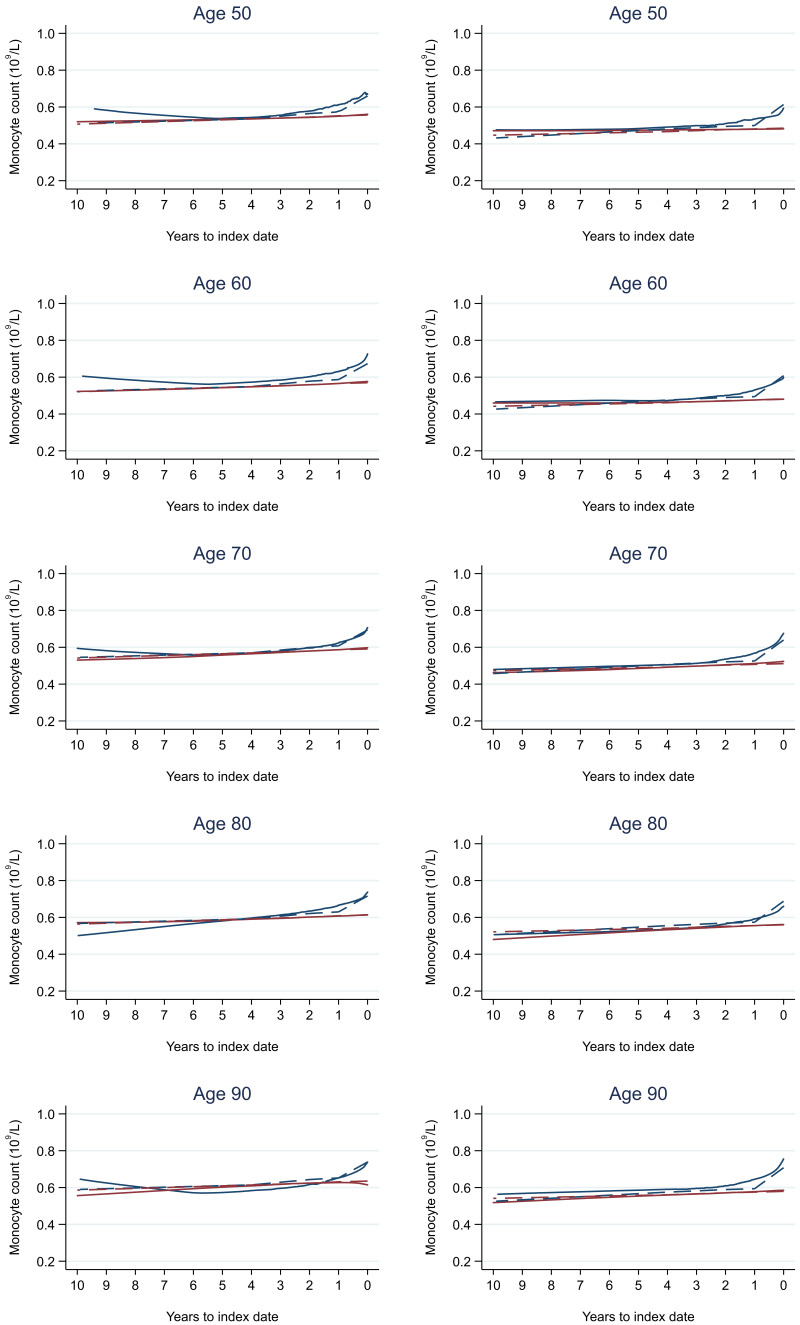
Monocyte count trends between cases and controls, by age at index date ^1,2^. ^1^Index date was the date of diagnosis for cases and a randomly selected date in the patient’s study period for controls. ^2^LOWESS trends are age (+/- 3 years) and modelled (fixed effects) trends are taken at that specific age. Legend: males (left) and females (right). Colorectal cancer (blue line) and no cancer (red line). LOWESS trend (solid line) and modelled trend (dashed line).

**Figure 17.**
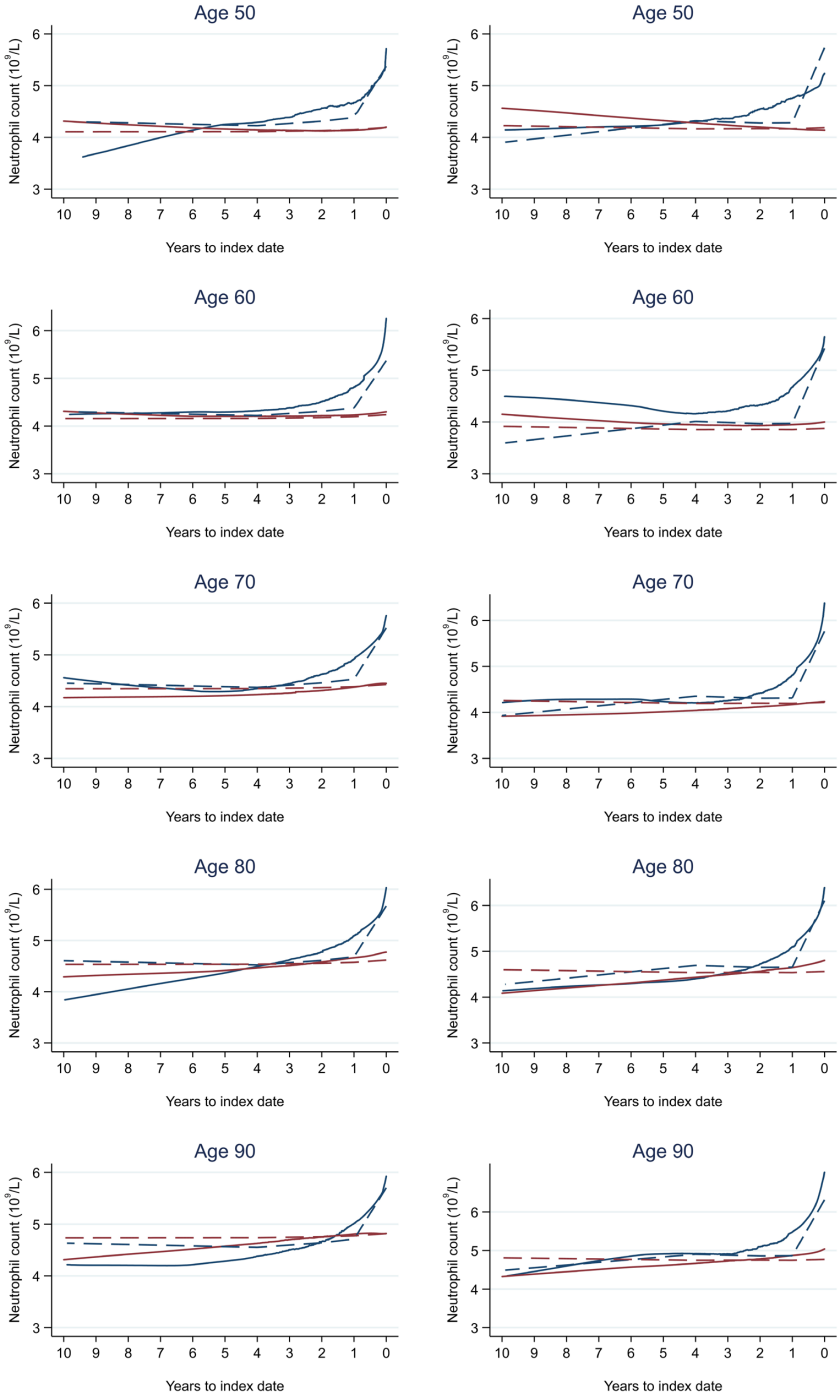
Neutrophil count trends between cases and controls, by age at index date ^1,2^. ^1^Index date was the date of diagnosis for cases and a randomly selected date in the patient’s study period for controls. ^2^LOWESS trends are age (+/- 3 years) and modelled (fixed effects) trends are taken at that specific age. Legend: males (left) and females (right). Colorectal cancer (blue line) and no cancer (red line). LOWESS trend (solid line) and modelled trend (dashed line).

**Figure 18.**
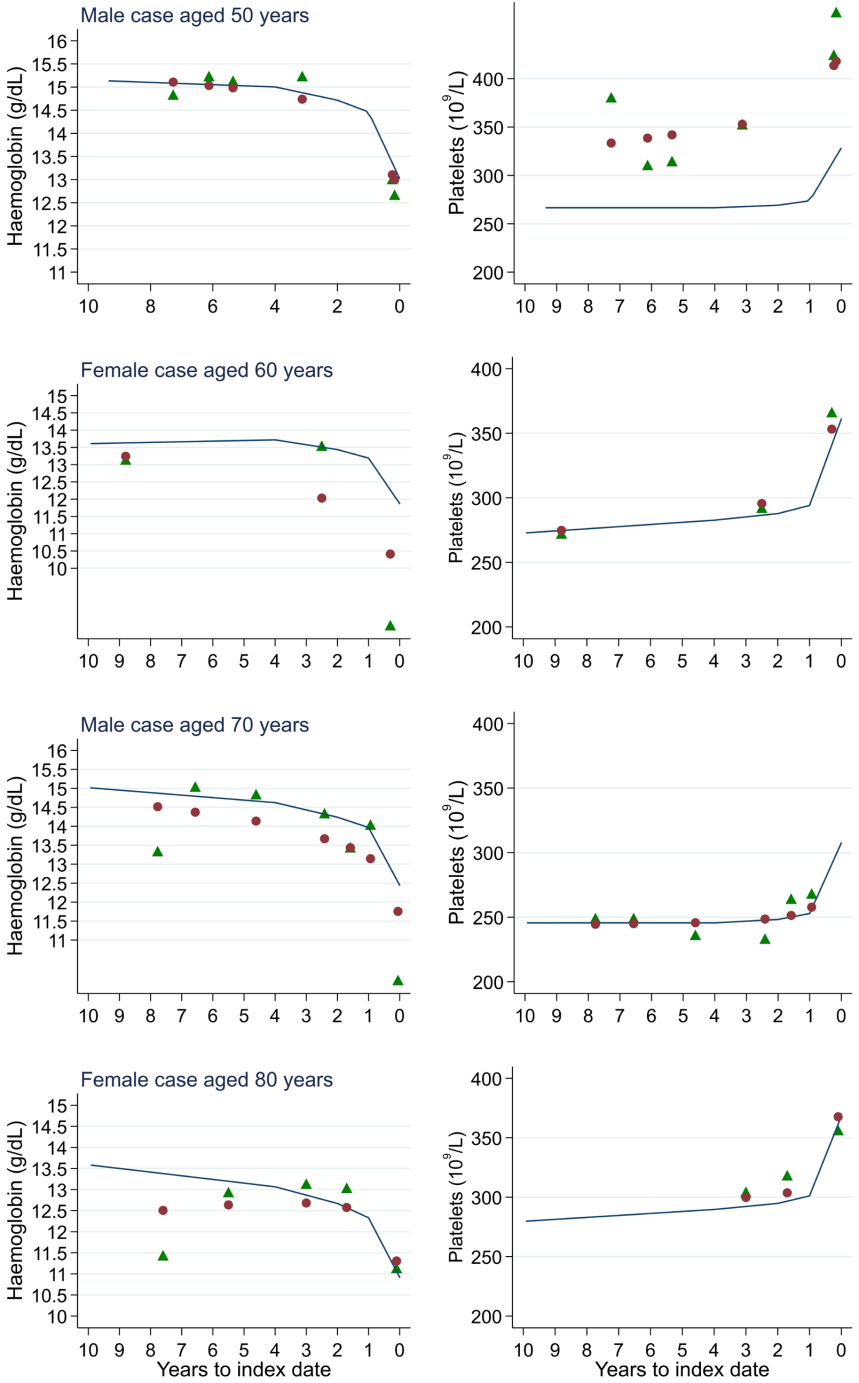
Individualised haemoglobin (left) and platelet (right) trends for four cases by age at index ^1^. ^1^Index date was the date of diagnosis for cases and a randomly selected date in the patient’s study period for controls. Legend: raw data (green triangle), modelled population-level trend (fixed effects) (blue line), modelled individualised patient-level trend (fixed and random effects) (red circle).

**Figure 19.**
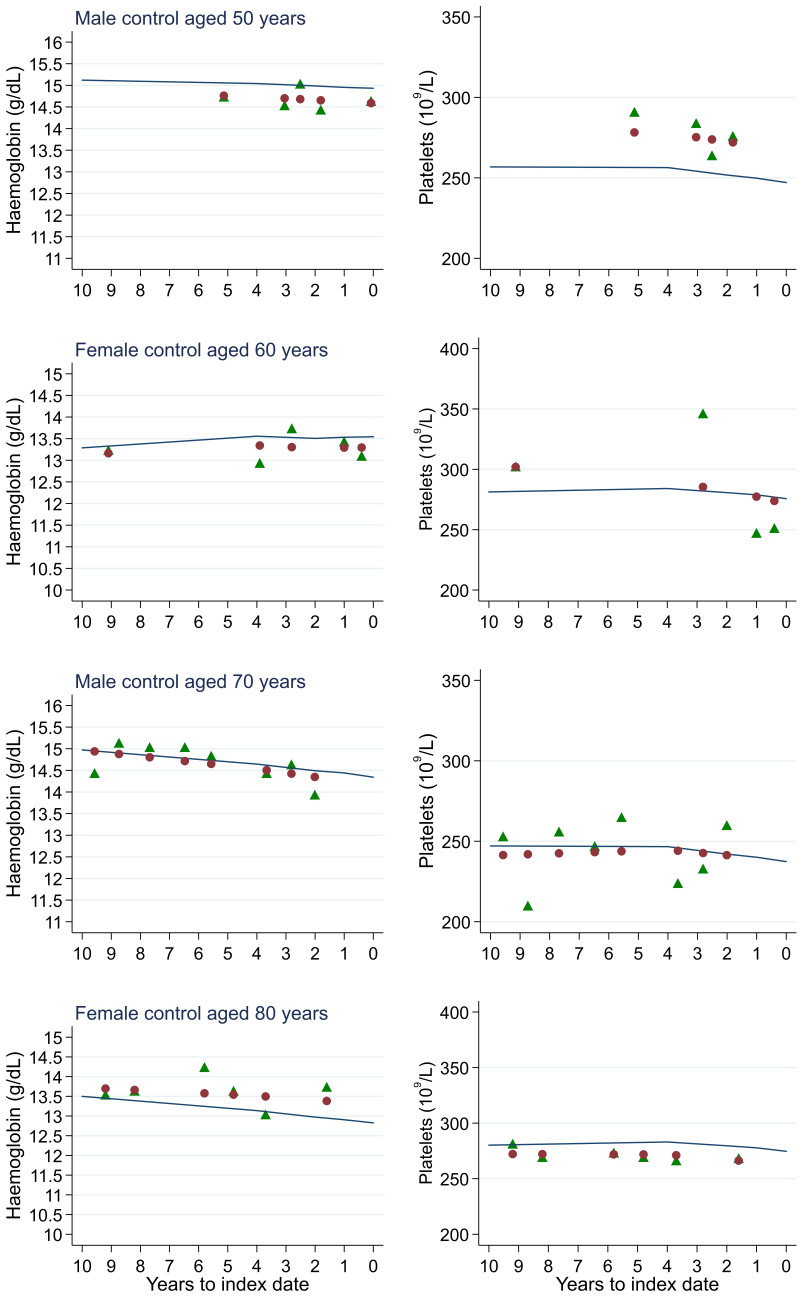
Individualised haemoglobin (left) and platelet (right) trends for four controls by age at index ^1^. ^1^Index date was the date of diagnosis for cases and a randomly selected date in the patient’s study period for controls. Legend: raw data (green triangle), modelled population-level trend (fixed effects) (blue line), modelled individualised patient-level trend (fixed and random effects) (red circle).

### Colorectal cancer and microcytic anaemia

**Figure 20.**
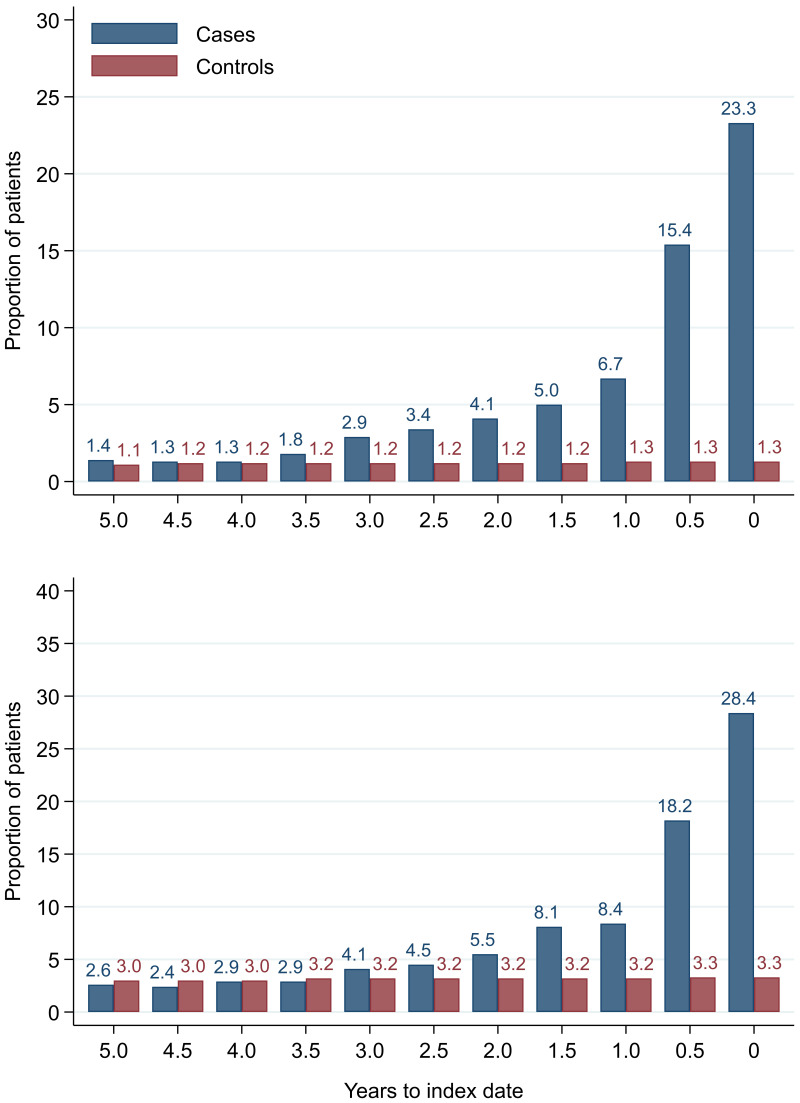
Microcytic anaemia prevalence over six-monthly (+/- 3 months) time band. Legend: males (top) and females (bottom).

Anaemia was present in 48.8% of male cases and 54.3% of female cases on any FBC within one year of diagnosis. At each six-monthly interval up to five years before index date, the proportion with microcytic anaemia was higher in cases than controls ( Figure 20). In cases, the proportion increased as the time to diagnosis approached and was highest at 0–3 months prior to diagnosis: 23.3% (n = 1,188) of 5,107 males and 28.4% (n = 1,286) of 4,521 females with FBCs in that period. The odds of diagnosis (corresponding to microcytic anaemia presence) increased as time to index date approached ( Table 11). Presence of microcytic anaemia statistically significantly increased odds of diagnosis at each time band within three years before index date for both males (three-year OR = 2.2 (95% CI = 1.5, 3.1)) and females (three-year OR = 1.7 (95% CI = 1.3, 2.3)). No odds ratio achieved statistical significance at earlier time points.

**Table 11.** Adjusted odds ratios for diagnosis between patients with and without microcytic anaemia at six-monthly (+/- three months) time intervals prior to index date ^1^.

Time interval (years)	Cases		Controls		Odds ratio (95% CI) ^2^
	Microcytic anaemia	Total	Microcytic anaemia	Total	
Males:					
0	1188	5107	937	69648	19.6 (95% CI=17.7, 21.7)
0.5	529	3424	1456	115123	10.4 (95% CI=9.1, 11.8)
1	171	2560	1301	103281	4.2 (95% CI=3.4, 5.1)
1.5	115	2280	1058	91084	3.5 (95% CI=2.7, 4.4)
2	83	2025	930	79130	2.4 (95% CI=1.8, 3.3)
2.5	63	1837	864	70678	2.3 (95% CI=1.7, 3.1)
3	49	1662	757	61840	2.2 (95% CI=1.5, 3.1)
3.5	29	1593	648	54331	0.9 (95% CI=0.6, 1.5)
4	18	1380	549	47470	0.9 (95% CI=0.5, 1.6)
4.5	15	1186	505	41655	1.0 (95% CI=0.6, 1.7)
5	15	1063	419	36578	1.0 (95% CI=0.5, 1.8)
Females:					
0	1286	4521	3473	105359	12.2 (95% CI=11.3, 13.3)
0.5	588	3237	5684	171034	6.6 (95% CI=5.9, 7.4)
1	208	2483	4948	152754	2.9 (95% CI=2.4, 3.4)
1.5	178	2194	4360	136087	3.0 (95% CI=2.5, 3.6)
2	108	1976	3822	119565	2.0 (95% CI=1.6, 2.6)
2.5	82	1814	3376	106114	1.5 (95% CI=1.1, 2.0)
3	66	1600	2963	93266	1.7 (95% CI=1.3, 2.3)
3.5	44	1492	2597	82236	1.1 (95% CI=0.7, 1.5)
4	40	1359	2233	73537	1.3 (95% CI=0.9, 1.9)
4.5	31	1266	1920	64388	0.8 (95% CI=0.5, 1.3)
5	29	1097	1722	56759	0.9 (95% CI=0.5, 1.4)

^1^Index date was the date of diagnosis for cases and a randomly selected date in the patient’s study period for controls ^2^Adjusted for age at diagnosis (years)

We compared our graphical trends to microcytic anaemia thresholds. For haemoglobin, trends suggest that the threshold is on average only reached in cases very close to the time of diagnosis, except in the oldest age groups, where the threshold is reached slightly earlier but even controls in this age group reach the threshold. For mean corpuscular volume, trends suggest the threshold is on average not reached, regardless of age group. This suggests only a minority of patients have iron-deficiency determined from the FBC test (maximum 23.3% males and 28.4% females, Figure 20).

### Colorectal cancer and FBC reference ranges

For all FBC parameters, the graphical trends showed that levels remained in the reference range for both cases and controls, except red blood cell count, haemoglobin, haematocrit, mean corpuscular volume, and mean platelet volume. In these five parameters, the trends suggest blood levels often only reach abnormal thresholds within approximately six months of diagnosis in younger cases. However, in older cases, levels are abnormal for approximately three years before diagnosis, which was also observed for older controls.

### Tumour staging and the FBC

**Figure 21.**
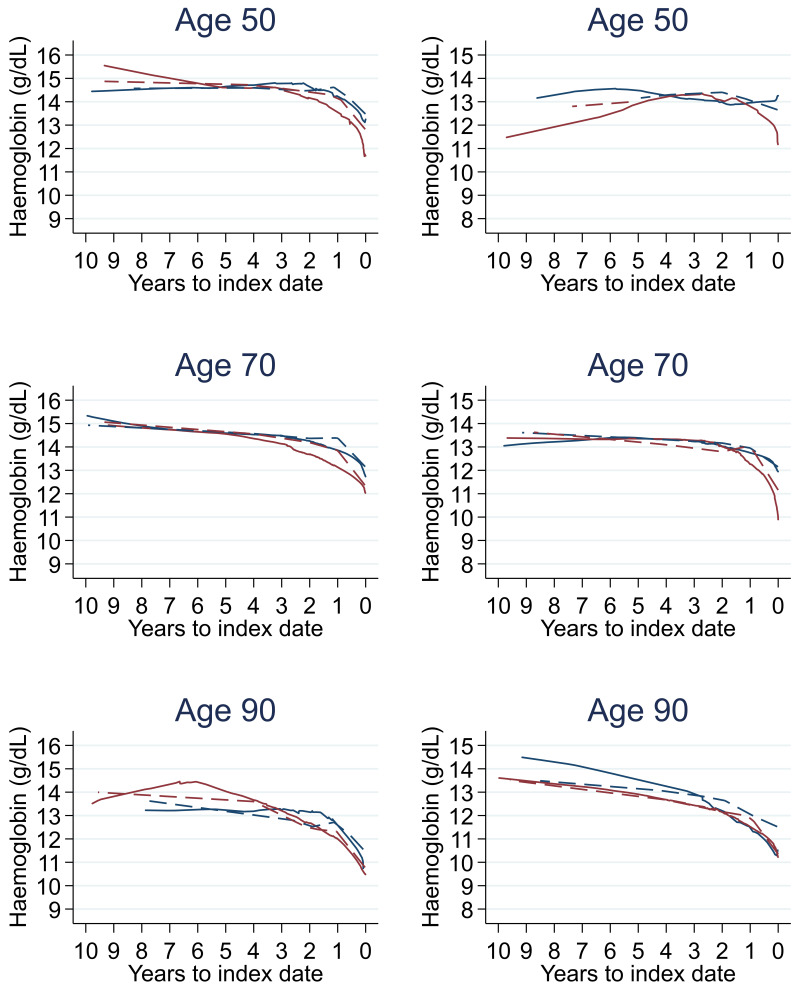
Haemoglobin trends between Duke’s A and D tumour stage by age at index date ^1,2^. ^1^Index date was the date of diagnosis for cases and a randomly selected date in the patient’s study period for controls. ^2^LOWESS trends are age (+/- 3 years) and modelled (fixed effects) trends are taken at that specific age. Legend: males (left) and females (right). Stage A (blue line) and Stage D (red line). LOWESS trend (solid line) and modelled trend (dashed line).

**Figure 22.**
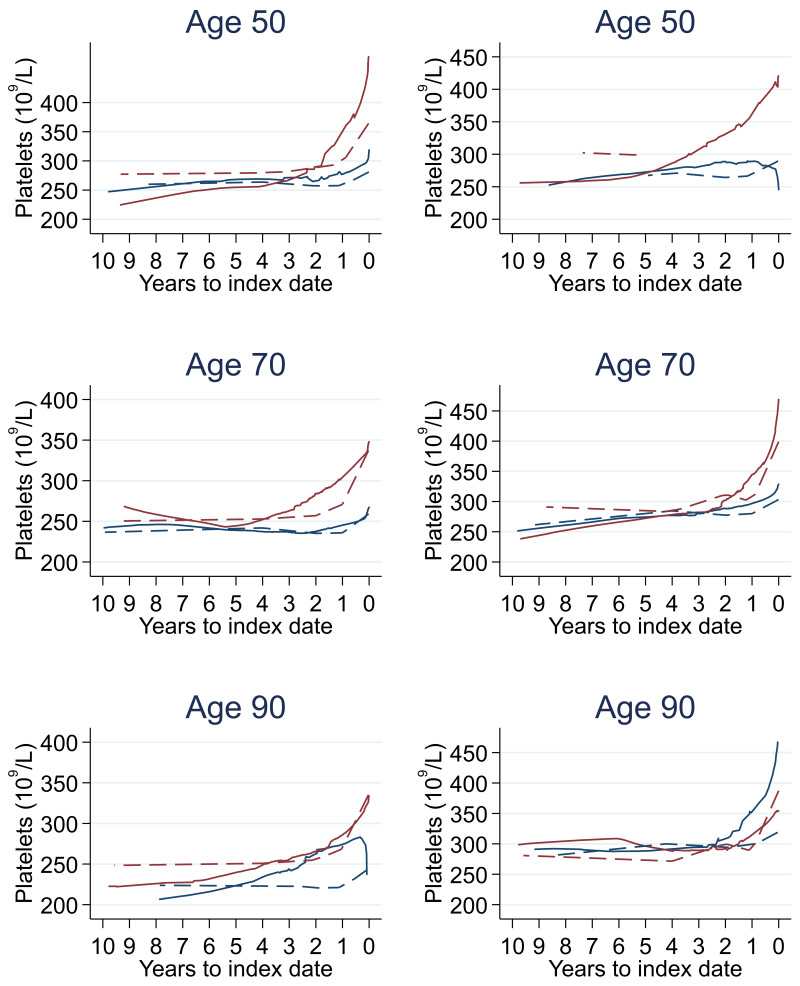
Platelet trends between Duke’s A and D tumour stage by age at index date ^1,2^. ^1^Index date was the date of diagnosis for cases and a randomly selected date in the patient’s study period for controls. ^2^LOWESS trends are age (+/- 3 years) and modelled (fixed effects) trends are taken at that specific age. Legend: males (left) and females (right). Stage A (blue line) and Stage D (red line). LOWESS trend (solid line) and modelled trend (dashed line).

The number of cases diagnosed per Duke’s tumour stage is in Table 12. Mixed models including Duke’s stage at diagnosis, developed using cases alone, are provided for red blood cell-related parameters in Table 13 (males) and Table 14 (males), platelet-related in Table 15, and white blood cell-related in Table 16 (males) and Table 17 (females). In the raw data (LOWESS curves), there appeared to be no difference in trends over time between Stage A and Stage D tumours among older patients. However, changes started up to one year earlier in patients with Stage D in younger patients (see Figure 21 for haemoglobin and Figure 22 for platelets – for the remaining parameters, please see ‘Data availability’). This was observed in all FBC parameters except mean platelet volume, basophil count, eosinophil count, and lymphocyte count for both males and females, which showed no apparent difference between tumour stages.

**Table 12.** Number of cases per Duke’s tumour stage at diagnosis.

Duke’s stage	Males		Females	
	Number	% of cases	Number	% of cases
**A**	850	9.2%	688	8.4%
**B**	2,049	22.1%	1,778	21.8%
**C**	2,038	22.0%	1,797	22.0%
**D**	737	7.8%	532	6.5%
**Unknown**	3,581	38.7%	3,358	41.2%
**Total**	9,255	100%	8,153	100%

**Table 13.** Mixed effect model coefficients for red blood cell count parameters (cases only) containing the Duke’s stage at diagnosis - males.

Variable	RBC	Hb	Hc	MCV	MCH	MCHC
*N*	1515	1571	423	1551	1503	1427
*n cases*	725	736	214	711	706	661
Fixed effects:						
Constant	4.4035 (3.5802, 5.2267)	9.9164 (6.8338, 12.9990)	0.4394 (0.2527, 0.6261)	76.8717 (67.8548, 85.8886)	26.1164 (22.8572, 29.3757)	30.6694 (28.9221, 32.4166)
Age at index date ^1^ (years)	0.0059 (-0.0088, 0.0206)	0.0674 (0.0122, 0.1225)	-0.0006 (-0.0040, 0.0027)	0.1869 (0.0271, 0.3467)	0.0597 (0.0019, 0.1174)	0.0403 (0.0094, 0.0713)
Age at index date ^1^ – knot at 60 (years)	-0.0341 (-0.0595, -0.0088)	-0.1571 (-0.2520, -0.0621)	-0.0019 (-0.0077, 0.0038)	-0.1782 (-0.4420, 0.0856)	-0.0656 (-0.1608, 0.0296)	-0.0575 (-0.1081, -0.0069)
Age at index date ^1^ – knot at 70 (years)	0.0255 (0.0007, 0.0503)	0.0398 (-0.0524, 0.1321)	0.0030 (-0.0024, 0.0084)	-0.1188 (-0.3732, 0.1356)	-0.0565 (-0.1482, 0.0351)	-0.0092 (-0.0575, 0.0392)
Age at index date ^1^ – knot at 80 (years)	-0.0526 (-0.0852, -0.0200)	-0.0548 (-0.1697, 0.0601)	-0.0011 (-0.0071, 0.0048)	0.2693 (-0.0910, 0.6297)	0.0847 (-0.0445, 0.2139)	-0.0229 (-0.0930, 0.0471)
Time to index date ^1^ (years)	0.3864 (-0.5294, 1.3021)	5.3873 (1.7396, 9.0351)	0.0942 (-0.1498, 0.3382)	3.1593 (2.5114, 3.8072)	1.1156 (0.8740, 1.3571)	0.6050 (0.4330, 0.7770)
Time to index date ^1^ date – knot at one (years)	-0.2071 (-1.8449, 1.4307)	-7.4027 (-13.8704, -0.9350)	-0.1852 (-0.5750, 0.2045)	-3.5320 (-4.6623, -2.4018)	-1.2569 (-1.6765, -0.8373)	-0.6380 (-0.9382, -0.3377)
Time to index date ^1^ date – knot at two (years)	-0.2240 (-1.4605, 1.0125)	1.4891 (-3.3180, 6.2961)	0.0021 (-0.3250, 0.3292)	0.5712 (-0.2145, 1.3568)	0.2999 (0.0082, 0.5916)	0.2018 (-0.0068, 0.4104)
Time to index date ^1^ date – knot at four (years)	-0.1963 (-0.8706, 0.4779)	0.2407 (-2.4176, 2.8990)	0.0804 (-0.2513, 0.4120)	-0.0318 (-0.3913, 0.3277)	-0.1184 (-0.2531, 0.0163)	-0.1839 (-0.2781, -0.0897)
Dukes D (vs. A)	1.2082 (0.1845, 2.2319)	-0.3514 (-3.8808, 3.1780)	-0.0991 (-0.3154, 0.1172)	-13.3449 (-27.1015, 0.4117)	-5.5770 (-10.5296, -0.6245)	-0.6897 (-3.3618, 1.9824)
Dukes D (vs. A) by time to index date ^1^ interaction	0.0133 (-0.0550, 0.0817)	0.2794 (0.0148, 0.5441)	-0.0214 (-0.0367, -0.0060)	1.2163 (0.3164, 2.1162)	0.6000 (0.2613, 0.9388)	0.0991 (-0.1409, 0.3391)
Dukes D (vs. A) by time to index date ^1^ (knot at one) interaction	0.0410 (-0.0818, 0.1639)	0.0667 (-0.4056, 0.5391)	0.0558 (0.0280, 0.0835)	-0.1100 (-1.7108, 1.4908)	-0.1705 (-0.7691, 0.4280)	0.0550 (-0.3734, 0.4834)
Dukes D (vs. A) by time to index date ^1^ (knot at two) interaction	-0.0298 (-0.1197, 0.0601)	-0.2409 (-0.5837, 0.1019)	-0.0352 (-0.0562, -0.0141)	-0.7959 (-1.9493, 0.3576)	-0.2955 (-0.7248, 0.1337)	-0.1125 (-0.4221, 0.1970)
Dukes D (vs. A) by time to index date ^1^ (knot at four) interaction	-0.0231 (-0.0664, 0.0201)	-0.0772 (-0.2428, 0.0883)	-0.0001 (-0.0118, 0.0117)	-0.4362 (-0.9805, 0.1081)	-0.1503 (-0.3543, 0.0537)	-0.0132 (-0.1575, 0.1312)
Time by age interaction	-0.0052 (-0.0215, 0.0111)	-0.0768 (-0.1417, -0.0118)	-0.0010 (-0.0053, 0.0033)			
Time (knot at one) by age interaction	0.0028 (-0.0264, 0.0320)	0.1098 (-0.0053, 0.2250)	0.0025 (-0.0044, 0.0094)			
Time (knot at two) by age interaction	0.0035 (-0.0185, 0.0256)	-0.0207 (-0.1062, 0.0647)	0.0001 (-0.0056, 0.0059)			
Time (knot at four) by age interaction	0.0029 (-0.0091, 0.0148)	-0.0070 (-0.0540, 0.0399)	-0.0014 (-0.0071, 0.0043)			
Time by age (knot at 60) interaction	0.0215 (-0.0059, 0.0488)	0.1336 (0.0256, 0.2415)	0.0042 (-0.0025, 0.0108)			
Time (knot at one) by age (knot at 60) interaction	-0.0229 (-0.0721, 0.0263)	-0.1587 (-0.3506, 0.0332)	-0.0069 (-0.0181, 0.0044)			
Time (knot at two) by age (knot at 60) interaction	-0.0038 (-0.0403, 0.0326)	-0.0063 (-0.1461, 0.1335)	0.0009 (-0.0081, 0.0100)			
Time (knot at four) by age (knot at 60) interaction	0.0049 (-0.0136, 0.0234)	0.0310 (-0.0411, 0.1030)	0.0010 (-0.0064, 0.0085)			
Time by age (knot at 70) interaction	-0.0288 (-0.0534, -0.0042)	-0.0989 (-0.1946, -0.0032)	-0.0062 (-0.0120, -0.0005)			
Time (knot at one) by age (knot at 70) interaction	0.0374 (-0.0065, 0.0814)	0.1150 (-0.0548, 0.2849)	0.0086 (-0.0019, 0.0191)			
Time (knot at two) by age (knot at 70) interaction	0.0040 (-0.0271, 0.0351)	0.0200 (-0.0989, 0.1389)	-0.0022 (-0.0100, 0.0055)			
Time (knot at four) by age (knot at 70) interaction	-0.0196 (-0.0338, -0.0053)	-0.0401 (-0.0950, 0.0149)	0.0006 (-0.0037, 0.0049)			
Time by age (knot at 80) interaction	0.0345 (0.0115, 0.0575)	0.0858 (-0.0041, 0.1756)	0.0027 (-0.0020, 0.0074)			
Time (knot at one) by age (knot at 80) interaction	-0.0484 (-0.0889, -0.0078)	-0.1608 (-0.3179, -0.0037)	-0.0063 (-0.0148, 0.0021)			
Time (knot at two) by age (knot at 80) interaction	0.0031 (-0.0270, 0.0332)	0.0706 (-0.0441, 0.1853)	0.0034 (-0.0031, 0.0099)			
Time (knot at four) by age (knot at 80) interaction	0.0161 (0.0008, 0.0313)	0.0029 (-0.0560, 0.0619)	0.0003 (-0.0036, 0.0042)			
Dukes D (vs. A) by age interaction	-0.0232 (-0.0415, -0.0050)	-0.0068 (-0.0696, 0.0559)	0.0015 (-0.0023, 0.0053)	0.1972 (-0.0471, 0.4414)	0.0831 (-0.0048, 0.1710)	0.0103 (-0.0370, 0.0577)
Dukes D (vs. A) by age (knot at 60) interaction	0.0375 (0.0065, 0.0685)	0.0010 (-0.1040, 0.1061)	-0.0011 (-0.0074, 0.0052)	-0.3402 (-0.7459, 0.0654)	-0.1877 (-0.3337, -0.0417)	-0.0645 (-0.1428, 0.0137)
Dukes D (vs. A) by age (knot at 70) interaction	-0.0200 (-0.0502, 0.0103)	0.0313 (-0.0685, 0.1311)	-0.0010 (-0.0068, 0.0047)	0.3235 (-0.0607, 0.7077)	0.2145 (0.0766, 0.3524)	0.0881 (0.0146, 0.1615)
Dukes D (vs. A) by age (knot at 80) interaction	0.0258 (-0.0112, 0.0628)	-0.0375 (-0.1562, 0.0812)	0.0024 (-0.0039, 0.0088)	-0.5334 (-0.9972, -0.0697)	-0.2267 (-0.3930, -0.0604)	-0.0222 (-0.1116, 0.0672)
Random effects:						
Intercept for patient (variance)	0.1923 (0.1756, 0.2107)	2.9902 (2.7319, 3.2730)	0.0023 (0.0019, 0.0027)	45.3333 (41.6183, 49.3800)	5.3157 (4.8602, 5.8138)	1.3501 (1.2131, 1.5027)
Slope for time to index date ^1^ (variance)	0.0027 (0.0021, 0.0033)	0.0770 (0.0650, 0.0913)	0.0001 (0.0000, 0.0001)	0.8162 (0.6868, 0.9701)	0.1059 (0.0895, 0.1252)	0.0260 (0.0210, 0.0321)
Covariance between Intercept for patient and slope for time to index date (covariance)	-0.0117 (-0.0143, -0.0090)	-0.3652 (-0.4175, -0.3128)	-0.0003 (-0.0004, - 0.0002)	-4.7011 (-5.3479, -4.0543)	-0.5593 (-0.6397, -0.4789)	-0.1499 (-0.1752, -0.1245)
Residual (variance)	0.0600 (0.0574, 0.0627)	0.9575 (0.9180, 0.9988)	0.0007 (0.0006, 0.0008)	10.7772 (10.3277, 11.2462)	1.4429 (1.3814, 1.5071)	0.7146 (0.6834, 0.7472)

^1^Index date was the date of diagnosis for cases and a randomly selected date in the patient’s study period for controls. Abbreviations: RBC = red blood cells; Hb = haemoglobin; Hc = haematocrit; MCV = mean corpuscular volume; MCH = mean corpuscular haemoglobin; MCHC = mean corpuscular haemoglobin concentration.

**Table 14.** Mixed effect model coefficients for red blood cell count parameters (cases only) containing the Duke’s stage at diagnosis - females.

Variable	RBC	Hb	Hc	MCV	MCH	MCHC
*N*	1155	1213	332	1194	1134	1100
*n cases*	499	529	152	522	489	469
Fixed effects:						
Constant	4.6958 (3.7582, 5.6334)	12.6106 (9.0741, 16.1471)	0.4568 (0.2469, 0.6667)	77.7683 (66.4981, 89.0385)	26.8108 (22.9838, 30.6379)	33.4073 (31.2499, 35.5647)
Age at index date ^1^ (years)	-0.0054 (-0.0221, 0.0114)	0.0046 (-0.0586, 0.0679)	-0.0012 (-0.0050, 0.0026)	0.1753 (-0.0262, 0.3769)	0.0408 (-0.0275, 0.1092)	-0.0123 (-0.0508, 0.0262)
Age at index date ^1^ – knot at 60 (years)	0.0065 (-0.0224, 0.0355)	-0.0601 (-0.1692, 0.0491)	-0.0013 (-0.0078, 0.0053)	-0.4503 (-0.7993, -0.1013)	-0.1080 (-0.2259, 0.0098)	0.0091 (-0.0563, 0.0745)
Age at index date ^1^ – knot at 70 (years)	-0.0251 (-0.0524, 0.0022)	-0.0546 (-0.1570, 0.0479)	0.0014 (-0.0049, 0.0076)	0.2975 (-0.0389, 0.6338)	0.0211 (-0.0926, 0.1348)	-0.0229 (-0.0836, 0.0378)
Age at index date ^1^ – knot at 80 (years)	0.0205 (-0.0085, 0.0495)	0.1426 (0.0365, 0.2488)	0.0070 (0.0002, 0.0138)	0.0828 (-0.2991, 0.4647)	0.0892 (-0.0411, 0.2195)	0.0060 (-0.0618, 0.0739)
Time to index date ^1^ (years)	-0.3456 (-1.4876, 0.7965)	-2.5994 (-6.8355, 1.6367)	-0.2053 (-0.5182, 0.1076)	3.3268 (2.5960, 4.0576)	1.2300 (0.9639, 1.4962)	0.4343 (0.2523, 0.6162)
Time to index date ^1^ date – knot at one (years)	0.1585 (-2.0315, 2.3485)	3.2106 (-4.7104, 11.1316)	0.2821 (-0.2574, 0.8216)	-2.7413 (-3.9939, -1.4888)	-1.0680 (-1.5217, -0.6143)	-0.2886 (-0.5989, 0.0217)
Time to index date ^1^ date – knot at two (years)	0.0743 (-1.6653, 1.8139)	-1.0014 (-7.1529, 5.1501)	-0.0790 (-0.4257, 0.2677)	-0.5796 (-1.4373, 0.2781)	-0.1513 (-0.4610, 0.1583)	-0.1554 (-0.3666, 0.0558)
Time to index date ^1^ date – knot at four (years)	0.1521 (-0.7876, 1.0918)	0.1373 (-3.2328, 3.5075)	-0.0025 (-0.3025, 0.2975)	0.1417 (-0.2589, 0.5423)	0.0508 (-0.0943, 0.1960)	0.0769 (-0.0214, 0.1751)
Dukes D (vs. A)	-0.2849 (-1.3157, 0.7459)	0.6777 (-2.9215, 4.2769)	0.0177 (-0.1976, 0.2331)	3.1397 (-12.8396, 19.1190)	0.3363 (-5.1398, 5.8123)	-0.2814 (-3.3824, 2.8197)
Dukes D (vs. A) by time to index date ^1^ interaction	0.2006 (0.1239, 0.2773)	1.0733 (0.7859, 1.3608)	0.0245 (0.0065, 0.0425)	1.6564 (0.5941, 2.7188)	0.8505 (0.4597, 1.2413)	0.3606 (0.0935, 0.6278)
Dukes D (vs. A) by time to index date ^1^ (knot at one) interaction	-0.2790 (-0.4140, - 0.1440)	-1.5478 (-2.0524, -1.0433)	-0.0226 (-0.0542, 0.0089)	-1.9403 (-3.8032, -0.0775)	-0.9683 (-1.6478, -0.2887)	-0.6136 (-1.0834, -0.1438)
Dukes D (vs. A) by time to index date ^1^ (knot at two) interaction	0.0709 (-0.0236, 0.1654)	0.5693 (0.2189, 0.9197)	-0.0038 (-0.0256, 0.0180)	0.7481 (-0.5538, 2.0499)	0.3231 (-0.1483, 0.7946)	0.4441 (0.1153, 0.7729)
Dukes D (vs. A) by time to index date ^1^ (knot at four) interaction	0.0386 (-0.0053, 0.0824)	-0.0479 (-0.2079, 0.1122)	0.0005 (-0.0109, 0.0118)	-0.7502 (-1.3526, -0.1477)	-0.3024 (-0.5215, -0.0832)	-0.2300 (-0.3835, -0.0765)
Time by age interaction	0.0082 (-0.0119, 0.0284)	0.0549 (-0.0201, 0.1299)	0.0040 (-0.0015, 0.0096)			
Time (knot at one) by age interaction	-0.0053 (-0.0437, 0.0330)	-0.0611 (-0.2004, 0.0783)	-0.0057 (-0.0153, 0.0039)			
Time (knot at two) by age interaction	-0.0004 (-0.0307, 0.0299)	0.0152 (-0.0924, 0.1227)	0.0018 (-0.0044, 0.0080)			
Time (knot at four) by age interaction	-0.0042 (-0.0206, 0.0123)	-0.0081 (-0.0673, 0.0510)	-0.0002 (-0.0054, 0.0050)			
Time by age (knot at 60) interaction	-0.0215 (-0.0539, 0.0110)	-0.0584 (-0.1808, 0.0639)	-0.0053 (-0.0145, 0.0040)			
Time (knot at one) by age (knot at 60) interaction	0.0241 (-0.0348, 0.0829)	0.0458 (-0.1729, 0.2646)	0.0109 (-0.0053, 0.0270)			
Time (knot at two) by age (knot at 60) interaction	-0.0070 (-0.0513, 0.0373)	-0.0004 (-0.1620, 0.1613)	-0.0058 (-0.0164, 0.0049)			
Time (knot at four) by age (knot at 60) interaction	0.0126 (-0.0114, 0.0365)	0.0428 (-0.0447, 0.1304)	0.0006 (-0.0065, 0.0077)			
Time by age (knot at 70) interaction	0.0266 (-0.0011, 0.0542)	0.0572 (-0.0477, 0.1621)	0.0012 (-0.0063, 0.0087)			
Time (knot at one) by age (knot at 70) interaction	-0.0204 (-0.0681, 0.0273)	0.0328 (-0.1478, 0.2133)	-0.0055 (-0.0185, 0.0075)			
Time (knot at two) by age (knot at 70) interaction	-0.0038 (-0.0367, 0.0292)	-0.0902 (-0.2144, 0.0341)	0.0033 (-0.0057, 0.0122)			
Time (knot at four) by age (knot at 70) interaction	-0.0115 (-0.0273, 0.0042)	-0.0464 (-0.1054, 0.0125)	0.0013 (-0.0030, 0.0056)			
Time by age (knot at 80) interaction	-0.0184 (-0.0432, 0.0064)	-0.1069 (-0.2011, -0.0128)	-0.0036 (-0.0095, 0.0023)			
Time (knot at one) by age (knot at 80) interaction	0.0009 (-0.0425, 0.0444)	0.0114 (-0.1529, 0.1756)	0.0034 (-0.0069, 0.0138)			
Time (knot at two) by age (knot at 80) interaction	0.0158 (-0.0142, 0.0459)	0.0992 (-0.0132, 0.2117)	0.0025 (-0.0047, 0.0097)			
Time (knot at four) by age (knot at 80) interaction	0.0097 (-0.0035, 0.0228)	0.0357 (-0.0130, 0.0843)	-0.0027 (-0.0064, 0.0010)			
Dukes D (vs. A) by age interaction	0.0010 (-0.0175, 0.0196)	-0.0334 (-0.0980, 0.0312)	-0.0006 (-0.0044, 0.0032)	-0.1143 (-0.4021, 0.1736)	-0.0245 (-0.1233, 0.0743)	-0.0005 (-0.0563, 0.0554)
Dukes D (vs. A) by age (knot at 60) interaction	0.0027 (-0.0308, 0.0363)	0.0653 (-0.0498, 0.1805)	-0.0016 (-0.0082, 0.0049)	0.3876 (-0.1364, 0.9116)	0.0511 (-0.1294, 0.2316)	-0.0243 (-0.1242, 0.0755)
Dukes D (vs. A) by age (knot at 70) interaction	-0.0003 (-0.0340, 0.0334)	-0.0143 (-0.1279, 0.0993)	0.0047 (-0.0018, 0.0112)	-0.4225 (-0.9505, 0.1055)	-0.0322 (-0.2135, 0.1490)	0.0490 (-0.0481, 0.1462)
Dukes D (vs. A) by age (knot at 80) interaction	-0.0032 (-0.0364, 0.0301)	-0.0517 (-0.1629, 0.0594)	-0.0071 (-0.0138, - 0.0004)	-0.0146 (-0.5356, 0.5064)	-0.0571 (-0.2347, 0.1205)	-0.0383 (-0.1326, 0.0559)
Random effects:						
Intercept for patient (variance)	0.1515 (0.1361, 0.1685)	2.4850 (2.2428, 2.7534)	0.0019 (0.0015, 0.0024)	46.3906 (42.0590, 51.1684)	4.7755 (4.3019, 5.3013)	1.4257 (1.2693, 1.6013)
Slope for time to index date ^1^ (variance)	0.0021 (0.0017, 0.0027)	0.0564 (0.0465, 0.0684)	0.0001 (0.0000, 0.0001)	0.8589 (0.6965, 1.0592)	0.0950 (0.0773, 0.1169)	0.0259 (0.0204, 0.0330)
Covariance between Intercept for patient and slope for time to index date (covariance)	-0.0097 (-0.0121, -0.0072)	-0.2864 (-0.3327, -0.2401)	-0.0003 (-0.0004, -0.0002)	-4.1096 (-4.8676, -3.3516)	-0.4127 (-0.4939, -0.3314)	-0.1432 (-0.1718, -0.1145)
Residual (variance)	0.0582 (0.0557, 0.0608)	0.8588 (0.8231, 0.8961)	0.0006 (0.0005, 0.0007)	11.9004 (11.3826, 12.4418)	1.4914 (1.4255, 1.5603)	0.6944 (0.6633, 0.7270)

^1^Index date was the date of diagnosis for cases and a randomly selected date in the patient’s study period for controls. Abbreviations: RBC = red blood cells; Hb = haemoglobin; Hc = haematocrit; MCV = mean corpuscular volume; MCH = mean corpuscular haemoglobin; MCHC = mean corpuscular haemoglobin concentration.

**Table 15.** Mixed effect model coefficients for platelet parameters (cases only) containing the Duke’s stage at diagnosis.

Variable	Males		Females	
	Platelets	MPV	Platelets	MPV
*N*	1528	211	1181	176
*n cases*	94	720	68	514
Fixed effects:				
Constant	362.7745 (244.9091, 480.6400)	10.1625 (9.0258, 11.2992)	228.8615 (80.0121, 377.7110)	9.3772 (8.2775, 10.4769)
Age at index date ^1^ (years)	-1.6245 (-3.7115, 0.4624)	-0.0159 (-0.0322, 0.0003)	1.2336 (-1.4253, 3.8925)	-0.0065 (-0.0218, 0.0088)
Age at index date ^1^ – knot at 60 (years)	1.0595 (-2.3577, 4.4768)		-1.0526 (-5.6207, 3.5154)	
Age at index date ^1^ – knot at 70 (years)	2.4923 (-0.7897, 5.7742)		-0.7692 (-5.1260, 3.5876)	
Age at index date ^1^ – knot at 80 (years)	-5.2987 (-9.9446, -0.6528)		3.0129 (-1.9186, 7.9443)	
Time to index date ^1^ (years)	-23.8380 (-32.9754, -14.7006)	0.2090 (-0.1216, 0.5395)	-24.0242 (-33.9622, -14.0861)	0.0973 (-0.2211, 0.4158)
Time to index date ^1^ date – knot at one (years)	23.2526 (7.2207, 39.2845)	-0.3958 (-0.9658, 0.1742)	21.6704 (4.6010, 38.7399)	-0.1026 (-0.6409, 0.4356)
Time to index date ^1^ date – knot at two (years)	3.9099 (-7.2401, 15.0600)	0.1521 (-0.2582, 0.5624)	5.8590 (-5.7724, 17.4905)	-0.0305 (-0.3859, 0.3249)
Time to index date ^1^ date – knot at four (years)	-4.1553 (-9.1666, 0.8560)	0.0945 (-0.1040, 0.2929)	-7.3941 (-12.7141, -2.0742)	0.0728 (-0.0984, 0.2441)
Dukes D (vs. A)	165.7902 (-11.5791, 343.1595)	-0.2669 (-0.7052, 0.1715)	316.5678 (104.2109, 528.9247)	-0.4065 (-0.8700, 0.0570)
Dukes D (vs. A) by time to index date ^1^ interaction	-44.5130 (-57.1080, -31.9180)	-0.1359 (-0.5956, 0.3238)	-75.3165 (-89.7311, -60.9018)	0.1330 (-0.3165, 0.5825)
Dukes D (vs. A) by time to index date ^1^ (knot at one) interaction	31.4208 (8.9414, 53.9002)	0.1574 (-0.6725, 0.9873)	89.7211 (64.4038, 115.0384)	-0.2428 (-1.0078, 0.5222)
Dukes D (vs. A) by time to index date ^1^ (knot at two) interaction	7.8357 (-8.3477, 24.0191)	0.0784 (-0.5564, 0.7132)	-31.7440 (-49.3822, -14.1057)	0.2785 (-0.2305, 0.7875)
Dukes D (vs. A) by time to index date ^1^ (knot at four) interaction	5.6127 (-1.9590, 13.1844)	-0.1989 (-0.5133, 0.1155)	22.9637 (14.9723, 30.9552)	-0.2549 (-0.5125, 0.0028)
Dukes D (vs. A) by age interaction	-1.6210 (-4.7706, 1.5287)		-3.8954 (-7.7167, -0.0740)	
Dukes D (vs. A) by age (knot at 60) interaction	2.6723 (-2.5642, 7.9088)		5.0388 (-1.8602, 11.9379)	
Dukes D (vs. A) by age (knot at 70) interaction	-4.3882 (-9.3424, 0.5659)		-1.5712 (-8.4503, 5.3079)	
Dukes D (vs. A) by age (knot at 80) interaction	7.9080 (1.9337, 13.8823)		-2.1388 (-8.8827, 4.6052)	
Random effects:				
Intercept for patient (variance)	7.0e+03 (6.4e+03, 7.7e+03)	1.5550 (1.2196, 1.9824)	8.4e+03 (7.6e+03, 9.2e+03)	1.4818 (1.1520, 1.9060)
Slope for time to index date ^1^ (variance)	104.8360 (85.6861, 128.2657)	0.0265 (0.0159, 0.0441)	96.2314 (77.1465, 120.0376)	0.0194 (0.0121, 0.0312)
Covariance between Intercept for patient and slope for time to index date (covariance)	-6.7e+02 (-7.7e+02, -5.7e+02)	-0.1070 (-0.1661, -0.0478)	-6.6e+02 (-7.8e+02, -5.5e+02)	-0.0873 (-0.1350, -0.0396)
Residual (variance)	2.1e+03 (2.1e+03, 2.2e+03)	0.3302 (0.2892, 0.3770)	2.2e+03 (2.1e+03, 2.3e+03)	0.2551 (0.2274, 0.2862)

^1^Index date was the date of diagnosis for cases and a randomly selected date in the patient’s study period for controls. Abbreviations: MPV = mean platelet volume.

**Table 16.** Mixed effect model coefficients for white blood cell count parameters (cases only) containing the Duke’s stage at diagnosis – males.

Variable	WBC	Basophils	Eosinophils	Lymphocytes	Monocytes	Neutrophils
*N*	1466	1381	1385	1419	1414	1428
*n cases*	673	654	656	670	667	691
Fixed effects:						
Constant	7.7472 (3.6877, 11.8068)	0.0674 (0.0512, 0.0837)	0.0812 (-0.0790, 0.2414)	2.7383 (2.0743, 3.4024)	0.6958 (0.3986, 0.9931)	6.2180 (3.5621, 8.8738)
Age at index date ^1^ (years)	-0.0077 (-0.0794, 0.0641)	-0.0000 (-0.0003, 0.0002)	0.0027 (0.0002, 0.0052)	-0.0131 (-0.0226, -0.0036)	-0.0017 (-0.0069, 0.0036)	-0.0321 (-0.0785, 0.0144)
Age at index date ^1^ – knot at 60 (years)	-0.0008 (-0.1151, 0.1136)				0.0038 (-0.0046, 0.0122)	0.0565 (-0.0019, 0.1150)
Age at index date ^1^ – knot at 70 (years)	0.0355 (-0.0571, 0.1282)		-0.0050 (-0.0097, -0.0003)		0.0007 (-0.0054, 0.0068)	
Age at index date ^1^ – knot at 80 (years)				0.0120 (-0.0303, 0.0544)		-0.0239 (-0.1081, 0.0603)
Age at index date ^1^ – knot at 85 (years)	-0.0098 (-0.3422, 0.3226)					
Age at index date ^1^ – knot at 90 (years)						
Time to index date ^1^ (years)	-0.3386 (-0.6635, -0.0137)	-0.0001 (-0.0010, 0.0009)	-0.0015 (-0.0046, 0.0015)	0.1539 (0.0033, 0.3044)	-0.0216 (-0.0636, 0.0205)	-0.3035 (-0.6557, 0.0487)
Time to index date ^1^ date – knot at one (years)	0.4575 (-0.1122, 1.0271)			-0.1788 (-0.4439, 0.0864)	0.0066 (-0.0684, 0.0816)	0.2374 (-0.3948, 0.8697)
Time to index date ^1^ date – knot at two (years)	-0.1188 (-0.5173, 0.2798)			0.0463 (-0.1407, 0.2333)	0.0040 (-0.0494, 0.0573)	0.1494 (-0.3053, 0.6040)
Time to index date ^1^ date – knot at four (years)	-0.0243 (-0.2011, 0.1526)			-0.0019 (-0.0843, 0.0804)	0.0095 (-0.0139, 0.0328)	-0.1059 (-0.3126, 0.1009)
Dukes D (vs. A)	-1.9183 (-8.0906, 4.2541)	0.0025 (-0.0034, 0.0084)	0.0425 (-0.1947, 0.2797)	-0.0489 (-0.2636, 0.1657)	0.1502 (0.1103, 0.1902)	-0.4425 (-4.4857, 3.6007)
Dukes D (vs. A) by time to index date ^1^ interaction	-1.3254 (-1.7719, -0.8789)	-0.0001 (-0.0015, 0.0013)	-0.0005 (-0.0050, 0.0040)	0.1569 (-0.0509, 0.3647)	-0.1632 (-0.2215, -0.1050)	-1.4907 (-1.9796, -1.0017)
Dukes D (vs. A) by time to index date ^1^ (knot at one) interaction	1.0373 (0.2407, 1.8338)			-0.1110 (-0.4831, 0.2611)	0.2058 (0.1002, 0.3115)	1.4853 (0.5924, 2.3782)
CRC present by time to index date ^1^ (knot at two) interaction	0.2554 (-0.3221, 0.8328)			-0.0800 (-0.3519, 0.1918)	-0.0628 (-0.1404, 0.0149)	-0.1088 (-0.7715, 0.5540)
Dukes D (vs. A) by time to index date ^1^ (knot at four) interaction	0.0695 (-0.1985, 0.3374)			0.0279 (-0.0981, 0.1538)	0.0201 (-0.0155, 0.0557)	0.1268 (-0.1886, 0.4423)
Dukes D (vs. A) by age interaction	0.0721 (-0.0373, 0.1814)		-0.0009 (-0.0046, 0.0028)			0.0412 (-0.0294, 0.1117)
Dukes D (vs. A) by age (knot at 60) interaction	-0.1128 (-0.2888, 0.0632)					-0.0626 (-0.1508, 0.0256)
Dukes D (vs. A) by age (knot at 70) interaction	0.0054 (-0.1323, 0.1431)		0.0025 (-0.0040, 0.0090)			
Dukes D (vs. A) by age (knot at 80) interaction						0.0471 (-0.0571, 0.1513)
Dukes D (vs. A) by age (knot at 85) interaction	0.0766 (-0.2908, 0.4441)					
Random effects:						
Intercept for patient (variance)	8.5090 (7.7695, 9.3188)	0.0017 (0.0014, 0.0019)	0.0299 (0.0267, 0.0335)	2.9781 (2.7073, 3.2759)	0.0508 (0.0452, 0.0570)	1.7479 (1.4838, 2.0591)
Slope for time to index date ^1^ (variance)	0.1028 (0.0806, 0.1311)	0.0000 (0.0000, 0.0000)	0.0002 (0.0001, 0.0003)	0.0111 (0.0073, 0.0170)	0.0003 (0.0002, 0.0005)	0.0550 (0.0419, 0.0722)
Covariance between Intercept for patient and slope for time to index date (covariance)	-0.7927 (-0.9275, -0.6580)	-0.0001 (-0.0002, -0.0001)	-0.0016 (-0.0021, -0.0010)	-0.1506 (-0.1994, -0.1018)	-0.0034 (-0.0043, -0.0024)	-0.1551 (-0.2124, -0.0979)
Residual (variance)	2.3451 (2.2429, 2.4520)	0.0016 (0.0015, 0.0017)	0.0213 (0.0203, 0.0223)	0.4792 (0.4578, 0.5016)	0.0421 (0.0403, 0.0440)	3.1919 (3.0522, 3.3380)

^1^Index date was the date of diagnosis for cases and a randomly selected date in the patient’s study period for controls. Abbreviations: WBC = white blood cell count.

**Table 17.** Mixed effect model coefficients for white blood cell count parameters (cases only) containing the Duke’s stage at diagnosis – females.

Variable	WBC	Basophils	Eosinophils	Lymphocytes	Monocytes	Neutrophils
*N*	1137	1055	1057	1073	1071	1077
*n cases*	471	462	463	470	468	494
Fixed effects:						
Constant	5.7967 (1.1308, 10.4626)	0.0685 (0.0518, 0.0853)	0.0165 (-0.1111, 0.1440)	2.0711 (1.6091, 2.5331)	0.5706 (0.3092, 0.8320)	5.5388 (3.2693, 7.8083)
Age at index date ^1^ (years)	0.0229 (-0.0602, 0.1060)	0.0000 (-0.0002, 0.0002)	0.0029 (0.0009, 0.0050)	0.0010 (-0.0054, 0.0074)	-0.0008 (-0.0055, 0.0039)	-0.0186 (-0.0584, 0.0212)
Age at index date ^1^ – knot at 60 (years)	0.0544 (-0.0831, 0.1920)				0.0066 (-0.0018, 0.0149)	0.0410 (-0.0101, 0.0921)
Age at index date ^1^ – knot at 70 (years)	-0.0761 (-0.1823, 0.0300)		-0.0043 (-0.0078, -0.0007)		-0.0051 (-0.0132, 0.0030)	
Age at index date ^1^ – knot at 80 (years)				-0.0381 (-0.0657, -0.0104)	0.0046 (-0.0031, 0.0122)	0.0323 (-0.0185, 0.0832)
Age at index date ^1^ – knot at 85 (years)	0.0569 (-0.1531, 0.2669)					
Age at index date ^1^ – knot at 90 (years)				0.1158 (0.0017, 0.2298)		
Time to index date ^1^ (years)	-0.7255 (-1.2054, -0.2455)	-0.0010 (-0.0019, -0.0000)	-0.0024 (-0.0051, 0.0002)	-0.0529 (-0.1992, 0.0934)	-0.0489 (-0.0835, -0.0144)	-0.6451 (-1.0030, -0.2871)
Time to index date ^1^ date – knot at one (years)	0.7098 (-0.1280, 1.5477)			-0.0228 (-0.2689, 0.2233)	0.0491 (-0.0109, 0.1091)	0.8206 (0.1925, 1.4486)
Time to index date ^1^ date – knot at two (years)	0.1504 (-0.4295, 0.7304)			0.1343 (-0.0333, 0.3019)	-0.0121 (-0.0537, 0.0295)	-0.1369 (-0.5769, 0.3031)
Time to index date ^1^ date – knot at four (years)	-0.2785 (-0.5369, -0.0200)			-0.0957 (-0.1750, -0.0164)	0.0089 (-0.0099, 0.0278)	-0.1555 (-0.3541, 0.0430)
Dukes D (vs. A)	7.2915 (0.7634, 13.8195)	-0.0047 (-0.0112, 0.0019)	0.1932 (0.0069, 0.3796)	-0.3276 (-0.5498, -0.1055)	0.1740 (0.1376, 0.2104)	1.6704 (1.3286, 2.0122)
Dukes D (vs. A) by time to index date ^1^ interaction	-1.1431 (-1.8428, -0.4434)	0.0007 (-0.0008, 0.0021)	-0.0010 (-0.0049, 0.0029)	0.3316 (0.1189, 0.5443)	-0.1791 (-0.2293, -0.1288)	-1.3494 (-1.8709, -0.8280)
Dukes D (vs. A) by time to index date ^1^ (knot at one) interaction	1.1213 (-0.1308, 2.3734)			-0.2867 (-0.6540, 0.0806)	0.1888 (0.0991, 0.2785)	1.1001 (0.1604, 2.0399)
CRC present by time to index date ^1^ (knot at two) interaction	-0.1675 (-1.0561, 0.7210)			-0.0714 (-0.3286, 0.1857)	-0.0134 (-0.0772, 0.0504)	0.1630 (-0.5133, 0.8393)
Dukes D (vs. A) by time to index date ^1^ (knot at four) interaction	0.3730 (-0.0208, 0.7668)			0.0909 (-0.0321, 0.2138)	0.0060 (-0.0230, 0.0351)	0.2539 (-0.0527, 0.5605)
Dukes D (vs. A) by age interaction	-0.0959 (-0.2133, 0.0215)		-0.0027 (-0.0056, 0.0002)			
Dukes D (vs. A) by age (knot at 60) interaction	0.0633 (-0.1418, 0.2685)					
Dukes D (vs. A) by age (knot at 70) interaction	0.0310 (-0.1353, 0.1973)		0.0025 (-0.0025, 0.0075)			
Dukes D (vs. A) by age (knot at 80) interaction						
Dukes D (vs. A) by age (knot at 85) interaction	0.0651 (-0.1961, 0.3264)					
Random effects:						
Intercept for patient (variance)	4.6264 (4.0525, 5.2815)	0.0017 (0.0015, 0.0020)	0.0188 (0.0167, 0.0212)	2.2505 (2.0433, 2.4788)	0.0356 (0.0314, 0.0403)	2.2038 (1.9020, 2.5535)
Slope for time to index date ^1^ (variance)	0.0449 (0.0283, 0.0713)	0.0000 (0.0000, 0.0000)	0.0002 (0.0002, 0.0003)	0.1144 (0.1011, 0.1295)	0.0004 (0.0003, 0.0005)	0.0190 (0.0114, 0.0319)
Covariance between Intercept for patient and slope for time to index date (covariance)	-0.3046 (-0.4090, -0.2001)	-0.0001 (-0.0002, -0.0001)	-0.0014 (-0.0018, -0.0011)	-0.4654 (-0.5169, -0.4139)	-0.0028 (-0.0035, -0.0021)	-0.1071 (-0.1568, -0.0575)
Residual (variance)	5.1725 (4.9509, 5.4041)	0.0014 (0.0013, 0.0014)	0.0080 (0.0076, 0.0084)	0.3481 (0.3309, 0.3661)	0.0233 (0.0222, 0.0245)	2.7399 (2.6150, 2.8707)

^1^Index date was the date of diagnosis for cases and a randomly selected date in the patient’s study period for controls. Abbreviations: WBC = white blood cell count.

### Sensitivity analysis

A matched design gave 54,198 males (9,033 cases, 45,165 controls) and 48,918 females (8,153 cases, 40,765 controls). For cases and controls separately, mean (SD) age at index was 70.8 (10.6) for males and 73.0 (11.8) for females and median (min-max) follow-up time from first FBC to index was 3.1 (0–10) years for males and 3.3 (0–10) years for females.

**Figure 23.**
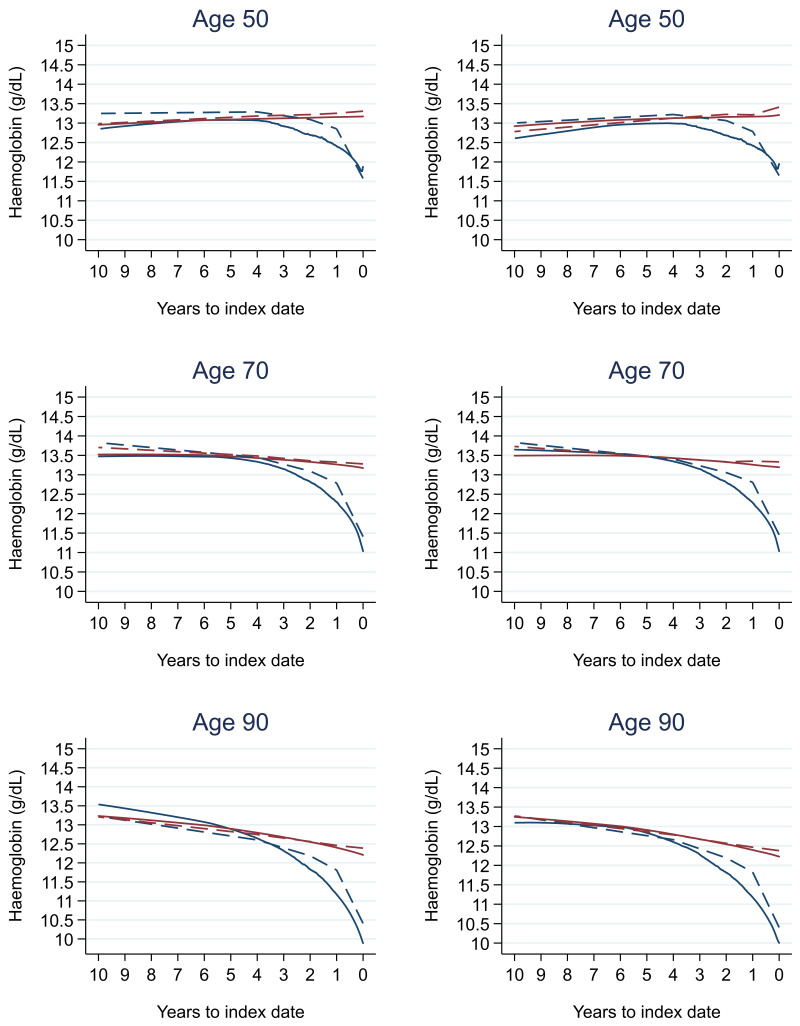
Haemoglobin trends for females by age at index ^1,2^: unmatched (left) and matched (right). ^1^Index date was the date of diagnosis for cases and a randomly selected date in the patient’s study period for controls. ^2^LOWESS trends are age (+/- 3 years) and modelled (fixed effects) trends are taken at that specific age. Legend: colorectal cancer (blue line) and no cancer (red line). LOWESS trend (solid line) and modelled trend (dashed line).

**Figure 24.**
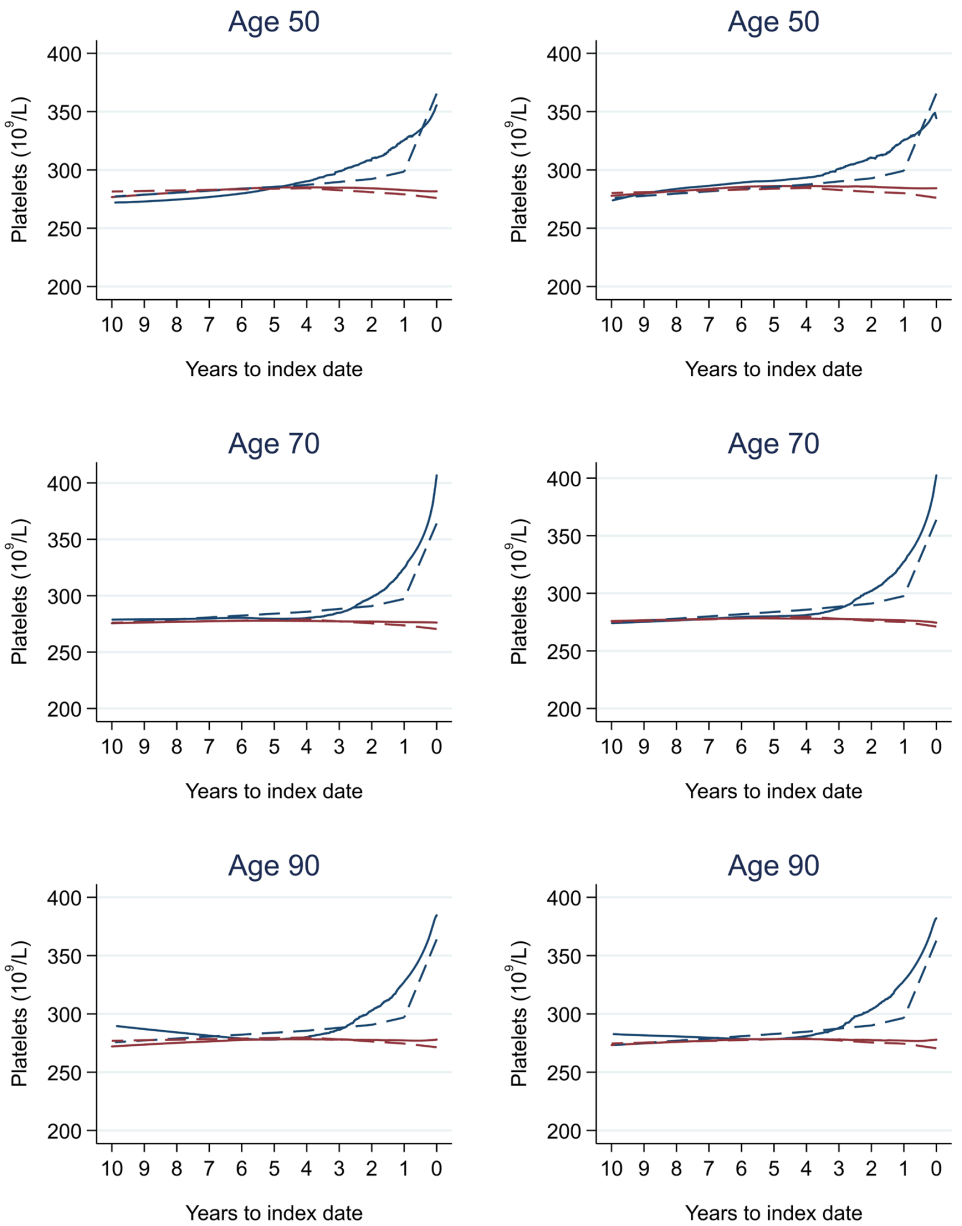
Platelets trends for females by age at index ^1,2^: unmatched (left) and matched (right). ^1^Index date was the date of diagnosis for cases and a randomly selected date in the patient’s study period for controls. ^2^LOWESS trends are age (+/- 3 years) and modelled (fixed effects) trends are taken at that specific age. Legend: colorectal cancer (blue line) and no cancer (red line). LOWESS trend (solid line) and modelled trend (dashed line).

There was no difference in graphical trends when using a matched design (see Figure 23 for haemoglobin and Figure 24 for platelets – for the remaining parameters, please see ‘Data availability’). Additionally, coefficients from mixed effects models changed only slightly, with 86.4% and 86.3% of coefficients changing by only <0.1 for males and females respectively – for the models, please see ‘Data availability’.

## Discussion

### Summary

We identified age- and sex-adjusted trends in many FBC parameters that differed between patients with and without a diagnosis within approximately four years before diagnosis. Differences in cases grew larger in the run up to diagnosis, with levels in patients without a diagnosis changing less rapidly over time. Trends may be more useful to identify cases than relying on FBC abnormalities or referral thresholds, as these thresholds were only reached close to diagnosis, with relevant trends present beforehand.

Summary statistics indicate an imbalance in age at index and follow-up time between cases and controls. Our sensitivity analysis showed no apparent differences between matched and unmatched designs. This was expected a priori, because in our unmatched design, graphical trends are already reported by age and sex separately. Additionally, (sex-stratified) mixed effects models included age, accounting for the imbalance. Furthermore, length of follow-up, despite imbalanced ( Figure 2), did not influence the trends, as there were many cases and controls with tests available at each time-point, increasing the quality/precision of the trend.

### Comparison with existing literature

Two prior studies assessed changes over time in haemoglobin between patients with and without colorectal cancer: one in an Israeli population ^11^ and one in a combined Swedish and Danish population ^12^. We report similar findings in a UK population, with haemoglobin levels that diverge around four years before diagnosis and a greater decline in the run up to diagnosis. We also report changes over time for many other FBC parameters. Another study used machine-learning methods to develop an algorithm called the ColonFlag, which assesses change over time in various parameters (at 18 and 36 months before index FBC) from a single patient to derive a monotone score for diagnosis from 0–100 in an Israeli population ( EarlySign) ^13^. It is unclear what trends are considered related to colorectal cancer. A fourth study used logistic regression to test whether the difference between the two most recent FBCs was associated with diagnosis for five parameters in UK primary care data ^14^. With the two tests performed at any time in a mean follow-up period of 6.3 years, they report no association in change in red blood cell count (p = 0.13), white blood cell count (p = 0.06), or haematocrit (p = 0.23) but do report an association for change in mean corpuscular volume (p = 0.04) and mean corpuscular haemoglobin (p = 0.02). However, our study suggests that red blood cell count, white blood cell count, and haematocrit do change over time due to colorectal cancer.

A recent study of haemoglobin levels in newly diagnosed colorectal cancer patients (mean age approximately 70 years) in Finland reported lower haemoglobin levels at the time of diagnosing higher stage tumours (13.2 g/dL at Stage 1 and 12.2 g/dL at Stage 4) ^15^. These levels are similar to those identified in this study ( Figure 21), where the trends in most FBC parameters were similar between Stage A and D colorectal cancers, but the divergence from controls started up to one year earlier in Stage D diagnoses compared to Stage A. This divergence in patients diagnosed with Stage A colorectal cancer often occurred within one year prior to diagnosis, suggesting a relatively short time window for detection between the earliest and latest stage.

When compared to NICE and WHO guidelines for anaemia, which could be due to any reason including iron-deficiency, our trends indicated haemoglobin levels often reached the threshold for males (<13 g/dL) and females (<12 g/dL) ( NICE: Suspected cancer recognition and referral, NICE: Anaemia - iron deficiency, WHO: anaemia) at approximately 6–12 months before diagnosis. Up to one year before diagnosis, anaemia was present in 48.8% of male cases and 54.3% of female cases. These results are similar to those reported in a previous UK primary care study of anaemia within one year prior to colorectal cancer diagnosis ^16^. Microcytic anaemia, commonly caused by iron-deficiency, which may warrant further investigation for colorectal cancer, was present in 23% of male cases and 28% of female cases within a year prior to diagnosis. Our study suggests there are relevant changes that occur up to three years before the presence of anaemia, including iron-deficiency anaemia, and these changes could be more helpful to facilitate early detection than relying on low haemoglobin levels.

We also compared the FBC results to normal reference ranges ( Oxford University Hospitals NHS UK). Abnormal FBC parameter levels are considered to represent health-related conditions or disease. Although differences in most FBC parameters between cases and controls grew larger as the time to diagnosis approached, they remained small overall and often remained in the normal reference range, except for red blood cell count, haemoglobin, haematocrit, mean corpuscular volume, and mean platelet volume. These parameters often only became abnormal close to diagnosis in younger cases but for many years in older cases, which was also observed in older controls. Therefore, such differences in trends between cases and controls may not be obvious to a clinician in general practice, as these differences would be considered to represent little-to-no concern, and the opportunity to utilise these changes over time to identify colorectal cancer would be missed. Our study supports the conclusions of another recent report that highlighted how the normal range does not necessarily reflect a healthy individual ^17^.

### Limitations

FBC blood tests are ordered for many reasons in primary care, not colorectal cancer specifically, but these reasons are not recorded in CPRD. Patients with FBCs are suspected to be generally less well than patients who are not tested (except in antenatal screening, which includes a FBC), so controls included in this study may not be entirely healthy. However, many existing studies identified in our recent systematic review have shown that in patients with FBCs, the test has potential to distinguish between patients with and without colorectal cancer ^4^.

Red blood cell distribution width is a parameter used to diagnose medical conditions, especially colorectal cancer ( Medline: Red cell distribution width). Historically, this parameter has not been reported to primary care practices until relatively recently, hence why almost all tests in our data have this value missing. Consequently, we excluded this relevant parameter from all analyses.

Due the nature of the case-control study design, we modelled 10 years of longitudinal data before index for each age group separately. Therefore, the trends in older age groups for many FBC parameters were subject to the ‘survivor effect’. For example, in Figure 5 (trends in haemoglobin), controls who survived to 90 years at index are likely healthier 10 years earlier than controls who survived to 80 years at index, who may or may not have survived to age 90. Thus, older patients would often have FBC levels that reflected healthier individuals 10 years earlier on average than patients diagnosed/censored at that younger age. This ‘survivor effect’ could not be adjusted for in our analyses.

Other known factors influencing colorectal cancer risk, such as ethnicity and family history, and FBC confounders, such as comorbidity status, were not available so were not included in our models. Many relevant FBC confounders, such as diet, vitamin use, and sleeping patterns, are not recorded in electronic health records. Nonetheless, age and sex are key characteristics to adjust trends. Adjustments for additional factors may be considered in future work.

Tumour stage at diagnosis was missing for approximately one-third of colorectal cancer diagnoses. Therefore, many cases were excluded from analyses of tumour staging, reducing sample size and precision of estimates.

### Implications for practice

The differences between cases and controls in the trends over time identified in this study would often go unnoticed in routine practice. It is difficult for busy clinicians filing results to notice minor changes in parameter values over time. Therefore, we are developing a dynamic statistical prediction model ^18, 19^ that makes use of trends in the FBC to derive an individual’s risk of diagnosis in the future. To develop the model, we will first determine the predictive value of patient-level trends – in this study, we only report that relevant trends exist and may be of help. The prediction model will aim to support the current colorectal cancer UK screening programme by identifying possible cases for investigation ( NHS: Bowel cancer screening). It will utilise trends in multiple FBC parameters over time to detect colorectal cancer at earlier time points than is possible using single parameter thresholds.

## Conclusions

Many FBC results change due to the presence of colorectal cancer. We identified differences in trends over a five-year period before diagnosis that differed to trends in patients without colorectal cancer. Such trends may pre-date single-value thresholds for referral for cancer investigation and blood-abnormality. They may therefore facilitate earlier detection, improving the likelihood of successful treatment and improved survival rates.

## Data availability

### Underlying data

The datasets used in this study are available from the CPRD. The CPRD maintain access rights to the data to ensure it is only used for research purposes by trustworthy organisations, so sharing of data is prohibited ( https://cprd.com/data-access). Checks are conducted on organisations carrying out and funding research to assess whether they are suitable to receive CPRD data. This is to ensure, as examples, that the data stays confidential, and it is only used for its approved purpose. An application to access the data can be made at https://cprd.com/data-access.

### Extended data

Additional full blood count parameters were analysed (tumour staging and sensitivity analysis) but are not related to the main claims in the article. These data are available from the authors on request.

## Consent

CPRD has ethical approval from the Health Research Authority to hold anonymised patient data and to support research using that data. CPRD’s approval of data access for individual research projects includes ethics approval and consent for those projects. Ethical approval was therefore covered for this study by the CPRD (protocol 14_195RMn2A2R).
