# Trends in the full blood count blood test and colorectal cancer detection: a longitudinal, case-control study of UK primary care patient data [version 2; peer review: 2 approved, 1 not approved]

**DOI:** 10.3310/nihropenres.13266.1

**Published:** 2022-10-18

**Authors:** Pradeep S. Virdee, Julietta Patnick, Peter Watkinson, Jacqueline Birks, Tim A. Holt

**Affiliations:** 1 Nuffield Department of Primary Care Health Sciences, University of Oxford, Oxford, OX2 6GG, UK; 2 Nuffield Department of Population Health, University of Oxford, Oxford, OX3 7LF, UK; 3 Kadoorie Centre for Critical Care Research and Education, Oxford University Hospitals NHS Trust, Oxford, OX3 9DU, UK; 4 Centre for Statistics in Medicine, NDORMS, University of Oxford, Oxford, OX3 7LD, UK

**Keywords:** full blood count, blood test, primary care, colorectal cancer, clinical practice research datalink

## Abstract

**Background:**

The full blood count (FBC) is a common blood test performed in general practice. It consists of many individual parameters that may change over time due to colorectal cancer. Such changes are likely missed in practice. We identified trends in these FBC parameters to facilitate early detection of colorectal cancer.

**Methods:**

We performed a retrospective, case-control, longitudinal analysis of UK primary care patient data. LOWESS smoothing and mixed effects models were derived to compare trends in each FBC parameter between patients diagnosed and not diagnosed over a prior 10-year period.

**Results:**

There were 399,405 males (2.3%, n = 9,255 diagnosed) and 540,544 females (1.5%, n = 8,153 diagnosed) in the study. There was no difference between cases and controls in FBC trends between 10 and four years before diagnosis. Within four years of diagnosis, trends in many FBC levels statistically significantly differed between cases and controls, including red blood cell count, haemoglobin, white blood cell count, and platelets (interaction between time and colorectal cancer presence: p <0.05). FBC trends were similar between Duke’s Stage A and D colorectal tumours, but started around one year earlier in Stage D diagnoses.

**Conclusions:**

Trends in FBC parameters are different between patients with and without colorectal cancer for up to four years prior to diagnosis. Such trends could help earlier identification.

## Introduction

Population incidence rates for colorectal cancer have been decreasing only slightly yearly since 2012. Colorectal cancer currently accounts for 11% of all new cancers diagnosed in the UK, being the fourth most common type of cancer. It is the second most common cause of cancer-related death. Prognosis is heavily influenced by tumour stage at diagnosis, which can be assessed in various ways. Five-year survival is 93% if diagnosed at Stage A, where the cancer is confined to the bowel lining, and 10% if at Stage D, where it has spread to other organs (Cancer Research UK - Bowel cancer statistics, Cancer Research UK – Bowel cancer survival).

Symptoms for colorectal cancer, such as abdominal pain and change in bowel habit, often appear when the disease has developed to a relatively late-stage, where it is difficult to treat and the likelihood of survival reduced. Current evidence suggests that symptoms are on average first reported to clinical care less than six months before diagnosis^1^. Identifying colorectal cancer at earlier stages, where the likelihood of survival is greatest and before overt symptoms appear, would be of considerable benefit to reduce mortality^2^.

The full blood count (FBC), a blood test commonly performed in primary care practices, may play a role in earlier detection^3^. For example, anaemia determined from the FBC test is a known risk factor for diagnosis and may warrant further investigation under the current screening programme if due to iron deficiency (WHO: Guide to early diagnosis, NICE: Suspected cancer recognition and referral). The FBC test consists of up to 20 individual parameters, including haemoglobin, platelet count, and white blood cell count.

Our recent systematic review identified 53 studies that assessed the FBC blood test for colorectal cancer diagnosis^4,5^. Our review indicated that diagnosed patients have a significantly lower red blood cell count, haemoglobin, and mean corpuscular volume and higher red blood cell distribution width, white blood cell count, and platelets within six months of diagnosis compared to patients not diagnosed. Smaller differences were observed compared to those observed earlier than six months before diagnosis, suggesting changes in the FBC differ over time. There may be relevant trends that could help identify patients who have a diagnosis.

The aim of this study was to identify trends in FBC parameter levels prior to colorectal cancer diagnosis and compare trends to those in patients without a diagnosis. To identify opportunities for earlier detection, we also assessed trends between tumour stages in diagnosed patients.

## Methods

Study reporting follows the Strengthening the Reporting of Observational Studies in Epidemiology (STROBE) guidelines^6^.

### Study design

We performed a retrospective case-control longitudinal study to explore changes in FBC results before a diagnosis compared to patients without a diagnosis. FBC data were obtained from a UK primary care database, the Clinical Practice Research Datalink (CPRD) GOLD, and diagnosis data from the UK National Cancer Registration and Analysis Service (NCRAS). CPRD has ethical approval from the Health Research Authority to hold anonymised patient data and support research using that data. The CPRD Independent Scientific Advisory Committee’s approval of data access for individual research projects includes ethics approval and consent for those projects. Ethical approval was therefore covered for this study by the CPRD (protocol 14_195RMn2A2R). Clinical codes to extract data from CPRD and NCRAS are in Table 1 and Table 2.

Study entry was defined as the latest date of registration with the practice, patient’s 40^th^ birthday, or 1^st^ January 2000. Study exit was defined as the earliest date of leaving the practice, date of death, or 14^th^ January 2014 (the NCRAS data-cut date).

### Participants

Patients with at least one FBC blood test within 10 years before index date (defined below) were included. Patients were excluded if registered with their primary care practice for less than one year, had a history of colorectal cancer before study entry, or diagnosed after study exit. Patients diagnosed with another cancer type before or simultaneously with colorectal cancer diagnosis were excluded. Patients with an available date of diagnosis but no indication of the cancer type were excluded.

### Clinical outcome

The outcome was the first diagnosis of colorectal cancer in the NCRAS database. For cases (patients diagnosed), the index date was the date of colorectal cancer diagnosis in the patient’s study period. For controls (patients without a diagnosis), the index date was a randomly selected date in the patient’s study period. A random date was chosen to mimic the sporadic nature of diagnoses in the overall study period for cases.

### Demographic and FBC variables

Year of birth and sex were available for all patients in the CPRD dataset. We extracted the date of each FBC test and included 14 of the 20 parameters (exposure variables of interest) that make up the FBC in this study. We excluded five: percentage basophils, eosinophils, lymphocytes, monocytes, and neutrophils because we used the corresponding counts (also FBC parameters). Additionally, red blood cell distribution width was excluded because this parameter is not recorded in general practice so was missing for almost all FBCs. We excluded FBC results outside biologically plausible ranges, such as negative values (see Table 3 for further details), FBCs performed earlier than 10 years before index date, and FBCs performed after index date.

Iron-deficiency anaemia warrants further investigation for colorectal cancer. Anaemia was defined as haemoglobin level <13 g/dL for men and <12 g/dL for women, as recommended by the National Institute for Health and Care Excellence (NICE) (NICE: Suspected cancer recognition and referral, NICE: anaemia - iron deficiency) and World Health Organisation (WHO) (WHO: anaemia). Microcytic anaemia, commonly present in iron-deficiency anaemia (Mean Corpuscular Volume), was defined as the presence of microcytosis (mean corpuscular volume <80 fL, as recommended by NICE (NICE: Investigations to confirm iron deficiency anaemia)) with anaemia.

Each FBC parameter has a normal or reference range of values considered to reflect a healthy individual (Mayo Clinic: Complete blood count). Reference ranges differ based on age and sex and may differ slightly across primary care practices (Royal Wolverhampton NHS UK, York Hospitals NHS UK, Maidstone and Tunbridge Wells NHS UK, Gloucestershire Hospitals NHS UK). We used reference ranges provided by the Department of Laboratory Haematology at Oxford University Hospitals Trust in this study (Table 4) (Oxford University Hospitals NHS UK) for comparison with trends.

A detailed account of our data preparation and validation processes has previously been reported^7^. We also previously provided summary statistics for each FBC parameter.

### Statistical analysis

#### Trends in raw data

We used LOWESS smoothing to describe trends in FBC parameters graphically for 10-yearly age groups. Controls with many FBCs are likely to have some other condition/disease, which could affect blood levels. Therefore, for both cases and controls, we randomly selected three FBCs per patient (if there were more than three) to reduce the influence of these many effected FBCs on LOWESS trends.

#### Modelling trends

Mixed effects models were developed for each FBC parameter separately (using all available FBCs per patient), using restricted maximum likelihood estimation. To model differences in FBC levels between cases and controls over time, colorectal cancer status (yes/no) and time to index date (years) were included as fixed effects together with an interaction between them. Each model was adjusted for age at index date (years) as a fixed effect and interactions between age and time and age and colorectal cancer status were included if trends over time or by colorectal cancer status differed by age group upon graphical inspection of LOWESS plots. Each patient was modelled using a random intercept and time using a random slope with an unstructured covariance matrix to account for correlation in repeated measures.

Non-linearity of continuous variables was based on visual inspection, Akaike information criteria, and Bayesian information criteria, which compared linear splines, restricted cubic splines, and fractional polynomials, and number of knots and knot locations^8–10^. Where non-linear, time to index date was modelled using piecewise linear splines with three knots: at one, two, and four years before index date. Age at index date was modelled using piecewise linear splines with knots at ages 60, 70, and 80 years for red blood cell-related parameters and platelet count and were variable for white blood cell count-related parameters.

#### Association of trends with cancer

Joint modelling of longitudinal and time-to-event data was used to quantify the association between FBC trends and cancer diagnosis. Joint modelling uses mixed effects modelling to model longitudinal data and are linked to a Cox model to provide hazard ratios (HRs) for outcomes. Univariate joint models (one for each FBC parameter) were developed. Mixed methods were as described above, except colorectal cancer status terms were removed because these are treated as the outcome in the linked time-to-event outcome for cancer presence. Due to the computationally intensive nature of joint models, these models were limited to a random sample of 50,000 patients.

#### Early detection opportunities

To identify opportunities for early detection, we assessed differences in FBC levels over time between cases diagnosed at Duke’s tumour Stage A (earliest stage) and D (latest stage). Mixed effects models were developed using the same methods described above but were limited to cases alone and included Duke’s tumour stage (A versus D) accordingly as fixed effects instead of colorectal cancer status. Joint models were not developed for Stage A versus D tumours due to limited sample sizes.

To explore the association between microcytic anaemia and diagnosis, we calculated the proportion of patients with microcytic anaemia per six-monthly time band, up to five years before index date. Microcytic anaemia presence was based on any FBC in the time band, if a patient had multiple, and proportions were calculated out of the number of patients in the time band. Additionally, we derived age-adjusted odds ratios (95% confidence interval (CI)) for microcytic anaemia presence using logistic regression for each time band separately. We visually compared trends to microcytic anaemia thresholds and FBC reference ranges to identify whether trends can pre-date single-value, iron-deficiency referral thresholds and blood-abnormality.

All analyses were stratified by sex. A two-sided significance level of 5% was used for all statistical analyses. Analyses were conducted using Stata/SE 15.1 (RRID: SCR_012763). Alternative, open-access software, such as R (RRID: SCR_001905), can perform the equivalent analyses.

### Sensitivity analysis

We recreated the trends and mixed effects models for each FBC parameter using a matched design. Cases and controls were matched 1:5 on age at index and follow-up time. Follow-up was time (years) from first FBC to index, converted into six-monthly bands for matching. A random index date within the control’s study period was used instead of the index date of their matched case because the latter heavily reduced the sample size. For example, many controls had an index date after study exit or had no FBCs before index.

## Results

**Figure 1:**
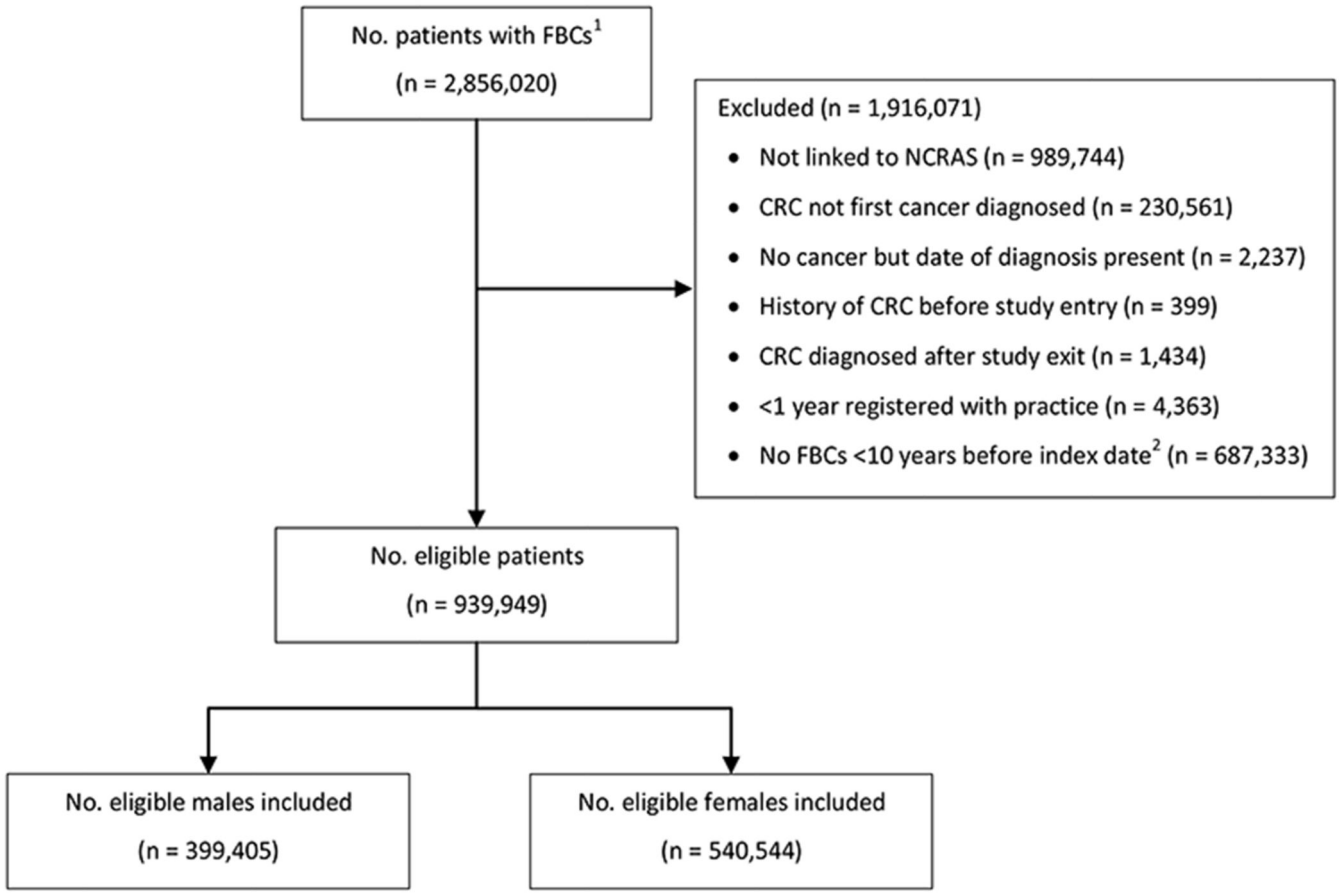
Patient flow chart. ^1^Number of patients available in the CPRD data extract. ^2^Index date was the date of diagnosis for cases and a randomly selected date in the patient’s study period for controls. Abbreviations: FBC = full blood count; CRC = colorectal cancer; NCRAS = National Cancer Registration and Analysis Service.

We identified 939,949 patients with at least one FBC within 10 years before index date who satisfied the eligibility criteria and were included in the study (Figure 1). There were 399,405 males with 1,193,619 FBC tests among them and 540,544 females with 1,874,609 FBC tests among them. See Table 5 for a description of the patient sample.

### The FBC test

On average, 14 of the 15 parameters were available within a FBC across FBCs for both males and females separately. Red blood cell distribution width was the FBC parameter missing for almost 100% of FBC tests for both males and females. This is likely because this parameter was historically not reported to the general practice by haematology laboratories, despite being automatically derived by haematology analysers (i.e. machines). Consequently, red blood cell distribution width was excluded from further analyses. Haemoglobin had the least amount of missing data, missing for 1.9% (n = 22,637) and 1.8% (n = 34,678) of FBC tests for males and females, respectively. Missing data for each parameter are provided in Table 3.

### Colorectal cancer and the FBC

**Figure 2:**
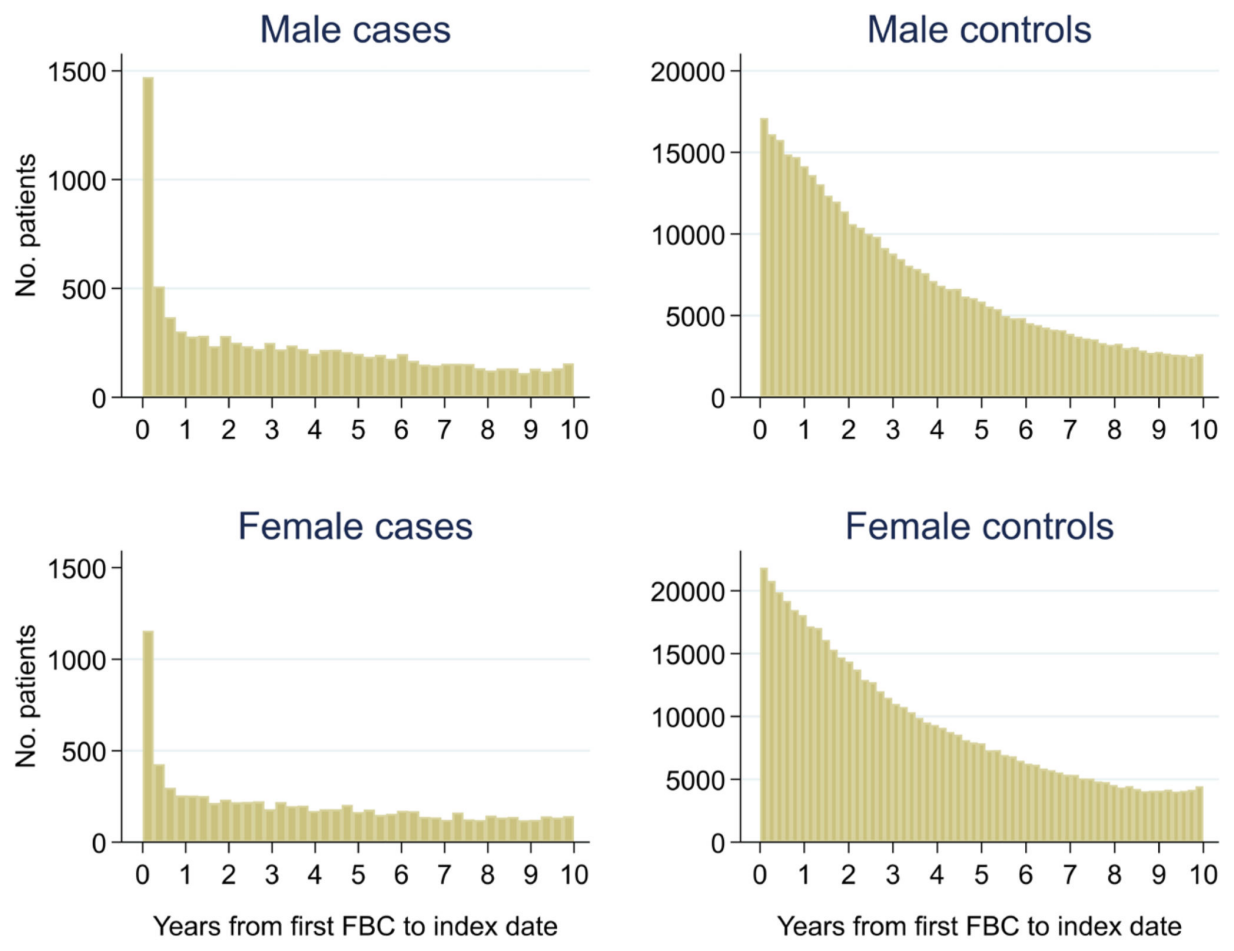
Histogram of follow-up time from first FBC to index date^1,2^. ^1^Index date was the date of diagnosis for cases and a randomly selected date in the patient’s study period for controls. ^2^The spike at time=0 in cases is likely due to patients undergoing cancer investigation. This was not expected to influence trends, as the trends rely on sufficient data at each time-point, not comparability of follow-up.

**Figure 3:**
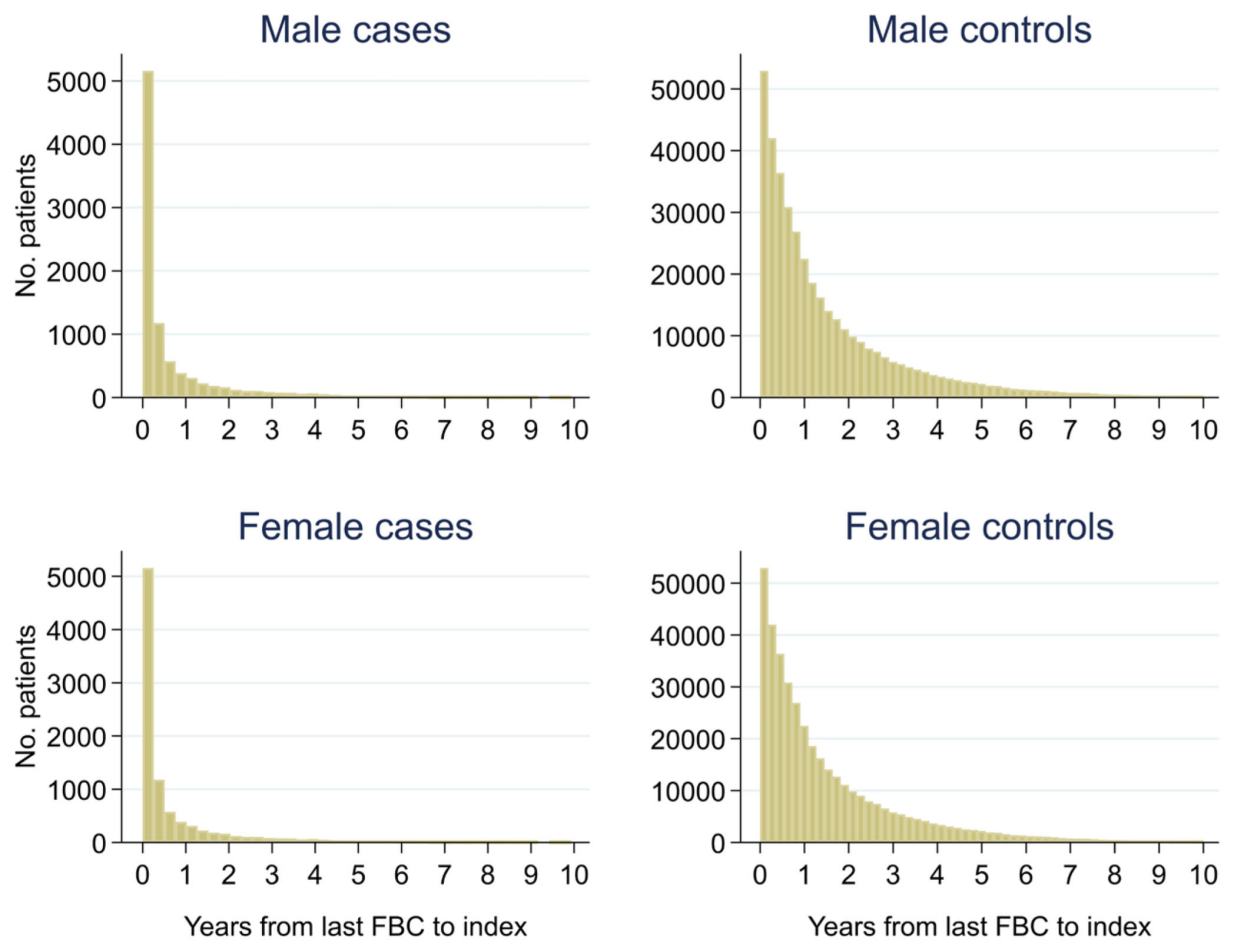
Histogram of time between last FBC and index date^1,2^. ^1^Index date was the date of diagnosis for cases and a randomly selected date in the patient’s study period for controls. ^2^The spike at time=0 in cases is likely due to patients undergoing cancer investigation. This was not expected to influence trends, as the trends rely on sufficient data at each time-point, not comparability of follow-up.

Of 399,405 males, 2.3% (n = 9,255) had a colorectal cancer diagnosis and of 540,544 females, 1.5% (n = 8,153) were diagnosed. Median (min, max) follow-up was 3.0 (0, 10.0) for male cases, 2.7 (0, 10.0) for male controls, 3.3 (0, 10.0) for female cases and 2.8 (0, 10.0) for female controls (Figure 2). Median (min, max) time between the last FBC and index date was 0.2 (0, 9.9) years for male cases, 1.0 (0, 10.0) years for male controls, 0.2 (0, 10.0) years for female cases and 0.9 (0, 10.0) years for female controls (Figure 3).

Mixed models for red blood cell-related parameters are in Table 6 (males) and Table 7 (females), platelet-related in Table 8, and white blood cell count-related in Table 9 (males) and Table 10 (females). The presence of colorectal cancer was statistically significantly associated with all parameter levels (p <0.05 for each model) except white blood cell count and eosinophil count for both males and females.

**Figure 4:**
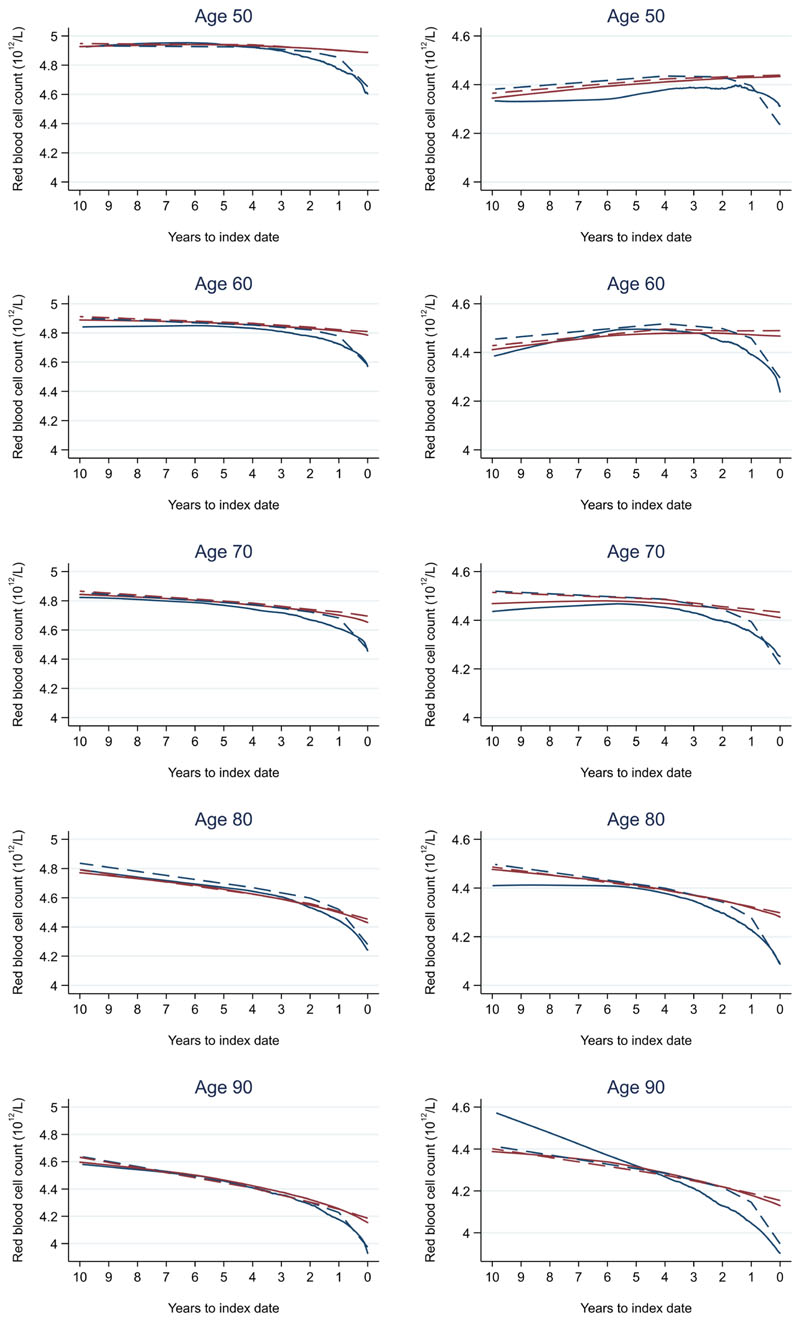
Red blood cell count trends between cases and controls, by age at index date^1,2^. ^1^Index date was the date of diagnosis for cases and a randomly selected date in the patient’s study period for controls. ^2^LOWESS trends are age (+/- 3 years) and modelled (fixed effects) trends are taken at that specific age. Legend: males (left) and females (right). Colorectal cancer (blue line) and no cancer (red line). LOWESS trend (solid line) and modelled trend (dashed line).

Trends in cases and controls for all FBC parameters are in Figure 4–Figure 17. In the raw data (LOWESS curves), there was no apparent difference in trends measured from 10 to four years before index date between cases and controls for both males and females. Within four years before index date, levels changed steadily over time in patients without a diagnosis, such as the reduction in haemoglobin over time that may be due to increasing age. However, cases had trends in FBC levels that diverged from controls for all parameters, except basophil count and eosinophil count. Additionally for cases, our Figures indicate that the rate of change in many FBC levels increased as the time to diagnosis approached. Individualised trends (raw and predicted) are given for four cases (Figure 18) and four controls (Figure 19).

### Colorectal cancer and FBC trends

Using joint modelling, we quantified the relationship between FBC trends and cancer presence (Table 11). The HRs represent how a change in a patient-level trend from the background trend (i.e. trend in patients without cancer) is associated with cancer presence. For example, a decrease in patient-level haemoglobin from the background haemoglobin trend is associated with an increased diagnosis rate (males: 1.783, 95% CI: 1.730, 1.835 and females: 2.037, 95% CI: 1.953, 2.128). There was no statistically significant association between trends in basophil count and eosinophil (both males and females), neutrophil count (males), and white blood cell count (females) and colorectal cancer diagnosis.

### Colorectal cancer and microcytic anaemia

Anaemia was present in 48.8% of male cases and 54.3% of female cases on any FBC within one year of diagnosis. At each six-monthly interval up to five years before index date, the proportion with microcytic anaemia was higher in cases than controls (Figure 20). In cases, the proportion increased as the time to diagnosis approached and was highest at 0–3 months prior to diagnosis: 23.3% (n = 1,188) of 5,107 males and 28.4% (n = 1,286) of 4,521 females with FBCs in that period. The odds of diagnosis (corresponding to microcytic anaemia presence) increased as time to index date approached (Table 12). Presence of microcytic anaemia statistically significantly increased odds of diagnosis at each time band within three years before index date for both males (three-year OR = 2.2 (95% CI = 1.5, 3.1)) and females (three-year OR = 1.7 (95% CI = 1.3, 2.3)). No odds ratio achieved statistical significance at earlier time points.

We compared our graphical trends to microcytic anaemia thresholds. For haemoglobin, trends suggest that the threshold is on average only reached in cases very close to the time of diagnosis, except in the oldest age groups, where the threshold is reached slightly earlier but even controls in this age group reach the threshold. For mean corpuscular volume, trends suggest the threshold is on average not reached, regardless of age group. This suggests only a minority of patients have iron-deficiency determined from the FBC test (maximum 23.3% males and 28.4% females, Figure 20).

### Colorectal cancer and FBC reference ranges

For all FBC parameters, the graphical trends showed that levels remained in the reference range for both cases and controls, except red blood cell count, haemoglobin, haematocrit, mean corpuscular volume, and mean platelet volume. In these five parameters, the trends suggest blood levels often only reach abnormal thresholds within approximately six months of diagnosis in younger cases. However, in older cases, levels are abnormal for approximately three years before diagnosis, which was also observed for older controls.

### Tumour staging and the FBC

The number of cases diagnosed per Duke’s tumour stage is in Table 13. Mixed models including Duke’s stage at diagnosis, developed using cases alone, are provided for red blood cell-related parameters in 14 (males) and 15 (males), plate-let-related in Table 16, and white blood cell-related in 17 (males) and 18 (females). In the raw data (LOWESS curves), there appeared to be no difference in trends over time between Stage A and Stage D tumours among older patients. However, changes started up to one year earlier in patients with Stage D in younger patients (see Figure 21 for haemoglobin and Figure 22 for platelets – for the remaining parameters, please see ‘Data availability’). This was observed in all FBC parameters except mean platelet volume, basophil count, eosinophil count, and lymphocyte count for both males and females, which showed no apparent difference between tumour stages.

### Sensitivity analysis

A matched design gave 54,198 males (9,033 cases, 45,165 controls) and 48,918 females (8,153 cases, 40,765 controls). For cases and controls separately, mean (SD) age at index was 70.8 (10.6) for males and 73.0 (11.8) for females and median (min-max) follow-up time from first FBC to index was 3.1 (0–10) years for males and 3.3 (0–10) years for females.

There was no difference in graphical trends when using a matched design (see Figure 23 for haemoglobin and Figure 24 for platelets – for the remaining parameters, please see ‘Data availability’). Additionally, coefficients from mixed effects models changed only slightly, with 86.4% and 86.3% of coefficients changing by only <0.1 for males and females respectively – for the models, please see ‘Data availability’.

## Discussion

### Summary

We identified age- and sex-adjusted trends in many FBC parameters that differed between patients with and without a diagnosis within approximately four years before diagnosis. Differences in cases grew larger in the run up to diagnosis, with levels in patients without a diagnosis changing less rapidly over time. Joint models showed a statistically significant association between trends in many FBC parameters and colorectal cancer diagnosis. Trends were also different between cases and controls before the onset of FBC abnormalities or referral thresholds, as these thresholds were only reached close to diagnosis.

Summary statistics indicate an imbalance in age at index and follow-up time between cases and controls. Our sensitivity analysis showed no apparent differences between matched and unmatched designs. This was expected a priori, because in our unmatched design, graphical trends are already reported by age and sex separately. Additionally, (sex-stratified) mixed effects models included age, accounting for the imbalance. Further-more, length of follow-up, despite imbalanced (Figure 2), did not influence the trends, as there were many cases and controls with tests available at each time-point, increasing the quality/precision of the trend.

### Comparison with existing literature

Two prior studies assessed changes over time in haemoglobin between patients with and without colorectal cancer: one in an Israeli population^11^ and one in a combined Swedish and Danish population^12^. We report similar findings in a UK population, with haemoglobin levels that diverge around four years before diagnosis and a greater decline in the run up to diagnosis. We also report changes over time for many other FBC parameters. Another study used machine-learning methods to develop an algorithm called the ColonFlag, which assesses change over time in various parameters (at 18 and 36 months before index FBC) from a single patient to derive a monotone score for diagnosis from 0–100 in an Israeli population (EarlySign)^13^. It is unclear what trends are considered related to colorectal cancer. A fourth study used logistic regression to test whether the difference between the two most recent FBCs was associated with diagnosis for five parameters in UK primary care data^14^. With the two tests performed at any time in a mean follow-up period of 6.3 years, they report no association in change in red blood cell count (p = 0.13), white blood cell count (p = 0.06), or haematocrit (p = 0.23) but do report an association for change in mean corpuscular volume (p = 0.04) and mean corpuscular haemoglobin (p = 0.02). However, our study suggests that red blood cell count, white blood cell count, and haematocrit do change over time due to colorectal cancer.

A recent study of haemoglobin levels in newly diagnosed colorectal cancer patients (mean age approximately 70 years) in Finland reported lower haemoglobin levels at the time of diagnosing higher stage tumours (13.2 g/dL at Stage 1 and 12.2 g/dL at Stage 4)^15^. These levels are similar to those identified in this study (Figure 21), where the trends in most FBC parameters were similar between Stage A and D colorectal cancers, but the divergence from controls started up to one year earlier in Stage D diagnoses compared to Stage A. This divergence in patients diagnosed with Stage A colorectal cancer often occurred within one year prior to diagnosis, suggesting a relatively short time window for detection between the earliest and latest stage.

When compared to NICE and WHO guidelines for anaemia, which could be due to any reason including iron-deficiency, our trends indicated haemoglobin levels often reached the threshold for males (<13 g/dL) and females (<12 g/dL) (NICE: Suspected cancer recognition and referral, NICE: Anaemia - iron deficiency, WHO: anaemia) at approximately 6–12 months before diagnosis. Up to one year before diagnosis, anaemia was present in 48.8% of male cases and 54.3% of female cases. These results are similar to those reported in a previous UK primary care study of anaemia within one year prior to colorectal cancer diagnosis^16^. Microcytic anaemia, commonly caused by iron-deficiency, which may warrant further investigation for colorectal cancer, was present in 23% of male cases and 28% of female cases within a year prior to diagnosis. Our study suggests there are relevant changes that occur up to three years before the presence of anaemia, including iron-deficiency anaemia, and these changes could be more helpful to facilitate early detection than relying on low haemoglobin levels.

We also compared the FBC results to normal reference ranges (Oxford University Hospitals NHS UK). Abnormal FBC parameter levels are considered to represent health-related conditions or disease. Although differences in most FBC parameters between cases and controls grew larger as the time to diagnosis approached, they remained small overall and often remained in the normal reference range, except for red blood cell count, haemoglobin, haematocrit, mean corpuscular volume, and mean platelet volume. These parameters often only became abnormal close to diagnosis in younger cases but for many years in older cases, which was also observed in older controls. Therefore, such differences in trends between cases and controls may not be obvious to a clinician in general practice, as these differences would be considered to represent little-to-no concern, and the opportunity to utilise these changes over time to identify colorectal cancer would be missed. Our study supports the conclusions of another recent report that highlighted how the normal range does not necessarily reflect a healthy individual^17^.

### Limitations

FBC blood tests are ordered for many reasons in primary care, not colorectal cancer specifically, but these reasons are not recorded in CPRD. Patients with FBCs are suspected to be generally less well than patients who are not tested (except in antenatal screening, which includes a FBC), so controls included in this study may not be entirely healthy. However, many existing studies identified in our recent systematic review have shown that in patients with FBCs, the test has potential to distinguish between patients with and without colorectal cancer^4^.

Red blood cell distribution width is a parameter used to diagnose medical conditions, especially colorectal cancer (Medline: Red cell distribution width). Historically, this parameter has not been reported to primary care practices until relatively recently, hence why almost all tests in our data have this value missing. Consequently, we excluded this relevant parameter from all analyses.

**Figure 5:**
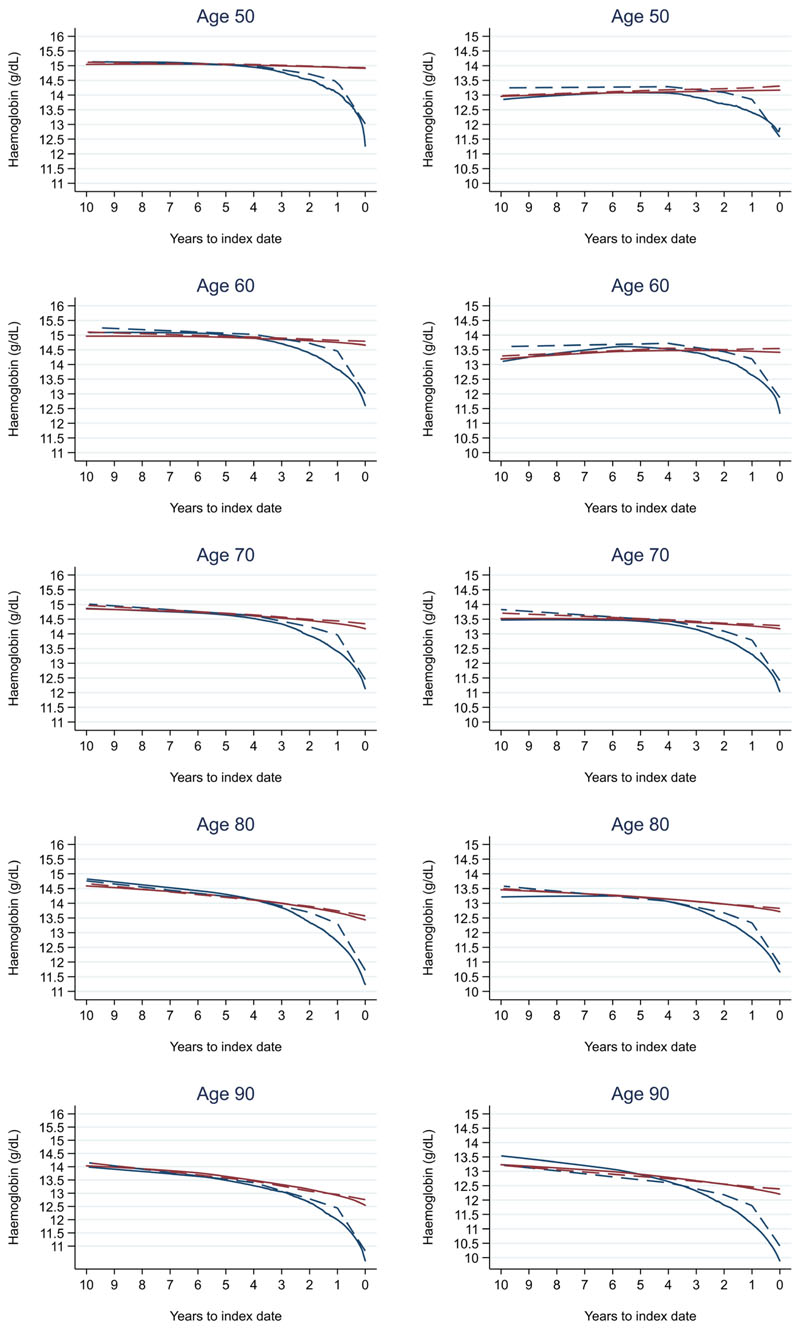
Haemoglobin trends between cases and controls, by age at index date^1,2^. ^1^Index date was the date of diagnosis for cases and a randomly selected date in the patient's study period for controls. ^2^LOWESS trends are age (+/- 3 years) and modelled (fixed effects) trends are taken at that specific age. Legend: males (left) and females (right). Colorectal cancer (blue line) and no cancer (red line). LOWESS trend (solid line) and modelled trend (dashed line).

**Figure 17:**
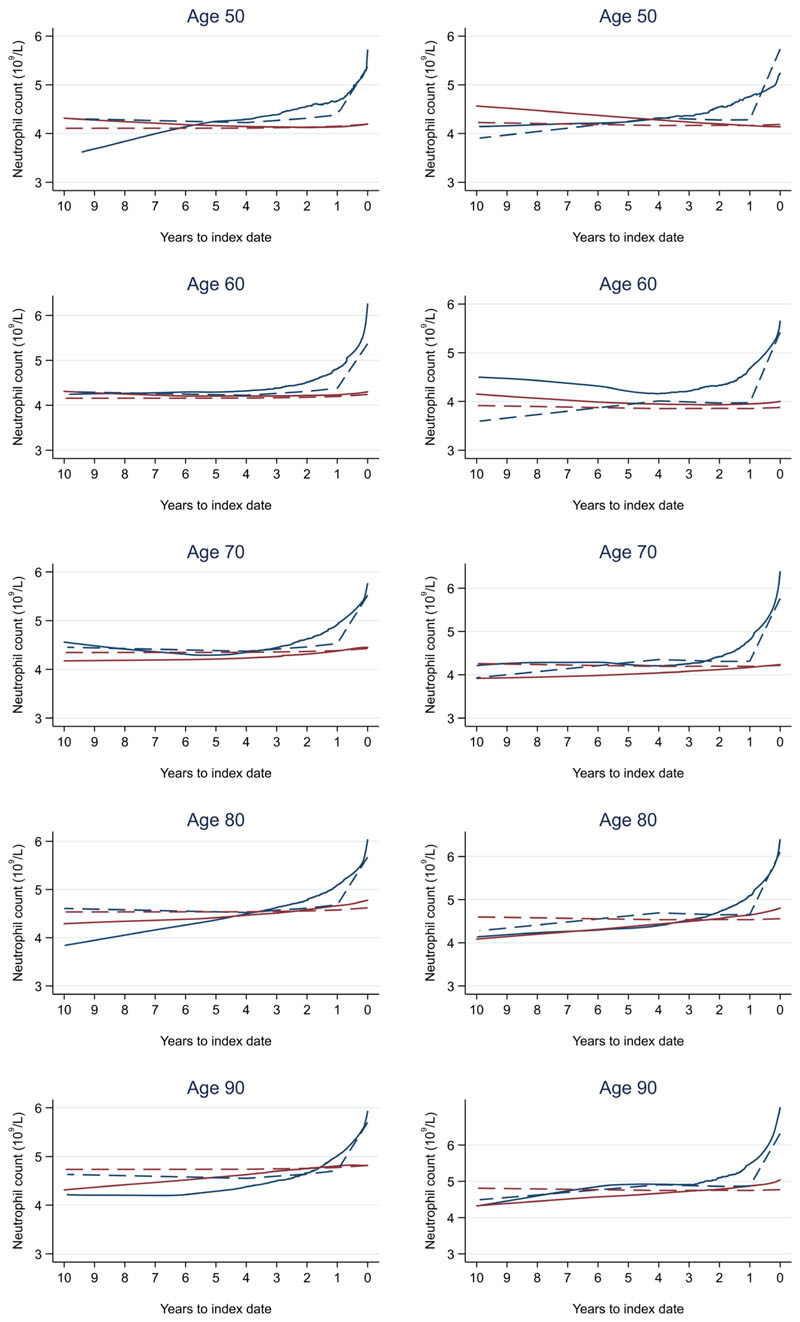
Neutrophil count trends between cases and controls, by age at index date^1,2^. ^1^Index date was the date of diagnosis for cases and a randomly selected date in the patient’s study period for controls. ^2^LOWESS trends are age (+/- 3 years) and modelled (fixed effects) trends are taken at that specific age. Legend: males (left) and females (right). Colorectal cancer (blue line) and no cancer (red line). LOWESS trend (solid line) and modelled trend (dashed line).

**Figure 18:**
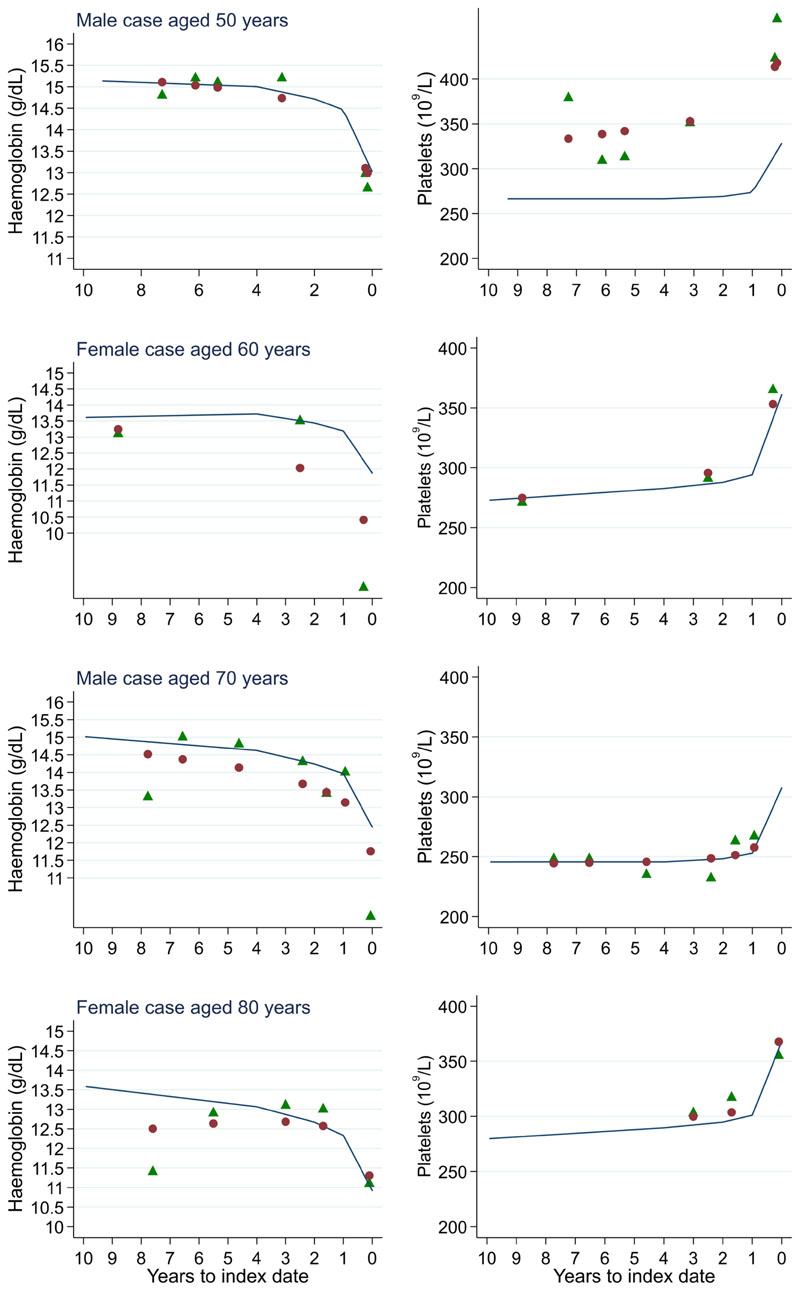
Individualised haemoglobin (left) and platelet (right) trends for four cases by age at index^1^. ^1^Index date was the date of diagnosis for cases and a randomly selected date in the patient’s study period for controls. Legend: raw data (green triangle), modelled population-level trend (fixed effects) (blue line), modelled individualised patient-level trend (fixed and random effects) (red circle).

**Figure 19:**
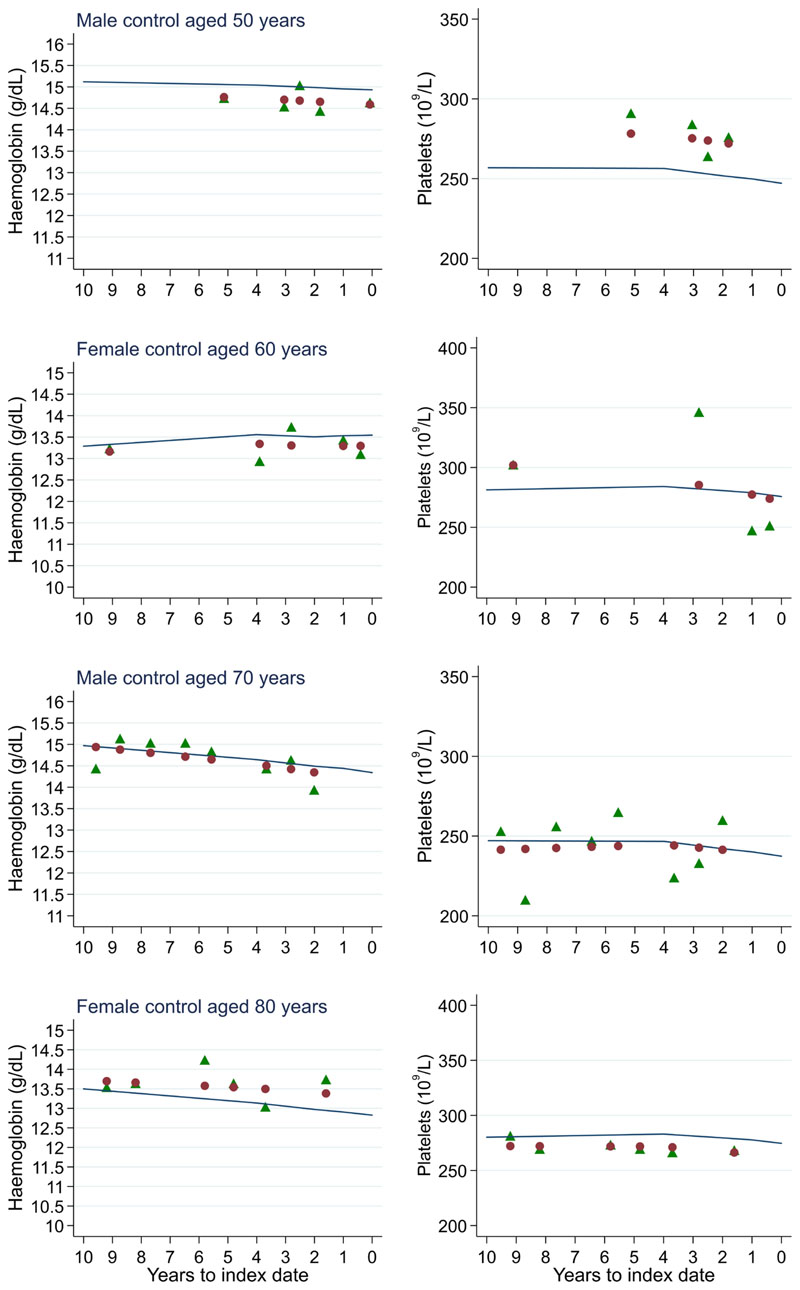
Individualised haemoglobin (left) and platelet (right) trends for four controls by age at index^1^. ^1^Index date was the date of diagnosis for cases and a randomly selected date in the patient’s study period for controls. Legend: raw data (green triangle), modelled population-level trend (fixed effects) (blue line), modelled individualised patient-level trend (fixed and random effects) (red circle).

**Figure 20:**
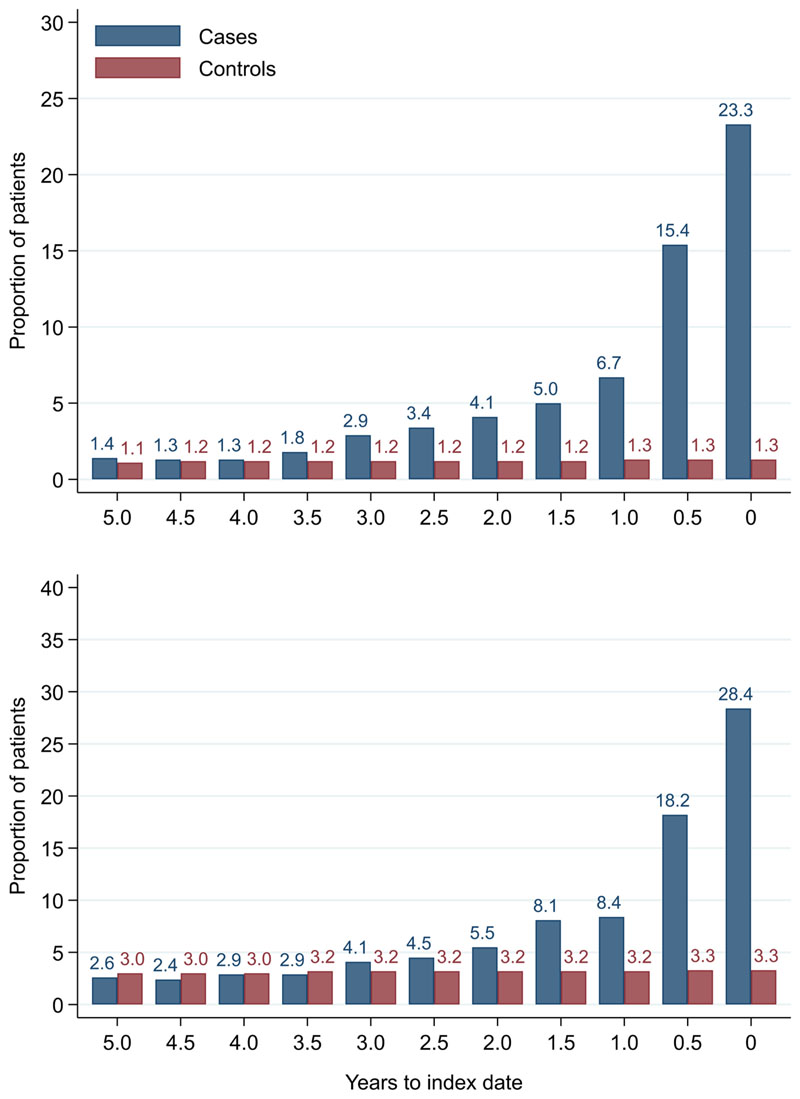
Microcytic anaemia prevalence over six-monthly (+/- 3 months) time band. Legend: males (top) and females (bottom).

**Figure 21:**
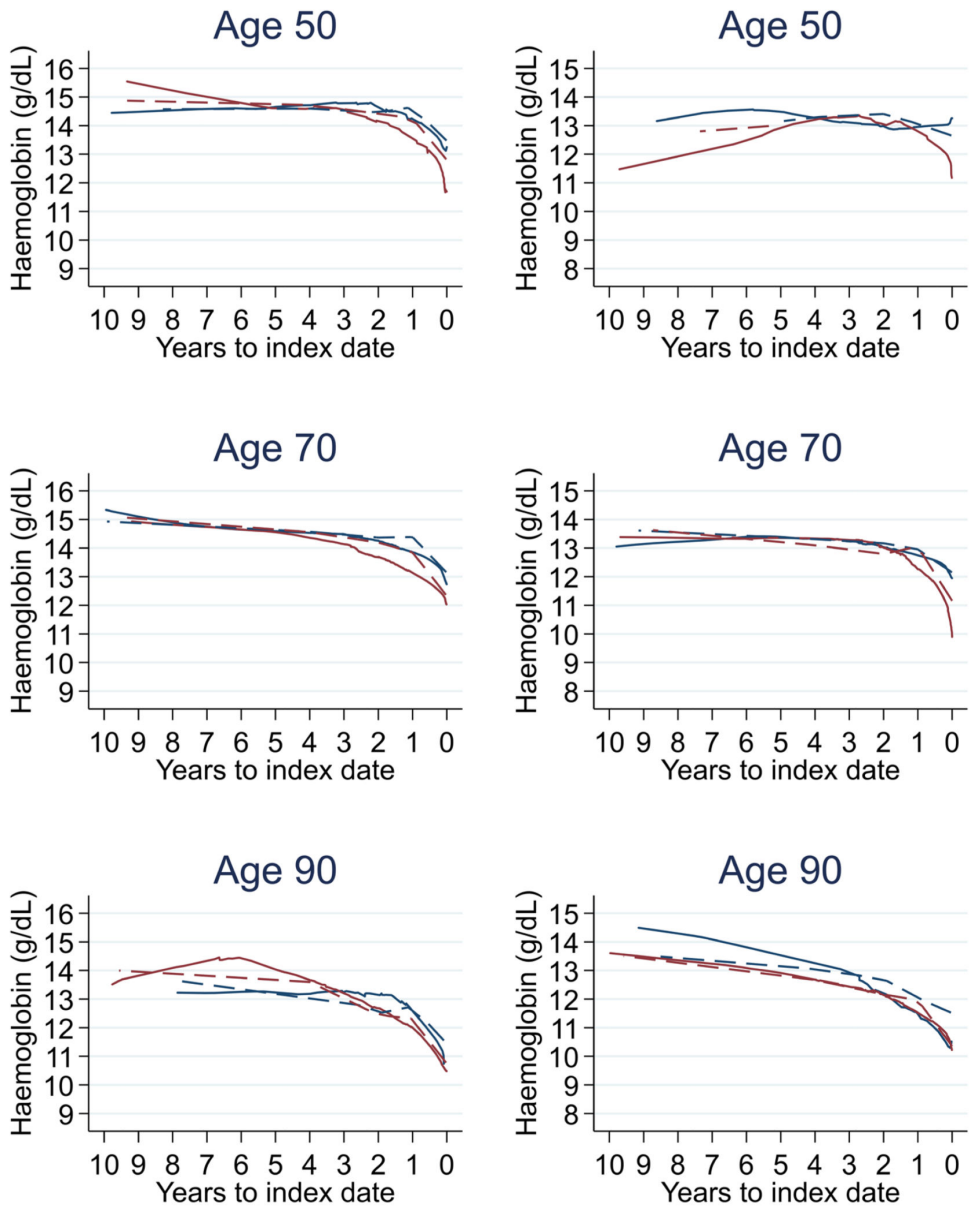
Haemoglobin trends between Duke’s A and D tumour stage by age at index date^1,2^. ^1^Index date was the date of diagnosis for cases and a randomly selected date in the patient’s study period for controls. ^2^LOWESS trends are age (+/- 3 years) and modelled (fixed effects) trends are taken at that specific age. Legend: males (left) and females (right). Stage A (blue line) and Stage D (red line). LOWESS trend (solid line) and modelled trend (dashed line).

**Figure 22:**
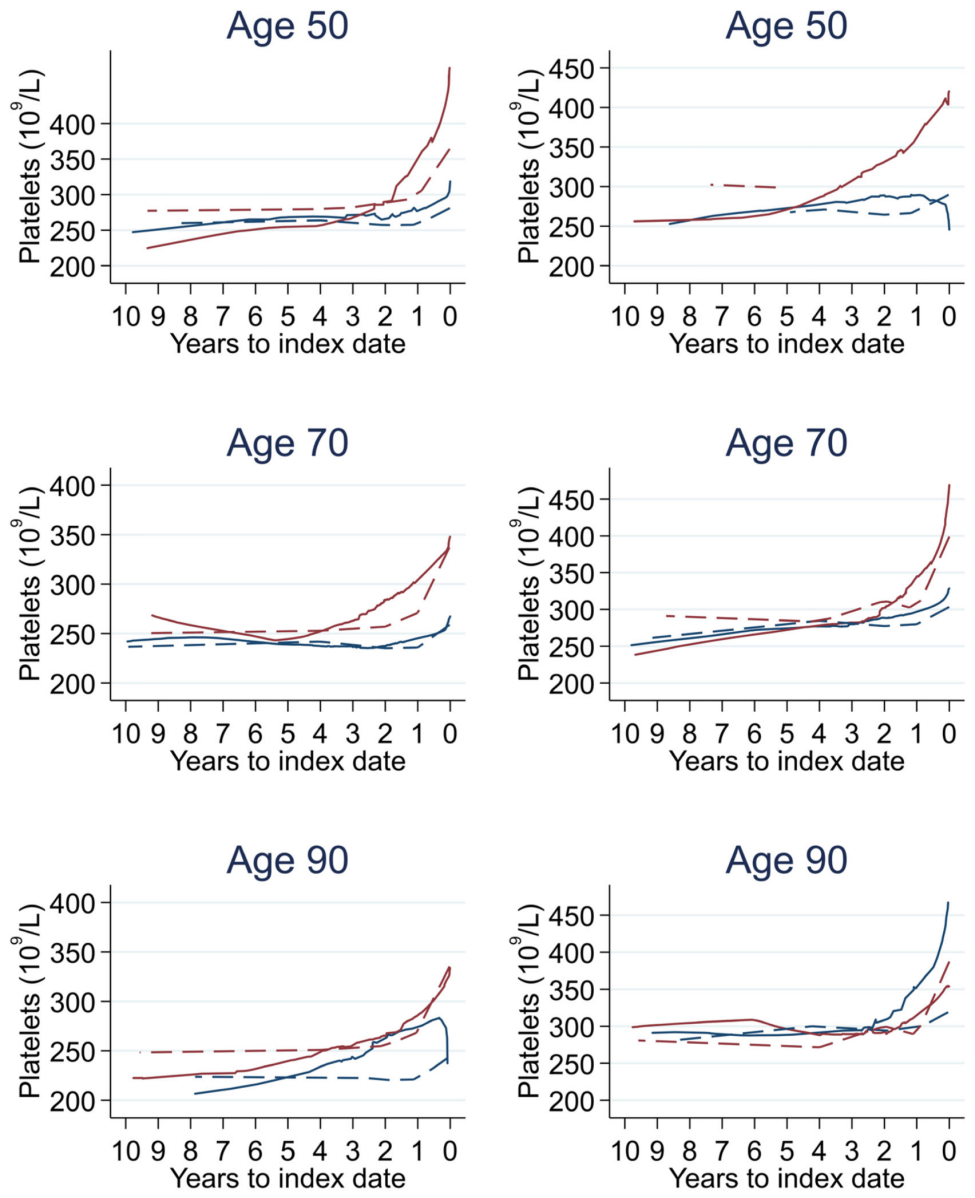
Platelet trends between Duke’s A and D tumour stage by age at index date^1,2^. ^1^Index date was the date of diagnosis for cases and a randomly selected date in the patient’s study period for controls. ^2^LOWESS trends are age (+/- 3 years) and modelled (fixed effects) trends are taken at that specific age. Legend: males (left) and females (right). Stage A (blue line) and Stage D (red line). LOWESS trend (solid line) and modelled trend (dashed line).

**Figure 23:**
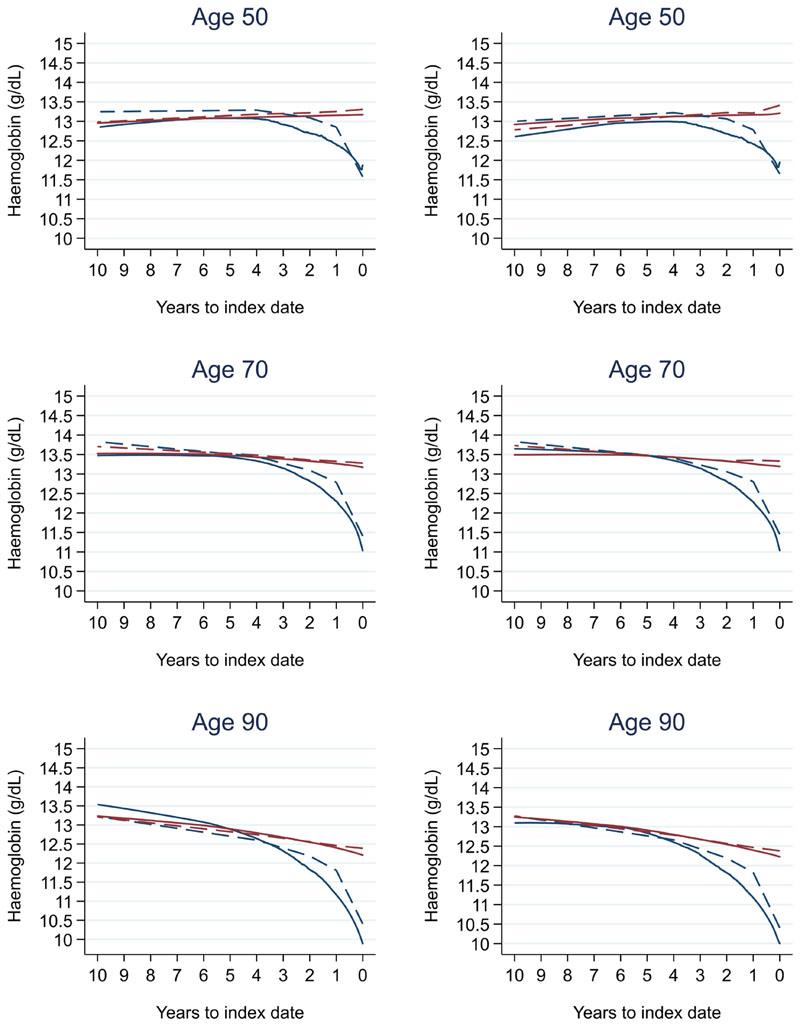
Haemoglobin trends for females by age at index^1,2^: unmatched (left) and matched (right). ^1^Index date was the date of diagnosis for cases and a randomly selected date in the patient’s study period for controls. ^2^LOWESS trends are age (+/- 3 years) and modelled (fixed effects) trends are taken at that specific age. Legend: colorectal cancer (blue line) and no cancer (red line). LOWESS trend (solid line) and modelled trend (dashed line).

**Figure 24:**
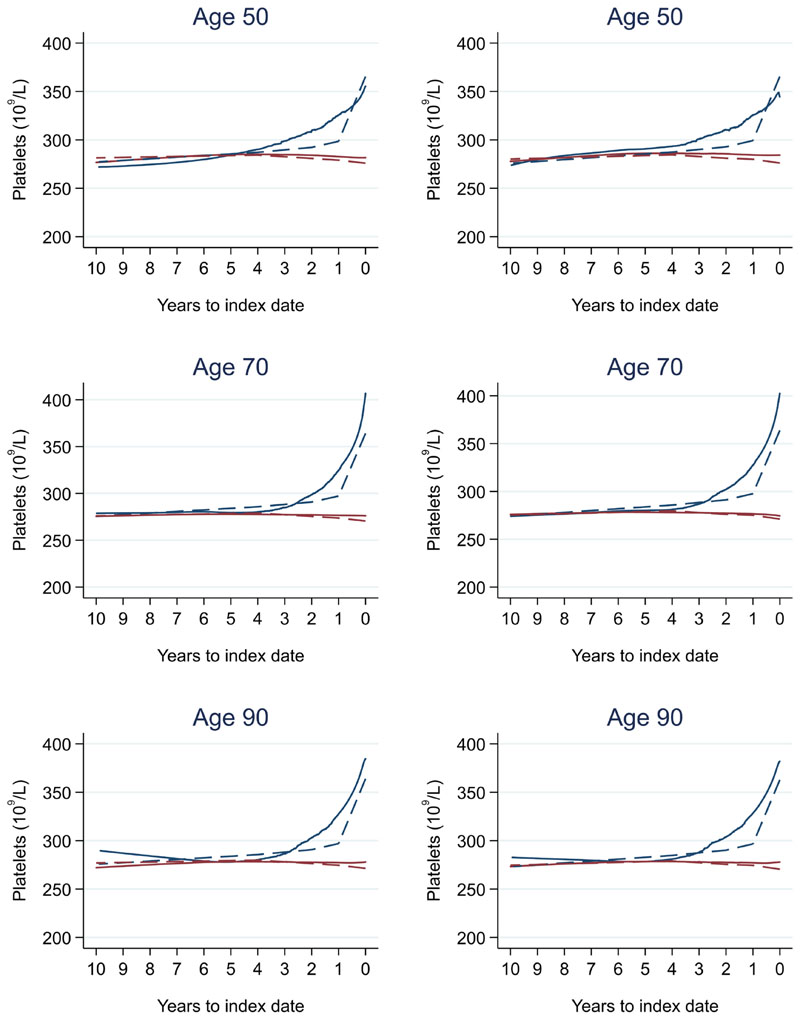
Platelets trends for females by age at index^1,2^: unmatched (left) and matched (right). ^1^Index date was the date of diagnosis for cases and a randomly selected date in the patient’s study period for controls. ^2^LOWESS trends are age (+/- 3 years) and modelled (fixed effects) trends are taken at that specific age. Legend: colorectal cancer (blue line) and no cancer (red line). LOWESS trend (solid line) and modelled trend (dashed line).

Due the nature of the case-control study design, we modelled 10 years of longitudinal data before index for each age group separately. Therefore, the trends in older age groups for many FBC parameters were subject to the ‘survivor effect’. For example, in Figure 5 (trends in haemoglobin), controls who survived to 90 years at index are likely healthier 10 years earlier than controls who survived to 80 years at index, who may or may not have survived to age 90. Thus, older patients would often have FBC levels that reflected healthier individuals 10 years earlier on average than patients diagnosed/censored at that younger age. This ‘survivor effect’ could not be adjusted for in our analyses.

Other known factors influencing colorectal cancer risk, such as ethnicity and family history, and FBC confounders, such as comorbidity status, were not available so were not included in our models. Many relevant FBC confounders, such as diet, vitamin use, and sleeping patterns, are not recorded in electronic health records. Nonetheless, age and sex are key characteristics to adjust trends. Adjustments for additional factors may be considered in future work.

Tumour stage at diagnosis was missing for approximately one-third of colorectal cancer diagnoses. Therefore, many cases were excluded from analyses of tumour staging, reducing sample size and precision of estimates.

### Implications for practice

The differences between cases and controls in the trends over time identified in this study would often go unnoticed in routine practice. It is difficult for busy clinicians filing results to notice minor changes in parameter values over time. Therefore, we are developing a dynamic statistical prediction model^18,19^ that makes use of trends in the FBC to derive an individual’s risk of diagnosis in the future. To develop the model, we will first determine the predictive value of patient-level trends – in this study, we only report that relevant trends exist and may be of help. The prediction model will aim to support the current colorectal cancer UK screening programme by identifying possible cases for investigation (NHS: Bowel cancer screening). It will utilise trends in multiple FBC parameters over time to detect colorectal cancer at earlier time points than is possible using single parameter thresholds. For example, high-risk individuals (without alarming symptoms) could be offered a faecal immunochemical test (FIT) test (to identify any rectal bleeding), which is slowly making its way for use outside the screening programme in patients with low-risk symptoms. FIT testing is cheaper and has less burden on patients and healthcare services than colonoscopy. Therefore, patients identified through our models who subsequently have a positive FIT result could then be referred for further testing under the fast-track system.

## Conclusions

Many FBC results change due to the presence of colorectal cancer. We identified differences in trends over a five-year period before diagnosis that differed to trends in patients without colorectal cancer. Such trends may pre-date single-value thresholds for referral for cancer investigation and blood-abnormality. They may therefore facilitate earlier detection, improving the likelihood of successful treatment and improved survival rates.

## Figures and Tables

**Figure 6:**
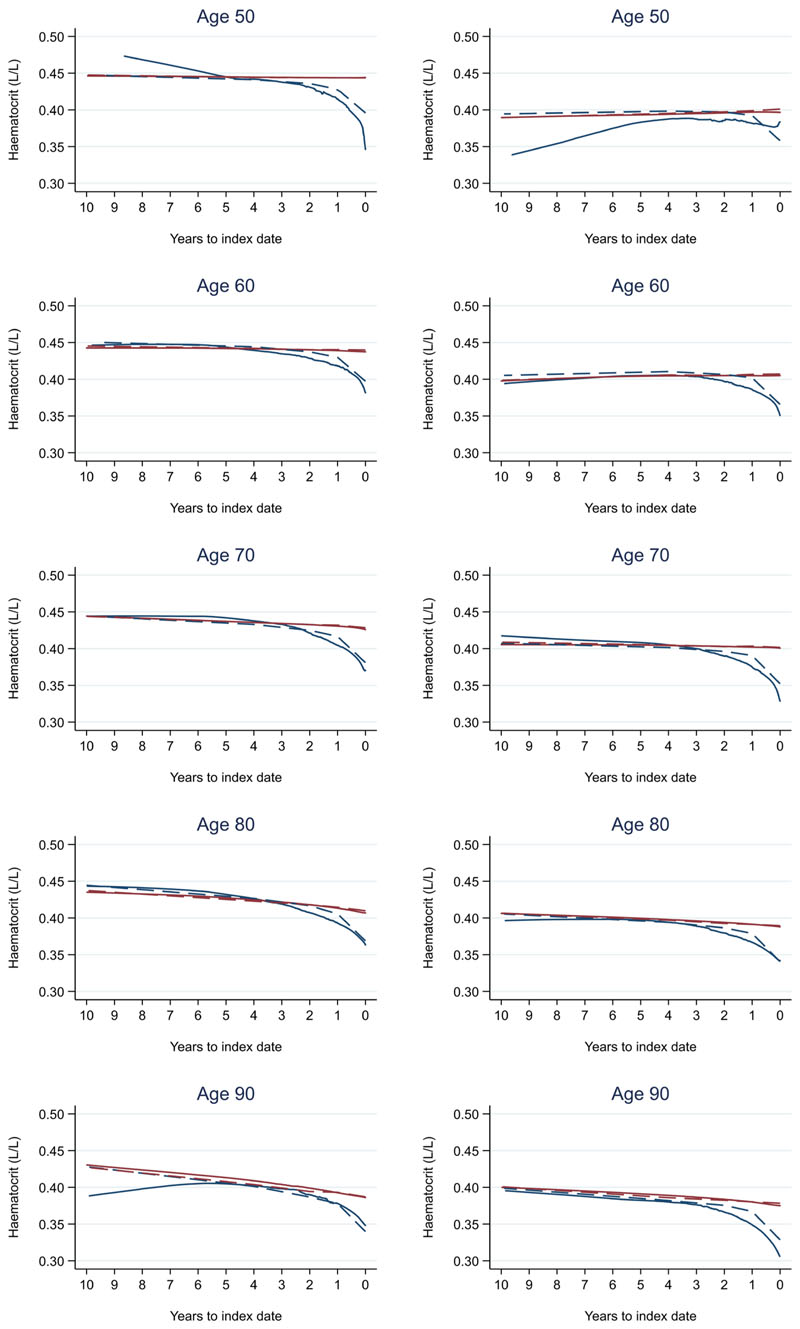
Haematocrit trends between cases and controls, by age at index date^1,2^. ^1^Index date was the date of diagnosis for cases and a randomly selected date in the patient's study period for controls. ^2^LOWESS trends are age (+/- 3 years) and modelled (fixed effects) trends are taken at that specific age. Legend: males (left) and females (right). Colorectal cancer (blue line) and no cancer (red line). LOWESS trend (solid line) and modelled trend (dashed line).

**Figure 7:**
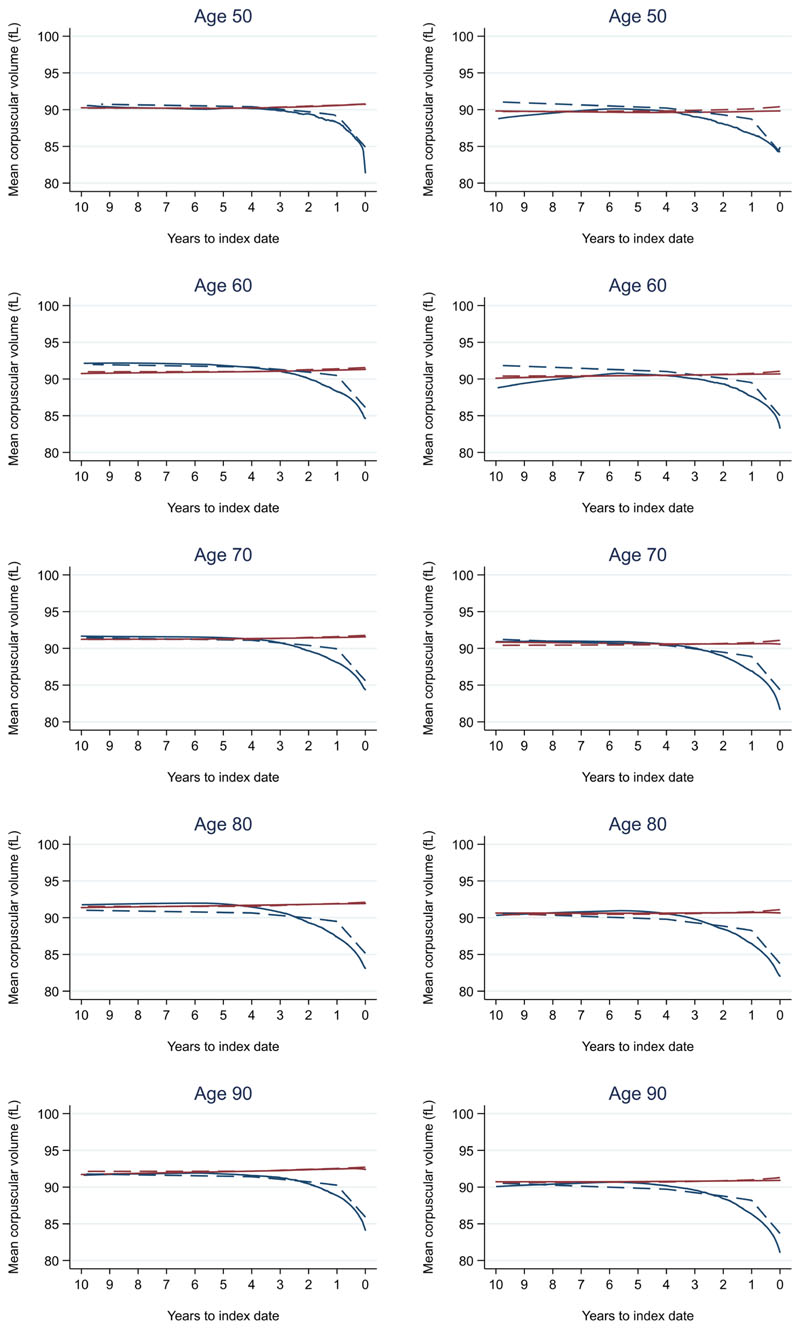
Mean corpuscular volume trends between cases and controls, by age at index date^1,2^. ^1^Index date was the date of diagnosis for cases and a randomly selected date in the patient’s study period for controls. ^2^LOWESS trends are age (+/- 3 years) and modelled (fixed effects) trends are taken at that specific age. Legend: males (left) and females (right). Colorectal cancer (blue line) and no cancer (red line). LOWESS trend (solid line) and modelled trend (dashed line).

**Figure 8:**
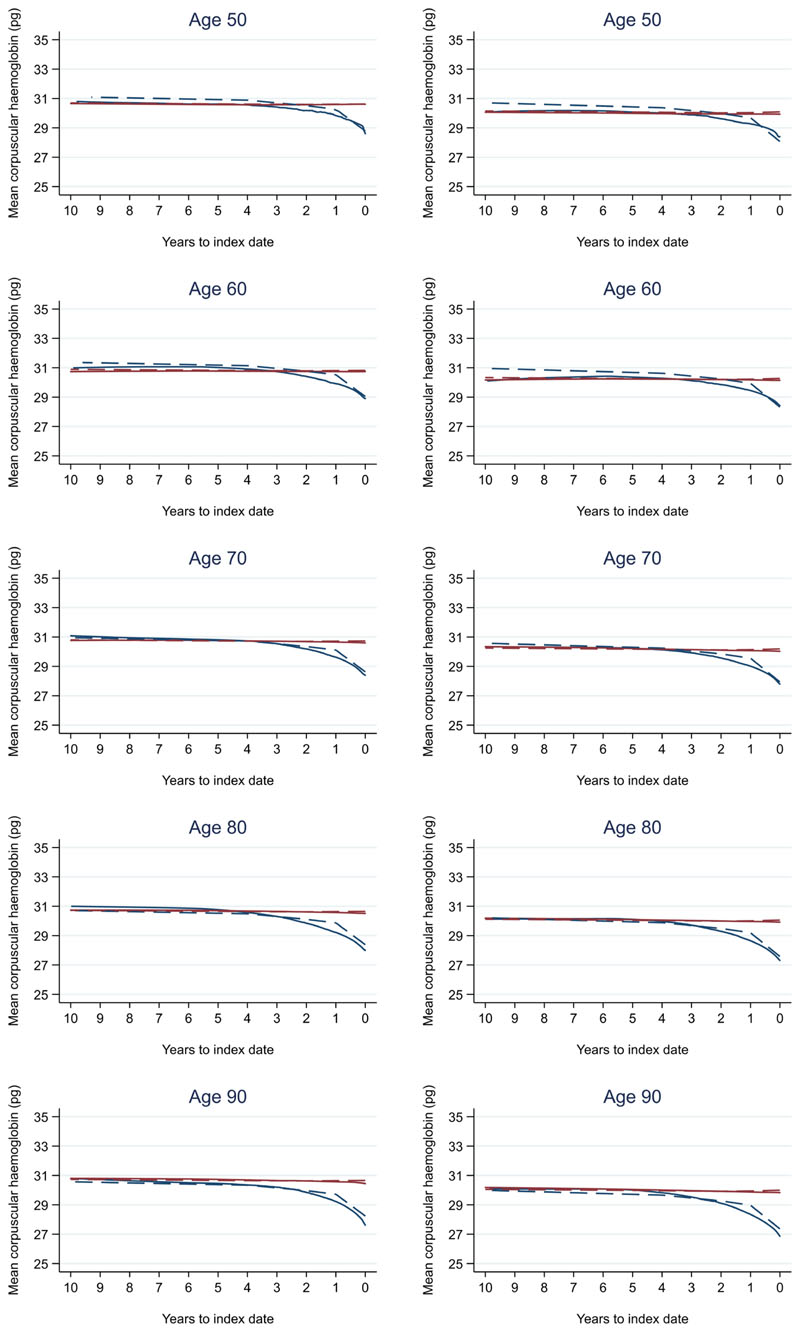
Mean corpuscular haemoglobin trends between cases and controls, by age at index date^1,2^. ^1^Index date was the date of diagnosis for cases and a randomly selected date in the patient’s study period for controls. ^2^LOWESS trends are age (+/- 3 years) and modelled (fixed effects) trends are taken at that specific age. Legend: males (left) and females (right). Colorectal cancer (blue line) and no cancer (red line). LOWESS trend (solid line) and modelled trend (dashed line).

**Figure 9:**
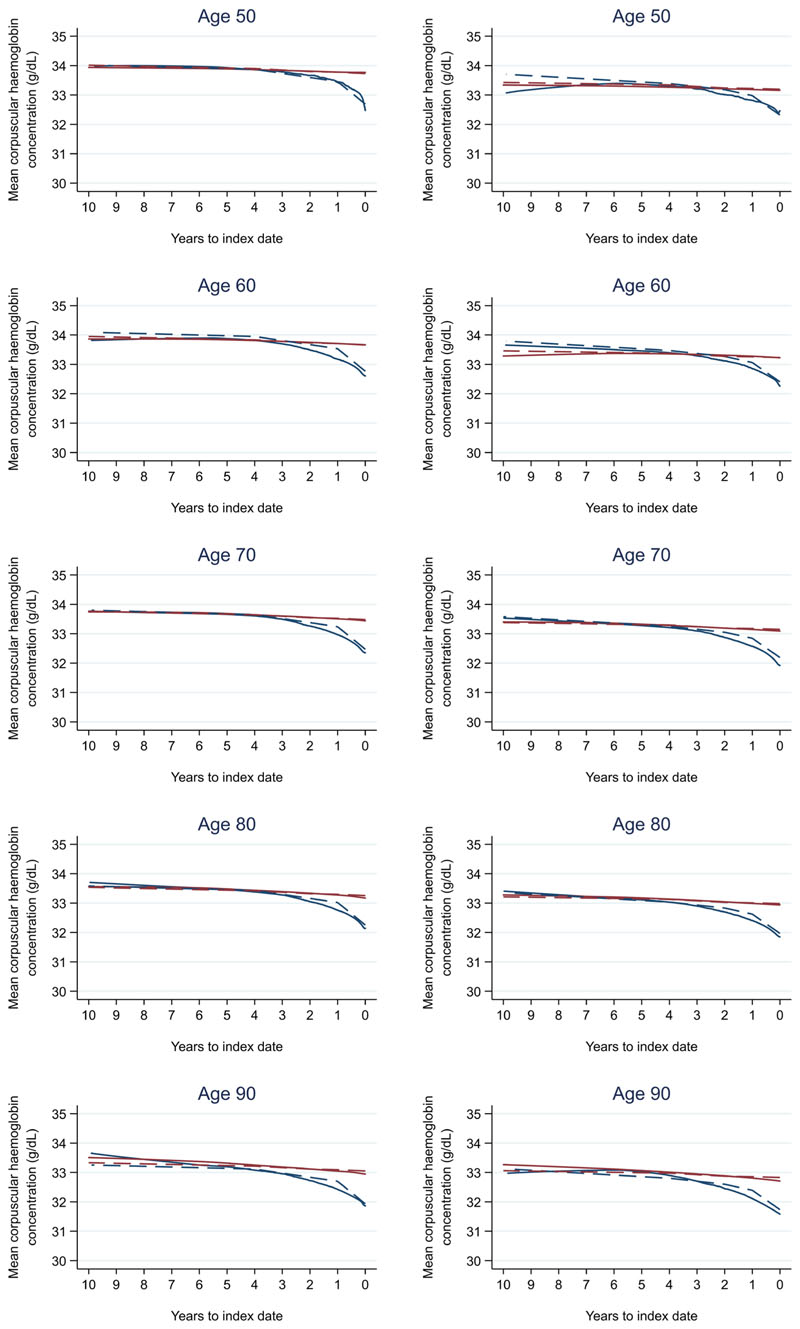
Mean corpuscular haemoglobin concentration trends between cases and controls, by age at index date^1,2^. ^1^Index date was the date of diagnosis for cases and a randomly selected date in the patient’s study period for controls. ^2^LOWESS trends are age (+/- 3 years) and modelled (fixed effects) trends are taken at that specific age. Legend: males (left) and females (right). Colorectal cancer (blue line) and no cancer (red line). LOWESS trend (solid line) and modelled trend (dashed line).

**Figure 10:**
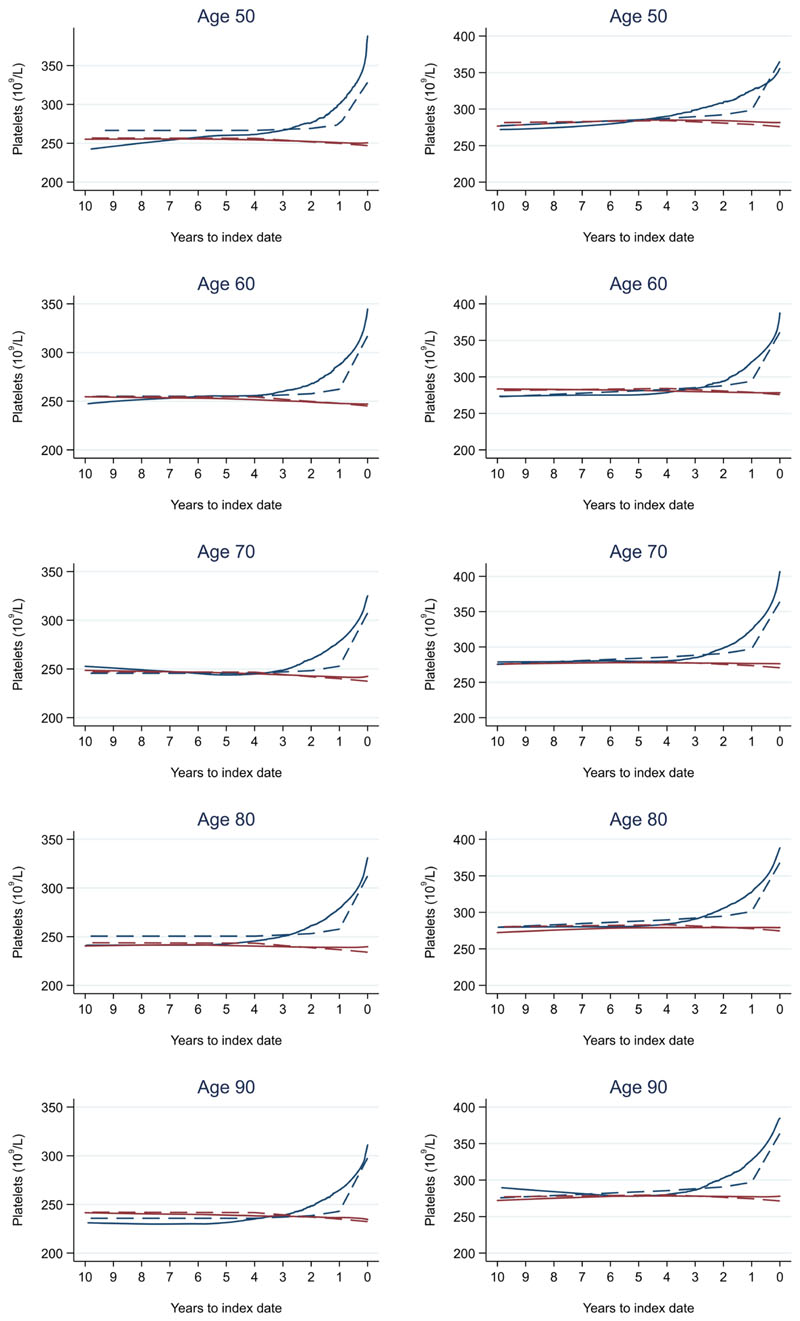
Platelets trends between cases and controls, by age at index date^1,2^. ^1^Index date was the date of diagnosis for cases and a randomly selected date in the patient’s study period for controls. ^2^LOWESS trends are age (+/- 3 years) and modelled (fixed effects) trends are taken at that specific age. Legend: males (left) and females (right). Colorectal cancer (blue line) and no cancer (red line). LOWESS trend (solid line) and modelled trend (dashed line).

**Figure 11:**
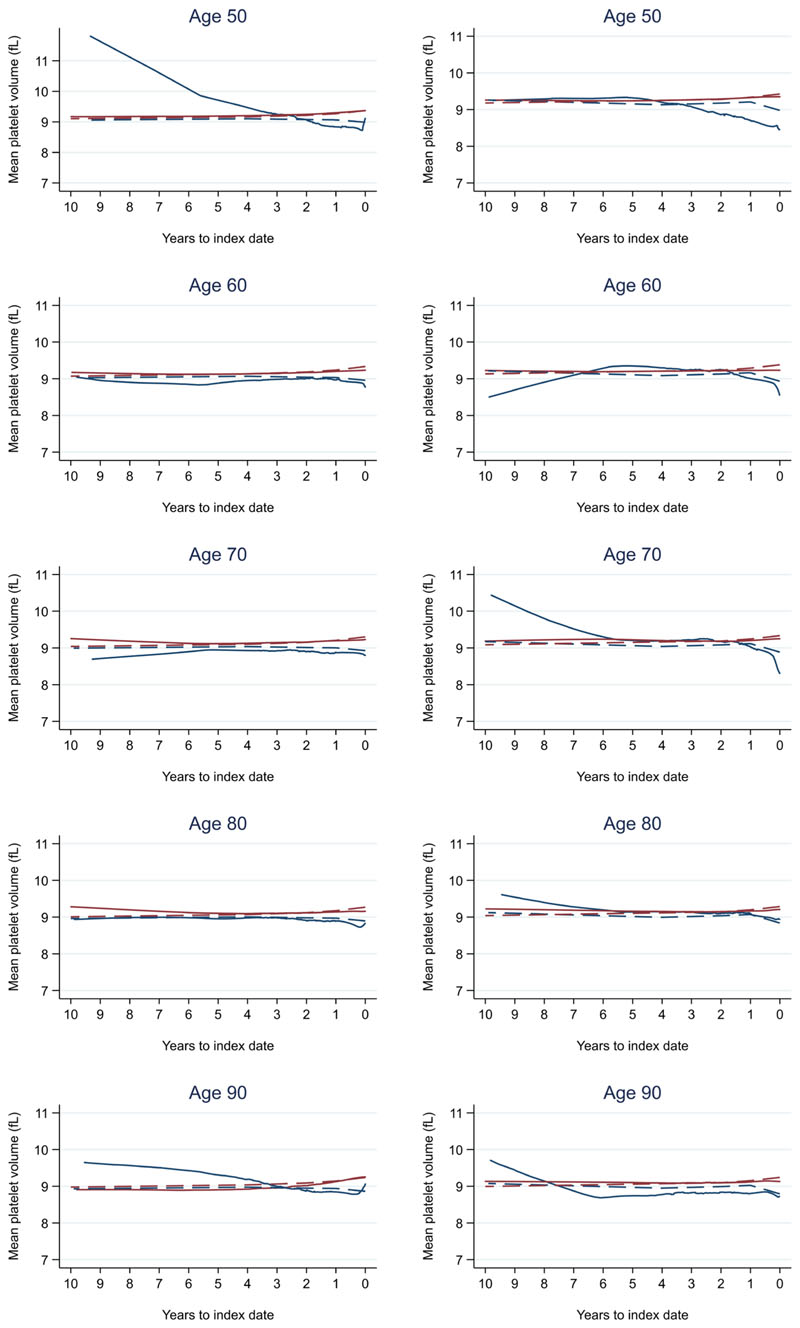
Mean platelet volume trends between cases and controls, by age at index date^1,2^. ^1^Index date was the date of diagnosis for cases and a randomly selected date in the patient's study period for controls. ^2^LOWESS trends are age (+/- 3 years) and modelled (fixed effects) trends are taken at that specific age. Legend: males (left) and females (right). Colorectal cancer (blue line) and no cancer (red line). LOWESS trend (solid line) and modelled trend (dashed line).

**Figure 12:**
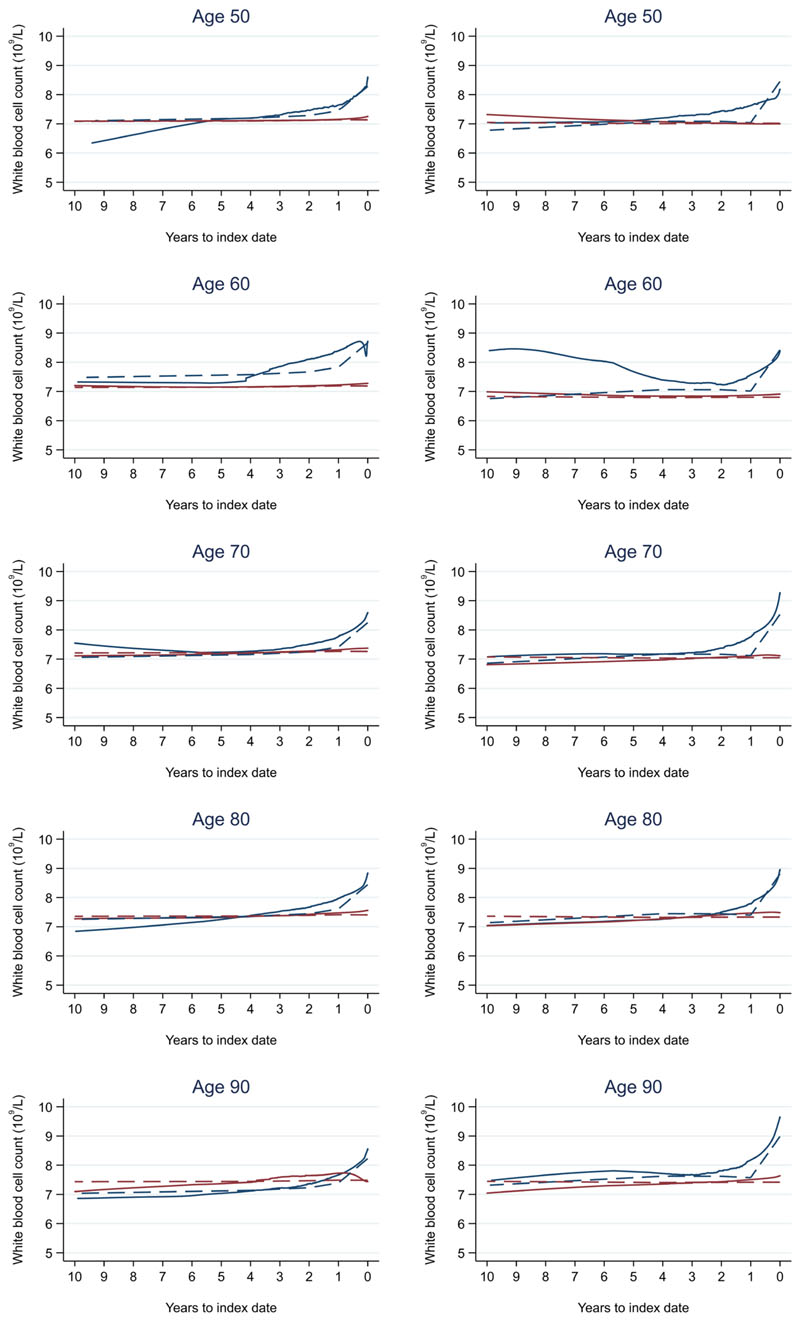
White blood cell count trends between cases and controls, by age at index date^1,2^. ^1^Index date was the date of diagnosis for cases and a randomly selected date in the patient's study period for controls. ^2^LOWESS trends are age (+/- 3 years) and modelled (fixed effects) trends are taken at that specific age. Legend: males (left) and females (right). Colorectal cancer (blue line) and no cancer (red line). LOWESS trend (solid line) and modelled trend (dashed line).

**Figure 13:**
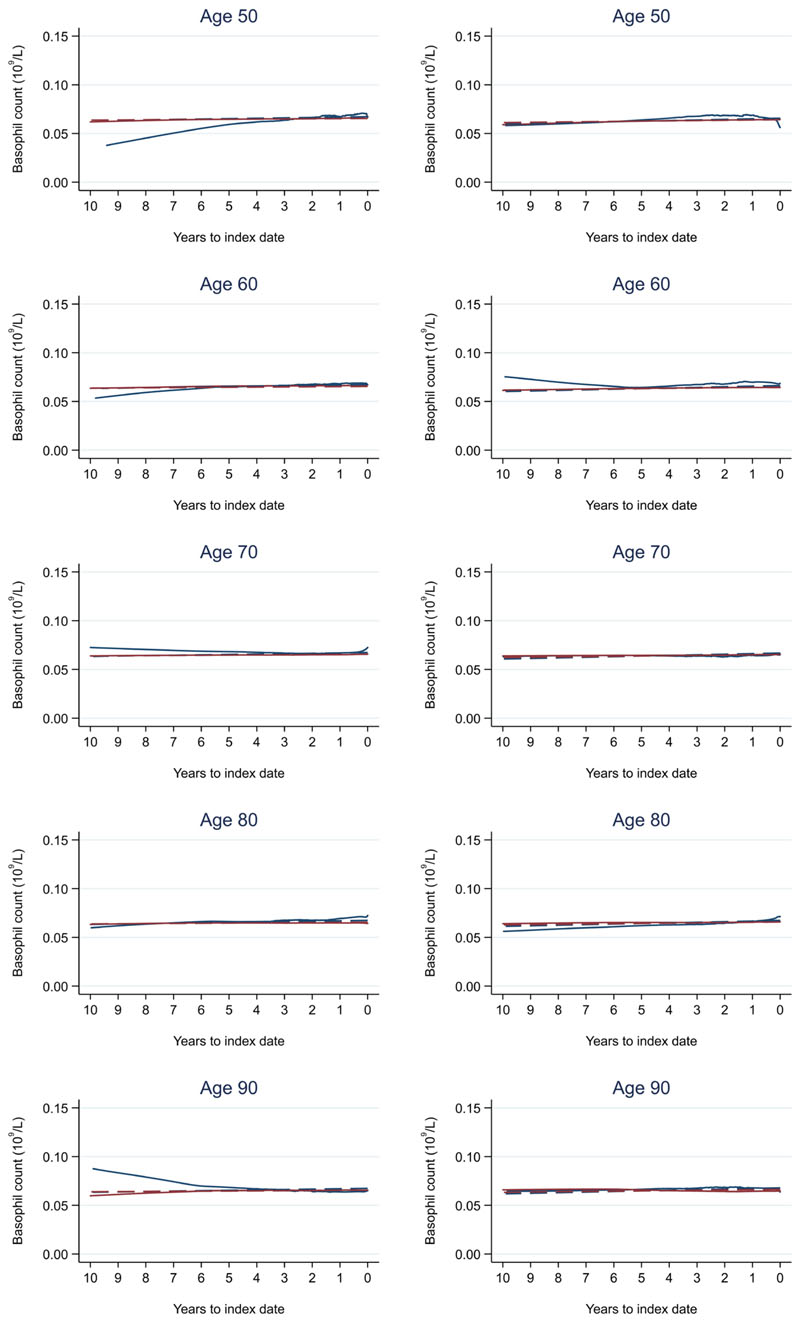
Basophil count trends between cases and controls, by age at index date^1,2^. ^1^Index date was the date of diagnosis for cases and a randomly selected date in the patient’s study period for controls. ^2^LOWESS trends are age (+/- 3 years) and modelled (fixed effects) trends are taken at that specific age. Legend: males (left) and females (right). Colorectal cancer (blue line) and no cancer (red line). LOWESS trend (solid line) and modelled trend (dashed line).

**Figure 14:**
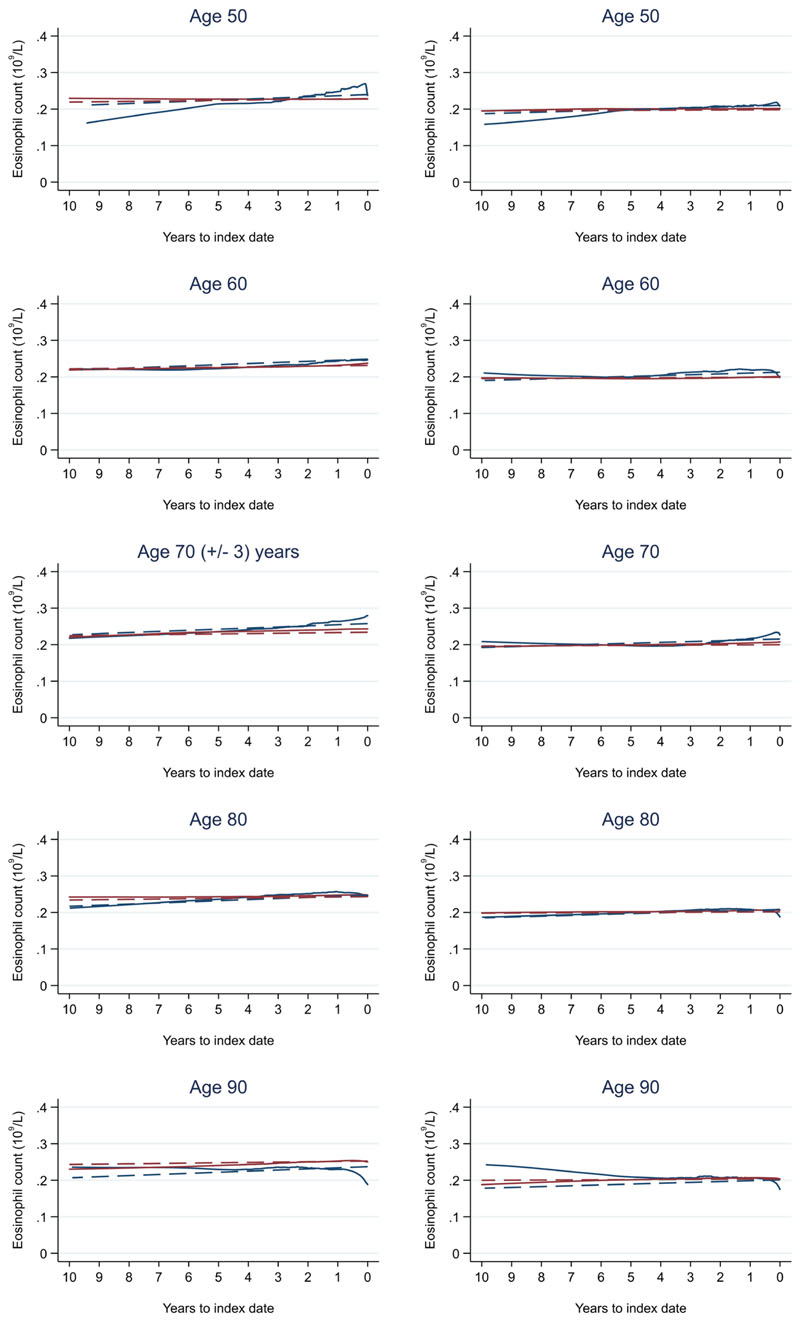
Eosinophil count trends between cases and controls, by age at index date^1,2^. ^1^Index date was the date of diagnosis for cases and a randomly selected date in the patient’s study period for controls. ^2^LOWESS trends are age (+/- 3 years) and modelled (fixed effects) trends are taken at that specific age. Legend: males (left) and females (right). Colorectal cancer (blue line) and no cancer (red line). LOWESS trend (solid line) and modelled trend (dashed line).

**Figure 15:**
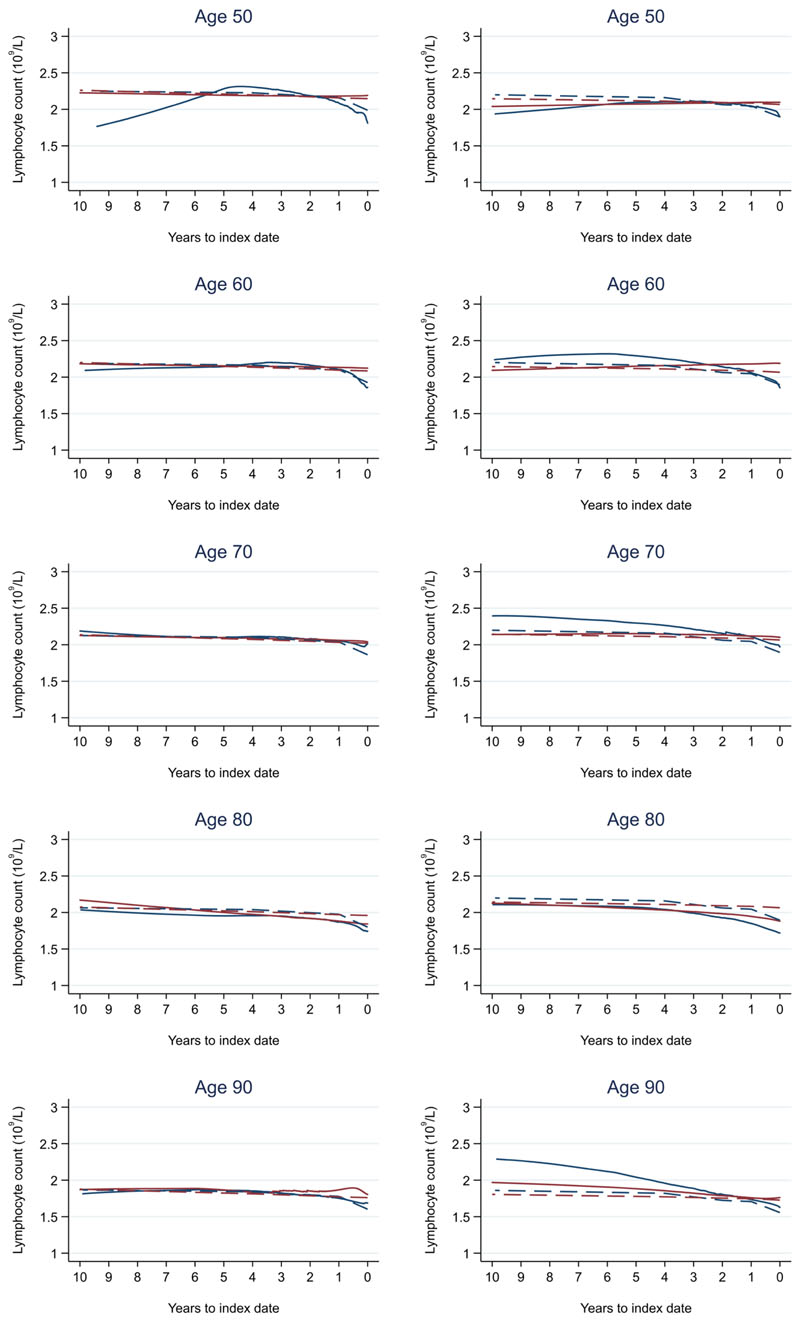
Lymphocyte trends between cases and controls, by age at index date^1,2^. ^1^Index date was the date of diagnosis for cases and a randomly selected date in the patient’s study period for controls. ^2^LOWESS trends are age (+/- 3 years) and modelled (fixed effects) trends are taken at that specific age. Legend: males (left) and females (right). Colorectal cancer (blue line) and no cancer (red line). LOWESS trend (solid line) and modelled trend (dashed line).

**Figure 16:**
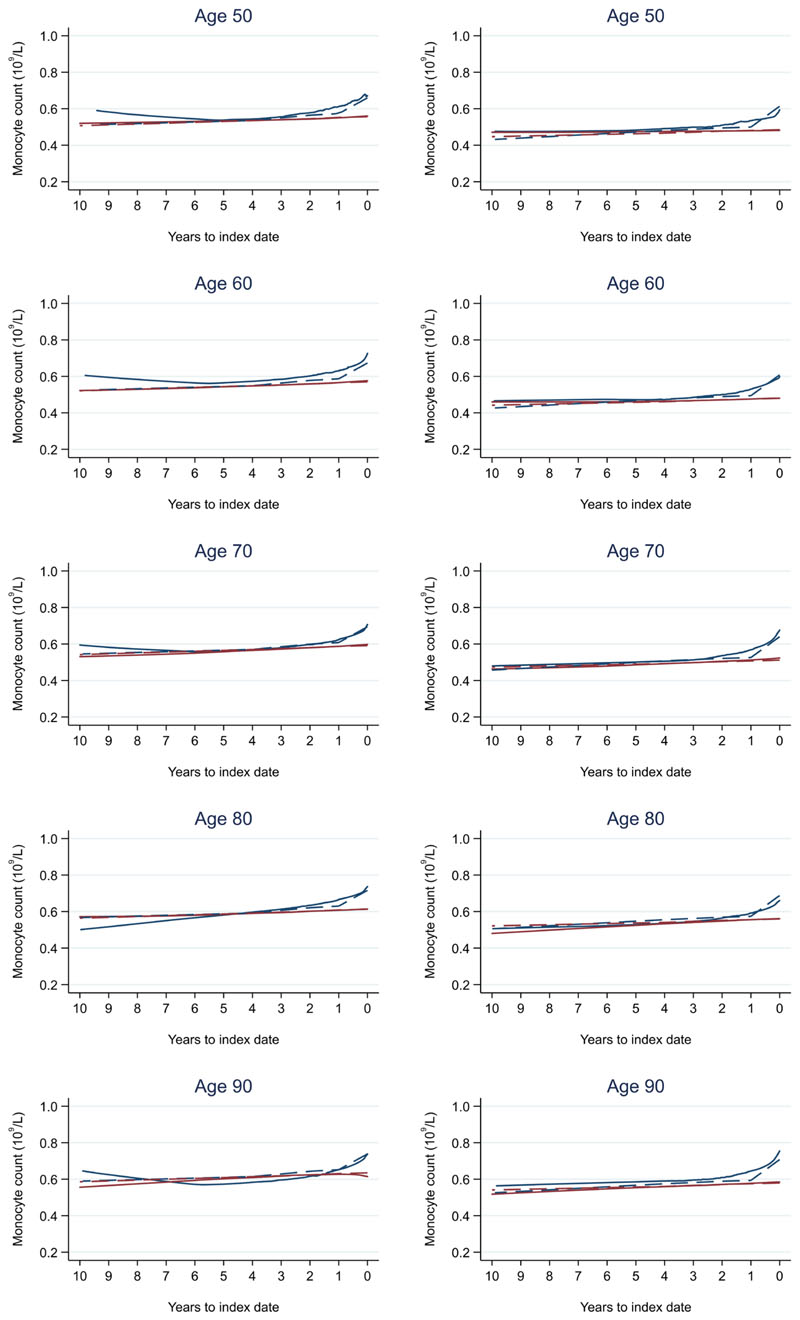
Monocyte count trends between cases and controls, by age at index date^1,2^. ^1^Index date was the date of diagnosis for cases and a randomly selected date in the patient’s study period for controls. ^2^LOWESS trends are age (+/- 3 years) and modelled (fixed effects) trends are taken at that specific age. Legend: males (left) and females (right). Colorectal cancer (blue line) and no cancer (red line). LOWESS trend (solid line) and modelled trend (dashed line).

**Table 1:** Clinical codes relating to the full blood count from CPRD.

FBC parameter	Entity code	Medical code
Red blood cell count	194	17, 13788, 26931, 26932, 26933, 44213, 50182, 57136, 58853, 70079
White blood cell count	207	15, 1955, 3372, 4760, 4996, 13817, 13818, 18516, 22293, 26325, 26946, 26947, 26948, 38198, 45115, 48015, 48341, 53865, 92372
Haemoglobin	173	4, 739, 795, 3942, 10404, 13755, 26272, 26908, 26909, 26910, 26912, 26913, 33284, 35749, 39601, 41531
Haematocrit/packed cell volume	312	40, 99, 14240, 19836, 23476, 27143, 27144, 27145, 41478, 55365, 62347,
Mean corpuscular volume	182	10, 2480, 13774, 26920, 26921, 26922, 41160, 52874,
Mean corpuscular haemoglobin	180	20, 23214, 26917, 26918, 40170, 47174, 49225, 51616, 61951,
Mean corpuscular haemoglobin concentration	181	30, 26919, 39202, 47345, 55183, 64474, 72488
Red blood cell distribution width	361	64, 1191, 1962, 9933, 19837, 51484, 52050
Platelets	189	7, 3320, 4006, 4415, 26926, 26927, 37666
Mean platelet volume	-	14166
Basophil count	313	25, 27146, 27147, 27148, 53404
Eosinophil count	168	22, 13742, 18531, 26905, 26906
Lymphocyte count	208	19, 3189, 11240, 23120, 23121, 26949, 26950, 32932, 34551, 37677, 42346, 74019
Monocyte count	183	21, 9248, 13776, 26923, 26924, 26925, 44189, 72849
Neutrophil count	184	18, 4463, 13777, 15725, 23112, 23113, 31382, 105211,

**Table 2:** Clinical codes relating to colorectal cancer from NCRAS.

ICD-10 code	Term
C18	Malignant neoplasm of colon
C18.0	Caecum (incl.: Ileocaecal valve)
C18.1	Appendix
C18.2	Ascending colon
C18.3	Hepatic flexure
C18.4	Transverse colon
C18.5	Splenic flexure
C18.6	Descending colon
C18.7	Sigmoid colon (Incl.: Sigmoid (flexure); Excl.: rectosigmoid junction (C19))
C18.8	Overlapping lesion of colon
C18.9	Colon, unspecified (Incl.: Large intestine NOS)
C19	Malignant neoplasm of rectosigmoid junction (Incl.: Colon with rectum, Rectosigmoid (colon))
C20	Malignant neoplasm of rectum (Incl.: Rectal ampulla)
C21	Malignant neoplasm of anus and anal canal
C21.0	Anus, unspecified (Excl.: anal: margin (C43.5, C44.5), skin (C43.5, C44.5) perianal skin (C43.5, C44.5))
C21.1	Anal canal (Incl.: Anal sphincter)
C21.2	Cloacogenic zone
C21.8	Overlapping lesion of rectum, anus and anal canal (Incl.: Anorectal junction, Anorectum, and Malignant neoplasm of rectum, anus and anal canal whose point of origin cannot be classified to any one of the categories C20-C21.2)

**Table 3:** Details of the full blood count blood test parameters included in the analysis.

Full blood count parameter	Biologically plausible range	Number (%) missing^1^	
		Males (n=1,193,619 FBCs)	Females (n=1,874,609 FBCs)
Red blood cell count (10^12^/L)	2.5 – 7.5	103,607 (8.7%)	165,608 (8.8%)
Haemoglobin (g/dL)	0.3 – 21.0	22,637 (1.9%)	34,678 (1.8%)
Haematocrit/packed cell volume (L/L)	0.1 – 0.6	968,246 (81.1%)	1,514,243 (80.8%)
Mean corpuscular volume (fL)	53 – 125	57,807 (4.8%)	86,606 (4.6%)
Mean corpuscular haemoglobin (pg)	22 – 36	92,460 (7.7%)	150,566 (8.0%)
Mean corpuscular haemoglobin concentration (g/dL)	27 – 37	165,983 (13.9%)	262,094 (14.0%)
Red cell distribution width (%)	9 – 15	1,193,587 (100%)	1,874,530 (100%)
Platelet count (10^9^/L)	0 – 1500	71,385 (6.0%)	114,575 (6.1%)
Mean platelet volume (fL)	5 – 14	1,040,399 (87.2%)	1,630,832 (87.0%)
White blood cell count (10^9^/L)	0 – 850	223,059 (18.7%)	351,858 (18.8%)
Basophil count (10^9^/L)	0 – 1.5	326,914 (27.4%)	535,356 (28.6%)
Eosinophil count (10^9^/L)	0 – 8	322,907 (27.1%)	529,122 (28.2%)
Lymphocyte count (10^9^/L)	0 – 850	289,640 (24.3%)	457,255 (24.4%)
Monocyte count (10^9^/L)	0 – 40	298,940 (25.0%)	473,324 (25.2%)
Neutrophil count (10^9^/L)	0 – 850	285,027 (23.9%)	451,291 (24.1%)

1 This is missing data that remained after applying known mathematical equations that can be used to derive many components.

**Table 4:** Normal reference range for patients aged 13+ years for each FBC parameter.

FBC parameter	Males normal range	Females normal range
Red blood cell count (10^9^/L)	4.5–5.5	3.8–4.8
Haemoglobin (g/dL)	13–17	12–15
Haematocrit (L/L)	0.40–0.50	0.36–0.46
Mean corpuscular volume (fL)	83–101	83–101
Mean corpuscular haemoglobin (pg)	27–32	27–32
Mean corpuscular haemoglobin concentration (g/dL)	31.5–34.5	31.5–34.5
Platelets (10^12^/L)	150–400	150–400
Mean platelet volume (fL)	9.0–12.1	9.0–12.1
White blood cell count (10^12^/L)	4–11	4–11
Basophil count (10^12^/L)	0.02–0.10	0.02–0.10
Eosinophil count (10^12^/L)	0.02–0.50	0.02–0.50
Lymphocyte count (10^12^/L)	1–4	1–4
Monocyte count (10^12^/L)	0.2–1.0	0.2–1.0
Neutrophil count (10^12^/L)	2–7	2–7

**Table 5:** Characteristics of the patient sample.

	Diagnosis of colorectal cancer			No diagnosis of colorectal cancer		
	Number	Mean age^1^ (SD)	Age^1^ range	Number	Mean age^1^ (SD)	Age^1^ range
Male	9,255	70.8 (10.6)	40 – 101	390,150	59.5 (12.9)	40 – 111
Female	8,153	73.0 (11.8)	40 – 101	532,391	60.6 (14.7)	40 – 109
Total	17,408	71.8 (11.2)	40 – 101	922,541	60.1 (14.0)	40 – 111

1 Age at index date: date of diagnosis (cases) or a randomly selected date in the patient’s study period (controls)

**Table 6:** Mixed effect model coefficients for red blood cell count parameters - males.

Variable	RBC	Hb	Hc	MCV	MCH	MCHC
*N*	*376516*	*394601*	*93128*	*388464*	*380868*	*360071*
*n cases*	*9024*	*9161*	*2256*	*8792*	*8631*	*8363*
**Fixed effects:**						
Constant	5.2721 (5.2458, 5.2984)	15.6416 (15.5582, 15.7249)	0.4627 (0.4577, 0.4677)	86.8880 (86.7174, 87.0586)	29.6513 (29.5910, 29.7115)	34.0711 (34.0369, 34.1054)
Age at index date^1^ (years)	-0.0077 (-0.0082, -0.0072)	-0.0142 (-0.0158, -0.0126)	-0.0004 (-0.0005, -0.0003)	0.0777 (0.0744, 0.0810)	0.0194 (0.0183, 0.0206)	-0.0067 (-0.0074, -0.0061)
Age at index date^1^ – knot at 60 (years)	-0.0037 (-0.0051, -0.0024)	-0.0307 (-0.0350, -0.0264)	-0.0008 (-0.0010, -0.0005)	-0.0551 (-0.0642, -0.0461)	-0.0281 (-0.0313, -0.0250)	-0.0117 (-0.0134, -0.0100)
Age at index date^1^ – knot at 70 (years)	-0.0127 (-0.0147, -0.0107)	-0.0326 (-0.0388, -0.0263)	-0.0007 (-0.0011, -0.0003)	0.0094 (-0.0044, 0.0233)	0.0010 (-0.0039, 0.0059)	-0.0043 (-0.0069, -0.0016)
Age at index date^1^ – knot at 80 (years)	-0.0027 (-0.0050, -0.0003)	-0.0040 (-0.0115, 0.0034)	-0.0004 (-0.0009, 0.0000)	0.0289 (0.0112, 0.0465)	0.0080 (0.0019, 0.0143)	0.0021 (-0.0012, 0.0054)
Time to index date^1^ (years)	0.0148 (-0.0197, 0.0493)	-0.0125 (-0.1250, 0.1000)	-0.0014 (-0.0083, 0.0056)	-0.1748 (-0.2074, -0.1422)	-0.0137 (-0.0258, -0.0017)	0.0421 (0.0324, 0.0519)
Time to index date^1^ date – knot at one (years)	-0.0107 (-0.0673, 0.0460)	-0.0014 (-0.1867, 0.1839)	0.0026 (-0.0089, 0.0142)	0.0625 (0.0108, 0.1141)	-0.0018 (-0.0209, 0.0173)	-0.0205 (-0.0361, -0.0049)
Time to index date^1^ date – knot at two (years)	0.0054 (-0.0348, 0.0456)	-0.0063 (-0.1378, 0.1252)	-0.0047 (-0.0131, 0.0037)	-0.0148 (-0.0481, 0.0185)	0.0257 (0.0135, 0.0381)	0.0298 (0.0197, 0.0400)
Time to index date^1^ date – knot at four (years)	-0.0395 (-0.0646, -0.0144)	-0.0437 (-0.1257, 0.0383)	0.0038 (-0.0017, 0.0094)	0.1234 (0.1068, 0.1400)	0.0037 (-0.0024, 0.0098)	-0.0324 (-0.0374, -0.0274)
CRC present	-0.2529 (-0.4629, -0.0428)	-2.5579 (-3.1632, -1.9526)	-0.0740 (-0.1091, -0.0389)	-8.0698 (-10.5710, -5.5686)	-2.1303 (-3.0312, -1.2293)	-1.7476 (-2.2370, -1.2582)
CRC present by time to index date^1^ interaction	0.1869 (0.1732, 0.2005)	1.4226 (1.3771, 1.4682)	0.0321 (0.0293, 0.0350)	4.5147 (4.3692, 4.6603)	1.4979 (1.4426, 1.5532)	0.7227 (0.6773, 0.7681)
CRC present by time to index date^1^ (knot at one) interaction	-0.1625 (-0.1868, -0.1381)	-1.2030 (-1.2841, -1.1220)	-0.0246 (-0.0298, -0.0195)	-3.9394 (-4.1983, -3.6805)	-1.2412 (-1.3386, -1.1438)	-0.6063 (-0.6876, -0.5250)
CRC present by time to index date^1^ (knot at two) interaction	-0.0208 (-0.0384, -0.0033)	-0.1037 (-0.1622, -0.0451)	-0.0050 (-0.0088, -0.0011)	-0.1012 (-0.2886, 0.0862)	-0.0784 (-0.1483, -0.0086)	-0.0287 (-0.0876, 0.0302)
CRC present by time to index date^1^ (knot at four) interaction	-0.0035 (-0.0120, 0.0051)	-0.1046 (-0.1332, -0.0761)	-0.0020 (-0.0041, 0.0000)	-0.4093 (-0.5016, -0.3170)	-0.1545 (-0.1888, -0.1201)	-0.0828 (-0.1114, -0.0542)
Time by age interaction	-0.0000 (-0.0007, 0.0006)	0.0007 (-0.0015, 0.0028)	0.0000 (-0.0001, 0.0002)			
Time (knot at one) by age interaction	0.0003 (-0.0008, 0.0013)	0.0003 (-0.0032, 0.0038)	-0.0001 (-0.0003, 0.0002)			
Time (knot at two) by age interaction	-0.0002 (-0.0009, 0.0006)	0.0000 (-0.0025, 0.0025)	0.0001 (-0.0001, 0.0003)			
Time (knot at four) by age interaction	0.0006 (0.0001, 0.0010)	0.0006 (-0.0009, 0.0021)	-0.0001 (-0.0002, 0.0000)			
Time by age (knot at 60) interaction	0.0016 (-0.0000, 0.0033)	0.0065 (0.0011, 0.0120)	0.0003 (-0.0001, 0.0006)			
Time (knot at one) by age (knot at 60) interaction	-0.0019 (-0.0046, 0.0007)	-0.0067 (-0.0155, 0.0021)	-0.0002 (-0.0007, 0.0004)			
Time (knot at two) by age (knot at 60) interaction	0.0011 (-0.0006, 0.0029)	0.0032 (-0.0027, 0.0090)	-0.0001 (-0.0005, 0.0003)			
Time (knot at four) by age (knot at 60) interaction	-0.0008 (-0.0018, 0.0001)	-0.0019 (-0.0050, 0.0012)	0.0001 (-0.0001, 0.0003)			
Time by age (knot at 70) interaction	0.0009 (-0.0014, 0.0031)	0.0003 (-0.0073, 0.0078)	-0.0002 (-0.0007, 0.0003)			
Time (knot at one) by age (knot at 70) interaction	0.0029 (-0.0007, 0.0065)	0.0095 (-0.0024, 0.0215)	0.0004 (-0.0004, 0.0011)			
Time (knot at two) by age (knot at 70) interaction	-0.0035 (-0.0059, -0.0012)	-0.0109 (-0.0186, -0.0033)	-0.0002 (-0.0007, 0.0003)			
Time (knot at four) by age (knot at 70) interaction	0.0006 (-0.0005, 0.0018)	0.0025 (-0.0012, 0.0062)	0.0000 (-0.0003, 0.0003)			
Time by age (knot at 80) interaction	-0.0011 (-0.0037, 0.0015)	-0.0047 (-0.0135, 0.0041)	0.0000 (-0.0006, 0.0006)			
Time (knot at one) by age (knot at 80) interaction	-0.0028 (-0.0070, 0.0014)	-0.0092 (-0.0232, 0.0048)	-0.0005 (-0.0014, 0.0004)			
Time (knot at two) by age (knot at 80) interaction	0.0048 (0.0021, 0.0075)	0.0199 (0.0108, 0.0290)	0.0006 (0.0000, 0.0012)			
Time (knot at four) by age (knot at 80) interaction	-0.0014 (-0.0027, -0.0000)	-0.0078 (-0.0121, -0.0034)	-0.0001 (-0.0004, 0.0002)			
CRC presence by age interaction	0.0004 (-0.0034, 0.0041)	0.0129 (0.0021, 0.0237)	0.0005 (-0.0001, 0.0011)	0.0441 (-0.0004, 0.0887)	0.0059 (-0.0101, 0.0219)	0.0141 (0.0054, 0.0228)
CRC presence by age (knot at 60) interaction	-0.0003 (-0.0066, 0.0061)	-0.0241 (-0.0422, -0.0059)	-0.0010 (-0.0021, 0.0001)	-0.1196 (-0.1951, -0.0441)	-0.0385 (-0.0656, -0.0115)	-0.0250 (-0.0395, -0.0104)
CRC presence by age (knot at 70) interaction	0.0053 (-0.0008, 0.0115)	0.0155 (-0.0020, 0.0330)	0.0011 (0.0001, 0.0022)	-0.0011 (-0.0747, 0.0724)	0.0161 (-0.0100, 0.0423)	0.0116 (-0.0022, 0.0255)
CRC presence by age (knot at 80) interaction	-0.0094 (-0.0160, -0.0027)	-0.0128 (-0.0319, 0.0063)	-0.0013 (-0.0023, -0.0002)	0.0924 (0.0114, 0.1734)	0.0014 (-0.0272, 0.0301)	-0.0124 (-0.0274, 0.0026)
**Random effects:**						
Intercept for patient (variance)	0.1740 (0.1729, 0.1751)	1.4811 (1.4713, 1.4909)	0.0012 (0.0011, 0.0012)	25.9019 (25.7479, 26.0569)	3.1215 (3.1022, 3.1409)	0.7548 (0.7481, 0.7615)
Slope for time to index date^1^(variance)	0.0020 (0.0019, 0.0020)	0.0248 (0.0244, 0.0252)	0.0000 (0.0000, 0.0000)	0.3127 (0.3078, 0.3178)	0.0384 (0.0378, 0.0390)	0.0137 (0.0134, 0.0141)
Covariance between Intercept for patient and slope for time to index date (covariance)	-0.0097 (-0.0099, -0.0095)	-0.1099 (-0.1117, -0.1082)	-0.0001 (-0.0001, -0.0001)	-1.3578 (-1.3820, -1.3336)	-0.1680 (-0.1711, -0.1650)	-0.0622 (-0.0635, -0.0609)
Residual (variance)	0.0506 (0.0505, 0.0508)	0.6220 (0.6200, 0.6241)	0.0005 (0.0005, 0.0005)	6.0476 (6.0268, 6.0685)	0.8071 (0.8043, 0.8100)	0.5825 (0.5804, 0.5845)

1 Index date was the date of diagnosis for cases and a randomly selected date in the patient’s study period for controls.

Abbreviations: RBC = red blood cells; Hb = haemoglobin; Hc = haematocrit; MCV = mean corpuscular volume; MCH = mean corpuscular haemoglobin; MCHC = mean corpuscular haemoglobin concentration.

**Table 7:** Mixed effect model coefficients for red blood cell count parameters - females.

Variable	RBC	Hb	Hc	MCV	MCH	MCHC
*N*	509513	534243	129276	526287	514876	488199
*n cases*	7976	8070	2052	7745	7567	7396
**Fixed effects:**						
Constant	4.1865 (4.1675, 4.2056)	12.1175 (12.0546, 12.1803)	0.3706 (0.3668, 0.3743)	87.1667 (87.0087, 87.3246)	29.1787 (29.1224, 29.2350)	33.0309 (33.0000, 33.0618)
Age at index date^1^ (years)	0.0050 (0.0047, 0.0054)	0.0238 (0.0225, 0.0250)	0.0006 (0.0005, 0.0007)	0.0649 (0.0617, 0.0680)	0.0182 (0.0171, 0.0193)	0.0033 (0.0027, 0.0039)
Age at index date^1^ – knot at 60 (years)	-0.0107 (-0.0117, -0.0096)	-0.0503 (-0.0538, -0.0467)	-0.0012 (-0.0014, -0.0009)	-0.0621 (-0.0710, -0.0532)	-0.0264 (-0.0295, -0.0232)	-0.0113 (-0.0130, -0.0096)
Age at index date^1^ – knot at 70 (years)	-0.0079 (-0.0094, -0.0063)	-0.0188 (-0.0238, -0.0138)	-0.0007 (-0.0010, -0.0004)	-0.0023 (-0.0154, 0.0108)	-0.0047 (-0.0093, -0.0000)	-0.0088 (-0.0112, -0.0063)
Age at index date^1^ – knot at 80 (years)	-0.0009 (-0.0024, 0.0006)	0.0014 (-0.0036, 0.0064)	0.0001 (-0.0002, 0.0004)	0.0190 (0.0056, 0.0324)	0.0060 (0.0012, 0.0108)	0.0014 (-0.0011, 0.0039)
Time to index date^1^ (years)	-0.0161 (-0.0404, 0.0082)	-0.2697 (-0.3529, -0.1865)	-0.0111 (-0.0161, -0.0060)	-0.3001 (-0.3289, -0.2712)	-0.0532 (-0.0637, -0.0427)	0.0267 (0.0186, 0.0347)
Time to index date^1^ date – knot at one (years)	-0.0063 (-0.0461, 0.0335)	0.2064 (0.0697, 0.3430)	0.0101 (0.0017, 0.0185)	0.1804 (0.1348, 0.2261)	0.0352 (0.0186, 0.0518)	-0.0103 (-0.0231, 0.0025)
Time to index date^1^ date – knot at two (years)	-0.0215 (-0.0496, 0.0066)	-0.1740 (-0.2709, -0.0772)	-0.0072 (-0.0133, -0.0011)	0.0340 (0.0045, 0.0635)	0.0399 (0.0291, 0.0506)	0.0349 (0.0266, 0.0432)
Time to index date^1^ date – knot at four (years)	0.0420 (0.0248, 0.0593)	0.2626 (0.2031, 0.3221)	0.0085 (0.0046, 0.0125)	0.0727 (0.0580, 0.0873)	-0.0073 (-0.0126, -0.0019)	-0.0373 (-0.0414, -0.0332)
CRC present	-0.2530 (-0.4467, -0.0593)	-2.0165 (-2.6006, -1.4324)	-0.0508 (-0.0843, -0.0173)	-6.9632 (-9.7568, -4.1697)	-2.3591 (-3.3783, -1.3399)	-1.1348 (-1.6733, -0.5962)
CRC present by time to index date^1^ interaction	0.1643 (0.1517, 0.1769)	1.3311 (1.2874, 1.3748)	0.0366 (0.0337, 0.0394)	4.8123 (4.6482, 4.9764)	1.6663 (1.6043, 1.7283)	0.6327 (0.5858, 0.6796)
CRC present by time to index date^1^ (knot at one) interaction	-0.1247 (-0.1468, -0.1025)	-1.0582 (-1.1352, -0.9813)	-0.0303 (-0.0354, -0.0253)	-4.1124 (-4.4004, -3.8244)	-1.3530 (-1.4605, -1.2454)	-0.4455 (-0.5282, -0.3628)
CRC present by time to index date^1^ (knot at two) interaction	-0.0336 (-0.0493, -0.0178)	-0.1575 (-0.2123, -0.1026)	-0.0047 (-0.0083, -0.0011)	-0.1472 (-0.3527, 0.0583)	-0.1389 (-0.2148, -0.0630)	-0.1358 (-0.1951, -0.0766)
CRC present by time to index date^1^ (knot at four) interaction	-0.0053 (-0.0130, 0.0023)	-0.0888 (-0.1152, -0.0624)	-0.0012 (-0.0031, 0.0007)	-0.4000 (-0.5002, -0.2999)	-0.1338 (-0.1708, -0.0969)	-0.0112 (-0.0400, 0.0176)
Time by age interaction	0.0003 (-0.0002, 0.0007)	0.0043 (0.0027, 0.0059)	0.0002 (0.0001, 0.0003)			
Time (knot at one) by age interaction	0.0001 (-0.0007, 0.0009)	-0.0037 (-0.0064, -0.0010)	-0.0002 (-0.0003, -0.0000)			
Time (knot at two) by age interaction	0.0004 (-0.0001, 0.0010)	0.0038 (0.0019, 0.0056)	0.0001 (0.0000, 0.0003)			
Time (knot at four) by age interaction	-0.0010 (-0.0013, -0.0006)	-0.0056 (-0.0067, -0.0044)	-0.0002 (-0.0002, -0.0001)			
Time by age (knot at 60) interaction	0.0010 (-0.0003, 0.0023)	0.0015 (-0.0029, 0.0060)	0.0001 (-0.0002, 0.0003)			
Time (knot at one) by age (knot at 60) interaction	-0.0002 (-0.0023, 0.0018)	0.0039 (-0.0032, 0.0110)	0.0001 (-0.0004, 0.0005)			
Time (knot at two) by age (knot at 60) interaction	-0.0005 (-0.0019, 0.0009)	-0.0061 (-0.0108, -0.0014)	-0.0002 (-0.0005, 0.0001)			
Time (knot at four) by age (knot at 60) interaction	0.0015 (0.0008, 0.0022)	0.0100 (0.0076, 0.0124)	0.0003 (0.0001, 0.0005)			
Time by age (knot at 70) interaction	0.0000 (-0.0017, 0.0017)	-0.0025 (-0.0085, 0.0035)	-0.0002 (-0.0006, 0.0002)			
Time (knot at one) by age (knot at 70) interaction	0.0002 (-0.0025, 0.0030)	-0.0002 (-0.0098, 0.0094)	0.0003 (-0.0003, 0.0009)			
Time (knot at two) by age (knot at 70) interaction	-0.0006 (-0.0023, 0.0012)	0.0010 (-0.0051, 0.0072)	-0.0000 (-0.0004, 0.0003)			
Time (knot at four) by age (knot at 70) interaction	-0.0002 (-0.0011, 0.0006)	-0.0041 (-0.0070, -0.0011)	-0.0001 (-0.0003, 0.0001)			
Time by age (knot at 80) interaction	-0.0003 (-0.0020, 0.0014)	-0.0044 (-0.0103, 0.0016)	-0.0000 (-0.0004, 0.0004)			
Time (knot at one) by age (knot at 80) interaction	-0.0004 (-0.0031, 0.0023)	0.0047 (-0.0048, 0.0141)	-0.0001 (-0.0007, 0.0005)			
Time (knot at two) by age (knot at 80) interaction	0.0006 (-0.0012, 0.0023)	-0.0012 (-0.0073, 0.0049)	-0.0001 (-0.0005, 0.0003)			
Time (knot at four) by age (knot at 80) interaction	-0.0004 (-0.0013, 0.0004)	0.0002 (-0.0027, 0.0031)	0.0002 (-0.0000, 0.0004)			
CRC presence by age interaction	0.0010 (-0.0025, 0.0045)	0.0057 (-0.0048, 0.0163)	0.0002 (-0.0004, 0.0008)	0.0151 (-0.0355, 0.0657)	0.0069 (-0.0115, 0.0254)	0.0051 (-0.0046, 0.0149)
CRC presence by age (knot at 60) interaction	-0.0030 (-0.0095, 0.0034)	-0.0255 (-0.0449, -0.0061)	-0.0010 (-0.0021, 0.0001)	-0.0800 (-0.1733, 0.0132)	-0.0370 (-0.0710, -0.0031)	-0.0188 (-0.0366, -0.0010)
CRC presence by age (knot at 70) interaction	0.0027 (-0.0036, 0.0090)	0.0163 (-0.0026, 0.0352)	0.0009 (-0.0002, 0.0019)	0.0025 (-0.0892, 0.0943)	0.0068 (-0.0264, 0.0400)	0.0083 (-0.0088, 0.0255)
CRC presence by age (knot at 80) interaction	-0.0006 (-0.0060, 0.0049)	-0.0038 (-0.0200, 0.0124)	-0.0001 (-0.0011, 0.0008)	0.0359 (-0.0429, 0.1147)	0.0080 (-0.0205, 0.0364)	-0.0027 (-0.0173, 0.0119)
**Random effects:**						
Intercept for patient (variance)	0.1372 (0.1365, 0.1380)	1.2528 (1.2456, 1.2601)	0.0010 (0.0010, 0.0010)	29.6896 (29.5368, 29.8432)	3.7164 (3.6968, 3.7362)	0.9040 (0.8976, 0.9104)
Slope for time to index date^1^(variance)	0.0015 (0.0015, 0.0016)	0.0212 (0.0209, 0.0215)	0.0000 (0.0000, 0.0000)	0.4220 (0.4166, 0.4276)	0.0538 (0.0531, 0.0545)	0.0174 (0.0171, 0.0177)
Covariance between Intercept for patient and slope for time to index date (covariance)	-0.0071 (-0.0072, -0.0069)	-0.0829 (-0.0842, -0.0816)	-0.0001 (-0.0001, -0.0001)	-1.4631 (-1.4882, -1.4380)	-0.1961 (-0.1994, -0.1929)	-0.0713 (-0.0725, -0.0701)
Residual (variance)	0.0447 (0.0446, 0.0449)	0.5960 (0.5945, 0.5976)	0.0005 (0.0004, 0.0005)	7.9522 (7.9311, 7.9734)	1.0205 (1.0178, 1.0233)	0.6239 (0.6222, 0.6256)

1 Index date was the date of diagnosis for cases and a randomly selected date in the patient’s study period for controls.

Abbreviations: RBC = red blood cells; Hb = haemoglobin; Hc = haematocrit; MCV = mean corpuscular volume; MCH = mean corpuscular haemoglobin; MCHC = mean corpuscular haemoglobin concentration.

**Table 8:** Mixed effect model coefficients for platelet parameters.

Variable	Males		Females	
	Platelets	MPV	Platelets	MPV
*N*	*383016*	*59412*	*517999*	*81765*
*n cases*	*1283*	*8912*	*1175*	*7868*
**Fixed effects:**				
Constant	257.1629 (254.9297, 259.3962)	9.5231 (9.4712, 9.5749)	276.9223 (274.8745, 278.9700)	9.6562 (9.6165, 9.6959)
Age at index date^1^ (years)	-0.2013 (-0.2445, -0.1581)	-0.0031 (-0.0040, -0.0023)	-0.0199 (-0.0603, 0.0205)	-0.0046 (-0.0052, -0.0040)
Age at index date^1^ – knot at 60 (years)	-0.5702 (-0.6874, -0.4529)		-0.4970 (-0.6110, -0.3830)	
Age at index date^1^ – knot at 70 (years)	0.4389 (0.2598, 0.6180)		0.9261 (0.7584, 1.0938)	
Age at index date^1^ – knot at 80 (years)	0.1632 (-0.0635, 0.3899)		-0.7335 (-0.9056, -0.5615)	
Time to index date^1^ (years)	2.7155 (2.2004, 3.2306)	-0.0974 (-0.1178, -0.0770)	3.1482 (2.7212, 3.5753)	-0.0902 (-0.1057, -0.0746)
Time to index date^1^ date – knot at one (years)	-0.7797 (-1.6028, 0.0434)	0.0433 (0.0112, 0.0753)	-1.3100 (-1.9898, -0.6302)	0.0331 (0.0087, 0.0576)
Time to index date^1^ date – knot at two (years)	0.3853 (-0.1495, 0.9200)	0.0291 (0.0084, 0.0498)	-0.1326 (-0.5733, 0.3080)	0.0453 (0.0295, 0.0612)
Time to index date^1^ date – knot at four (years)	-2.2486 (-2.5110, -1.9862)	0.0148 (0.0038, 0.0258)	-2.1814 (-2.3969, -1.9658)	-0.0010 (-0.0094, 0.0074)
CRC present	128.6209 (96.3364, 160.9054)	-0.3766 (-0.4700, -0.2831)	110.9094 (74.8031, 147.0156)	-0.4480 (-0.5434, -0.3525)
CRC present by time to index date^1^ interaction	-57.3824 (-59.7265, -55.0383)	0.1735 (0.0770, 0.2699)	-70.0709 (-72.5177, -67.6241)	0.3215 (0.2301, 0.4130)
CRC present by time to index date^1^ (knot at one) interaction	50.8421 (46.6410, 55.0433)	-0.1107 (-0.2790, 0.0576)	61.9149 (57.5957, 66.2340)	-0.2983 (-0.4579, -0.1387)
CRC present by time to index date^1^ (knot at two) interaction	2.9225 (-0.1281, 5.9731)	-0.0245 (-0.1446, 0.0955)	3.9056 (0.8180, 6.9931)	-0.0328 (-0.1470, 0.0813)
CRC present by time to index date^1^ (knot at four) interaction	3.5438 (2.0653, 5.0224)	-0.0356 (-0.0989, 0.0276)	3.0603 (1.5774, 4.5433)	0.0439 (-0.0152, 0.1031)
CRC presence by age interaction	-0.9456 (-1.5201, -0.3710)		-0.4272 (-1.0804, 0.2260)	
CRC presence by age (knot at 60) interaction	0.7702 (-0.2018, 1.7422)		1.2526 (0.0544, 2.4507)	
CRC presence by age (knot at 70) interaction	0.9991 (0.0572, 1.9410)		-0.8446 (-2.0166, 0.3275)	
CRC presence by age (knot at 80) interaction	-2.1333 (-3.1669, -1.0998)		-0.0662 (-1.0705, 0.9380)	
**Random effects:**				
Intercept for patient (variance)	3.8e+03 (3.7e+03, 3.8e+03)	1.8264 (1.7987, 1.8546)	4.5e+03 (4.5e+03, 4.6e+03)	1.8457 (1.8225, 1.8691)
Slope for time to index date^1^ (variance)	39.5014 (38.6595, 40.3616)	0.0271 (0.0260, 0.0282)	47.4155 (46.6445, 48.1993)	0.0274 (0.0266, 0.0283)
Covariance between Intercept for patient and slope for time to index date (covariance)	-1.7e+02 (-1.7e+02, -1.6e+02)	-0.1103 (-0.1151, -0.1055)	-1.9e+02 (-2.0e+02, -1.9e+02)	-0.1117 (-0.1155, -0.1079)
Residual (variance)	1.6e+03 (1.6e+03, 1.7e+03)	0.2919 (0.2890, 0.2949)	1.8e+03 (1.8e+03, 1.8e+03)	0.2884 (0.2862, 0.2906)

1 Index date was the date of diagnosis for cases and a randomly selected date in the patient’s study period for controls.

Abbreviations: MPV = mean platelet volume.

**Table 9:** Mixed effect model coefficients for white blood cell count parameters - males.

Variable	WBC	Basophils	Eosinophils	Lymphocytes	Monocytes	Neutrophils
*N*	*356191*	*328523*	*329715*	*338453*	*335510*	*339093*
*n cases*	*8133*	*7869*	*7892*	*8113*	*8055*	*8509*
**Fixed effects:**						
Constant	6.8860 (6.7850, 6.9871)	0.0653 (0.0645, 0.0660)	0.2148 (0.2110, 0.2186)	2.4595 (2.4323, 2.4867)	0.4892 (0.4808, 0.4977)	3.9542 (3.8711, 4.0372)
Age at index date^1^ (years)	0.0050 (0.0031, 0.0070)	0.0000 (-0.0000, 0.0000)	0.0003 (0.0002, 0.0003)	-0.0063 (-0.0067, -0.0058)	0.0013 (0.0012, 0.0015)	0.0048 (0.0032, 0.0063)
Age at index date^1^ – knot at 60 (years)	0.0025 (-0.0025, 0.0074)				0.0008 (0.0003, 0.0012)	0.0141 (0.0113, 0.0170)
Age at index date^1^ – knot at 70 (years)	0.0066 (0.0004, 0.0127)		0.0006 (0.0005, 0.0008)		0.0001 (-0.0003, 0.0006)	
Age at index date^1^ – knot at 80 (years)				-0.0135 (-0.0165, -0.0105)		0.0010 (-0.0049, 0.0070)
Age at index date^1^ – knot at 85 (years)	-0.0126 (-0.0254, 0.0002)					
Time to index date^1^ (years)	0.0046 (-0.0261, 0.0354)	-0.0002 (-0.0002, -0.0001)	-0.0009 (-0.0011, -0.0008)	0.0102 (-0.0019, 0.0223)	-0.0040 (-0.0065, -0.0016)	-0.0436 (-0.0706, -0.0166)
Time to index date^1^ date – knot at one (years)	-0.0248 (-0.0743, 0.0246)			0.0058 (-0.0134, 0.0249)	-0.0031 (-0.0070, 0.0008)	0.0254 (-0.0186, 0.0694)
Time to index date^1^ date – knot at two (years)	0.0081 (-0.0244, 0.0405)			-0.0029 (-0.0152, 0.0094)	0.0021 (-0.0005, 0.0046)	0.0091 (-0.0206, 0.0388)
Time to index date^1^ date – knot at four (years)	0.0105 (-0.0054, 0.0264)			-0.0025 (-0.0088, 0.0037)	0.0004 (-0.0008, 0.0018)	0.0086 (-0.0064, 0.0235)
CRC present	-0.5156 (-1.9304, 0.8992)	0.0018 (0.0006, 0.0031)	-0.0198 (-0.0636, 0.0241)	-0.1594 (-0.2052, -0.1136)	0.1034 (0.0959, 0.1108)	1.4463 (0.3253, 2.5673)
CRC present by time to index date^1^ interaction	-0.8386 (-0.9812, -0.6959)	-0.0002 (-0.0005, 0.0001)	-0.0021 (-0.0031, -0.0012)	0.1662 (0.1123, 0.2202)	-0.0823 (-0.0935, -0.0710)	-0.9473 (-1.0745, -0.8201)
CRC present by time to index date^1^ (knot at one) interaction	0.6959 (0.4376, 0.9541)			-0.1616 (-0.2574, -0.0658)	0.0806 (0.0602, 0.1011)	0.8945 (0.6613, 1.1278)
CRC present by time to index date^1^ (knot at two) interaction	0.1083 (-0.0814, 0.2980)			0.0037 (-0.0658, 0.0731)	-0.0079 (-0.0230, 0.0072)	0.0181 (-0.1570, 0.1933)
CRC present by time to index date^1^ (knot at four) interaction	0.0194 (-0.0721, 0.1110)			-0.0147 (-0.0497, 0.0202)	0.0100 (0.0026, 0.0174)	0.0487 (-0.0376, 0.1349)
CRC presence by age interaction	0.0332 (0.0081, 0.0583)		0.0006 (-0.0001, 0.0013)			-0.0053 (-0.0249, 0.0144)
CRC presence by age (knot at 60) interaction	-0.0825 (-0.1236, -0.0415)					0.0015 (-0.0232, 0.0263)
CRC presence by age (knot at 70) interaction	0.0546 (0.0221, 0.0872)		-0.0026 (-0.0037, -0.0014)			
CRC presence by age (knot at 80) interaction						-0.0134 (-0.0403, 0.0135)
CRC presence by age (knot at 85) interaction	-0.0695 (-0.1293, -0.0097)					
**Random effects:**						
Intercept for patient (variance)	5.3129 (5.2597, 5.3667)	0.0016 (0.0016, 0.0017)	0.0219 (0.0217, 0.0221)	2.3665 (2.3502, 2.3830)	0.0348 (0.0344, 0.0351)	2.1016 (2.0698, 2.1338)
Slope for time to index date^1^ (variance)	0.0876 (0.0853, 0.0899)	0.0000 (0.0000, 0.0000)	0.0003 (0.0003, 0.0003)	0.0583 (0.0574, 0.0592)	0.0005 (0.0005, 0.0006)	0.0410 (0.0389, 0.0432)
Covariance between Intercept for patient and slope for time to index date (covariance)	-0.3649 (-0.3756, -0.3542)	-0.0001 (-0.0001, -0.0001)	-0.0011 (-0.0012, -0.0011)	-0.2447 (-0.2483, -0.2410)	-0.0014 (-0.0014, -0.0013)	0.1588 (0.1528, 0.1648)
Residual (variance)	5.8167 (5.7964, 5.8372)	0.0017 (0.0017, 0.0017)	0.0141 (0.0140, 0.0141)	0.6405 (0.6380, 0.6430)	0.0334 (0.0333, 0.0336)	4.7837 (4.7650, 4.8024)

1 Index date was the date of diagnosis for cases and a randomly selected date in the patient’s study period for controls.

Abbreviations: WBC = white blood cell count.

**Table 10:** Mixed effect model coefficients for white blood cell count parameters - females.

Variable	WBC	Basophils	Eosinophils	Lymphocytes	Monocytes	Neutrophils
*N*	*485552*	*444145*	*445731*	*461204*	*456981*	*461893*
*n cases*	*7249*	*6941*	*6966*	*7224*	*7155*	*7574*
**Fixed effects:**						
Constant	8.1097 (8.0197, 8.1997)	0.0614 (0.0608, 0.0619)	0.1950 (0.1922, 0.1978)	2.0705 (2.0519, 2.0891)	0.5118 (0.5057, 0.5179)	5.7413 (5.6847, 5.7978)
Age at index date^1^ (years)	-0.0218 (-0.0235, -0.0200)	0.0001 (0.0001, 0.0001)	0.0001 (0.0000, 0.0001)	-0.0001 (-0.0004, 0.0002)	-0.0005 (-0.0006, -0.0004)	-0.0311 (-0.0322, -0.0300)
Age at index date^1^ – knot at 60 (years)	0.0461 (0.0416, 0.0507)				0.0037 (0.0033, 0.0040)	0.0652 (0.0633, 0.0671)
Age at index date^1^ – knot at 70 (years)	0.0041 (-0.0013, 0.0095)		0.0001 (0.0000, 0.0002)		0.0017 (0.0012, 0.0022)	
Age at index date^1^ – knot at 80 (years)				-0.0337 (-0.0357, -0.0316)	-0.0029 (-0.0034, -0.0024)	-0.0131 (-0.0161, -0.0100)
Age at index date^1^ – knot at 85 (years)	-0.0395 (-0.0473, -0.0317)					
Age at index date^1^ – knot at 90 (years)				0.0420 (0.0353, 0.0486)		
Time to index date^1^ (years)	0.0004 (-0.0289, 0.0298)	-0.0003 (-0.0003, -0.0003)	-0.0004 (-0.0005, -0.0002)	0.0192 (0.0098, 0.0286)	-0.0042 (-0.0058, -0.0025)	-0.0226 (-0.0417, -0.0035)
Time to index date^1^ date – knot at one (years)	-0.0040 (-0.0512, 0.0433)			-0.0118 (-0.0268, 0.0032)	0.0004 (-0.0023, 0.0031)	0.0269 (-0.0039, 0.0577)
Time to index date^1^ date – knot at two (years)	-0.0011 (-0.0320, 0.0299)			0.0020 (-0.0077, 0.0117)	-0.0016 (-0.0034, 0.0001)	-0.0066 (-0.0270, 0.0137)
Time to index date^1^ date – knot at four (years)	0.0114 (-0.0036, 0.0265)			-0.0039 (-0.0086, 0.0008)	0.0022 (0.0013, 0.0030)	0.0130 (0.0029, 0.0231)
CRC present	0.4778 (-1.0104, 1.9660)	0.0016 (0.0003, 0.0029)	0.0020 (-0.0378, 0.0418)	-0.1701 (-0.2112, -0.1290)	0.1282 (0.1214, 0.1350)	1.5446 (1.4734, 1.6159)
CRC present by time to index date^1^ interaction	-1.4093 (-1.5821, -1.2365)	-0.0003 (-0.0006, -0.0000)	-0.0019 (-0.0028, -0.0011)	0.1314 (0.0773, 0.1855)	-0.1098 (-0.1195, -0.1002)	-1.4270 (-1.5393, -1.3147)
CRC present by time to index date^1^ (knot at one) interaction	1.4600 (1.1503, 1.7696)			-0.1227 (-0.2185, -0.0269)	0.1090 (0.0918, 0.1263)	1.4181 (1.2162, 1.6200)
CRC present by time to index date^1^ (knot at two) interaction	-0.0444 (-0.2687, 0.1799)			0.0302 (-0.0384, 0.0989)	-0.0007 (-0.0132, 0.0118)	0.0331 (-0.1145, 0.1808)
CRC present by time to index date^1^ (knot at four) interaction	-0.0650 (-0.1713, 0.0413)			-0.0378 (-0.0706, -0.0049)	-0.0036 (-0.0096, 0.0025)	-0.1051 (-0.1766, -0.0335)
CRC presence by age interaction	0.0190 (-0.0079, 0.0459)		0.0002 (-0.0004, 0.0008)			
CRC presence by age (knot at 60) interaction	-0.0325 (-0.0800, 0.0150)					
CRC presence by age (knot at 70) interaction	0.0125 (-0.0251, 0.0501)		-0.0011 (-0.0021, -0.0001)			
CRC presence by age (knot at 85) interaction	0.0202 (-0.0244, 0.0647)					
**Random effects:**						
Intercept for patient (variance)	4.9756 (4.9232, 5.0286)	0.0016 (0.0016, 0.0016)	0.0169 (0.0167, 0.0170)	1.3624 (1.3534, 1.3716)	0.0295 (0.0292, 0.0297)	1.9321 (1.9110, 1.9535)
Slope for time to index date^1^ (variance)	0.1120 (0.1090, 0.1151)	0.0000 (0.0000, 0.0000)	0.0002 (0.0002, 0.0002)	0.0147 (0.0144, 0.0151)	0.0004 (0.0004, 0.0004)	0.0471 (0.0459, 0.0484)
Covariance between Intercept for patient and slope for time to index date (covariance)	-0.4378 (-0.4496, -0.4261)	-0.0001 (-0.0001, -0.0001)	-0.0009 (-0.0009, -0.0009)	-0.1000 (-0.1017, -0.0983)	-0.0018 (-0.0019, -0.0018)	-0.1056 (-0.1102, -0.1010)
Residual (variance)	9.0166 (8.9919, 9.0413)	0.0016 (0.0016, 0.0016)	0.0116 (0.0116, 0.0116)	0.7342 (0.7320, 0.7364)	0.0243 (0.0242, 0.0244)	3.6243 (3.6139, 3.6347)

1 Index date was the date of diagnosis for cases and a randomly selected date in the patient’s study period for controls.

Abbreviations: WBC = white blood cell count.

**Table 11:** Hazard ratios of age-adjusted FBC trends from univariate joint models.

FBC parameter	Cases	Controls	Hazard ratio (95% CI)
**Males:**			
Red blood cell count ↓	1135	48865	1.957 (1.706, 2.242)
Haemoglobin ↓	1230	48770	1.783 (1.730, 1.835)
Haematocrit ↓	1274	48726	1.001 (1.001, 1.001)
Mean corpuscular volume ↓	1181	48819	1.124 (1.116, 1.133)
Mean corpuscular haemoglobin ↓	1134	48866	1.376 (1.342, 1.410)
Mean corpuscular haemoglobin concentration ↓	1178	48822	2.169 (2.053, 2.288)
Platelets ↑	1107	48893	1.007 (1.006, 1.007)
Mean platelet volume ↓	1901	48909	1.142 (1.089, 1.196)
White blood cell count ↑	1203	48797	1.054 (1.012, 1.098)
Basophil count ↓	1207	48793	0.210 (0.040, 1.101)
Eosinophil count ↓	1233	48767	0.689 (0.440, 1.079)
Lymphocyte count ↓	1222	48778	1.235 (1.139, 1.339)
Monocyte count ↑	1164	48836	1.729 (1.463, 2.043)
Neutrophil count ↑	1186	48814	1.018 (0.970, 1.068)
**Females:**			
Red blood cell count ↓	734	49266	2.551 (2.132, 3.049)
Haemoglobin ↓	747	49253	2.037 (1.953, 2.128)
Haematocrit ↓	775	49225	1.001 (1.001, 1.001)
Mean corpuscular volume ↓	758	49242	1.110 (1.100, 1.121)
Mean corpuscular haemoglobin ↓	716	49284	1.333 (1.292, 1.376)
Mean corpuscular haemoglobin concentration ↓	736	49264	1.862 (1.748, 1.984)
Platelets ↑	718	49282	1.007 (1.006, 1.007)
Mean platelet volume ↓	734	49266	1.155 (1.092, 1.221)
White blood cell count ↑	809	49191	1.047 (0.973, 1.128)
Basophil count ↓	788	49212	0.225 (0.028, 1.835)
Eosinophil count ↓	796	49204	0.484 (0.252, 0.929)
Lymphocyte count ↓	775	49225	1.215 (1.098, 1.346)
Monocyte count ↑	784	49216	2.210 (1.691, 2.889)
Neutrophil count ↑	785	49215	1.093 (1.065, 1.121)

**Table 12:** Adjusted odds ratios for diagnosis between patients with and without microcytic anaemia at six-monthly (+/- three months) time intervals prior to index date^1^.

Time interval (years)	Cases		Controls		Odds ratio (95% CI)^2^
	Microcytic anaemia	Total	Microcytic anaemia	Total	
**Males:**					
0	1188	5107	937	69648	19.6 (95% CI=17.7, 21.7)
0.5	529	3424	1456	115123	10.4 (95% CI=9.1, 11.8)
1	171	2560	1301	103281	4.2 (95% CI=3.4, 5.1)
1.5	115	2280	1058	91084	3.5 (95% CI=2.7, 4.4)
2	83	2025	930	79130	2.4 (95% CI=1.8, 3.3)
2.5	63	1837	864	70678	2.3 (95% CI=1.7, 3.1)
3	49	1662	757	61840	2.2 (95% CI=1.5, 3.1)
3.5	29	1593	648	54331	0.9 (95% CI=0.6, 1.5)
4	18	1380	549	47470	0.9 (95% CI=0.5, 1.6)
4.5	15	1186	505	41655	1.0 (95% CI=0.6, 1.7)
5	15	1063	419	36578	1.0 (95% CI=0.5, 1.8)
**Females:**					
0	1286	4521	3473	105359	12.2 (95% CI=11.3, 13.3)
0.5	588	3237	5684	171034	6.6 (95% CI=5.9, 7.4)
1	208	2483	4948	152754	2.9 (95% CI=2.4, 3.4)
1.5	178	2194	4360	136087	3.0 (95% CI=2.5, 3.6)
2	108	1976	3822	119565	2.0 (95% CI=1.6, 2.6)
2.5	82	1814	3376	106114	1.5 (95% CI=1.1, 2.0)
3	66	1600	2963	93266	1.7 (95% CI=1.3, 2.3)
3.5	44	1492	2597	82236	1.1 (95% CI=0.7, 1.5)
4	40	1359	2233	73537	1.3 (95% CI=0.9, 1.9)
4.5	31	1266	1920	64388	0.8 (95% CI=0.5, 1.3)
5	29	1097	1722	56759	0.9 (95% CI=0.5, 1.4)

1 Index date was the date of diagnosis for cases and a randomly selected date in the patient’s study period for controls

2 Adjusted for age at diagnosis (years)

**Table 13:** Number of cases per Duke’s tumour stage at diagnosis.

Duke’s stage	Males		Females	
	Number	% of cases	Number	% of cases
**A**	850	9.2%	688	8.4%
**B**	2,049	22.1%	1,778	21.8%
**C**	2,038	22.0%	1,797	22.0%
**D**	737	7.8%	532	6.5%
**Unknown**	3,581	38.7%	3,358	41.2%
**Total**	9,255	100%	8,153	100%

**Table 14:** Mixed effect model coefficients for red blood cell count parameters (cases only) containing the Duke’s stage at diagnosis - males.

Variable	RBC	Hb	Hc	MCV	MCH	MCHC
*N*	1515	1571	423	1551	1503	1427
*n cases*	725	736	214	711	706	661
**Fixed effects:**						
Constant	4.4035 (3.5802, 5.2267)	9.9164 (6.8338, 12.9990)	0.4394 (0.2527, 0.6261)	76.8717 (67.8548, 85.8886)	26.1164 (22.8572, 29.3757)	30.6694 (28.9221, 32.4166)
Age at index date^1^ (years)	0.0059 (-0.0088, 0.0206)	0.0674 (0.0122, 0.1225)	-0.0006 (-0.0040, 0.0027)	0.1869 (0.0271, 0.3467)	0.0597 (0.0019, 0.1174)	0.0403 (0.0094, 0.0713)
Age at index date^1^ – knot at 60 (years)	-0.0341 (-0.0595, -0.0088)	-0.1571 (-0.2520, -0.0621)	-0.0019 (-0.0077, 0.0038)	-0.1782 (-0.4420, 0.0856)	-0.0656 (-0.1608, 0.0296)	-0.0575 (-0.1081, -0.0069)
Age at index date^1^ – knot at 70 (years)	0.0255 (0.0007, 0.0503)	0.0398 (-0.0524, 0.1321)	0.0030 (-0.0024, 0.0084)	-0.1188 (-0.3732, 0.1356)	-0.0565 (-0.1482, 0.0351)	-0.0092 (-0.0575, 0.0392)
Age at index date^1^ – knot at 80 (years)	-0.0526 (-0.0852, -0.0200)	-0.0548 (-0.1697, 0.0601)	-0.0011 (-0.0071, 0.0048)	0.2693 (-0.0910, 0.6297)	0.0847 (-0.0445, 0.2139)	-0.0229 (-0.0930, 0.0471)
Time to index date^1^ (years)	0.3864 (-0.5294, 1.3021)	5.3873 (1.7396, 9.0351)	0.0942 (-0.1498, 0.3382)	3.1593 (2.5114, 3.8072)	1.1156 (0.8740, 1.3571)	0.6050 (0.4330, 0.7770)
Time to index date^1^ date – knot at one (years)	-0.2071 (-1.8449, 1.4307)	-7.4027 (-13.8704, -0.9350)	-0.1852 (-0.5750, 0.2045)	-3.5320 (-4.6623, -2.4018)	-1.2569 (-1.6765, -0.8373)	-0.6380 (-0.9382, -0.3377)
Time to index date^1^ date – knot at two (years)	-0.2240 (-1.4605, 1.0125)	1.4891 (-3.3180, 6.2961)	0.0021 (-0.3250, 0.3292)	0.5712 (-0.2145, 1.3568)	0.2999 (0.0082, 0.5916)	0.2018 (-0.0068, 0.4104)
Time to index date^1^ date – knot at four (years)	-0.1963 (-0.8706, 0.4779)	0.2407 (-2.4176, 2.8990)	0.0804 (-0.2513, 0.4120)	-0.0318 (-0.3913, 0.3277)	-0.1184 (-0.2531, 0.0163)	-0.1839 (-0.2781, -0.0897)
Dukes D (vs. A)	1.2082 (0.1845, 2.2319)	-0.3514 (-3.8808, 3.1780)	-0.0991 (-0.3154, 0.1172)	-13.3449 (-27.1015, 0.4117)	-5.5770 (-10.5296, -0.6245)	-0.6897 (-3.3618, 1.9824)
Dukes D (vs. A) by time to index date^1^ interaction	0.0133 (-0.0550, 0.0817)	0.2794 (0.0148, 0.5441)	-0.0214 (-0.0367, -0.0060)	1.2163 (0.3164, 2.1162)	0.6000 (0.2613, 0.9388)	0.0991 (-0.1409, 0.3391)
Dukes D (vs. A) by time to index date^1^ (knot at one) interaction	0.0410 (-0.0818, 0.1639)	0.0667 (-0.4056, 0.5391)	0.0558 (0.0280, 0.0835)	-0.1100 (-1.7108, 1.4908)	-0.1705 (-0.7691, 0.4280)	0.0550 (-0.3734, 0.4834)
Dukes D (vs. A) by time to index date^1^ (knot at two) interaction	-0.0298 (-0.1197, 0.0601)	-0.2409 (-0.5837, 0.1019)	-0.0352 (-0.0562, -0.0141)	-0.7959 (-1.9493, 0.3576)	-0.2955 (-0.7248, 0.1337)	-0.1125 (-0.4221, 0.1970)
Dukes D (vs. A) by time to index date^1^ (knot at four) interaction	-0.0231 (-0.0664, 0.0201)	-0.0772 (-0.2428, 0.0883)	-0.0001 (-0.0118, 0.0117)	-0.4362 (-0.9805, 0.1081)	-0.1503 (-0.3543, 0.0537)	-0.0132 (-0.1575, 0.1312)
Time by age interaction	-0.0052 (-0.0215, 0.0111)	-0.0768 (-0.1417, -0.0118)	-0.0010 (-0.0053, 0.0033)			
Time (knot at one) by age interaction	0.0028 (-0.0264, 0.0320)	0.1098 (-0.0053, 0.2250)	0.0025 (-0.0044, 0.0094)			
Time (knot at two) by age interaction	0.0035 (-0.0185, 0.0256)	-0.0207 (-0.1062, 0.0647)	0.0001 (-0.0056, 0.0059)			
Time (knot at four) by age interaction	0.0029 (-0.0091, 0.0148)	-0.0070 (-0.0540, 0.0399)	-0.0014 (-0.0071, 0.0043)			
Time by age (knot at 60) interaction	0.0215 (-0.0059, 0.0488)	0.1336 (0.0256, 0.2415)	0.0042 (-0.0025, 0.0108)			
Time (knot at one) by age (knot at 60) interaction	-0.0229 (-0.0721, 0.0263)	-0.1587 (-0.3506, 0.0332)	-0.0069 (-0.0181, 0.0044)			
Time (knot at two) by age (knot at 60) interaction	-0.0038 (-0.0403, 0.0326)	-0.0063 (-0.1461, 0.1335)	0.0009 (-0.0081, 0.0100)			
Time (knot at four) by age (knot at 60) interaction	0.0049 (-0.0136, 0.0234)	0.0310 (-0.0411, 0.1030)	0.0010 (-0.0064, 0.0085)			
Time by age (knot at 70) interaction	-0.0288 (-0.0534, -0.0042)	-0.0989 (-0.1946, -0.0032)	-0.0062 (-0.0120, -0.0005)			
Time (knot at one) by age (knot at 70) interaction	0.0374 (-0.0065, 0.0814)	0.1150 (-0.0548, 0.2849)	0.0086 (-0.0019, 0.0191)			
Time (knot at two) by age (knot at 70) interaction	0.0040 (-0.0271, 0.0351)	0.0200 (-0.0989, 0.1389)	-0.0022 (-0.0100, 0.0055)			
Time (knot at four) by age (knot at 70) interaction	-0.0196 (-0.0338, -0.0053)	-0.0401 (-0.0950, 0.0149)	0.0006 (-0.0037, 0.0049)			
Time by age (knot at 80) interaction	0.0345 (0.0115, 0.0575)	0.0858 (-0.0041, 0.1756)	0.0027 (-0.0020, 0.0074)			
Time (knot at one) by age (knot at 80) interaction	-0.0484 (-0.0889, -0.0078)	-0.1608 (-0.3179, -0.0037)	-0.0063 (-0.0148, 0.0021)			
Time (knot at two) by age (knot at 80) interaction	0.0031 (-0.0270, 0.0332)	0.0706 (-0.0441, 0.1853)	0.0034 (-0.0031, 0.0099)			
Time (knot at four) by age (knot at 80) interaction	0.0161 (0.0008, 0.0313)	0.0029 (-0.0560, 0.0619)	0.0003 (-0.0036, 0.0042)			
Dukes D (vs. A) by age interaction	-0.0232 (-0.0415, -0.0050)	-0.0068 (-0.0696, 0.0559)	0.0015 (-0.0023, 0.0053)	0.1972 (-0.0471, 0.4414)	0.0831 (-0.0048, 0.1710)	0.0103 (-0.0370, 0.0577)
Dukes D (vs. A) by age (knot at 60) interaction	0.0375 (0.0065, 0.0685)	0.0010 (-0.1040, 0.1061)	-0.0011 (-0.0074, 0.0052)	-0.3402 (-0.7459, 0.0654)	-0.1877 (-0.3337, -0.0417)	-0.0645 (-0.1428, 0.0137)
Dukes D (vs. A) by age (knot at 70) interaction	-0.0200 (-0.0502, 0.0103)	0.0313 (-0.0685, 0.1311)	-0.0010 (-0.0068, 0.0047)	0.3235 (-0.0607, 0.7077)	0.2145 (0.0766, 0.3524)	0.0881 (0.0146, 0.1615)
Dukes D (vs. A) by age (knot at 80) interaction	0.0258 (-0.0112, 0.0628)	-0.0375 (-0.1562, 0.0812)	0.0024 (-0.0039, 0.0088)	-0.5334 (-0.9972, -0.0697)	-0.2267 (-0.3930, -0.0604)	-0.0222 (-0.1116, 0.0672)
**Random effects:**						
Intercept for patient (variance)	0.1923 (0.1756, 0.2107)	2.9902 (2.7319, 3.2730)	0.0023 (0.0019, 0.0027)	45.3333 (41.6183, 49.3800)	5.3157 (4.8602, 5.8138)	1.3501 (1.2131, 1.5027)
Slope for time to index date^1^ (variance)	0.0027 (0.0021, 0.0033)	0.0770 (0.0650, 0.0913)	0.0001 (0.0000, 0.0001)	0.8162 (0.6868, 0.9701)	0.1059 (0.0895, 0.1252)	0.0260 (0.0210, 0.0321)
Covariance between Intercept for patient and slope for time to index date (covariance)	-0.0117 (-0.0143, -0.0090)	-0.3652 (-0.4175, -0.3128)	-0.0003 (-0.0004, -0.0002)	-4.7011 (-5.3479, -4.0543)	-0.5593 (-0.6397, -0.4789)	-0.1499 (-0.1752, -0.1245)
Residual (variance)	0.0600 (0.0574, 0.0627)	0.9575 (0.9180, 0.9988)	0.0007 (0.0006, 0.0008)	10.7772 (10.3277, 11.2462)	1.4429 (1.3814, 1.5071)	0.7146 (0.6834, 0.7472)

1 Index date was the date of diagnosis for cases and a randomly selected date in the patient’s study period for controls.

Abbreviations: RBC = red blood cells; Hb = haemoglobin; Hc = haematocrit; MCV = mean corpuscular volume; MCH = mean corpuscular haemoglobin; MCHC = mean corpuscular haemoglobin concentration.

**Table 15:** Mixed effect model coefficients for red blood cell count parameters (cases only) containing the Duke’s stage at diagnosis - females.

Variable	RBC	Hb	Hc	MCV	MCH	MCHC
*N*	1155	1213	332	1194	1134	1100
*n cases*	499	529	152	522	489	469
**Fixed effects:**						
Constant	4.6958 (3.7582, 5.6334)	12.6106 (9.0741, 16.1471)	0.4568 (0.2469, 0.6667)	77.7683 (66.4981, 89.0385)	26.8108 (22.9838, 30.6379)	33.4073 (31.2499, 35.5647)
Age at index date^1^ (years)	-0.0054 (-0.0221, 0.0114)	0.0046 (-0.0586, 0.0679)	-0.0012 (-0.0050, 0.0026)	0.1753 (-0.0262, 0.3769)	0.0408 (-0.0275, 0.1092)	-0.0123 (-0.0508, 0.0262)
Age at index date^1^ – knot at 60 (years)	0.0065 (-0.0224, 0.0355)	-0.0601 (-0.1692, 0.0491)	-0.0013 (-0.0078, 0.0053)	-0.4503 (-0.7993, -0.1013)	-0.1080 (-0.2259, 0.0098)	0.0091 (-0.0563, 0.0745)
Age at index date^1^ – knot at 70 (years)	-0.0251 (-0.0524, 0.0022)	-0.0546 (-0.1570, 0.0479)	0.0014 (-0.0049, 0.0076)	0.2975 (-0.0389, 0.6338)	0.0211 (-0.0926, 0.1348)	-0.0229 (-0.0836, 0.0378)
Age at index date^1^ – knot at 80 (years)	0.0205 (-0.0085, 0.0495)	0.1426 (0.0365, 0.2488)	0.0070 (0.0002, 0.0138)	0.0828 (-0.2991, 0.4647)	0.0892 (-0.0411, 0.2195)	0.0060 (-0.0618, 0.0739)
Time to index date^1^ (years)	-0.3456 (-1.4876, 0.7965)	-2.5994 (-6.8355, 1.6367)	-0.2053 (-0.5182, 0.1076)	3.3268 (2.5960, 4.0576)	1.2300 (0.9639, 1.4962)	0.4343 (0.2523, 0.6162)
Time to index date^1^ date – knot at one (years)	0.1585 (-2.0315, 2.3485)	3.2106 (-4.7104, 11.1316)	0.2821 (-0.2574, 0.8216)	-2.7413 (-3.9939, -1.4888)	-1.0680 (-1.5217, -0.6143)	-0.2886 (-0.5989, 0.0217)
Time to index date^1^ date – knot at two (years)	0.0743 (-1.6653, 1.8139)	-1.0014 (-7.1529, 5.1501)	-0.0790 (-0.4257, 0.2677)	-0.5796 (-1.4373, 0.2781)	-0.1513 (-0.4610, 0.1583)	-0.1554 (-0.3666, 0.0558)
Time to index date^1^ date – knot at four (years)	0.1521 (-0.7876, 1.0918)	0.1373 (-3.2328, 3.5075)	-0.0025 (-0.3025, 0.2975)	0.1417 (-0.2589, 0.5423)	0.0508 (-0.0943, 0.1960)	0.0769 (-0.0214, 0.1751)
Dukes D (vs. A)	-0.2849 (-1.3157, 0.7459)	0.6777 (-2.9215, 4.2769)	0.0177 (-0.1976, 0.2331)	3.1397 (-12.8396, 19.1190)	0.3363 (-5.1398, 5.8123)	-0.2814 (-3.3824, 2.8197)
Dukes D (vs. A) by time to index date^1^ interaction	0.2006 (0.1239, 0.2773)	1.0733 (0.7859, 1.3608)	0.0245 (0.0065, 0.0425)	1.6564 (0.5941, 2.7188)	0.8505 (0.4597, 1.2413)	0.3606 (0.0935, 0.6278)
Dukes D (vs. A) by time to index date^1^ (knot at one) interaction	-0.2790 (-0.4140, -0.1440)	-1.5478 (-2.0524, -1.0433)	-0.0226 (-0.0542, 0.0089)	-1.9403 (-3.8032, -0.0775)	-0.9683 (-1.6478, -0.2887)	-0.6136 (-1.0834, -0.1438)
Dukes D (vs. A) by time to index date^1^ (knot at two) interaction	0.0709 (-0.0236, 0.1654)	0.5693 (0.2189, 0.9197)	-0.0038 (-0.0256, 0.0180)	0.7481 (-0.5538, 2.0499)	0.3231 (-0.1483, 0.7946)	0.4441 (0.1153, 0.7729)
Dukes D (vs. A) by time to index date^1^ (knot at four) interaction	0.0386 (-0.0053, 0.0824)	-0.0479 (-0.2079, 0.1122)	0.0005 (-0.0109, 0.0118)	-0.7502 (-1.3526, -0.1477)	-0.3024 (-0.5215, -0.0832)	-0.2300 (-0.3835, -0.0765)
Time by age interaction	0.0082 (-0.0119, 0.0284)	0.0549 (-0.0201, 0.1299)	0.0040 (-0.0015, 0.0096)			
Time (knot at one) by age interaction	-0.0053 (-0.0437, 0.0330)	-0.0611 (-0.2004, 0.0783)	-0.0057 (-0.0153, 0.0039)			
Time (knot at two) by age interaction	-0.0004 (-0.0307, 0.0299)	0.0152 (-0.0924, 0.1227)	0.0018 (-0.0044, 0.0080)			
Time (knot at four) by age interaction	-0.0042 (-0.0206, 0.0123)	-0.0081 (-0.0673, 0.0510)	-0.0002 (-0.0054, 0.0050)			
Time by age (knot at 60) interaction	-0.0215 (-0.0539, 0.0110)	-0.0584 (-0.1808, 0.0639)	-0.0053 (-0.0145, 0.0040)			
Time (knot at one) by age (knot at 60) interaction	0.0241 (-0.0348, 0.0829)	0.0458 (-0.1729, 0.2646)	0.0109 (-0.0053, 0.0270)			
Time (knot at two) by age (knot at 60) interaction	-0.0070 (-0.0513, 0.0373)	-0.0004 (-0.1620, 0.1613)	-0.0058 (-0.0164, 0.0049)			
Time (knot at four) by age (knot at 60) interaction	0.0126 (-0.0114, 0.0365)	0.0428 (-0.0447, 0.1304)	0.0006 (-0.0065, 0.0077)			
Time by age (knot at 70) interaction	0.0266 (-0.0011, 0.0542)	0.0572 (-0.0477, 0.1621)	0.0012 (-0.0063, 0.0087)			
Time (knot at one) by age (knot at 70) interaction	-0.0204 (-0.0681, 0.0273)	0.0328 (-0.1478, 0.2133)	-0.0055 (-0.0185, 0.0075)			
Time (knot at two) by age (knot at 70) interaction	-0.0038 (-0.0367, 0.0292)	-0.0902 (-0.2144, 0.0341)	0.0033 (-0.0057, 0.0122)			
Time (knot at four) by age (knot at 70) interaction	-0.0115 (-0.0273, 0.0042)	-0.0464 (-0.1054, 0.0125)	0.0013 (-0.0030, 0.0056)			
Time by age (knot at 80) interaction	-0.0184 (-0.0432, 0.0064)	-0.1069 (-0.2011, -0.0128)	-0.0036 (-0.0095, 0.0023)			
Time (knot at one) by age (knot at 80) interaction	0.0009 (-0.0425, 0.0444)	0.0114 (-0.1529, 0.1756)	0.0034 (-0.0069, 0.0138)			
Time (knot at two) by age (knot at 80) interaction	0.0158 (-0.0142, 0.0459)	0.0992 (-0.0132, 0.2117)	0.0025 (-0.0047, 0.0097)			
Time (knot at four) by age (knot at 80) interaction	0.0097 (-0.0035, 0.0228)	0.0357 (-0.0130, 0.0843)	-0.0027 (-0.0064, 0.0010)			
Dukes D (vs. A) by age interaction	0.0010 (-0.0175, 0.0196)	-0.0334 (-0.0980, 0.0312)	-0.0006 (-0.0044, 0.0032)	-0.1143 (-0.4021, 0.1736)	-0.0245 (-0.1233, 0.0743)	-0.0005 (-0.0563, 0.0554)
Dukes D (vs. A) by age (knot at 60) interaction	0.0027 (-0.0308, 0.0363)	0.0653 (-0.0498, 0.1805)	-0.0016 (-0.0082, 0.0049)	0.3876 (-0.1364, 0.9116)	0.0511 (-0.1294, 0.2316)	-0.0243 (-0.1242, 0.0755)
Dukes D (vs. A) by age (knot at 70) interaction	-0.0003 (-0.0340, 0.0334)	-0.0143 (-0.1279, 0.0993)	0.0047 (-0.0018, 0.0112)	-0.4225 (-0.9505, 0.1055)	-0.0322 (-0.2135, 0.1490)	0.0490 (-0.0481, 0.1462)
Dukes D (vs. A) by age (knot at 80) interaction	-0.0032 (-0.0364, 0.0301)	-0.0517 (-0.1629, 0.0594)	-0.0071 (-0.0138, -0.0004)	-0.0146 (-0.5356, 0.5064)	-0.0571 (-0.2347, 0.1205)	-0.0383 (-0.1326, 0.0559)
**Random effects:**						
Intercept for patient (variance)	0.1515 (0.1361, 0.1685)	2.4850 (2.2428, 2.7534)	0.0019 (0.0015, 0.0024)	46.3906 (42.0590, 51.1684)	4.7755 (4.3019, 5.3013)	1.4257 (1.2693, 1.6013)
Slope for time to index date^1^ (variance)	0.0021 (0.0017, 0.0027)	0.0564 (0.0465, 0.0684)	0.0001 (0.0000, 0.0001)	0.8589 (0.6965, 1.0592)	0.0950 (0.0773, 0.1169)	0.0259 (0.0204, 0.0330)
Covariance between Intercept for patient and slope for time to index date (covariance)	-0.0097 (-0.0121, -0.0072)	-0.2864 (-0.3327, -0.2401)	-0.0003 (-0.0004, -0.0002)	-4.1096 (-4.8676, -3.3516)	-0.4127 (-0.4939, -0.3314)	-0.1432 (-0.1718, -0.1145)
Residual (variance)	0.0582 (0.0557, 0.0608)	0.8588 (0.8231, 0.8961)	0.0006 (0.0005, 0.0007)	11.9004 (11.3826, 12.4418)	1.4914 (1.4255, 1.5603)	0.6944 (0.6633, 0.7270)

1 Index date was the date of diagnosis for cases and a randomly selected date in the patient’s study period for controls.

Abbreviations: RBC = red blood cells; Hb = haemoglobin; Hc = haematocrit; MCV = mean corpuscular volume; MCH = mean corpuscular haemoglobin; MCHC = mean corpuscular haemoglobin concentration.

**Table 16:** Mixed effect model coefficients for platelet parameters (cases only) containing the Duke’s stage at diagnosis.

Variable	Males		Females	
	Platelets	MPV	Platelets	MPV
*N*	1528	211	1181	176
*n cases*	94	720	68	514
**Fixed effects:**				
Constant	362.7745 (244.9091, 480.6400)	10.1625 (9.0258, 11.2992)	228.8615 (80.0121, 377.7110)	9.3772 (8.2775, 10.4769)
Age at index date^1^ (years)	-1.6245 (-3.7115, 0.4624)	-0.0159 (-0.0322, 0.0003)	1.2336 (-1.4253, 3.8925)	-0.0065 (-0.0218, 0.0088)
Age at index date^1^ – knot at 60 (years)	1.0595 (-2.3577, 4.4768)		-1.0526 (-5.6207, 3.5154)	
Age at index date^1^ – knot at 70 (years)	2.4923 (-0.7897, 5.7742)		-0.7692 (-5.1260, 3.5876)	
Age at index date^1^ – knot at 80 (years)	-5.2987 (-9.9446, -0.6528)		3.0129 (-1.9186, 7.9443)	
Time to index date^1^ (years)	-23.8380 (-32.9754, -14.7006)	0.2090 (-0.1216, 0.5395)	-24.0242 (-33.9622, -14.0861)	0.0973 (-0.2211, 0.4158)
Time to index date^1^ date – knot at one (years)	23.2526 (7.2207, 39.2845)	-0.3958 (-0.9658, 0.1742)	21.6704 (4.6010, 38.7399)	-0.1026 (-0.6409, 0.4356)
Time to index date^1^ date – knot at two (years)	3.9099 (-7.2401, 15.0600)	0.1521 (-0.2582, 0.5624)	5.8590 (-5.7724, 17.4905)	-0.0305 (-0.3859, 0.3249)
Time to index date^1^ date – knot at four (years)	-4.1553 (-9.1666, 0.8560)	0.0945 (-0.1040, 0.2929)	-7.3941 (-12.7141, -2.0742)	0.0728 (-0.0984, 0.2441)
Dukes D (vs. A)	165.7902 (-11.5791, 343.1595)	-0.2669 (-0.7052, 0.1715)	316.5678 (104.2109, 528.9247)	-0.4065 (-0.8700, 0.0570)
Dukes D (vs. A) by time to index date^1^ interaction	-44.5130 (-57.1080, -31.9180)	-0.1359 (-0.5956, 0.3238)	-75.3165 (-89.7311, -60.9018)	0.1330 (-0.3165, 0.5825)
Dukes D (vs. A) by time to index date^1^ (knot at one) interaction	31.4208 (8.9414, 53.9002)	0.1574 (-0.6725, 0.9873)	89.7211 (64.4038, 115.0384)	-0.2428 (-1.0078, 0.5222)
Dukes D (vs. A) by time to index date^1^ (knot at two) interaction	7.8357 (-8.3477, 24.0191)	0.0784 (-0.5564, 0.7132)	-31.7440 (-49.3822, -14.1057)	0.2785 (-0.2305, 0.7875)
Dukes D (vs. A) by time to index date^1^ (knot at four) interaction	5.6127 (-1.9590, 13.1844)	-0.1989 (-0.5133, 0.1155)	22.9637 (14.9723, 30.9552)	-0.2549 (-0.5125, 0.0028)
Dukes D (vs. A) by age interaction	-1.6210 (-4.7706, 1.5287)		-3.8954 (-7.7167, -0.0740)	
Dukes D (vs. A) by age (knot at 60) interaction	2.6723 (-2.5642, 7.9088)		5.0388 (-1.8602, 11.9379)	
Dukes D (vs. A) by age (knot at 70) interaction	-4.3882 (-9.3424, 0.5659)		-1.5712 (-8.4503, 5.3079)	
Dukes D (vs. A) by age (knot at 80) interaction	7.9080 (1.9337, 13.8823)		-2.1388 (-8.8827, 4.6052)	
**Random effects:**				
Intercept for patient (variance)	7.0e+03 (6.4e+03, 7.7e+03)	1.5550 (1.2196, 1.9824)	8.4e+03 (7.6e+03, 9.2e+03)	1.4818 (1.1520, 1.9060)
Slope for time to index date^1^ (variance)	104.8360 (85.6861, 128.2657)	0.0265 (0.0159, 0.0441)	96.2314 (77.1465, 120.0376)	0.0194 (0.0121, 0.0312)
Covariance between Intercept for patient and slope for time to index date (covariance)	-6.7e+02 (-7.7e+02, -5.7e+02)	-0.1070 (-0.1661, -0.0478)	-6.6e+02 (-7.8e+02, -5.5e+02)	-0.0873 (-0.1350, -0.0396)
Residual (variance)	2.1e+03 (2.1e+03, 2.2e+03)	0.3302 (0.2892, 0.3770)	2.2e+03 (2.1e+03, 2.3e+03)	0.2551 (0.2274, 0.2862)

1 Index date was the date of diagnosis for cases and a randomly selected date in the patient’s study period for controls.

Abbreviations: MPV = mean platelet volume.

**Table 17:** Mixed effect model coefficients for white blood cell count parameters (cases only) containing the Duke’s stage at diagnosis – males.

Variable	WBC	Basophils	Eosinophils	Lymphocytes	Monocytes	Neutrophils
*N*	1466	1381	1385	1419	1414	1428
*n cases*	673	654	656	670	667	691
**Fixed effects:**						
Constant	7.7472 (3.6877, 11.8068)	0.0674 (0.0512, 0.0837)	0.0812 (-0.0790, 0.2414)	2.7383 (2.0743, 3.4024)	0.6958 (0.3986, 0.9931)	6.2180 (3.5621, 8.8738)
Age at index date^1^ (years)	-0.0077 (-0.0794, 0.0641)	-0.0000 (-0.0003, 0.0002)	0.0027 (0.0002, 0.0052)	-0.0131 (-0.0226, -0.0036)	-0.0017 (-0.0069, 0.0036)	-0.0321 (-0.0785, 0.0144)
Age at index date^1^ – knot at 60 (years)	-0.0008 (-0.1151, 0.1136)				0.0038 (-0.0046, 0.0122)	0.0565 (-0.0019, 0.1150)
Age at index date^1^ – knot at 70 (years)	0.0355 (-0.0571, 0.1282)		-0.0050 (-0.0097, -0.0003)		0.0007 (-0.0054, 0.0068)	
Age at index date^1^ – knot at 80 (years)				0.0120 (-0.0303, 0.0544)		-0.0239 (-0.1081, 0.0603)
Age at index date^1^ – knot at 85 (years)	-0.0098 (-0.3422, 0.3226)					
Age at index date^1^ – knot at 90 (years)						
Time to index date^1^ (years)	-0.3386 (-0.6635, -0.0137)	-0.0001 (-0.0010, 0.0009)	-0.0015 (-0.0046, 0.0015)	0.1539 (0.0033, 0.3044)	-0.0216 (-0.0636, 0.0205)	-0.3035 (-0.6557, 0.0487)
Time to index date^1^ date – knot at one (years)	0.4575 (-0.1122, 1.0271)			-0.1788 (-0.4439, 0.0864)	0.0066 (-0.0684, 0.0816)	0.2374 (-0.3948, 0.8697)
Time to index date^1^ date – knot at two (years)	-0.1188 (-0.5173, 0.2798)			0.0463 (-0.1407, 0.2333)	0.0040 (-0.0494, 0.0573)	0.1494 (-0.3053, 0.6040)
Time to index date^1^ date – knot at four (years)	-0.0243 (-0.2011, 0.1526)			-0.0019 (-0.0843, 0.0804)	0.0095 (-0.0139, 0.0328)	-0.1059 (-0.3126, 0.1009)
Dukes D (vs. A)	-1.9183 (-8.0906, 4.2541)	0.0025 (-0.0034, 0.0084)	0.0425 (-0.1947, 0.2797)	-0.0489 (-0.2636, 0.1657)	0.1502 (0.1103, 0.1902)	-0.4425 (-4.4857, 3.6007)
Dukes D (vs. A) by time to index date^1^ interaction	-1.3254 (-1.7719, -0.8789)	-0.0001 (-0.0015, 0.0013)	-0.0005 (-0.0050, 0.0040)	0.1569 (-0.0509, 0.3647)	-0.1632 (-0.2215, -0.1050)	-1.4907 (-1.9796, -1.0017)
Dukes D (vs. A) by time to index date^1^ (knot at one) interaction	1.0373 (0.2407, 1.8338)			-0.1110 (-0.4831, 0.2611)	0.2058 (0.1002, 0.3115)	1.4853 (0.5924, 2.3782)
CRC present by time to index date^1^ (knot at two) interaction	0.2554 (-0.3221, 0.8328)			-0.0800 (-0.3519, 0.1918)	-0.0628 (-0.1404, 0.0149)	-0.1088 (-0.7715, 0.5540)
Dukes D (vs. A) by time to index date^1^ (knot at four) interaction	0.0695 (-0.1985, 0.3374)			0.0279 (-0.0981, 0.1538)	0.0201 (-0.0155, 0.0557)	0.1268 (-0.1886, 0.4423)
Dukes D (vs. A) by age interaction	0.0721 (-0.0373, 0.1814)		-0.0009 (-0.0046, 0.0028)			0.0412 (-0.0294, 0.1117)
Dukes D (vs. A) by age (knot at 60) interaction	-0.1128 (-0.2888, 0.0632)					-0.0626 (-0.1508, 0.0256)
Dukes D (vs. A) by age (knot at 70) interaction	0.0054 (-0.1323, 0.1431)		0.0025 (-0.0040, 0.0090)			
Dukes D (vs. A) by age (knot at 80) interaction						0.0471 (-0.0571, 0.1513)
Dukes D (vs. A) by age (knot at 85) interaction	0.0766 (-0.2908, 0.4441)					
**Random effects:**						
Intercept for patient (variance)	8.5090 (7.7695, 9.3188)	0.0017 (0.0014, 0.0019)	0.0299 (0.0267, 0.0335)	2.9781 (2.7073, 3.2759)	0.0508 (0.0452, 0.0570)	1.7479 (1.4838, 2.0591)
Slope for time to index date^1^ (variance)	0.1028 (0.0806, 0.1311)	0.0000 (0.0000, 0.0000)	0.0002 (0.0001, 0.0003)	0.0111 (0.0073, 0.0170)	0.0003 (0.0002, 0.0005)	0.0550 (0.0419, 0.0722)
Covariance between Intercept for patient and slope for time to index date (covariance)	-0.7927 (-0.9275, -0.6580)	-0.0001 (-0.0002, -0.0001)	-0.0016 (-0.0021, -0.0010)	-0.1506 (-0.1994, -0.1018)	-0.0034 (-0.0043, -0.0024)	-0.1551 (-0.2124, -0.0979)
Residual (variance)	2.3451 (2.2429, 2.4520)	0.0016 (0.0015, 0.0017)	0.0213 (0.0203, 0.0223)	0.4792 (0.4578, 0.5016)	0.0421 (0.0403, 0.0440)	3.1919 (3.0522, 3.3380)

1 Index date was the date of diagnosis for cases and a randomly selected date in the patient’s study period for controls.

Abbreviations: WBC = white blood cell count.

**Table 18:** Mixed effect model coefficients for white blood cell count parameters (cases only) containing the Duke’s stage at diagnosis – females.

Variable	WBC	Basophils	Eosinophils	Lymphocytes	Monocytes	Neutrophils
*N*	1137	1055	1057	1073	1071	1077
*n cases*	471	462	463	470	468	494
**Fixed effects:**						
Constant	5.7967 (1.1308, 10.4626)	0.0685 (0.0518, 0.0853)	0.0165 (-0.1111, 0.1440)	2.0711 (1.6091, 2.5331)	0.5706 (0.3092, 0.8320)	5.5388 (3.2693, 7.8083)
Age at index date^1^ (years)	0.0229 (-0.0602, 0.1060)	0.0000 (-0.0002, 0.0002)	0.0029 (0.0009, 0.0050)	0.0010 (-0.0054, 0.0074)	-0.0008 (-0.0055, 0.0039)	-0.0186 (-0.0584, 0.0212)
Age at index date^1^ – knot at 60 (years)	0.0544 (-0.0831, 0.1920)				0.0066 (-0.0018, 0.0149)	0.0410 (-0.0101, 0.0921)
Age at index date^1^ – knot at 70 (years)	-0.0761 (-0.1823, 0.0300)		-0.0043 (-0.0078, -0.0007)		-0.0051 (-0.0132, 0.0030)	
Age at index date^1^ – knot at 80 (years)				-0.0381 (-0.0657, -0.0104)	0.0046 (-0.0031, 0.0122)	0.0323 (-0.0185, 0.0832)
Age at index date^1^ – knot at 85 (years)	0.0569 (-0.1531, 0.2669)					
Age at index date^1^ – knot at 90 (years)				0.1158 (0.0017, 0.2298)		
Time to index date^1^ (years)	-0.7255 (-1.2054, -0.2455)	-0.0010 (-0.0019, -0.0000)	-0.0024 (-0.0051, 0.0002)	-0.0529 (-0.1992, 0.0934)	-0.0489 (-0.0835, -0.0144)	-0.6451 (-1.0030, -0.2871)
Time to index date^1^ date – knot at one (years)	0.7098 (-0.1280, 1.5477)			-0.0228 (-0.2689, 0.2233)	0.0491 (-0.0109, 0.1091)	0.8206 (0.1925, 1.4486)
Time to index date^1^ date – knot at two (years)	0.1504 (-0.4295, 0.7304)			0.1343 (-0.0333, 0.3019)	-0.0121 (-0.0537, 0.0295)	-0.1369 (-0.5769, 0.3031)
Time to index date^1^ date – knot at four (years)	-0.2785 (-0.5369, -0.0200)			-0.0957 (-0.1750, -0.0164)	0.0089 (-0.0099, 0.0278)	-0.1555 (-0.3541, 0.0430)
Dukes D (vs. A)	7.2915 (0.7634, 13.8195)	-0.0047 (-0.0112, 0.0019)	0.1932 (0.0069, 0.3796)	-0.3276 (-0.5498, -0.1055)	0.1740 (0.1376, 0.2104)	1.6704 (1.3286, 2.0122)
Dukes D (vs. A) by time to index date^1^ interaction	-1.1431 (-1.8428, -0.4434)	0.0007 (-0.0008, 0.0021)	-0.0010 (-0.0049, 0.0029)	0.3316 (0.1189, 0.5443)	-0.1791 (-0.2293, -0.1288)	-1.3494 (-1.8709, -0.8280)
Dukes D (vs. A) by time to index date^1^ (knot at one) interaction	1.1213 (-0.1308, 2.3734)			-0.2867 (-0.6540, 0.0806)	0.1888 (0.0991, 0.2785)	1.1001 (0.1604, 2.0399)
CRC present by time to index date^1^ (knot at two) interaction	-0.1675 (-1.0561, 0.7210)			-0.0714 (-0.3286, 0.1857)	-0.0134 (-0.0772, 0.0504)	0.1630 (-0.5133, 0.8393)
Dukes D (vs. A) by time to index date^1^ (knot at four) interaction	0.3730 (-0.0208, 0.7668)			0.0909 (-0.0321, 0.2138)	0.0060 (-0.0230, 0.0351)	0.2539 (-0.0527, 0.5605)
Dukes D (vs. A) by age interaction	-0.0959 (-0.2133, 0.0215)		-0.0027 (-0.0056, 0.0002)			
Dukes D (vs. A) by age (knot at 60) interaction	0.0633 (-0.1418, 0.2685)					
Dukes D (vs. A) by age (knot at 70) interaction	0.0310 (-0.1353, 0.1973)		0.0025 (-0.0025, 0.0075)			
Dukes D (vs. A) by age (knot at 80) interaction						
Dukes D (vs. A) by age (knot at 85) interaction	0.0651 (-0.1961, 0.3264)					
**Random effects:**						
Intercept for patient (variance)	4.6264 (4.0525, 5.2815)	0.0017 (0.0015, 0.0020)	0.0188 (0.0167, 0.0212)	2.2505 (2.0433, 2.4788)	0.0356 (0.0314, 0.0403)	2.2038 (1.9020, 2.5535)
Slope for time to index date^1^ (variance)	0.0449 (0.0283, 0.0713)	0.0000 (0.0000, 0.0000)	0.0002 (0.0002, 0.0003)	0.1144 (0.1011, 0.1295)	0.0004 (0.0003, 0.0005)	0.0190 (0.0114, 0.0319)
Covariance between Intercept for patient and slope for time to index date (covariance)	-0.3046 (-0.4090, -0.2001)	-0.0001 (-0.0002, -0.0001)	-0.0014 (-0.0018, -0.0011)	-0.4654 (-0.5169, -0.4139)	-0.0028 (-0.0035, -0.0021)	-0.1071 (-0.1568, -0.0575)
Residual (variance)	5.1725 (4.9509, 5.4041)	0.0014 (0.0013, 0.0014)	0.0080 (0.0076, 0.0084)	0.3481 (0.3309, 0.3661)	0.0233 (0.0222, 0.0245)	2.7399 (2.6150, 2.8707)

1 Index date was the date of diagnosis for cases and a randomly selected date in the patient’s study period for controls.

Abbreviations: WBC = white blood cell count.

## Data Availability

The datasets used in this study are available from the CPRD. The CPRD maintain access rights to the data to ensure it is only used for research purposes by trustworthy organisations, so sharing of data is prohibited (https://cprd.com/data-access). Checks are conducted on organisations carrying out and funding research to assess whether they are suitable to receive CPRD data. This is to ensure, as examples, that the data stays confidential, and it is only used for its approved purpose. An application to access the data can be made at https://cprd.com/data-access. Additional full blood count parameters were analysed (tumour staging and sensitivity analysis) but are not related to the main claims in the article. These data are available from the authors on request.

## Plain english summary

Colorectal cancer is a common type of cancer in the UK. It is the second most common cause of cancer-related death in the UK. Chances of surviving depend heavily on the tumour stage at diagnosis, which represents how much the tumour has developed. If diagnosed and treated at the earliest stage, where the tumour is confined to the colon, nine in 10 patients are expected to be alive five years later. If diagnosed at the latest stage, when the cancer has spread outside the colon, this drops to one in 10 surviving. The majority of UK patients with colorectal cancer are diagnosed with late-stage tumours, so are likely to die. Detecting and treating the cancer earlier can save lives.

There is a blood test called the Full Blood Count, which is commonly ordered by doctors for many reasons. This test includes many blood levels, such as haemoglobin, which carries oxygen around the body. Growing tumours cause subtle changes in the blood levels over time, but it is unclear what these changes are and if they could help find cancer in the early stages.

In our study, we looked at blood tests from almost one million patients in the UK, including around 17,000 with colorectal cancer. We checked how blood levels change over 10 years before diagnosis. We found that in the few years before patients are diagnosed, patients usually had blood levels that rapidly started increasing or declining (depending on the blood level) and this was often not seen in patients without colorectal cancer. Our study highlights that using trends over time in blood test results may be useful to identify colorectal cancer. Such trends could facilitate earlier detection because they were present for years before diagnosis. That would improve the chances of successful treatment and chances of survival.

## *REVISED*: Amendments from Version 1

The biggest update made in this new version is the addition of Table 11 (hazard ratios from univariate joint models), which quantify the association between FBC trends and colorectal cancer diagnosis. A further update is that further details have been added to the Discussion regarding future work to build a prediction model using trends and how this prediction model could fit into the UK screening programme.

**Any further responses from the reviewers can be found at the end of the article**

## Open Peer Review

**Current Peer Review Status:**

**Version 2**

Reviewer Report 27 February 2023

https://doi.org/10.3310/nihropenres.14424.r28954

**© 2023 Opie J.** This is an open access peer review report distributed under the terms of the Creative Commons Attribution License, which permits unrestricted use, distribution, and reproduction in any medium, provided the original work is properly cited.

**Jessica J. Opie** https://orcid.org/0000-0002-1408-2604

Department of Pathology, Division of Haematology, National Health Laboratory Service, Groote Schuur Hospital, University of Cape Town, Cape Town, South Africa

This is a retrospective case-control study analyzing FBC trends in a large cohort of UK primary health care patients.

FBC findings in the group later diagnosed with colorectal cancer (CLC) were compared with a control group who did not have a later diagnosis of CLC. FBC results from the preceding 10 years were analysed, comparing males and females in both groups.

Expected trends associated with chronic blood loss and iron deficiency were detected in FBC parameters in the CLC group. Notably, only 48.8% of men and 54.3% of women were anaemic in the year prior to CLC diagnosis. This emphasizes that monitoring trends in FBC parameters (i.e. looking for a drop in Hb over time, even when the Hb is "normal" and within the RI) is important since an Hb within the reference range may not be "normal" for that particular patient, and may indicate underlying pathology and the need for further investigations.

In order to improve the screening model to detect CLC earlier in the UK primary health care setting, in addition to the FBC and fecal occult blood testing, I would recommend adding the serum ferritin, as a marker of iron stores (caveat: serum ferritin is also an acute phase reactant). Typically in iron deficiency, the serum ferritin will drop before the patient becomes anaemic, and thus would allow earlier CLC detection.

I have no corrections or edits to the current version.

Thank you for the opportunity to review.

**Is the work clearly and accurately presented and does it cite the current literature?**

Yes

**Is the study design appropriate and is the work technically sound?**

Yes

**Are sufficient details of methods and analysis provided to allow replication by others?**

Yes

**If applicable, is the statistical analysis and its interpretation appropriate?**

I cannot comment. A qualified statistician is required.

**Are all the source data underlying the results available to ensure full reproducibility?**

Yes

**Are the conclusions drawn adequately supported by the results?**

Yes

***Reviewer Expertise:*** I am a practicing Haematology Pathologist [MBChB, MRCP, FRCPA (Haematology)] with broad research interests including automation and instrumentation, coagulation, anaemia, HIV-associated lymphoma and thrombosis.

**I confirm that I have read this submission and believe that I have an appropriate level of expertise to confirm that it is of an acceptable scientific standard.**

Reviewer Report 14 November 2022

https://doi.org/10.3310/nihropenres.14424.r28835

**© 2022 Gunnarsson U.** This is an open access peer review report distributed under the terms of the Creative Commons Attribution License, which permits unrestricted use, distribution, and reproduction in any medium, provided the original work is properly cited.

**Ulf Gunnarsson**

Department of Surgical and Perioperative Sciences, Surgery, Umeå University, Umeå, Sweden

The authors have improved their manuscript and adequately addressed most issues raised by the referees. However, one may still wonder, approximately which proportion of a population without alarming symptoms will be subject to a FIT-test as a result of FBC-monitoring? A proportion of these patients will thereafter also be subject to a colonoscopy, probably with a lower proportion of “positive” cases than when symptomatic patients are refereed via the “fast track” pathway, thus adding a burden to the health care system.

**Is the work clearly and accurately presented and does it cite the current literature?**

Yes

**Is the study design appropriate and is the work technically sound?**

Yes

**Are sufficient details of methods and analysis provided to allow replication by others?**

Yes

**If applicable, is the statistical analysis and its interpretation appropriate?**

Yes

**Are all the source data underlying the results available to ensure full reproducibility?**

Yes

**Are the conclusions drawn adequately supported by the results?**

Yes

***Reviewer Expertise:*** Colorectal cancer

**I confirm that I have read this submission and believe that I have an appropriate level of expertise to confirm that it is of an acceptable scientific standard.**

**Version 1**

Reviewer Report 07 June 2022

https://doi.org/10.3310/nihropenres.14385.r28638

**© 2022 Morrison D.** This is an open access peer review report distributed under the terms of the Creative Commons Attribution License, which permits unrestricted use, distribution, and reproduction in any medium, provided the original work is properly cited.

**David Morrison**

Institute of Health and Wellbeing, University of Glasgow, Glasgow, UK

I am providing a brief initial response at this stage.

The study is described as being "a retrospective case-control study". Case-control studies are by nature retrospective (the cases having already occurred) so it is not necessary to use this term. But I suspect it is not a case-control study. I am looking for a definition of cases and the methodology for identifying controls but I can't see it. This is crucial to judging the quality of the study as case-control studies are usually said to be inherently biased - the criteria for case selection and methodology for control selection are therefore critical. Hence, there are fundamental questions about the design of this study that preclude further assessment.

Case-control studies do not measure risks or rates (risk ratios may be inferred from odds ratios, but they are not directly measured), so the inclusion of plots of events over time makes me think this is in fact some form of a longitudinal study. It is probably not a cohort study, because there don't appear to be cohorts defined by FBC at the start of follow-up.

I won't suggest more at this stage but invite the authors to provide more information on the methodology to make it clear and unambiguous so that its merits can be assessed.

**Is the work clearly and accurately presented and does it cite the current literature?**

Yes

**Is the study design appropriate and is the work technically sound?**

No

**Are sufficient details of methods and analysis provided to allow replication by others?**

No

**If applicable, is the statistical analysis and its interpretation appropriate?**

No

**Are all the source data underlying the results available to ensure full reproducibility?**

Yes

**Are the conclusions drawn adequately supported by the results?**

No

***Reviewer Expertise:*** Epidemiology, cancer, population-based studies, obesity.

**I confirm that I have read this submission and believe that I have an appropriate level of expertise to state that I do not consider it to be of an acceptable scientific standard, for reasons outlined above.**

## Author Response 01 Oct 2022

**Pradeep Virdee**, University of Oxford, Oxford, UK

Dear reviewer,

Thank you for your comments on our manuscript. We have provided responses to your comments below:

Comment: The study is described as being "a retrospective case-control study". Case-control studies are by nature retrospective (the cases having already occurred) so it is not necessary to use this term. But I suspect it is not a case-control study. I am looking for a definition of cases and the methodology for identifying controls but I can't see it. This is crucial to judging the quality of the study as case-control studies are usually said to be inherently biased - the criteria for case selection and methodology for control selection are therefore critical. Hence, there are fundamental questions about the design of this study that preclude further assessment.

**Response:** Thanks for your comments. A definition of cases and controls and the methodology for identifying controls is already provided in the Methods, Outcome section.

Comment: Case-control studies do not measure risks or rates (risk ratios may be inferred from odds ratios, but they are not directly measured), so the inclusion of plots of events over time makes me think this is in fact some form of a longitudinal study. It is probably not a cohort study, because there don't appear to be cohorts defined by FBC at the start of follow-up.

**Response:** We agree that case-control studies do not measure (absolute) risk. However, identifying risk was not the aim of this paper – this study describes patterns or trends in FBC results over time in patients with colorectal cancer that were not seen in patients without colorectal cancer, we have not included any risks in this paper.

Apologies if we misunderstood, but we are unsure of your point regarding plots of events over time – we have not included any plots of diagnoses over time. We have however included plots of FBC test results over time, if that’s what you meant?

We describe this as a longitudinal study in the title but have now added it to the study design section too, thanks for pointing that out.

You are right that this is not a cohort study, as we have no defined cohort. This is a case-control analysis, in which we take all cases and look back over time before the diagnosis to identify trends in FBC results and compare these trends to those in controls (patients not diagnosed).

## Reviewer Report 16 May 2022

https://doi.org/10.3310/nihropenres.14385.r28580

**© 2022 Gunnarsson U.** This is an open access peer review report distributed under the terms of the Creative Commons Attribution License, which permits unrestricted use, distribution, and reproduction in any medium, provided the original work is properly cited.

**Ulf Gunnarsson**

Department of Surgical and Perioperative Sciences, Surgery, Umeå University, Umeå, Sweden

This is a formally well-written manuscript describing the time trend in Full Blood Cell Count (FBC) for patients with detection of a colorectal cancer. Statistical calculations and presentation of results seem to be technically correct. The main result is an “increasing” divergence from controls the four years preceding the detection of a colorectal cancer and the authors describe a future model for an automatised indication when enough of the aberrations in FBC described are present. As such, the idea is innovative since FBC is often used as a routine sample when assessing different complaints in primary health care. However, the manuscript would gain from the improvement of some shortcomings in the presentation:

1. In the Introduction, the authors use the modern term TNM stage while in the presentation of their own data stage is given as Dukes. Even if their data are old, it is usually easy to translate Dukes stage to the modern TNM standard.
2. Data used seem to be rather old – as far as I can see, gathered until 2014 and thus 8 years old. What was the reason for this? Probably this is also the reason for labelling stage with the Dukes classification?
3. The manuscript is in its present form just descriptive and there are seemingly no attempts to calculate the capacity to discriminate cases with colorectal cancer from those without. The study would gain substantially from some kind of model for calculation of sensitivity/specificity and likelihood ratio which must be a requisite for further tests in clinical practice. As a minimum, this perspective must be carefully addressed in the Discussion section.
4. Today, as far as I know, the UK uses a fast-track system for patients with symptoms indicating a colorectal cancer. The system described in this manuscript seems to be merely intended to detect suspected cases before the occurrence of alarming symptoms. Thus, the manuscript would also gain from a description of how to handle such cases without alarming symptoms. Furthermore, this handling must be based on the anticipated number of such cases.
5. The manuscript would also gain from a discussion of the proposed system against FIT and FOBT tests frequently used in primary health care. One obvious difference is, of course, that such tests are used for symptomatic patients while the proposed system will act when no suspicion is raised - but also, this scenario has to be carefully addressed to put the study into a routine clinical context.

**Is the work clearly and accurately presented and does it cite the current literature?**

Partly

**Is the study design appropriate and is the work technically sound?**

Yes

**Are sufficient details of methods and analysis provided to allow replication by others?**

Yes

**If applicable, is the statistical analysis and its interpretation appropriate?**

Yes

**Are all the source data underlying the results available to ensure full reproducibility?**

Yes

**Are the conclusions drawn adequately supported by the results?**

Partly

***Reviewer Expertise:*** Colorectal cancer

**I confirm that I have read this submission and believe that I have an appropriate level of expertise to confirm that it is of an acceptable scientific standard, however I have significant reservations, as outlined above.**

## Author Response 01 Oct 2022

**Pradeep Virdee**, University of Oxford, Oxford, UK

Dear reviewer,

Thank you for your comments on the manuscript. We have provided responses to each point below:

Comment: In the Introduction, the authors use the modern term TNM stage while in the presentation of their own data stage is given as Dukes. Even if their data are old, it is usually easy to translate Dukes stage to the modern TNM standard.

**Response:** Thanks for pointing this out. We have replaced TNM staging with Duke’s stage in the Introduction. The Duke’s staging system was provided by CPRD in our dataset so we prefer to use this rather than translate everything to TNM.

Comment: Data used seem to be rather old – as far as I can see, gathered until 2014 and thus 8 years old. What was the reason for this? Probably this is also the reason for labelling stage with the Dukes classification?

**Response:** The dataset used here was available from a prior study. Although the data is 8 years old, this would not affect the overall results or conclusions made. For example, we have analysed only routinely available blood test data, for which there is little-to-no change in practice over the last 8 years that would influence the current results. As mentioned, Duke’s was the only staging system provided to us by CPRD.

Comment: The manuscript is in its present form just descriptive and there are seemingly no attempts to calculate the capacity to discriminate cases with colorectal cancer from those without. The study would gain substantially from some kind of model for calculation of sensitivity/specificity and likelihood ratio which must be a requisite for further tests in clinical practice. As a minimum, this perspective must be carefully addressed in the Discussion section.

**Response:** You are right that we only describe the FBC trends and illustrate the difference between patients with and without colorectal cancer using graphs. The aim of this paper was to show that relevant trends exist and that these could be more helpful than single FBC tests and relying on symptoms for early detection (hence why we compared trends to FBC reference ranges and microcytic anaemia presence). You are right that a model for the calculation of performance measures would be important, and this is already considered a next step and described in the Discussion, Implications for practice section. It has not been described here because this is outside the remit of the paper, which is to describe patterns and trends in repeated FBCs in patients with and without colorectal cancer, not predictive ability. We have however added Table 11 to provide statistical inferences between trends and diagnosis, which reports hazard ratios for the effect of patient-level trends on colorectal cancer presence (derived using joint modelling), but not performance measures, which would be more appropriately derived using cohort study designs (again, this is a next step).

Comment: Today, as far as I know, the UK uses a fast-track system for patients with symptoms indicating a colorectal cancer. The system described in this manuscript seems to be merely intended to detect suspected cases before the occurrence of alarming symptoms. Thus, the manuscript would also gain from a description of how to handle such cases without alarming symptoms. Furthermore, this handling must be based on the anticipated number of such cases.

**Response:** Suspected cases without alarming symptoms could be offered a FIT test (to identify any rectal bleeding), which is slowly making its way for use outside the screening programme in patients with low-risk symptoms. FIT testing is cheaper and has less burden on patients and healthcare services than colonoscopy. Therefore, patients identified through our system who subsequently have a positive FIT result could then be referred for further testing under the fast-track system. I have added this into the Discussion, Implications for practice section.

Comment: The manuscript would also gain from a discussion of the proposed system against FIT and FOBT tests frequently used in primary health care. One obvious difference is, of course, that such tests are used for symptomatic patients while the proposed system will act when no suspicion is raised - but also, this scenario has to be carefully addressed to put the study into a routine clinical context.

**Response:** Thanks, another good point. We do not envisage that using FBC trends would replace FIT testing. In fact, we believe FBC trends could complement the use of FIT – as described in the previous response, patients identified through our system could be offered a FIT test (outside the screening programme) and if positive, would warrant fast-track referral.
