# Supplementary material for: Trends in the full blood count blood test and colorectal cancer detection: a longitudinal, case-control study of UK primary care patient data
Source: NIHR Open Res. 2022 Oct 18;2:32. Originally published 2022 Apr 21. [Version 2] doi: 10.3310/nihropenres.13266.2 (PMC7614427; doi:10.3310/nihropenres.13266.2)
Supplement: Peer review history

# Reviewer report: Reviewer response for version 2

**DOI:** 10.3310/nihropenres.14424.r28954

**Published:** 2023-02-27

**Authors:** Jessica J. Opie

**Affiliations:** 1 Department of Pathology, Division of Haematology, National Health Laboratory Service, Groote Schuur Hospital, University of Cape Town, Cape Town, South Africa

This is a retrospective case-control study analyzing FBC trends in a large cohort of UK primary health care patients.

 FBC findings in the group later diagnosed with colorectal cancer (CLC) were compared with a control group who did not have a later diagnosis of CLC. FBC results from the preceding 10 years were analysed, comparing males and females in both groups.  

 Expected trends associated with chronic blood loss and iron deficiency were detected in FBC parameters in the CLC group. Notably, only 48.8% of men and 54.3% of women were anaemic in the year prior to CLC diagnosis. This emphasizes that monitoring trends in FBC parameters (i.e. looking for a drop in Hb over time, even when the Hb is "normal" and within the RI) is important since an Hb within the reference range may not be "normal" for that particular patient, and may indicate underlying pathology and the need for further investigations. 

 In order to improve the screening model to detect CLC earlier in the UK primary health care setting, in addition to the FBC and fecal occult blood testing, I would recommend adding the serum ferritin, as a marker of iron stores (caveat: serum ferritin is also an acute phase reactant). Typically in iron deficiency, the serum ferritin will drop before the patient becomes anaemic, and thus would allow earlier CLC detection.  

 I have no corrections or edits to the current version. 

 Thank you for the opportunity to review.

Is the work clearly and accurately presented and does it cite the current literature?

Yes

If applicable, is the statistical analysis and its interpretation appropriate?

I cannot comment. A qualified statistician is required.

Are all the source data underlying the results available to ensure full reproducibility?

Yes

Is the study design appropriate and is the work technically sound?

Yes

Are the conclusions drawn adequately supported by the results?

Yes

Are sufficient details of methods and analysis provided to allow replication by others?

Yes

Reviewer Expertise:

I am a practicing Haematology Pathologist [MBChB, MRCP,  FRCPA (Haematology)] with broad research interests including automation and instrumentation, coagulation, anaemia,  HIV-associated lymphoma and thrombosis.

I confirm that I have read this submission and believe that I have an appropriate level of expertise to confirm that it is of an acceptable scientific standard.

# Reviewer report: Reviewer response for version 2

**DOI:** 10.3310/nihropenres.14424.r28835

**Published:** 2022-11-14

**Authors:** Ulf Gunnarsson

**Affiliations:** 1 Department of Surgical and Perioperative Sciences, Surgery, Umeå University, Umeå, Sweden

The authors have improved their manuscript and adequately addressed most issues raised by the referees. However, one may still wonder, approximately which proportion of a population without alarming symptoms will be subject to a FIT-test as a result of FBC-monitoring? A proportion of these patients will thereafter also be subject to a colonoscopy, probably with a lower proportion of “positive” cases than when symptomatic patients are refereed via the “fast track” pathway, thus adding a burden to the health care system.

Is the work clearly and accurately presented and does it cite the current literature?

Partly

If applicable, is the statistical analysis and its interpretation appropriate?

Yes

Are all the source data underlying the results available to ensure full reproducibility?

Yes

Is the study design appropriate and is the work technically sound?

Yes

Are the conclusions drawn adequately supported by the results?

Partly

Are sufficient details of methods and analysis provided to allow replication by others?

Yes

Reviewer Expertise:

Colorectal cancer

I confirm that I have read this submission and believe that I have an appropriate level of expertise to confirm that it is of an acceptable scientific standard.

# Reviewer report: Reviewer response for version 1

**DOI:** 10.3310/nihropenres.14385.r28638

**Published:** 2022-06-07

**Authors:** David Morrison

**Affiliations:** 1 Institute of Health and Wellbeing, University of Glasgow, Glasgow, UK

I am providing a brief initial response at this stage.

 The study is described as being "a retrospective case-control study". Case-control studies are by nature retrospective (the cases having already occurred) so it is not necessary to use this term. But I suspect it is not a case-control study. I am looking for a definition of cases and the methodology for identifying controls but I can't see it. This is crucial to judging the quality of the study as case-control studies are usually said to be inherently biased - the criteria for case selection and methodology for control selection are therefore critical. Hence, there are fundamental questions about the design of this study that preclude further assessment. 

 Case-control studies do not measure risks or rates (risk ratios may be inferred from odds ratios, but they are not directly measured), so the inclusion of plots of events over time makes me think this is in fact some form of a longitudinal study. It is probably not a cohort study, because there don't appear to be cohorts defined by FBC at the start of follow-up. 

 I won't suggest more at this stage but invite the authors to provide more information on the methodology to make it clear and unambiguous so that its merits can be assessed.

Is the work clearly and accurately presented and does it cite the current literature?

Yes

If applicable, is the statistical analysis and its interpretation appropriate?

No

Are all the source data underlying the results available to ensure full reproducibility?

Yes

Is the study design appropriate and is the work technically sound?

No

Are the conclusions drawn adequately supported by the results?

No

Are sufficient details of methods and analysis provided to allow replication by others?

No

Reviewer Expertise:

Epidemiology, cancer, population-based studies, obesity.

I confirm that I have read this submission and believe that I have an appropriate level of expertise to state that I do not consider it to be of an acceptable scientific standard, for reasons outlined above.

# Response

**Published:** 2022-10-01

**Authors:** Pradeep Virdee

**Affiliations:** University of Oxford, UK

Dear reviewer,

 Thank you for your comments on our manuscript. We have provided responses to your comments below:

 Comment: The study is described as being "a retrospective case-control study". Case-control studies are by nature retrospective (the cases having already occurred) so it is not necessary to use this term. But I suspect it is not a case-control study. I am looking for a definition of cases and the methodology for identifying controls but I can't see it. This is crucial to judging the quality of the study as case-control studies are usually said to be inherently biased - the criteria for case selection and methodology for control selection are therefore critical. Hence, there are fundamental questions about the design of this study that preclude further assessment. 

**Response: **Thanks for your comments. A definition of cases and controls and the methodology for identifying controls is already provided in the Methods, Outcome section.

 Comment: Case-control studies do not measure risks or rates (risk ratios may be inferred from odds ratios, but they are not directly measured), so the inclusion of plots of events over time makes me think this is in fact some form of a longitudinal study. It is probably not a cohort study, because there don't appear to be cohorts defined by FBC at the start of follow-up. 

**Response:** We agree that case-control studies do not measure (absolute) risk. However, identifying risk was not the aim of this paper – this study describes patterns or trends in FBC results over time in patients with colorectal cancer that were not seen in patients without colorectal cancer, we have not included any risks in this paper.

 Apologies if we misunderstood, but we are unsure of your point regarding plots of events over time – we have not included any plots of diagnoses over time. We have however included plots of FBC test results over time, if that’s what you meant?

 We describe this as a longitudinal study in the title but have now added it to the study design section too, thanks for pointing that out.

 You are right that this is not a cohort study, as we have no defined cohort. This is a case-control analysis, in which we take all cases and look back over time before the diagnosis to identify trends in FBC results and compare these trends to those in controls (patients not diagnosed).

# Reviewer report: Reviewer response for version 1

**DOI:** 10.3310/nihropenres.14385.r28580

**Published:** 2022-05-16

**Authors:** Ulf Gunnarsson

**Affiliations:** 1 Department of Surgical and Perioperative Sciences, Surgery, Umeå University, Umeå, Sweden

This is a formally well-written manuscript describing the time trend in Full Blood Cell Count (FBC) for patients with detection of a colorectal cancer. Statistical calculations and presentation of results seem to be technically correct. The main result is an “increasing” divergence from controls the four years preceding the detection of a colorectal cancer and the authors describe a future model for an automatised indication when enough of the aberrations in FBC described are present. As such, the idea is innovative since FBC is often used as a routine sample when assessing different complaints in primary health care. However, the manuscript would gain from the improvement of some shortcomings in the presentation:

1. In the Introduction, the authors use the modern term TNM stage while in the presentation of their own data stage is given as Dukes. Even if their data are old, it is usually easy to translate Dukes stage to the modern TNM standard.
2. Data used seem to be rather old – as far as I can see, gathered until 2014 and thus 8 years old. What was the reason for this? Probably this is also the reason for labelling stage with the Dukes classification?
3. The manuscript is in its present form just descriptive and there are seemingly no attempts to calculate the capacity to discriminate cases with colorectal cancer from those without. The study would gain substantially from some kind of model for calculation of sensitivity/specificity and likelihood ratio which must be a requisite for further tests in clinical practice. As a minimum, this perspective must be carefully addressed in the Discussion section.
4. Today, as far as I know, the UK uses a fast-track system for patients with symptoms indicating a colorectal cancer. The system described in this manuscript seems to be merely intended to detect suspected cases before the occurrence of alarming symptoms. Thus, the manuscript would also gain from a description of how to handle such cases without alarming symptoms. Furthermore, this handling must be based on the anticipated number of such cases.
5. The manuscript would also gain from a discussion of the proposed system against FIT and FOBT tests frequently used in primary health care. One obvious difference is, of course, that such tests are used for symptomatic patients while the proposed system will act when no suspicion is raised - but also, this scenario has to be carefully addressed to put the study into a routine clinical context.

Is the work clearly and accurately presented and does it cite the current literature?

Partly

If applicable, is the statistical analysis and its interpretation appropriate?

Yes

Are all the source data underlying the results available to ensure full reproducibility?

Yes

Is the study design appropriate and is the work technically sound?

Yes

Are the conclusions drawn adequately supported by the results?

Partly

Are sufficient details of methods and analysis provided to allow replication by others?

Yes

Reviewer Expertise:

Colorectal cancer

I confirm that I have read this submission and believe that I have an appropriate level of expertise to confirm that it is of an acceptable scientific standard, however I have significant reservations, as outlined above.

# Response

**Published:** 2022-10-01

**Authors:** Pradeep Virdee

**Affiliations:** University of Oxford, UK

Dear reviewer, 

 Thank you for your comments on the manuscript. We have provided responses to each point below:

 Comment: In the Introduction, the authors use the modern term TNM stage while in the presentation of their own data stage is given as Dukes. Even if their data are old, it is usually easy to translate Dukes stage to the modern TNM standard.

**Response:** Thanks for pointing this out. We have replaced TNM staging with Duke’s stage in the Introduction. The Duke’s staging system was provided by CPRD in our dataset so we prefer to use this rather than translate everything to TNM.

 Comment: Data used seem to be rather old – as far as I can see, gathered until 2014 and thus 8 years old. What was the reason for this? Probably this is also the reason for labelling stage with the Dukes classification?

**Response: **The dataset used here was available from a prior study. Although the data is 8 years old, this would not affect the overall results or conclusions made. For example, we have analysed only routinely available blood test data, for which there is little-to-no change in practice over the last 8 years that would influence the current results. As mentioned, Duke’s was the only staging system provided to us by CPRD.

 Comment: The manuscript is in its present form just descriptive and there are seemingly no attempts to calculate the capacity to discriminate cases with colorectal cancer from those without. The study would gain substantially from some kind of model for calculation of sensitivity/specificity and likelihood ratio which must be a requisite for further tests in clinical practice. As a minimum, this perspective must be carefully addressed in the Discussion section.

**Response: **You are right that we only describe the FBC trends and illustrate the difference between patients with and without colorectal cancer using graphs. The aim of this paper was to show that relevant trends exist and that these could be more helpful than single FBC tests and relying on symptoms for early detection (hence why we compared trends to FBC reference ranges and microcytic anaemia presence). You are right that a model for the calculation of performance measures would be important, and this is already considered a next step and described in the Discussion, Implications for practice section. It has not been described here because this is outside the remit of the paper, which is to describe patterns and trends in repeated FBCs in patients with and without colorectal cancer, not predictive ability. We have however added Table 11 to provide statistical inferences between trends and diagnosis, which reports hazard ratios for the effect of patient-level trends on colorectal cancer presence (derived using joint modelling), but not performance measures, which would be more appropriately derived using cohort study designs (again, this is a next step). 

 Comment: Today, as far as I know, the UK uses a fast-track system for patients with symptoms indicating a colorectal cancer. The system described in this manuscript seems to be merely intended to detect suspected cases before the occurrence of alarming symptoms. Thus, the manuscript would also gain from a description of how to handle such cases without alarming symptoms. Furthermore, this handling must be based on the anticipated number of such cases.

**Response:** Suspected cases without alarming symptoms could be offered a FIT test (to identify any rectal bleeding), which is slowly making its way for use outside the screening programme in patients with low-risk symptoms. FIT testing is cheaper and has less burden on patients and healthcare services than colonoscopy. Therefore, patients identified through our system who subsequently have a positive FIT result could then be referred for further testing under the fast-track system. I have added this into the Discussion, Implications for practice section.

 Comment: The manuscript would also gain from a discussion of the proposed system against FIT and FOBT tests frequently used in primary health care. One obvious difference is, of course, that such tests are used for symptomatic patients while the proposed system will act when no suspicion is raised - but also, this scenario has to be carefully addressed to put the study into a routine clinical context.

**Response:** Thanks, another good point. We do not envisage that using FBC trends would replace FIT testing. In fact, we believe FBC trends could complement the use of FIT – as described in the previous response, patients identified through our system could be offered a FIT test (outside the screening programme) and if positive, would warrant fast-track referral.
